# Thiazide and other Cl-benzenesulfonamide-bearing clinical drug affinities for human carbonic anhydrases

**DOI:** 10.1371/journal.pone.0253608

**Published:** 2021-06-24

**Authors:** Lina Baranauskiene, Lina Škiudaitė, Vilma Michailovienė, Vytautas Petrauskas, Daumantas Matulis

**Affiliations:** 1 Department of Biothermodynamics and Drug Design, Institute of Biotechnology, Life Sciences Center, Vilnius University, Vilnius, Lithuania; 2 Pharmacy Center, Institute of Biomedical Science, Faculty of Medicine, Vilnius University, Vilnius, Lithuania; Griffith University, AUSTRALIA

## Abstract

Twelve carbonic anhydrase (CA) isoforms catalyze carbon dioxide hydration to bicarbonate and acid protons and are responsible for many biological functions in human body. Despite their vital functions, they are also responsible for, or implicated in, numerous ailments and diseases such as glaucoma, high altitude sickness, and cancer. Because CA isoforms are highly homologous, clinical drugs designed to inhibit enzymatic activity of a particular isoform, can also bind to others with similar affinity causing toxic side effects. In this study, the affinities of twelve CA isoforms have been determined for nineteen clinically used drugs used to treat hypertension related diseases, i.e. thiazides, indapamide, and metolazone. Their affinities were determined using a fluorescent thermal shift assay. Stopped flow assay and isothermal titration calorimetry were also employed on a subset of compounds and proteins to confirm inhibition of CA enzymatic activity and verify the quantitative agreement between different assays. The findings of this study showed that pharmaceuticals could bind to human CA isoforms with variable affinities and inhibit their catalytic activity, even though the drug was intended to interact with a different (non-CA) protein target. Relatively minor structural changes of the compounds may cause significant changes in affinity and selectivity for a particular CA isoform.

## Introduction

Pharmaceuticals are designed to bind target proteins selectively; however, in practice, they often bind to other non-intended targetscausing undesired side effects. Thiazide and other chloro-benzenesulfonamide-bearing drugs are used as diuretics to treat hypertension. While the mechanism of lowering blood pressure is still poorly understood, the most likely targets are ion transporters [1, 2], although ion channels, carbonic anhydrases (CA), and other proteins could also play a role [1, 2]. The inhibition of CA by some members of this compound class has been demonstrated [3]. These enzymes contain zinc in their active sites and are involved in the progression of several electrolyte balance-linked diseases, such as glaucoma, edema, epilepsy, mountain sickness, osteoporosis, obesity, infertility, and cancer. The inhibition of CA using small molecule therapeutics is used to treat some of these disorders. Humans have twelve catalytically active CA isoforms containing structurally similar catalytic domains but different enzymatic activity, tissue distribution, and cellular localization [4, 5].

The last human carbonic anhydrase isoform, CA XIV, was identified in 1999 [6], and the bioinformatic analysis of the human genome confirmed that there are no other encoded CAs [7]. Because of the structural similarity at the catalytic site, sulfonamides can bind to more than one CA isoform. Given the majority of sulfonamide drugs entered the pharmaceutical market several decades before all CA isoforms were identified, their selectivity and specificity have not been determined against all known CA isoforms. This investigation addresses this shortfall.

**Fig 1:**
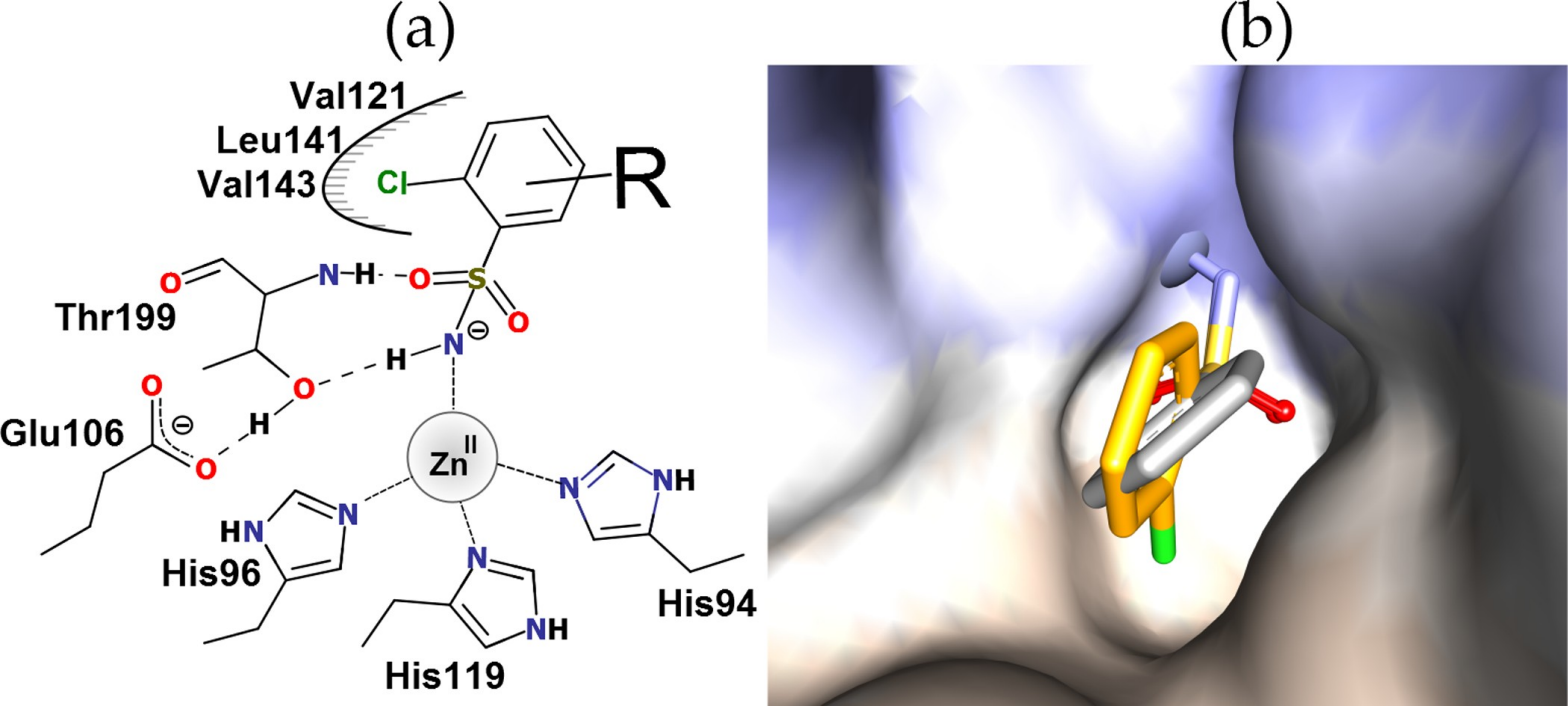
The interactions between CA II and the 2-chlorobenzenesulfonamide-bearing compound. Panel (a): The negatively charged amino group of the sulfonamide forms a coordination bond with the Zn(II) in the active site and prevents the CO_2_ substrate binding. Additional R substituents form hydrophobic and other contacts with the protein amino acids. Panel (b): The chlorine (green) of the 2-chloro-benzenesulfonamide (orange) occupies the binding site cavity and orients the compound in the active site of CA II as compared to the benzenesulfonamide (grey) (PDB IDs 2WEH and 2WEJ [10]).

The unsubstituted sulfonamide group is considered to be non-toxic. It is often used in medicinal chemistry for specific binding orientation and a solubility-enhancing group with an H-bond acceptor/donor [8]. The deprotonated sulfonamide NH^-^ group coordinates the zinc ion after displacing the zinc-bound water molecule and donates a hydrogen bond to the OH group of Thr199, while the sulfonamide S = O group accepts the H-bond from the same Thr199 backbone nitrogen (Fig 1A). The unsubstituted sulfonamide group acts as an "anchor" in the active site of CA, while the rest of the molecule strongly affects the binding affinity and selectivity for different CA isoforms [8, 9]. Substituents of the aromatic ring near the zinc ion can orient the inhibitor molecule within the CA active site pocket. For example, Cl substituent in 2-chlorobenzenesulfonamide fills the hydrophobic cavity formed by Val121, Leu141, and Val143 and rotates the benzene ring (Fig 1B) [10]. A similar position of the benzene ring is maintained in other compounds with Cl substituent in the ortho- position [11–13]. Besides, the Cl substituent has a significant impact on the *pK*_a_ of the sulfonamide amino group, thus increasing the affinity.

In this study, we used a group of compounds bearing the 2-chlorobenzenesulfonamide (or 2-trifluorobenzenesulfonamide) fragment in their structure that have been used as pharmaceutical drugs to treat different diseases in humans. We determined their binding to all twelve catalytically active human CA isoforms. Some of these compounds have been previously shown to bind several CA isoforms with nanomolar affinities inhibiting their function in the human body. However, binding affinities have never been determined for all twelve CA isoforms. The complete drug selectivity profile is possible only if a full set of affinities between all active CAs and a compound is available.

## Materials and methods

### Proteins

Recombinant human CA proteins (full-length or their catalytic domains) produced in bacterial or eukaryotic systems and chromatographically purified were used in the study. Protein preparation has been previously described [14].

### Compounds

Compounds were obtained from the following sources: 2-chlorobenzenesulfonamide, althiazide, bendroflumethiazide, benzthiazide, chlorothiazide, clopamide, cyclothiazide, dichlorofenamide, hydroflumethiazide, indapamide, metalozone, polythiazide, trichlormethiazide and xipamide were obtained from Sigma-Aldrich; chlorthalidone, clorexolone, fenquizone, and quinethazone were obtained from SantaCruz Biotechnology; hydrochlorothiazide and furosemide were obtained from AlfaAesar. Compound stock solutions (10–50) mM were prepared in dimethylsulfoxide (DMSO) and stored in a dark box at +4°C. Marvin was used for drawing, displaying and characterizing chemical structures, substructures and reactions; Marvin 19.4.0, 2019, ChemAxon (http://www.chemaxon.com).

### Fluorescence-based thermal shift assay

Fluorescence-based thermal shift assay (FTSA) was used to measure compound–protein binding affinity. This assay quantifies protein melting temperatures (*T*_*m*_) at various concentrations of the added compound. Protein unfolding is monitored by detecting the fluorescence of the solvatochromic dye 8-anilino-1-naphtalensulfonate (ANS), whose fluorescence increases upon protein unfolding [15–17]. Dependence of the protein *T*_*m*_ on ligand concentration was used to determine the dissociation constant *K*_*d*_. In this study, FTSA samples contained 5–20 μM protein, 50 μM ANS, 0–300 μM compound in 50 mM sodium phosphate or 25 mM Hepes buffer containing 50 mM NaCl, pH 7.5, and 2% (v/v) DMSO. Experiments were performed in Corbett Rotor-Gene 6000 instrument. Blue channel was used for excitation at 365 ± 20 nm and detection at 460 ± 15 nm of the ANS fluorescence. The heating rate of 1°/min was applied.

Affinities between 20 compounds and the 12 catalytically active isoforms of CA were determined by FTSA. Each *K*_*d*_ was estimated from a binding experiment consisting of 12 samples with different compound concentrations prepared by a 1.5x serial dilution (the last one consisted of a protein sample without the compound). The experiments were repeated at least twice, and averages are listed. The standard deviation analysis has been performed as previously described [18]. The data analysis was performed as previously described [19], and the equations used to fit the FTSA data and determine the *K*_*d*_ of compound interaction with each CA isoform are provided in the S1 File.

### Stopped-flow CO_2_ hydration activity assay

The inhibition of CA enzymatic activity by the compounds was measured by the stopped-flow CO_2_ hydration enzymatic activity assay (SFA) [20]. In this study, we used SFA to confirm that tested small molecules not only bind to CAs as shown by FTSA, but also inhibit their catalytic activity, and the observed affinity corresponds to *K*_*d*_s obtained by FTSA. The *IC*_50_ measured by SFA were compared to the affinities obtained by FTSA. The SFA was used to determine the *IC*_50_ values for **3** –CA II, **3** –CA IV, **4** –CA II, **4** –CA IV, **11** –CA IV, **12** –CA IV, **12** –CA XII, **16** –CA XII.

The CO_2_ spontaneously hydrates in an aqueous solution and the pH decreases. The CA enzyme significantly increases the hydration rate while an inhibitor decreases the rate in a dose-dependent manner until the spontaneous hydration rate is achieved. Experiments were performed using the Applied Photophysics SX.18MV-R stopped-flow spectrometer by following the absorbance change of Phenol-Red pH indicator at 557 nm. The saturated CO_2_ solution was prepared by bubbling the CO_2_ gas in MilliQ water at 25°C for 1 h. Experiments were performed at 25°C using 25 mM Hepes buffer containing 50 mM NaCl, pH 7.5, 0–100 μM inhibitor and 0.4% DMSO. The enzyme concentration in the assay was 20 nM for CA II, 25 nM for CA IV and 40 nM for CA XII. The rates of CO_2_ hydration were determined from the slopes of raw curves of initial absorbance change. To determine the fraction of inhibited enzyme, the spontaneous CO_2_ hydration rate was used as a zero, while the CA-catalyzed reaction rate in the absence of the compound—as a maximum value. Each experiment consisted of 17 compound concentrations, 16 prepared by serial ligand dilution and a last one without the compound. Many points ensured precision, and the experiments were not repeated because the values matched the results obtained by FTSA and ITC techniques. The dissociation constants *K*_*d*_ were determined using the Morrison equation as described by Copeland [21]. The equation and the fitting procedure is provided in the S1 File.

### Isothermal titration calorimetry

Isothermal titration calorimetry (ITC) is a standard technique to determine the affinity between a protein and a small-molecule compound. The heat absorbed or released upon the protein-ligand binding is measured in ITC. In this study, we used the technique to verify the *K*_*d*_ values obtained by FTSA and SFA. The *K*_*d*_ of compounds **2**, **3**, **4**, **8**, **9**, **12**, **13** and **16** binding to CA II were measured by ITC. The experiments were performed using the iTC200 instrument (Malvern Instruments Ltd, Malvern, UK). Protein concentration in the cell was 10–20 μM and the compound concentration in the titration syringe was 100–200 μM. The 50 mM sodium phosphate buffer with 50 mM NaCl, pH 7.5, was used both for the protein and compound solutions. Experiment consisted of 19–20 injections of 2 μL (with the first smaller injection of 0.4 μL) every 180 s at a reference power of 5.0 μcal/s (20.9 μJ/s) and the stirring speed of 750 rpm, temperature 25°C. All ITC experiments were repeated at least twice. The data analysis was performed using Microcal ITC module of the Origin 7.0 software using the single-site binding model.

## Results

**Fig 2:**
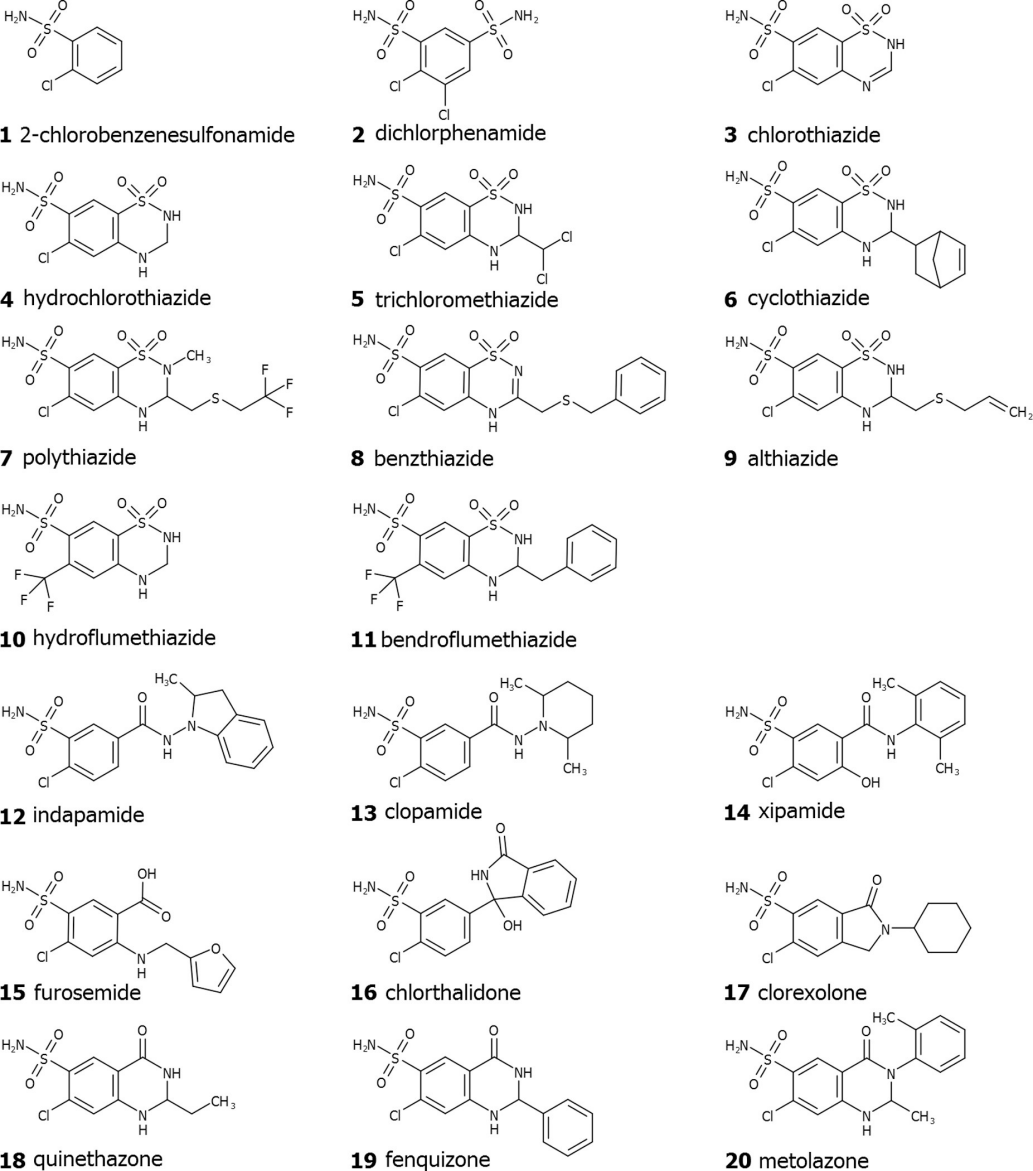
Chemical structures of compounds used in this study.

The chemical structures of compounds used in this study are shown in Fig 2. Compound **1**, 2-chloro-benzenesulfonamide, is a fragment of the remaining compounds, all of which have been used in the clinic. Dichlorphenamide **2** is an antiglaucoma drug; compounds **3**–**11** are thiazide diuretics and compounds **12**–**20** are structurally similar to thiazide drugs such as indapamide **12**, furosemide **15**, chlorthalidone **16**, metolazone **20** and others. The compounds in Fig 2 are arranged according to their structural similarities. Most compounds bear the chlorine substituent in the ortho- position relative to the primary sulfonamide group. The dichlorphenamide **2** bears two chloro- and two sulfonamide groups. The thiazides **3**–**11** bear the conjugated benzothiadiazine ring system and may have a trifluoromethyl group instead of the chlorine (**10**, **11**). The remaining drugs are structurally more diverse, but all contain the 2-chloro-benzenesulfonamide fragment.

We determined the affinities of the 19 pharmaceutical compounds (and compound **1**) with the complete set of 12 catalytically active human CA isoforms. The observed affinities of the 240 interactions are listed in Table 1. Some of the inhibition constants of those interactions have been previously determined and published (citations provided next to the previously determined values).

**Table 1:** Dissociation constants (*K*_*d*_, nM) of the compounds for 12 human CA isoforms.

No	CA I	CA II	CA III	CA IV	CA VA	CA VB	CA VI	CA VII	CA IX	CA XII	CA XIII	CA XIV
1.	270	560; 1125[10]	>200000	1200	320	470	2600	400	860	420	570	430
2.	480; 1200 [22]	3.2; 38 [22]	550; 680000[22]	6.3; 15000[22]	25; 630[22]	0.25; 21[22]	15; 79[22]	0.46; 26[22]	3.1; 50[22]	3.4; 50[22]	4.5; 23^m^[22]	0.92; 345[22]
3.	23000	370; 372 [23]	15000	310	830	48	620	70	130	180	39	250
4.	23000; 328[22]	2600; 7190 [23] 290[22]	200000; 790000[22]	1600; 427[22]	2000; 4225[22]	200; 603[22]	830; 3655[22]	870; 5010[22]	350; 367[22]	570; 355[22]	530; 3885^m^[22]	620; 4105[22]
5.	6200; 345 [24]	1700; 2600[23]; 91 [24]	>200000; 560000 [24]	1000; 449 [24]	3300; 763 [24]	1500; 134 [24]	1500; 2459 [24]	710; 7.9 [24]	260; 87 [24]	420; 312 [24]	940; 645^m^ [24]	590; 3450 [24]
6.	940	1200	270000	270	1100	140	430	500	150	170	430	360
7.	250	500	>200000	330	5000	280	580	100	100	1600	190	230
8.	4300	6.4; 16.7 [23]	4500	43	90	6.6	500	15	26	18	0.64	36
9.	1600	790	51000	330	1500	61	490	250	120	120	150	51
10.	50000; 2840 [22]	17000; 435 [22]	>200000; 870000 [22]	24000; 4780 [22]	40000; 10200 [22]	910; 429 [22]	29000; 8250 [22]	17000; 433 [22]	1600; 412 [22]	31000; 305 [22]	470; 15400^m^ [22]	3600; 360 [22]
11.	5800	1700	>200000	1600	32000	360	5300	440	210	480	910	130
12.	2800; 51900 [24]	130; 1420 [23] 2520 [24]	11000; 230000 [24]	47; 213 [24]	280; 890 [24]	13; 274 [24]	130; 1606 [24]	42; 0.23 [24]	140; 36 [24]	150; 10 [24]	41; 13^m^ [24]	88; 4950 [24]
13.	4900	370; 14600[23]	>200000	360	440	660	470	250	330	760	170	200
14.	56000	10000	>200000	1400	>200000	61000	36000	93000	15000	26000	680	15000
15.	830; 62 [24]	630; 3140 [23] 65 [24]	>200000; 3200000 [24]	920; 564 [24]	>200000; 499 [24]	120000; 322 [24]	49000; 245 [24]	620; 513 [24]	200; 420 [24]	120; 261 [24]	230; 550^m^ [24]	420; 52 [24]
16.	310; 348 [24]	35; 11.9 [23]; 138 [24]	17000; 11000 [24]	16; 196 [24]	4800; 917 [24]	470; 9 [24]	110; 1347[24]	43; 2.8 [24]	33; 23 [24]	12; 4.5 [24]	30; 15^m^ [24]	19; 4130[24]
17.	1600	76	>200000	250	42000	500	420	100	42	89	51	58
18.	51000; 35000 [22]	14000; 1260 [22]	180000	2100	7100	15000	3900	36000	2300	20000	1100	4700
19.	13000	15000	>200000	4400	11000	19000	5300	27000	2700	28000	930	5700
20.	130000; 54000 [22]	2800; 2000 [22]	>200000; 610000 [22]	91; 216 [22]	5800; 750 [22]	1400; 312 [22]	990; 1714 [22]	8300; 2.1 [22]	800; 320 [22]	15000; 5.4 [22]	180; 15^m^ [22]	1300; 5432 [22]

The *K*_d_ values in the top rows were determined in this study by FTSA and are the averages of at least two measurements. The second and the following rows in the cell (if provided) show the literature values of inhibition constants of CA enzymatic activity, *K*_i_ (nM), or the dissociation constants *K*_d_ (nM) (listed together with the citation).

^m^ murine protein.

To ensure the accuracy of the data, we applied three different assays to determine the dissociation constants: FTSA, ITC, and SFA. The FTSA, the most robust and universal for various proteins, was used to determine all 240 compound-protein pair interactions, while ITC and SFA were confirmatory methods for several protein-small molecule pairs. The FTSA can quantify interactions ranging from weak millimolar to tight picomolar, usually outside the limits of the other two techniques [9].

**Fig 3:**
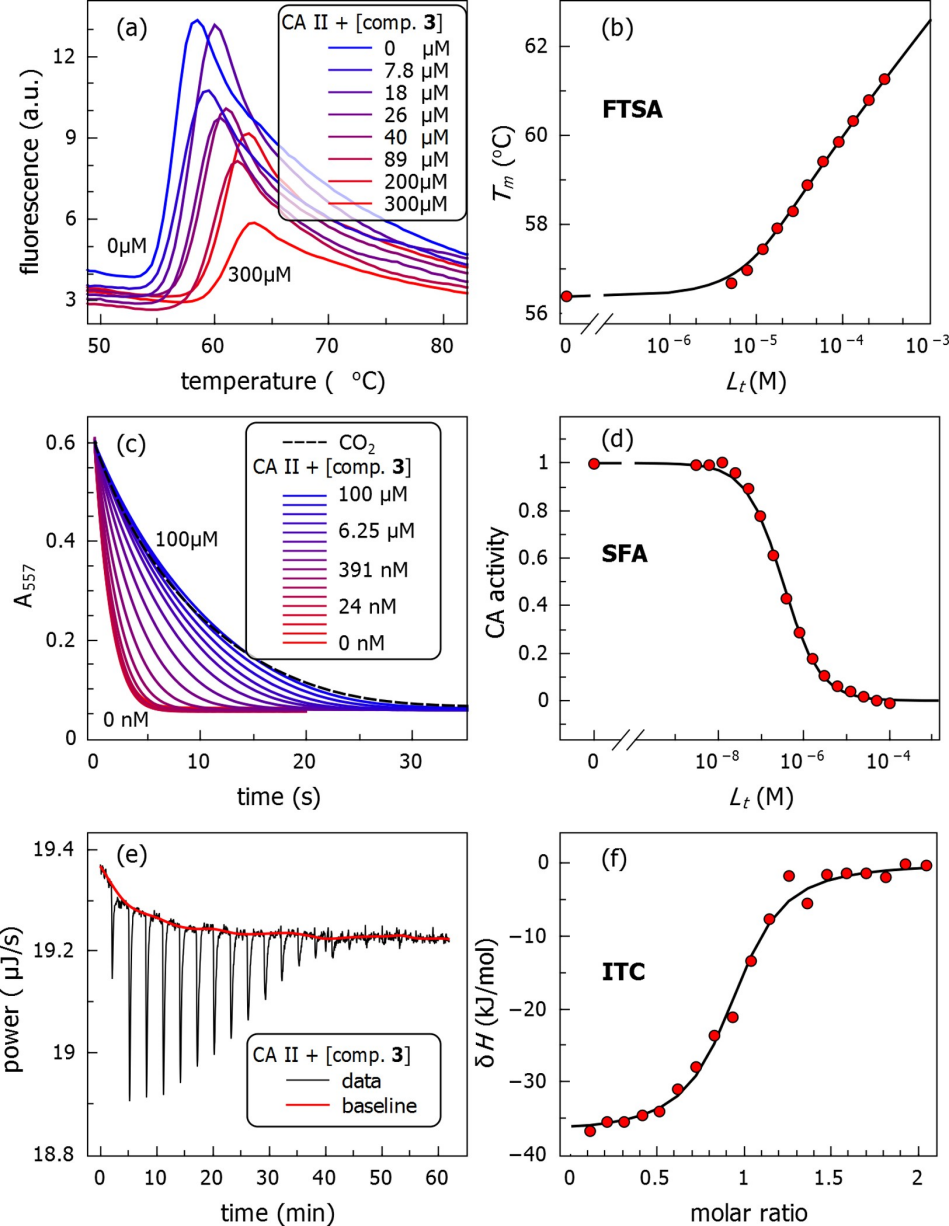
Binding isotherms of chlorothiazide 3 interaction with CA II determined by three assays. FTSA—panels (a) and (b)), SFA—panels (c) and (d), and ITC—panels (e) and (f). The left panels show the raw data, while the right panels—the dosing curves.

The FTSA curves of chlorothiazide **3** binding to CA II are shown in Fig 3A and 3B. The protein melting curves are shown on the left, while the dosing curves, obtained by arranging the *T*_m_ in the increasing compound concentration, are on the right. Usually, we obtained 12 data points per dosing curve, which is sufficient to ensure the appropriate accuracy of the experiment [25]. All compound dosing curves are provided in duplicates in the S1 File. The experiments have been performed independently with different protein preparations. The standard deviation of the dissociation constants is approximately plus-minus 2-fold of the value [18].

The major limitation of the FTSA is that the technique provides the *K*_*d*_, but does not show whether the compound is an inhibitor or just binds to some other site on the protein surface and thus may not inhibit its enzymatic activity. Thus, we performed the SFA that determines inhibition of enzymatic activity. The SFA raw absorbance curves of CA II enzymatic activity inhibition by chlorothiazide **3** are shown in Fig 3C. The decrease in absorbance was due to the decrease in pH, followed by spectrophotometric absorbance of the pH indicator Phenol Red. The compound dosing curve was assembled from the enzyme inhibition ratio as a function of compound concentration (Fig 3D). It was important to demonstrate that a compound completely inhibited the enzymatic activity at high concentrations and that the Hill or Morrison model could fit the dosing curve as previously discussed [9, 23, 26–29].

**Fig 4:**
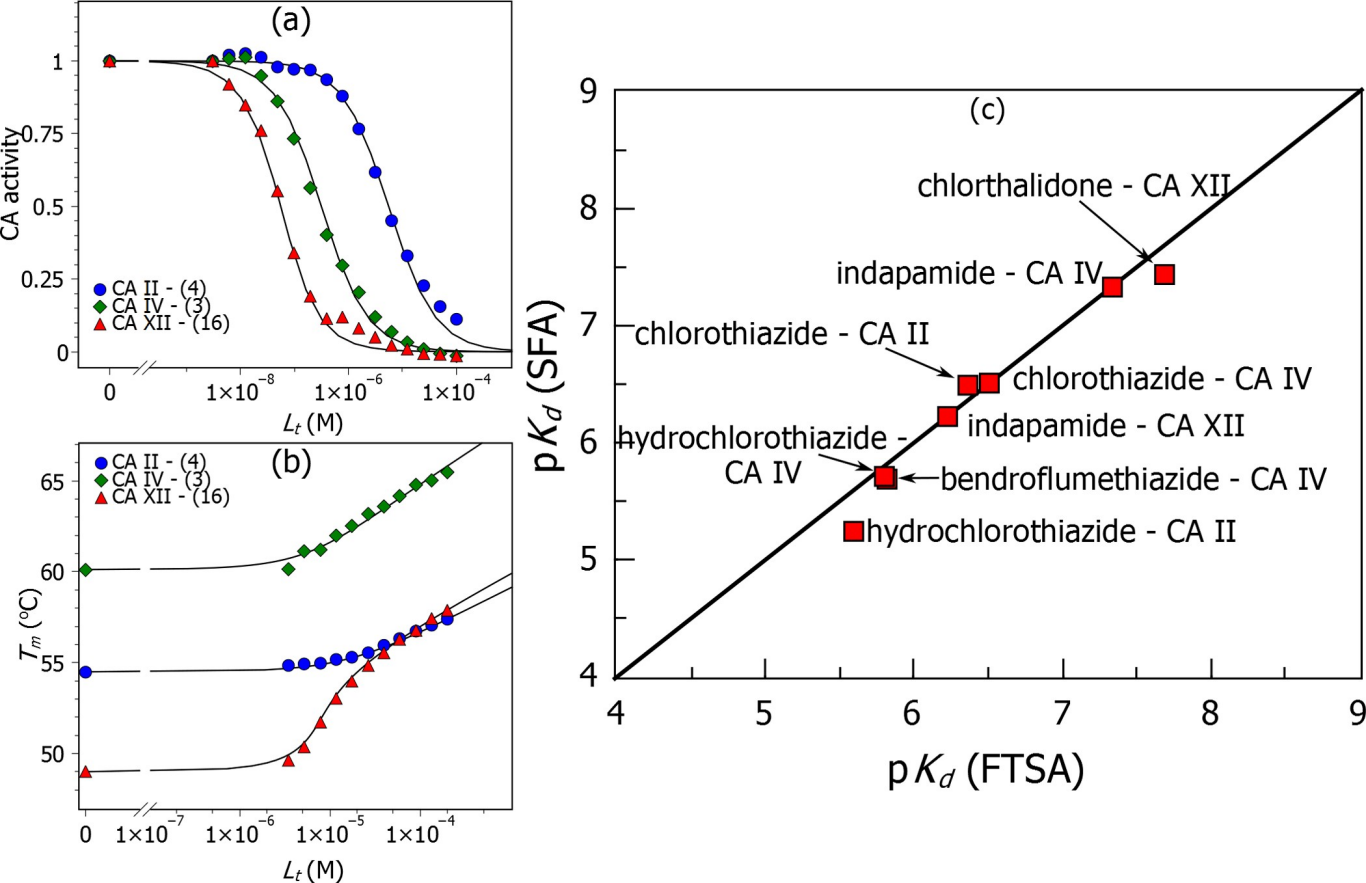
Comparison of compound 3, 4 and 16 binding affinities for CA II as determined by SFA (panel a) and FTSA (panel b). Panel (a) shows the SFA data: CA II binding to hydrochlorothiazide **4**, CA IV–chlorothiazide **3** and CA XII–chlorthalidone **16**. Panel (b) shows the FTSA data: CA II– **4**, CA IV– **3** and CA XII– **16**. Panel (c) compares the values and shows that both techniques yielded the same values (within the 2x error margin) for a wide range of affinities (micromolar to nanomolar compounds). The data points lie close to the solid diagonal line showing would-be-perfect agreement.

The inhibition constants obtained by the SFA method for the tested compound–CA isoform pairs matched the values obtained by the FTSA method within the error margin. The correlation between the two assays is shown in Fig 4. Despite the highly different principles of both assays, both techniques provided similar values under the condition that the affinities were in the micromolar to nanomolar range. For example, chlorothiazide **3** affinity to CA IV by FTSA was 310 nM, while by SFA, it was 300 nM. More tightly-binding chlorthalidone **16** to CA XII exhibited the affinity of 35 nM by SFA and 21 nM by FTSA. Similarly, more weakly-binding hydrochlorothiazide **4** exhibited the affinity for CA II of 5500 nM by SFA and 2600 nM by FTSA. SFA results confirmed that the compounds inhibit CAs by binding to Zn(II).

To triple-confirm the accuracy of the measurements, a subset of protein-ligand pairs was analyzed by the ITC. This technique consumes a relatively large amount of protein compared to FTSA and SFA. Therefore, not all interactions were determined using ITC. Typical ITC data are shown in Fig 3E, 3(f). The raw power-compensation curve is shown on the left and an integrated curve—on the right. The steepness of the integrated curve corresponds to the affinity of the interaction. The *K*_*d*_ determined by all three assays shown in Fig 3 yielded essentially the same values, namely, 370 nM by FTSA, 310 nM by SFA and 250 nM by ITC.

**Fig 5:**
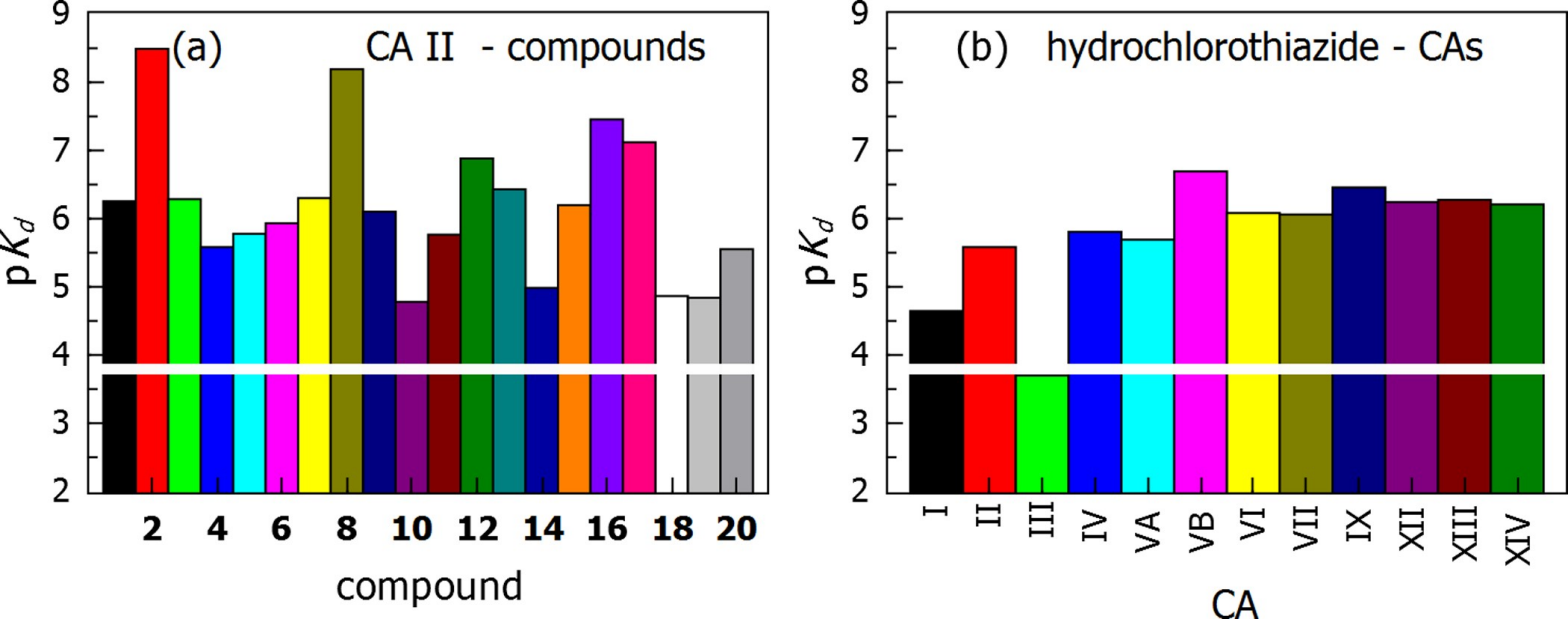
Selectivities of compound binding to CA isoforms are shown as affinity bars expressed in p*K*_d_. Panel (a) shows the binding of compounds to CA II. Affinities differed up to three orders of magnitude for various compounds. Panel (b) shows hydrochlorothiazide **4** binding to the twelve catalytically active human CA isoforms. Affinities varied by up to approximately one order of magnitude among CA isoforms. Most isoforms exhibited affinity of single-digit micromolar order of magnitude. The white horizontal line shows the lower limit of binding detection of 200 μM by FTSA.

Many compounds exhibited limited selectivity for any CA isoform (Table 1). For example, hydrochlorothiazide **4** bound most CA isoforms with the *K*_d_ of approximately 1 μM (Fig 5). However, some compounds of relatively similar chemical structures exhibited significantly different affinities for CA isoforms. For example, CA II bound the compounds with *K*_d_ ranging from 10 μM to 3 nM. Compounds **10**, **18,** and **19** were weak binders, while **2** and **8** –potent ones. The CA I and CA III exhibited weaker affinity than other isoforms, a characteristic feature of these isoforms.

**Fig 6:**
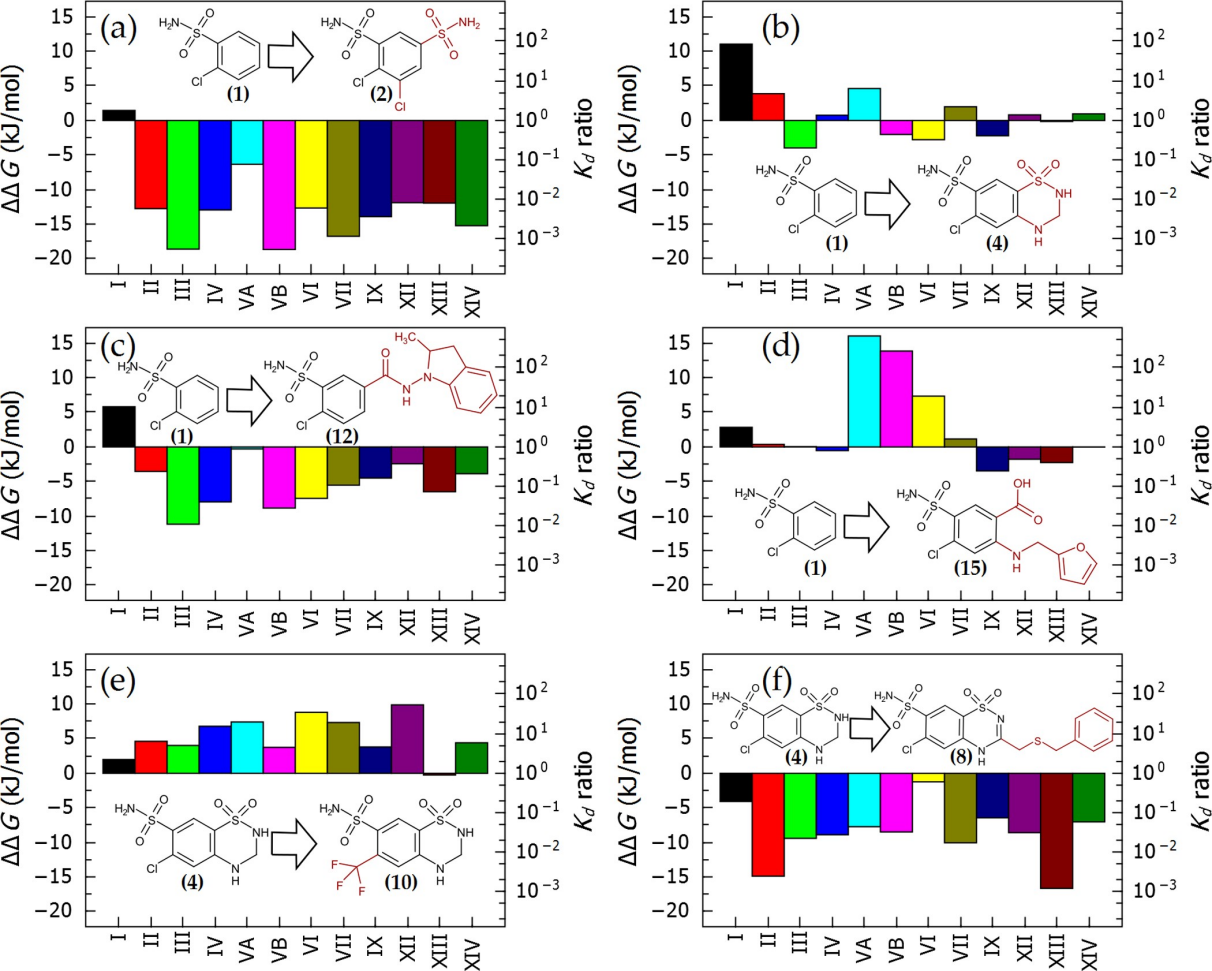
A comparison of two structurally related compound affinities. The addition of chemical groups that are shown in red may enhance or diminish compound affinity for a particular CA isoform. This effect is shown on the left axis as the difference in the standard Gibbs energies of binding of both compounds corresponding to the ratio of the dissociation constants of both compounds on the right axis. The addition of some groups led to a nearly universal increase in binding affinity (a, f), or affected only a narrow subset of CA isoforms (d).

The affinities of structurally similar compounds were compared, and the differences were assigned to the differing functional groups (Fig 6). For example, the addition of the second chlorine and a second sulfonamide group in **2** relatively to **1** increased the affinity by up to 1000-fold for CA III, CA VB, and CA VII (Fig 6, panel (a)). The affinity changes in this figure are shown on two scales: the standard Gibbs energy change upon binding is shown on the left, while the *K*_d_ ratio–on the right side of the graph. Isoform CA I was exceptional, and there was no gain in affinity upon changing the compound structure from **1** to **2**.

Fig 6B shows little or no gain in affinity when **1** was changed to hydrochlorothiazide **4**. There was even some 100-fold loss in affinity for CA I. The structural moiety shown in red of indapamide **12** contributed favorably, and thus its affinity for most isoforms increased, except for CA I, where there was a nearly 10-fold loss, and for CA VA, where there was no change observed. Furosemide **15** bound CA VA, CA VB, and CA VI significantly weaker than **1**, while the structural differences had little impact on other CA isoform binding.

The change of chlorine in thiazides to trifluoromethyl (Fig 6E) had a relatively minor effect but consistently diminished the affinity for nearly all CA isoforms. Thus, the trifluoromethyl group is likely sterically hindering the interaction compared with the chlorine atom or could form weaker interaction with the protein. On the other hand, the addition of the thio-phenyl group in compound **4** to form **8** increased the affinity up to 1000-fold, especially for CA II and CA XIII. Such comparisons identify the contribution of chemical groups to the compound affinity.

The studied compounds bound to every CA isoform (Table 1). The binding stoichiometry in ITC was approximately equal to 1:1, indicating that all compounds bound to the active site Zn(II) as confirmed for a subset of interactions by SFA. The isoform that usually exhibited the weakest binding was CA III. It contains Phe198 residue in the active site cavity that prevents binding of bulkier sulfonamide compounds that usually bind stronger to other CA isoforms [30, 31].

There were several cases where an affinity for CA exceeded 1 nM, namely, the dichlorophenamide **2** bound to CA VB, CA VII and CA XIV with 0.25 nM, 0.46 nM and 0.92 nM affinities, respectively. The benzthiazide **8** bound to CA XIII with 0.64 nM affinity. No other compounds reached sub-nanomolar affinities–most other compounds bound in the nanomolar to micromolar range. Thus, these pharmaceutically used compounds should be evaluated in the context that they bind many human CA isoforms in vitro and may strongly affect their activity and function *in vivo*, in addition to the primarily intended target isoforms.

## Discussion

It has been previously known that sulfonamide-bearing compounds bind and inhibit CAs, but only for several CA isoforms and not all compounds studied here. We determined the binding affinities of the pharmaceutically used compounds bearing 2-chlorobenzenesulfonamide moiety to all twelve human carbonic anhydrase isoforms by FTSA. In addition, the accuracy was checked by two additional independent techniques, SFA and ITC. Previously we have demonstrated the good correlation of affinity measured by FTSA, ITC, SFA and surface plasmon resonance [9]. Here we also observed good agreement between binding affinities measured by FTSA and SFA or ITC assays. The binding affinities of some sulfonamide diuretics to CAs have been previously determined [13, 22, 24]. Comparison between values obtained by FTSA with the previously determined values was partially in agreement, with some larger differences, especially for dichlorphenamide. There may be numerous reasons for disagreement. First, the recombinant proteins may have been somewhat different. Second, previous measurements have been performed by an enzymatic activity inhibition assay while we performed mostly FTSA. Third, the number of data points in the inhibition or FTSA curves was quite different. In our opinion, the number of data points should be at least 12 as was in this study. Fourth, the affinities depend strongly on exact experimental conditions that may have been different.

### Thiazides

Thiazides are one of the largest groups of drugs containing an unsubstituted sulfonamide moiety. These drugs, mainly used as diuretics, contain the benzothiadiazine group in their structure. This diuretic class with the first-in-class drug chlorothiazide was introduced in 1957 upon chemical modification of CA inhibitors [32, 33]. Currently, thiazide diuretics are often used as first-line treatment for hypertension, despite that their exact mechanism of action is still unclear–it depends on the time course of the drug use: the acute action relies on the inhibition of Na^+^/Cl^-^ cotransporter, while the mechanism of chronic action still has to be clarified [34]. One of their many possible targets, previously summarized [1], are CAs [35]. Carbonic anhydrase inhibition in proximal tubule increases HCO_3_^-^ and PO_4_^3-^ excretion. Even though CAs are not the primary targets of thiazide class compounds in the human body, the interaction of several members of this compound class is well documented in scientific publications using *in vivo* models and *in vitro* measurements [3, 24].

Here we analyzed nine thiazide diuretics: chlorothiazide **3**, hydrochlorothiazide **4**, trichloromethiazide **5**, cyclothiazide **6**, polythiazide **7**, benzthiazide **8**, althiazide **9**, hydroflumethiazide **10**, and bendroflumethiazide **11**. The fact that human CAs bind with **3**, **6**, **7**, **9**, and **11** has been demonstrated for the first time. The affinities of compound **4** usually matched the previously determined values within 10-fold, but several other affinities were weaker. Benzthiazide **8** showed stronger binding to all CA isoforms compared to other thiazide class compounds. Its high affinity to CA has been previously reported: it exhibited lower *K*_i_ (16 nM) compared to hydrochlorothiazide **4** (2350 nM), chlorothiazide **3** (460 nM) and furosemide **15** (80 nM), but without identification which CA isoform was used for the study [36].

The studied thiazide compounds had the highest affinity to CA XIII, CA IX, CA VB, CA XIV, and CA VII, while other isoforms, including CA I and CA II, were less affected. The largest observed changes in affinity (up to approx. 1000 fold for CA XIII and CA II) within the thiazide group were between compounds **8** and **4**, which differ by a single benzylsulfanylmethyl moiety. The reasons for such change cannot be unambiguously explained without structural information, but additional hydrophobic contacts close to the entrance of the CA active site are expected. The increase in affinity observed with an increase of hydrophobicity of compound substitutes distant from the sulfonamide group has been previously reported [37, 38]. Even minor structural modifications can result in a significant difference of affinity as in compounds **3** and **4**. The most likely explanation for observed stronger chlorothiazide **3** binding is the lower value of its sulfonamide group p*K*_*a*_ [39].

### Non-thiazide diuretics

Indapamide **12** and structurally related clopamide **13**, and xipamide **14** are used to treat edema and hypertension. These chloro-sulfonamides differ only in the ring system on the meta-position substitute. However, they exhibit different affinities: **14** was a very weak binder for all CA isoforms except CA XIII (680 nM) and CA IV (1.4 μM), while **13** bound most isoforms within 170–660 nM range, with lower affinity for CA I and CA III. The **12** had the highest affinity within this series: 13 nM for CA VB and within 41–280 nM for most other CAs except CA I and CA III.

Furosemide **15** is the first high ceiling (loop) diuretic. Its action is based on the inhibition of Na^+^K^+^2Cl^-^ co-transport. Furosemide is classified as a weak CA inhibitor, and its diuretic action is not linked to CA inhibition [40]. However, it exhibited moderate affinity (120–830 nM) for many human CA isoforms–only CA III, CA VA, CAVB, and CA VI had somewhat lower affinity for **15**.

Chlorthalidone **16** is used as an antihypertensive agent. Even though it is often classified as a "thiazide-like" drug according to its action, it has low structural similarity to thiazides [41]. Its antihypertensive action involves inhibition of human CAs [42]. In our study, it showed high affinity in the range of 12 nM to 43 nM for CA XII, CA IV, CA XIV, CA XIII, CA II, and CA VII.

Clorexolone **17** is structurally related both to thiazides and chlorthalidone **16**. *In vivo* inhibition of CA has little contribution to the overall diuretic activity [43], while *in vitro* binding shows the average binding affinity within 42–500 nM for most CA isoforms.

Quinazoline sulfonamides, yet another class of diuretic agents, were discovered approximately 60 years ago [44]. Their structural basis is the six-membered heterocyclic ring system quinazoline. Derivatives of quinazoline–metolazone **20**, fenquizone **19** and quinethazone **18** –are mainly used to control edema and hypertension. In general, this class of drugs showed low affinity to human CAs, with some selectivity for CA XIII (all three compounds) and CA IV (**20** only).

The affinities of analyzed compounds to human CAs were comparable or even more potent than to their primary targets. For instance, *IC*_50_ values in the presence of 2 mM Cl^-^ for TSC (thiazide sensitive co-transporter) were 350 nM for metolazone **20**; 500 nM for bendroflumethiazide **11**; 500 nM for trichloromethiazide **5**; 7000 nM for hydrochlorothiazide **4**; 20 nM for polythiazide **7** [45].

Many indicative conclusions could be made based on the *in vitro* enzyme inhibition and binding studies. However, the results should not be blindly used as a scoring method for drug effectiveness evaluation since many characteristics are defined by the processes observed only *in vivo*, such as permeability, distribution, and other pharmacokinetic properties [46]. Still, a comprehensive analysis of these compounds binding to proteins *in vitro* could predict or propose new applications and ideas about the repurposing of the existing drugs. Such measurements could add additional information for the design of new small molecule compounds targeting human CAs.

## Conclusions

A group of clinically used diuretic drugs that belong to thiazide and structurally related classes and bear a primary sulfonamide group, in addition to their primary pharmacological targets, quite strongly bind and inhibit the family of twelve catalytically active human CAs. Relatively minor structural changes of the compounds sometimes yielded significant changes in compound affinities and selectivities for a particular CA isoform.

## Supporting information

S1 File All raw data of compound affinity determinations by FTSA, SFA, and ITC techniques and the equations used to fit FTSA and SFA data are presented in S1 File. (PDF)
