# Supplementary material for: Thiazide and other Cl-benzenesulfonamide-bearing clinical drug affinities for human carbonic anhydrases
Source: PLoS One. 2021 Jun 24;16(6):e0253608. doi: 10.1371/journal.pone.0253608 (PMC8224972; doi:10.1371/journal.pone.0253608)
Supplement: S1 File — (PDF) [file pone.0253608.s001.pdf]

S1 File:

Thiazide and other Cl-benzenesulfonamide-bearing clinical drug affinities for human carbonic anhydrases

Lina Baranauskiene[1], Lina Škiudaitė[1,2], Vilma Michailovienė[1], Vytautas Petrauskas[1], Daumantas Matulis[1,*]

1 Department of Biothermodynamics and Drug Design, Institute of Biotechnology, Life Sciences Center, Vilnius University. Address: Saulėtekio 7, Vilnius, Lithuania
2 Pharmacy Center, Institute of Biomedical Science, Faculty of Medicine, Vilnius University. Address: M. K. Čiurlionio 21/27, Vilnius, Lithuania

*Correspondence:
Prof. Daumantas Matulis, Department of Biothermodynamics and Drug Design, Institute of Biotechnology, Life Sciences Center, Vilnius University. Address: Saulėtekio 7, Vilnius, LT-10257, Lithuania
Email: daumantas.matulis@bti.vu.lt

Contents:

Compounds used in this study 2

1. 2-chlorobenzenesulfonamide 3
2. Dichorphenamide 7
3. Chlorothiazide 12
4. Hydrochlorothiazide 18
5. Trichloromethiazide 24
6. Cyclothiazide 28
7. Polythiazide 32
8. Benzthiazide 36
9. Athiazide 41
10. Hydroflumethiazide 46
11. Bendroflumethiazide 50
12. Indapamide 55
13. Clopamide 61
14. Xipamide 66
15. Furosemide 70
16. Chlorthalidone 74
17. Clorexolone 80
18. Quinethazone 84
19. Fenquizone 88
20. Metolazone 92

Equations used to fit the fluorescence-based thermal shift assay (FTSA) data 96

Equation used to fit stopped-flow enzymatic inhibition assay (SFA) data 98

References 98

Compounds used in this study

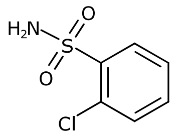

1 2-chlorobenzenesulfonamide

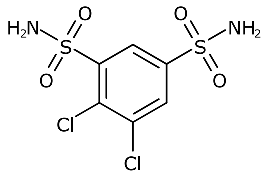

2 dichlorphenamide

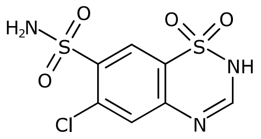

3 chlorothiazide

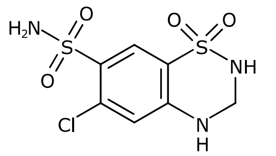

4 hydrochlorothiazide

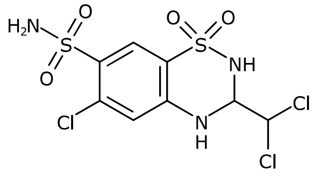

5 trichloromethiazide

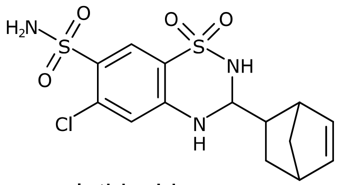

6 cyclothiazide

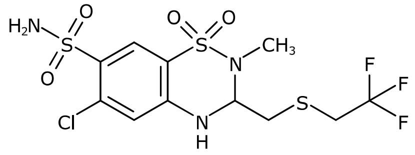

7 polythiazide

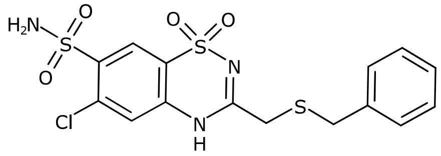

8 benzthiazide

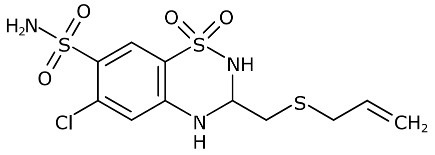

9 althiazide

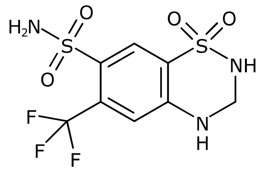

10 hydroflumethiazide

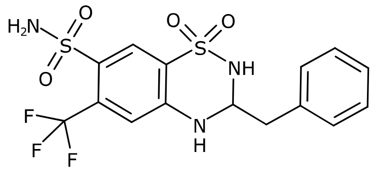

11 bendroflumethiazide

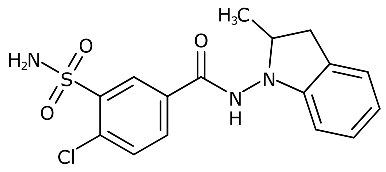

12 indapamide

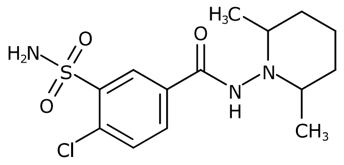

13 clopamide

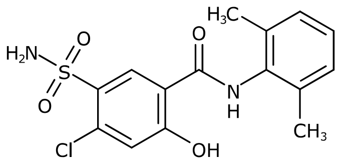

14 xipamide

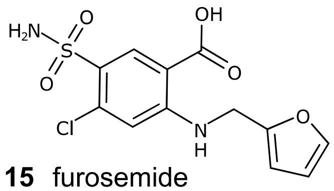

15 furosemide

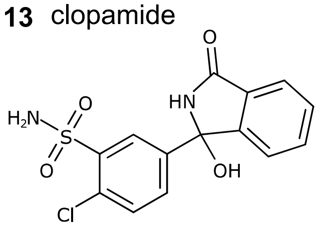

16 chlorthalidone

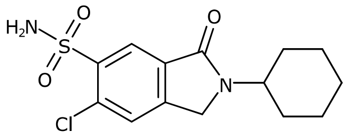

17 clorexolone

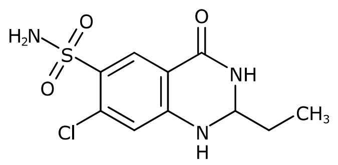

18 quinethazone

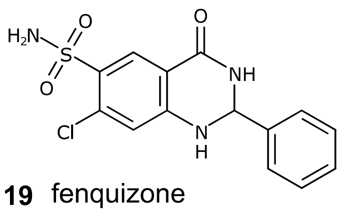

19 fenquizone

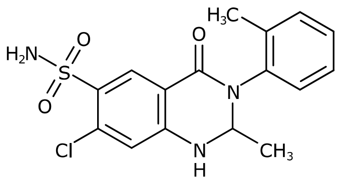

20 metolazone

1. 2-chlorobenzenesulfonamide

Fluorecence-based thermal shift assay

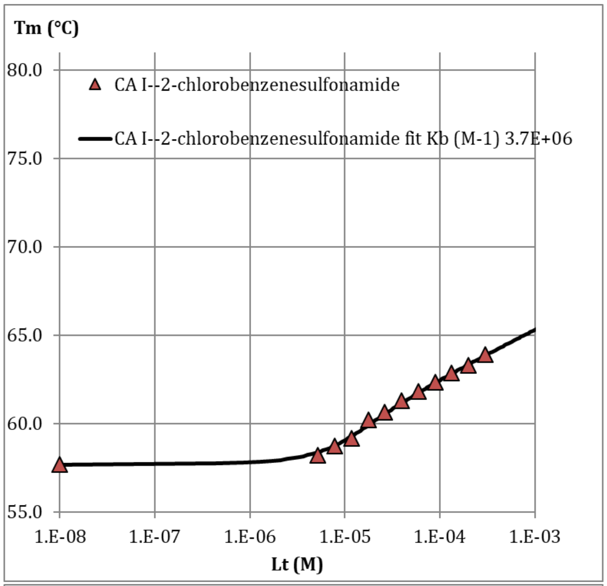

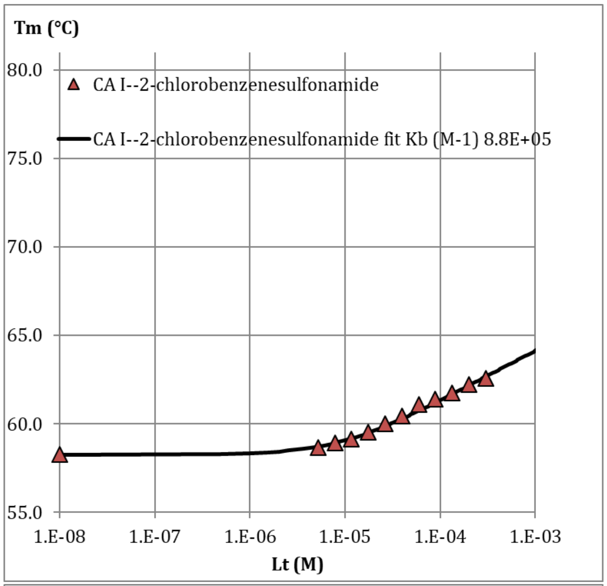

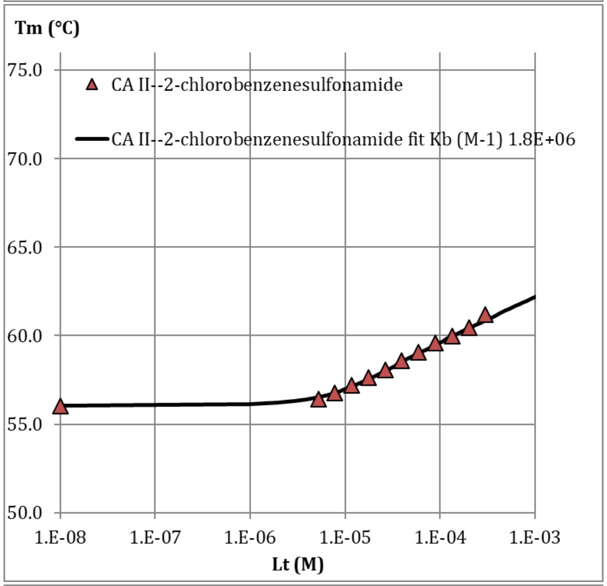

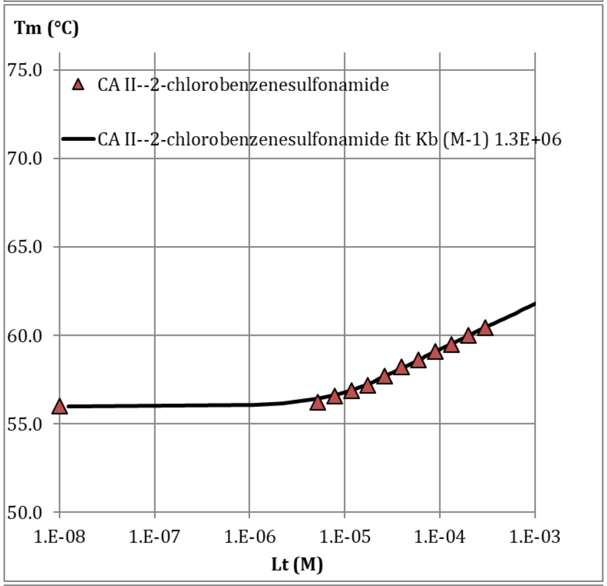

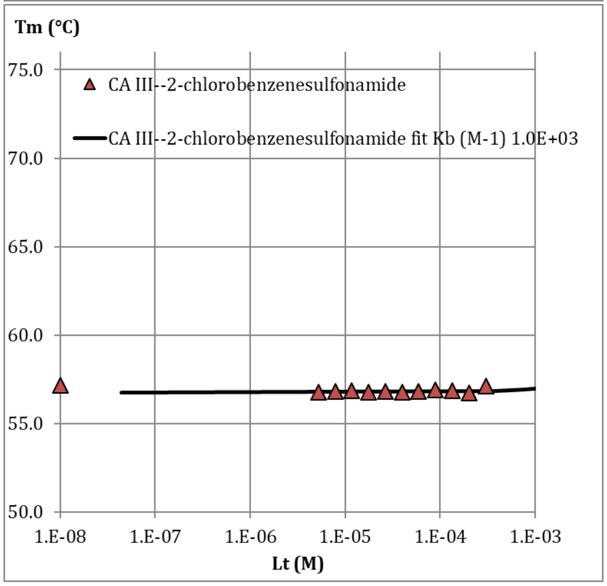

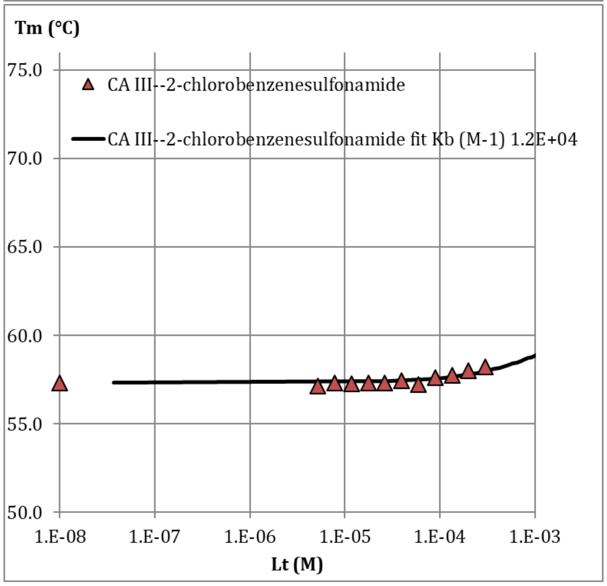

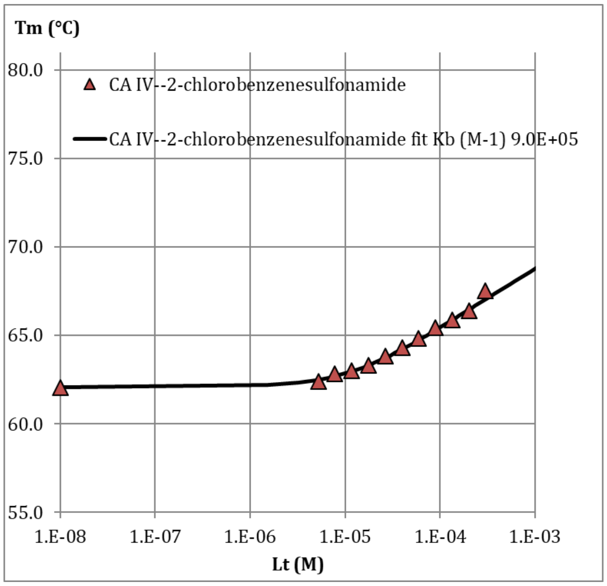
Tm (°C)
80.0
75.0
70.0
65.0
60.0
55.0
CA IV--2-chlorobenzenesulfonamide
CA IV--2-chlorobenzenesulfonamide fit Kb (M-1) 9.0E+05
1.E-08
1.E-07
1.E-06
1.E-05
1.E-04
1.E-03
Lt (M)

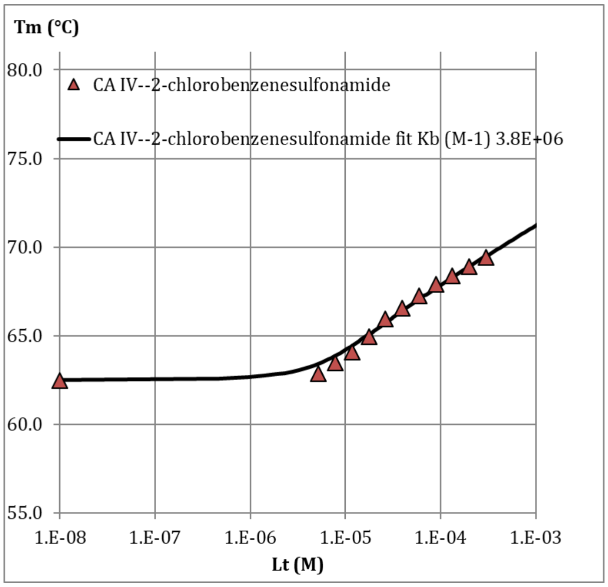
Tm (°C)
80.0
75.0
70.0
65.0
60.0
55.0
CA IV--2-chlorobenzenesulfonamide
CA IV--2-chlorobenzenesulfonamide fit Kb (M-1) 3.8E+06
1.E-08
1.E-07
1.E-06
1.E-05
1.E-04
1.E-03
Lt (M)

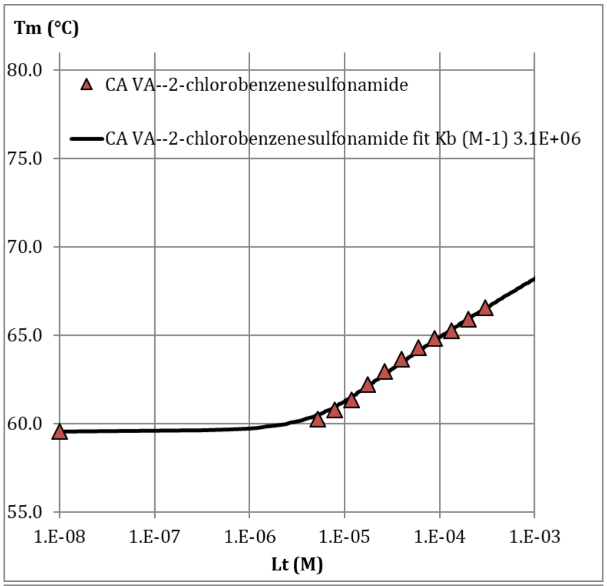
Tm (°C)
80.0
75.0
70.0
65.0
60.0
55.0
CA VA--2-chlorobenzenesulfonamide
CA VA--2-chlorobenzenesulfonamide fit Kb (M-1) 3.1E+06
1.E-08
1.E-07
1.E-06
1.E-05
1.E-04
1.E-03
Lt (M)

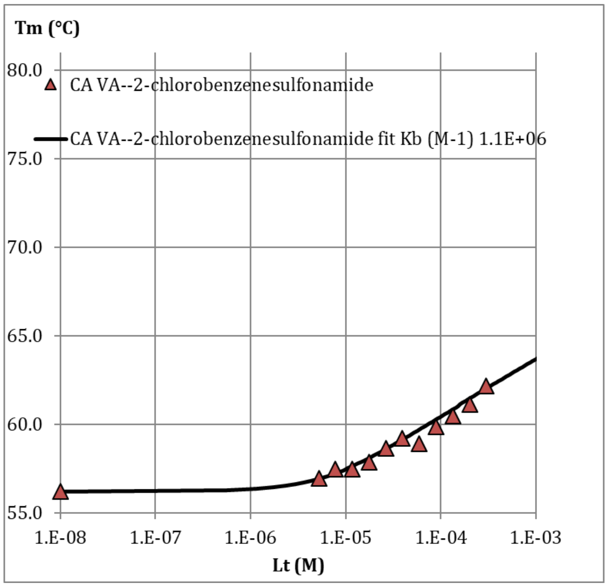
Tm (°C)
80.0
75.0
70.0
65.0
60.0
55.0
CA VA--2-chlorobenzenesulfonamide
CA VA--2-chlorobenzenesulfonamide fit Kb (M-1) 1.1E+06
1.E-08
1.E-07
1.E-06
1.E-05
1.E-04
1.E-03
Lt (M)

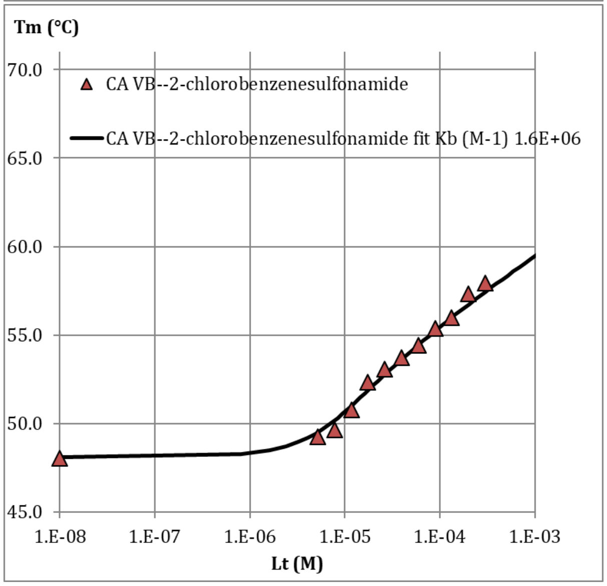
Tm (°C)
70.0
65.0
60.0
55.0
50.0
45.0
CA VB--2-chlorobenzenesulfonamide
CA VB--2-chlorobenzenesulfonamide fit Kb (M-1) 1.6E+06
1.E-08
1.E-07
1.E-06
1.E-05
1.E-04
1.E-03
Lt (M)

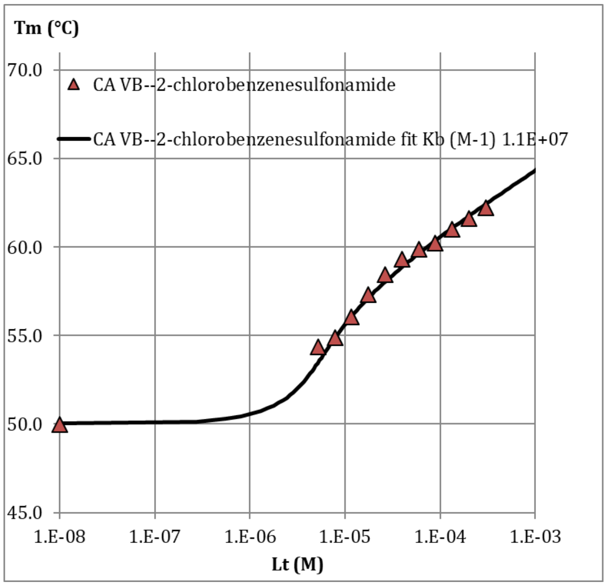
Tm (°C)
70.0
65.0
60.0
55.0
50.0
45.0
CA VB--2-chlorobenzenesulfonamide
CA VB--2-chlorobenzenesulfonamide fit Kb (M-1) 1.1E+07
1.E-08
1.E-07
1.E-06
1.E-05
1.E-04
1.E-03
Lt (M)

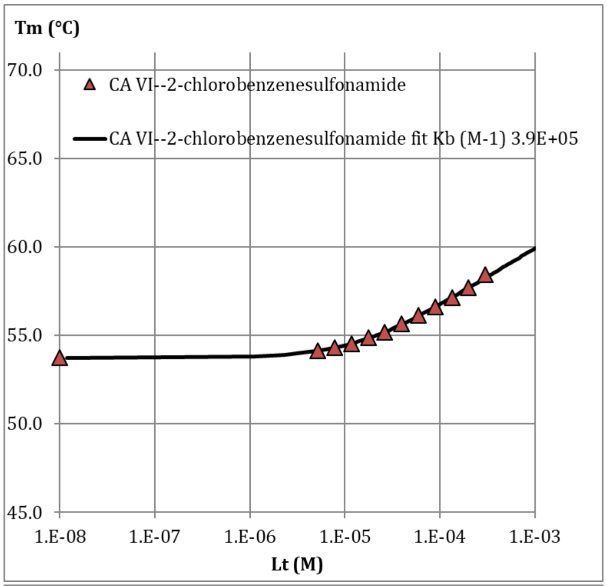
Tm (°C)
70.0
65.0
60.0
55.0
50.0
45.0
CA VI--2-chlorobenzenesulfonamide
CA VI--2-chlorobenzenesulfonamide fit Kb (M-1) 3.9E+05
1.E-08
1.E-07
1.E-06
1.E-05
1.E-04
1.E-03
Lt (M)

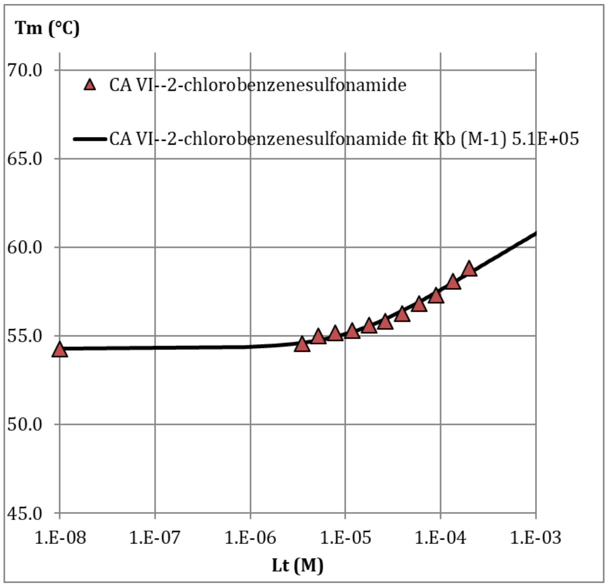
Tm (°C)
70.0
65.0
60.0
55.0
50.0
45.0
CA VI--2-chlorobenzenesulfonamide
CA VI--2-chlorobenzenesulfonamide fit Kb (M-1) 5.1E+05
1.E-08
1.E-07
1.E-06
1.E-05
1.E-04
1.E-03
Lt (M)

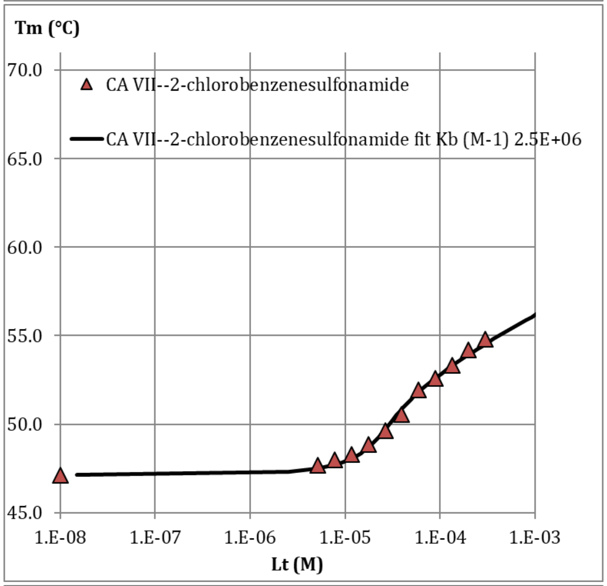
Tm (°C)
70.0
65.0
60.0
55.0
50.0
45.0
CA VII--2-chlorobenzenesulfonamide
CA VII--2-chlorobenzenesulfonamide fit Kb (M-1) 2.5E+06
1.E-08
1.E-07
1.E-06
1.E-05
1.E-04
1.E-03
Lt (M)

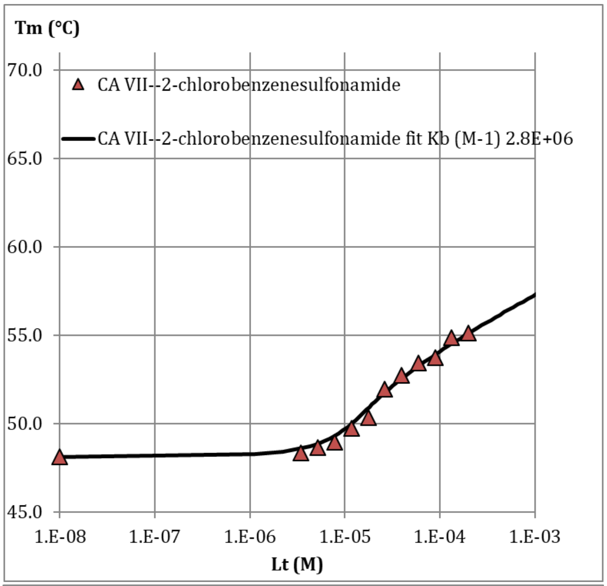
Tm (°C)
70.0
65.0
60.0
55.0
50.0
45.0
CA VII--2-chlorobenzenesulfonamide
CA VII--2-chlorobenzenesulfonamide fit Kb (M-1) 2.8E+06
1.E-08
1.E-07
1.E-06
1.E-05
1.E-04
1.E-03
Lt (M)

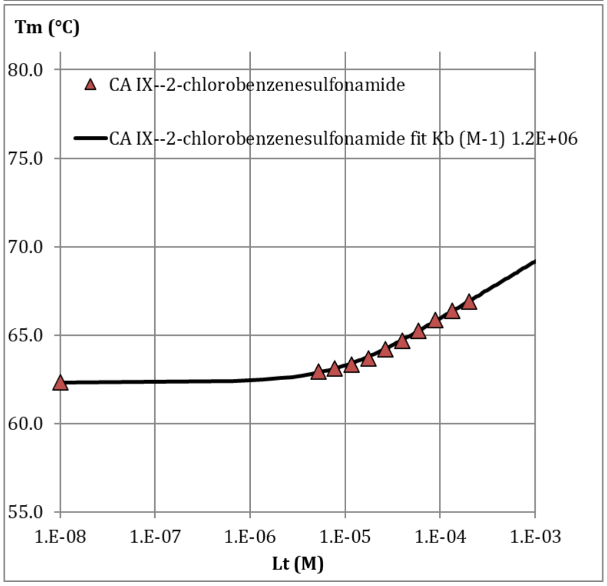
Tm (°C)
80.0
75.0
70.0
65.0
60.0
55.0
CA IX--2-chlorobenzenesulfonamide
CA IX--2-chlorobenzenesulfonamide fit Kb (M-1) 1.2E+06
1.E-08
1.E-07
1.E-06
1.E-05
1.E-04
1.E-03
Lt (M)

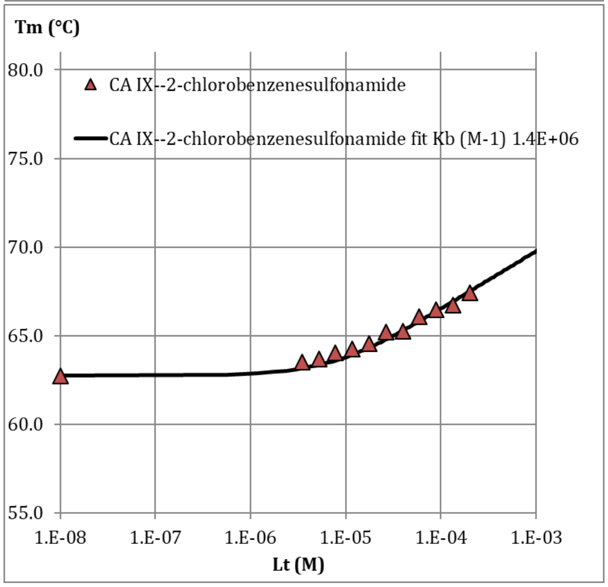
Tm (°C)
80.0
75.0
70.0
65.0
60.0
55.0
CA IX--2-chlorobenzenesulfonamide
CA IX--2-chlorobenzenesulfonamide fit Kb (M-1) 1.4E+06
1.E-08
1.E-07
1.E-06
1.E-05
1.E-04
1.E-03
Lt (M)

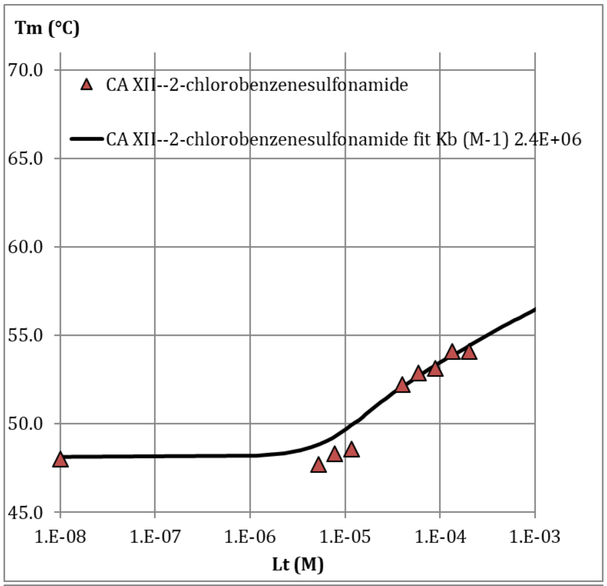
Tm (°C)
CA XII--2-chlorobenzenesulfonamide
CA XII--2-chlorobenzenesulfonamide fit Kb (M-1) 2.4E+06
Lt (M)

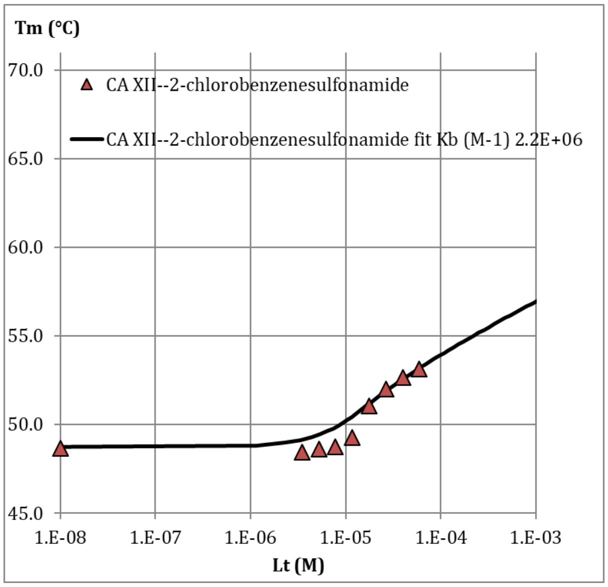
Tm (°C)
CA XII--2-chlorobenzenesulfonamide
CA XII--2-chlorobenzenesulfonamide fit Kb (M-1) 2.2E+06
Lt (M)

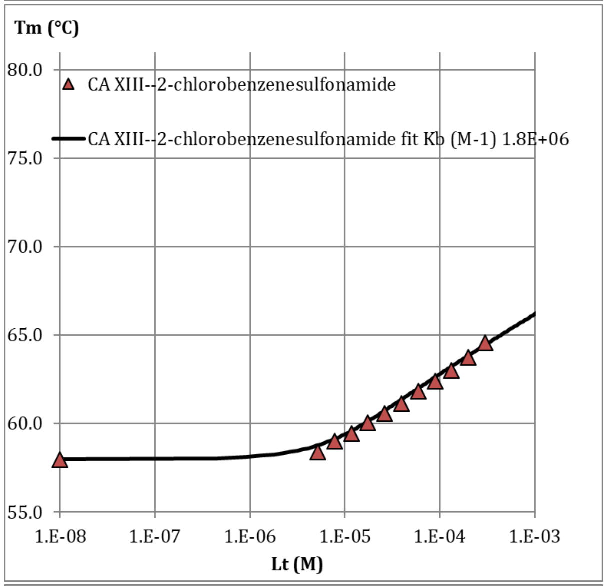
Tm (°C)
CA XIII--2-chlorobenzenesulfonamide
CA XIII--2-chlorobenzenesulfonamide fit Kb (M-1) 1.8E+06
Lt (M)

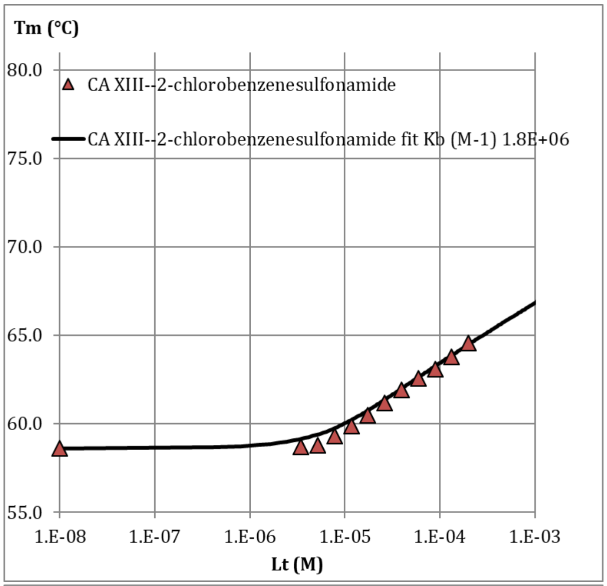
Tm (°C)
CA XIII--2-chlorobenzenesulfonamide
CA XIII--2-chlorobenzenesulfonamide fit Kb (M-1) 1.8E+06
Lt (M)

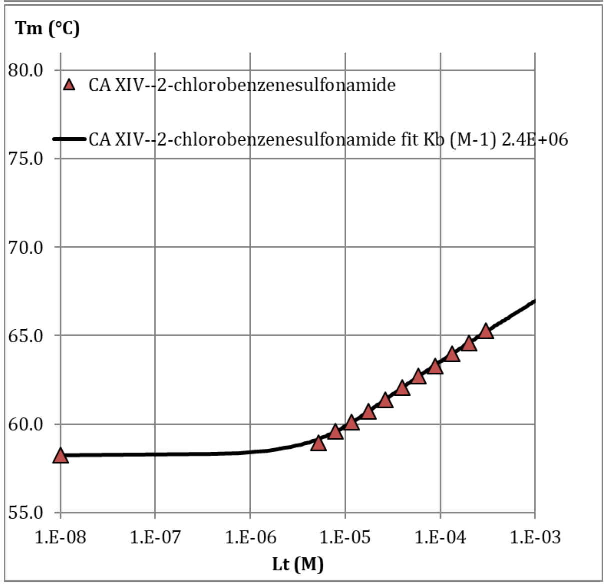
Tm (°C)
CA XIV--2-chlorobenzenesulfonamide
CA XIV--2-chlorobenzenesulfonamide fit Kb (M-1) 2.4E+06
Lt (M)

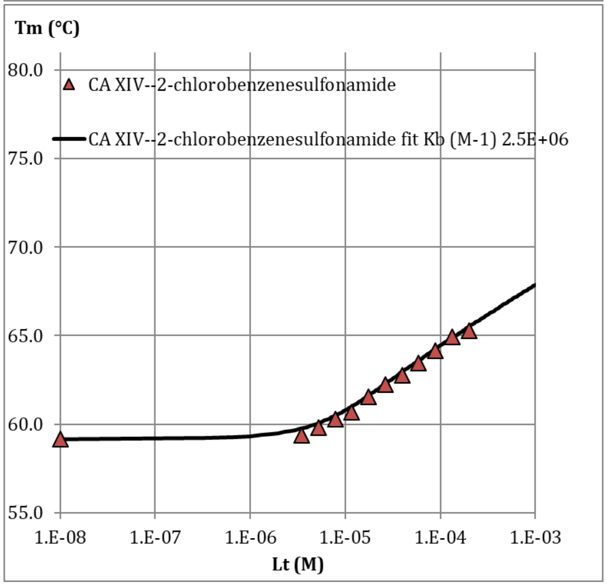
Tm (°C)
CA XIV--2-chlorobenzenesulfonamide
CA XIV--2-chlorobenzenesulfonamide fit Kb (M-1) 2.5E+06
Lt (M)

2. Dichorphenamide

Fluorecence-based thermal shift assay

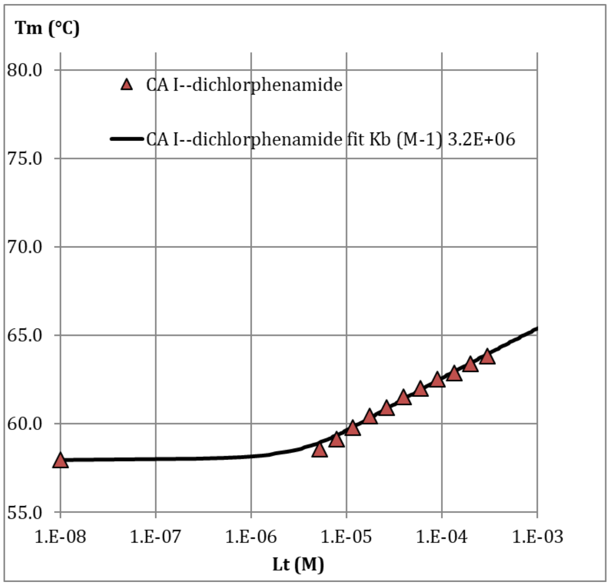

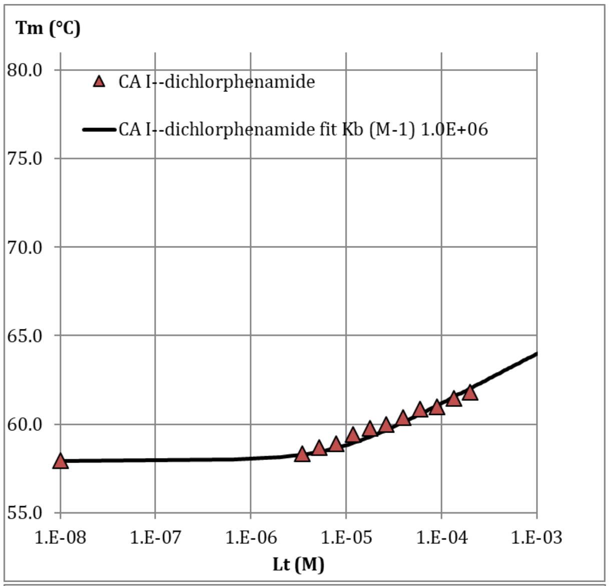

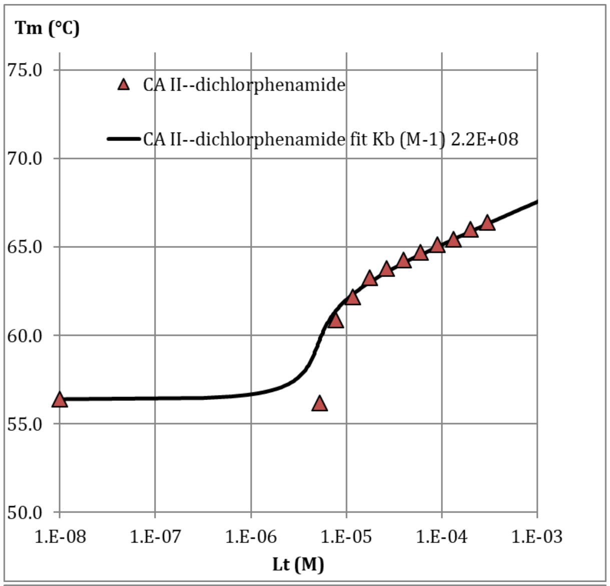

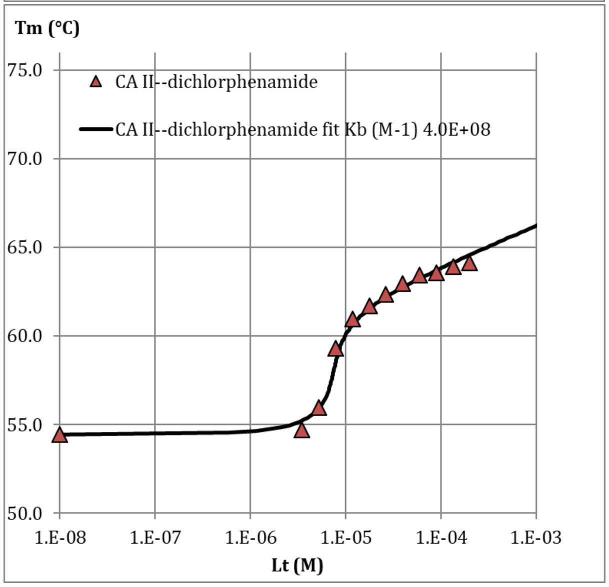

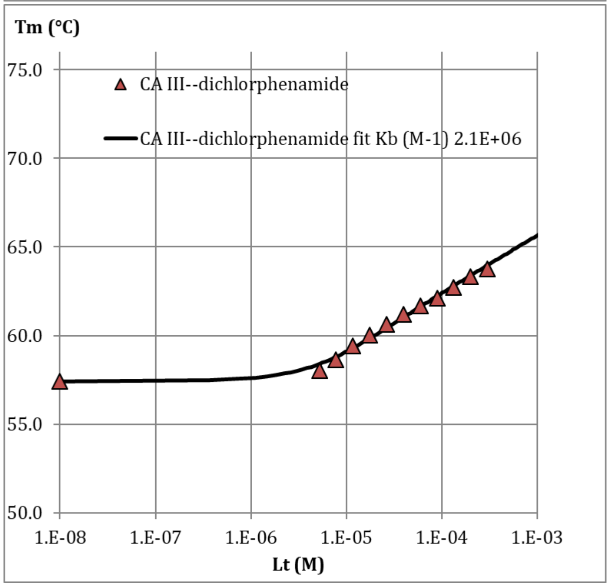

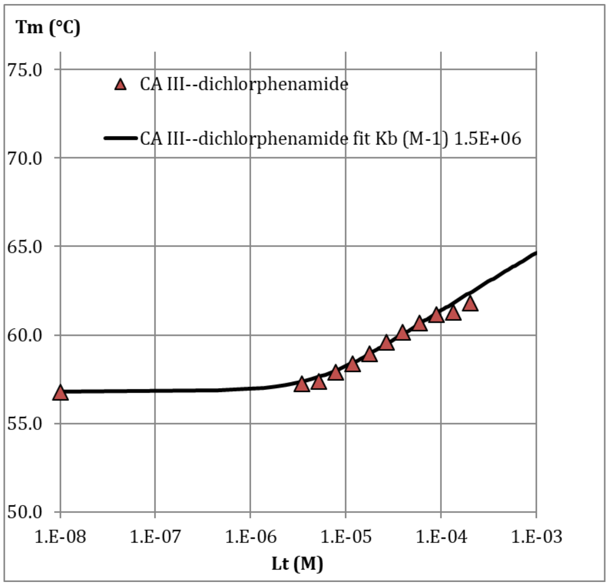

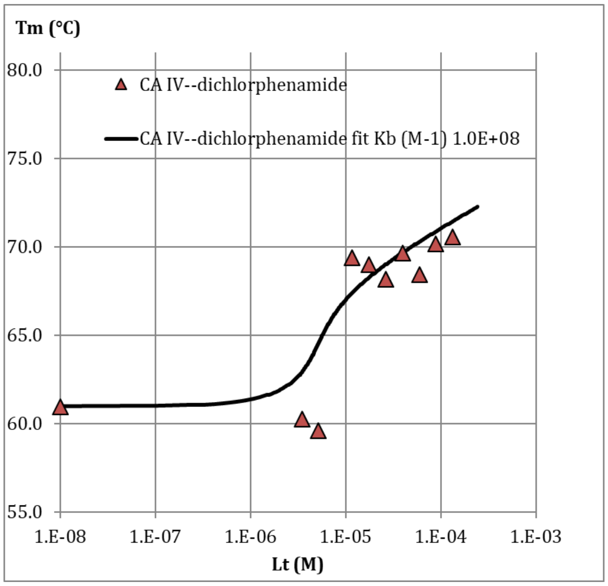
Tm (°C)
CA IV--dichlorphenamide
CA IV--dichlorphenamide fit Kb (M-1) 1.0E+08
Lt (M)

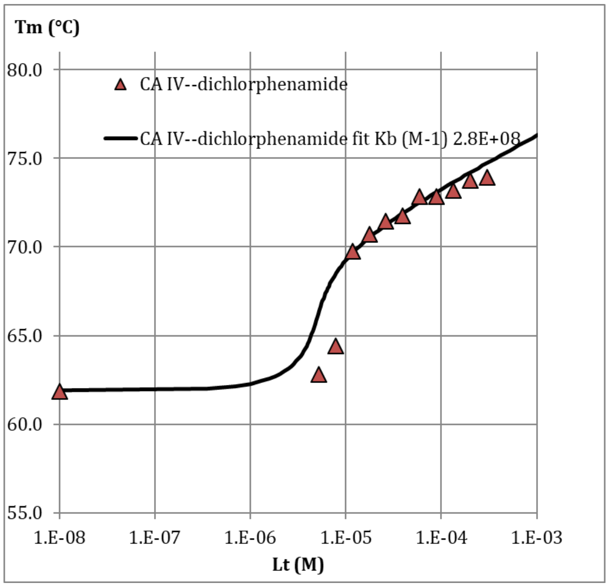
Tm (°C)
CA IV--dichlorphenamide
CA IV--dichlorphenamide fit Kb (M-1) 2.8E+08
Lt (M)

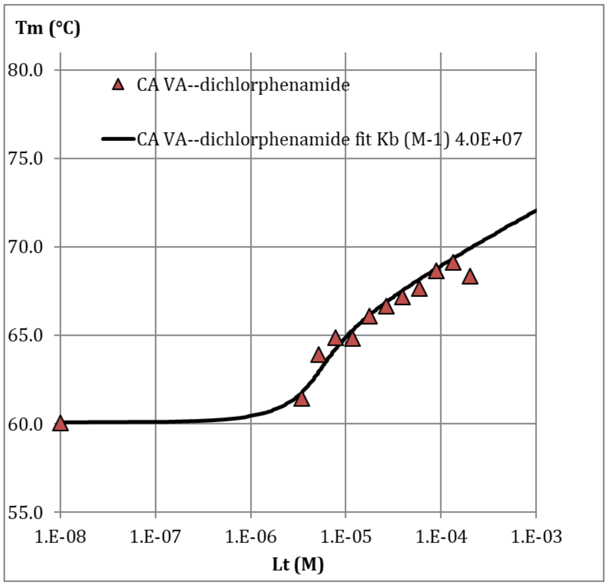
Tm (°C)
CA VA--dichlorphenamide
CA VA--dichlorphenamide fit Kb (M-1) 4.0E+07
Lt (M)

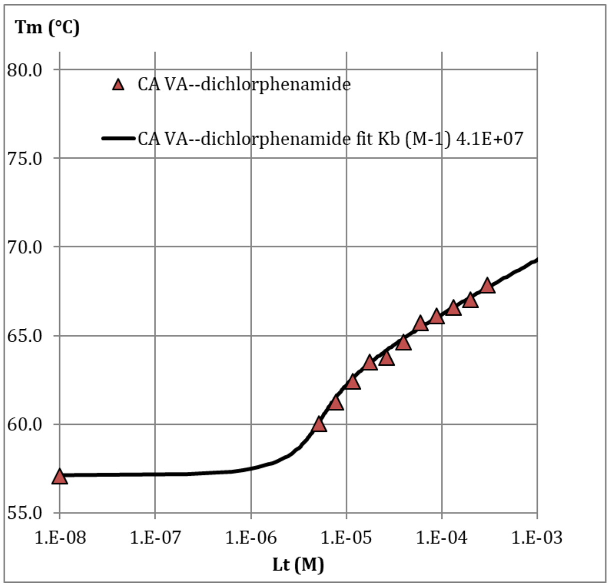
Tm (°C)
CA VA--dichlorphenamide
CA VA--dichlorphenamide fit Kb (M-1) 4.1E+07
Lt (M)

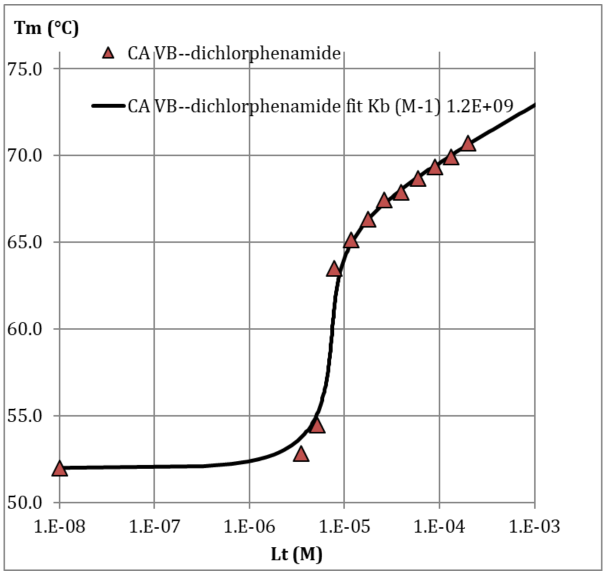
Tm (°C)
CA VB--dichlorphenamide
CA VB--dichlorphenamide fit Kb (M-1) 1.2E+09
Lt (M)

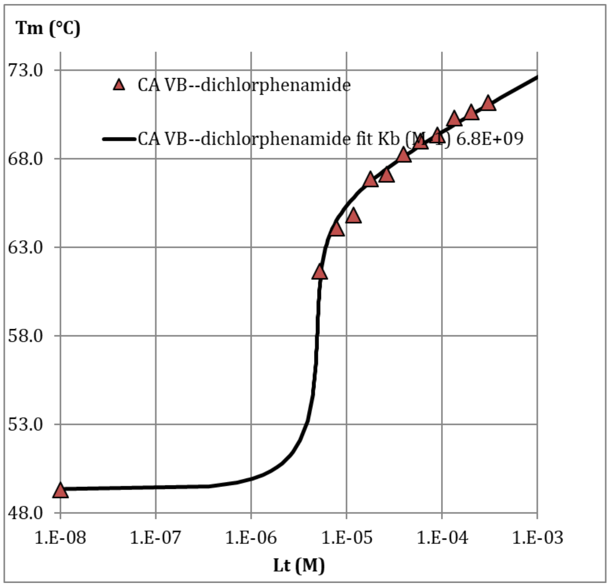
Tm (°C)
CA VB--dichlorphenamide
CA VB--dichlorphenamide fit Kb (M-1) 6.8E+09
Lt (M)

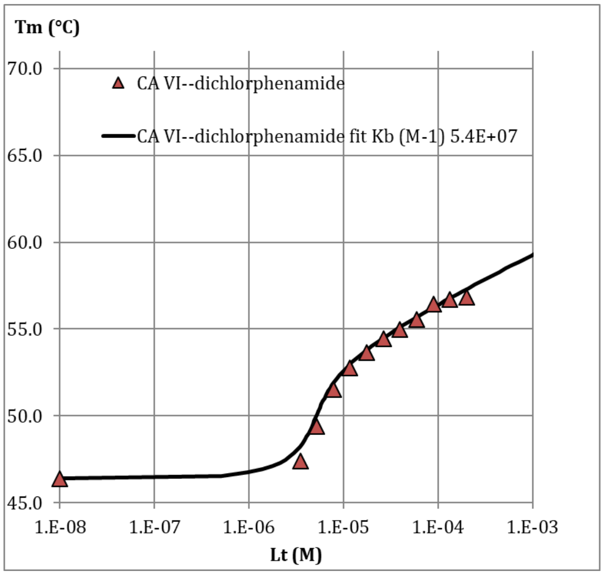
Tm (°C)
70.0
65.0
60.0
55.0
50.0
45.0
CA VI--dichlorphenamide
CA VI--dichlorphenamide fit Kb (M-1) 5.4E+07
1.E-08
1.E-07
1.E-06
1.E-05
1.E-04
1.E-03
Lt (M)

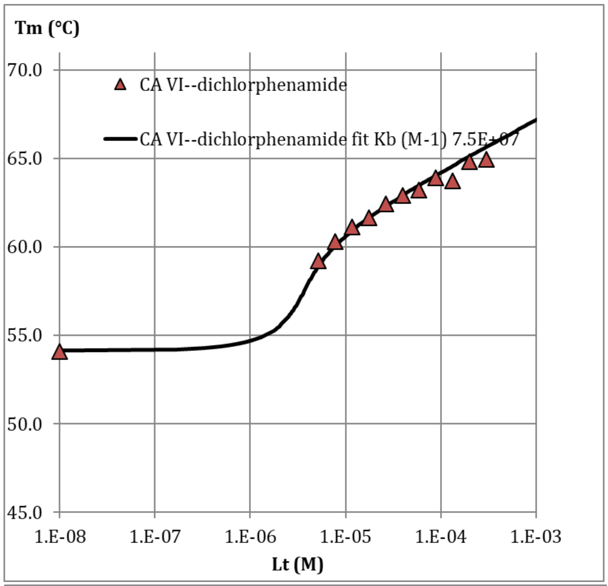
Tm (°C)
70.0
65.0
60.0
55.0
50.0
45.0
CA VI--dichlorphenamide
CA VI--dichlorphenamide fit Kb (M-1) 7.5E+07
1.E-08
1.E-07
1.E-06
1.E-05
1.E-04
1.E-03
Lt (M)

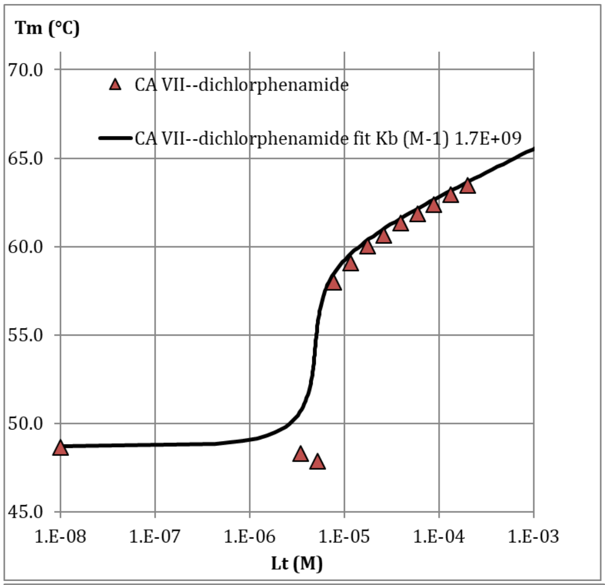
Tm (°C)
70.0
65.0
60.0
55.0
50.0
45.0
CA VII--dichlorphenamide
CA VII--dichlorphenamide fit Kb (M-1) 1.7E+09
1.E-08
1.E-07
1.E-06
1.E-05
1.E-04
1.E-03
Lt (M)

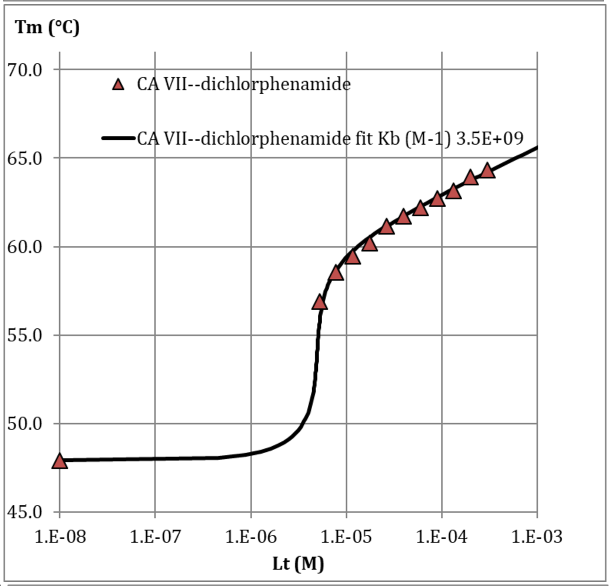
Tm (°C)
70.0
65.0
60.0
55.0
50.0
45.0
CA VII--dichlorphenamide
CA VII--dichlorphenamide fit Kb (M-1) 3.5E+09
1.E-08
1.E-07
1.E-06
1.E-05
1.E-04
1.E-03
Lt (M)

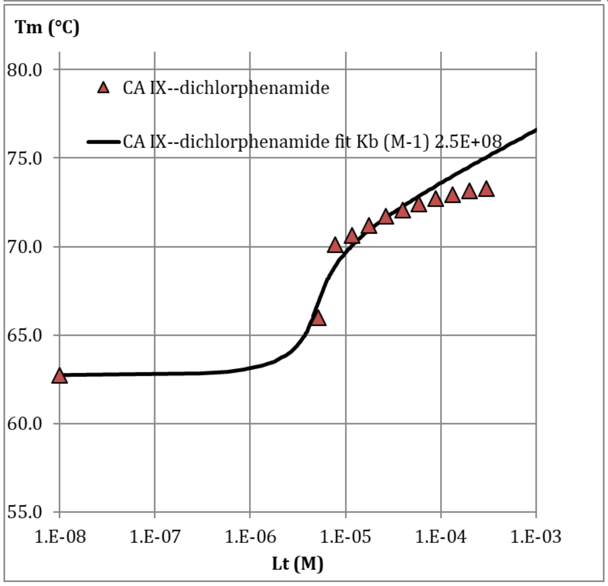
Tm (°C)
80.0
75.0
70.0
65.0
60.0
55.0
CA IX--dichlorphenamide
CA IX--dichlorphenamide fit Kb (M-1) 2.5E+08
1.E-08
1.E-07
1.E-06
1.E-05
1.E-04
1.E-03
Lt (M)

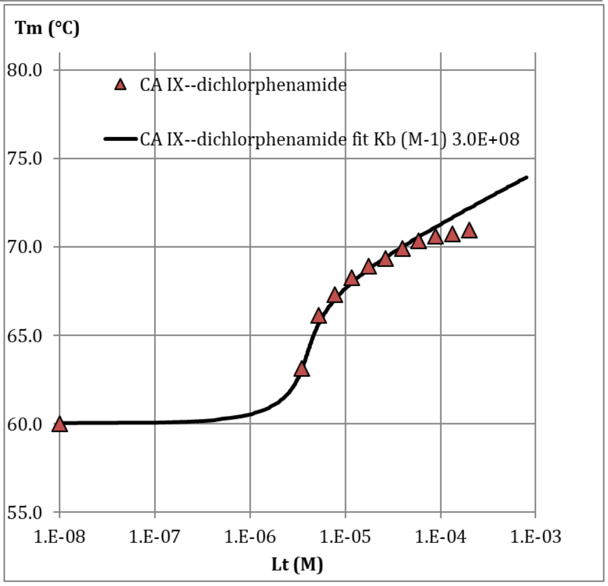
Tm (°C)
80.0
75.0
70.0
65.0
60.0
55.0
CA IX--dichlorphenamide
CA IX--dichlorphenamide fit Kb (M-1) 3.0E+08
1.E-08
1.E-07
1.E-06
1.E-05
1.E-04
1.E-03
Lt (M)

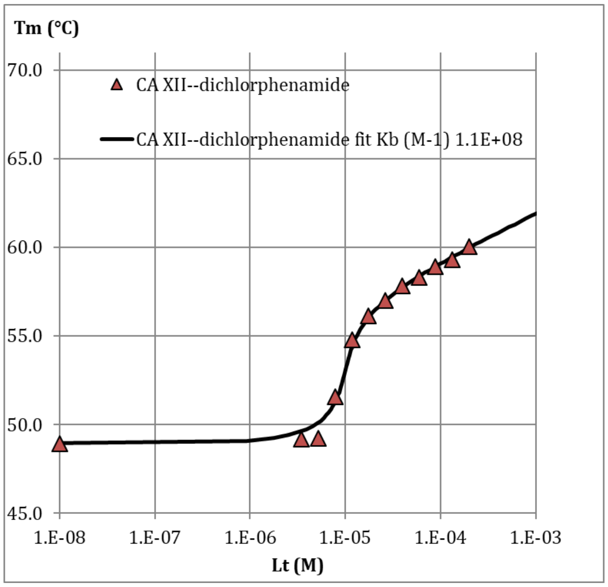
Tm (°C)
CA XII--dichlorphenamide
CA XII--dichlorphenamide fit Kb (M-1) 1.1E+08
Lt (M)

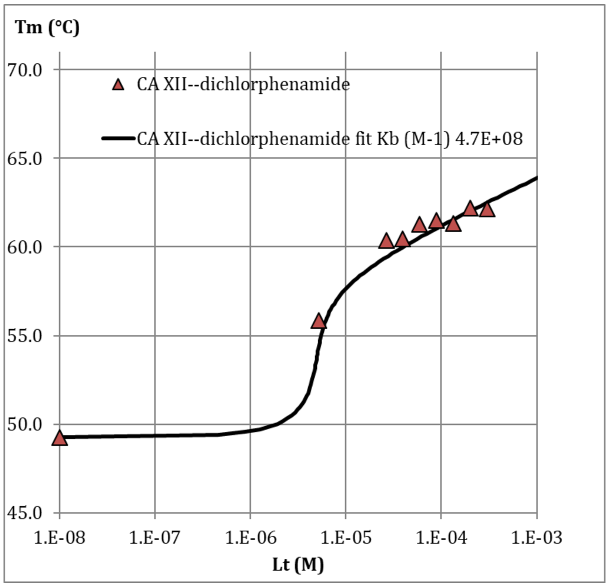
Tm (°C)
CA XII--dichlorphenamide
CA XII--dichlorphenamide fit Kb (M-1) 4.7E+08
Lt (M)

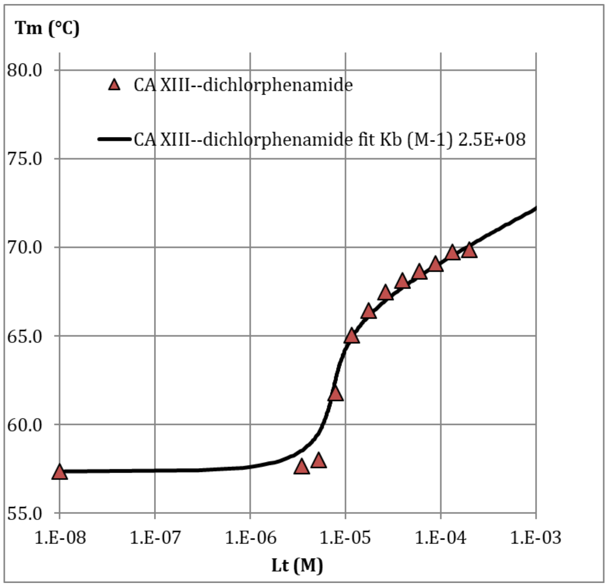
Tm (°C)
CA XIII--dichlorphenamide
CA XIII--dichlorphenamide fit Kb (M-1) 2.5E+08
Lt (M)

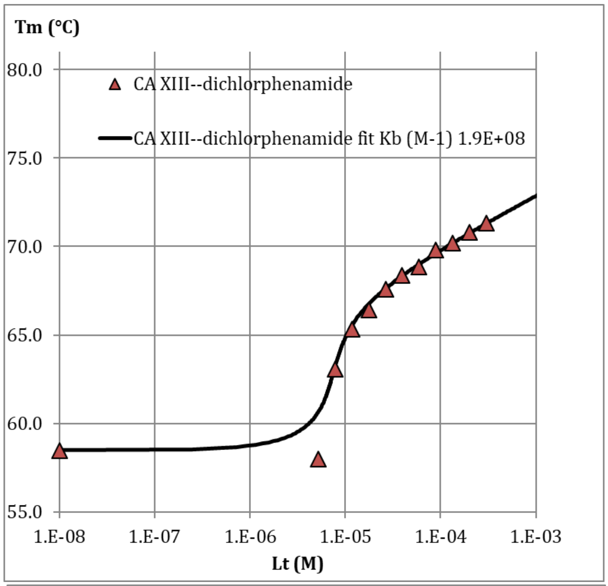
Tm (°C)
CA XIII--dichlorphenamide
CA XIII--dichlorphenamide fit Kb (M-1) 1.9E+08
Lt (M)

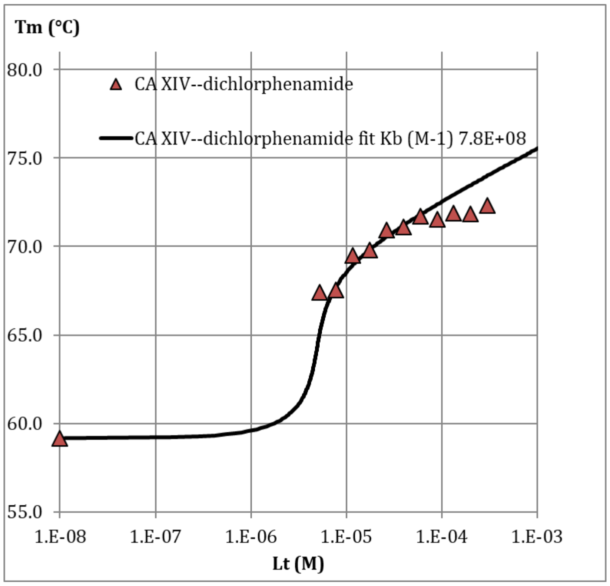
Tm (°C)
CA XIV--dichlorphenamide
CA XIV--dichlorphenamide fit Kb (M-1) 7.8E+08
Lt (M)

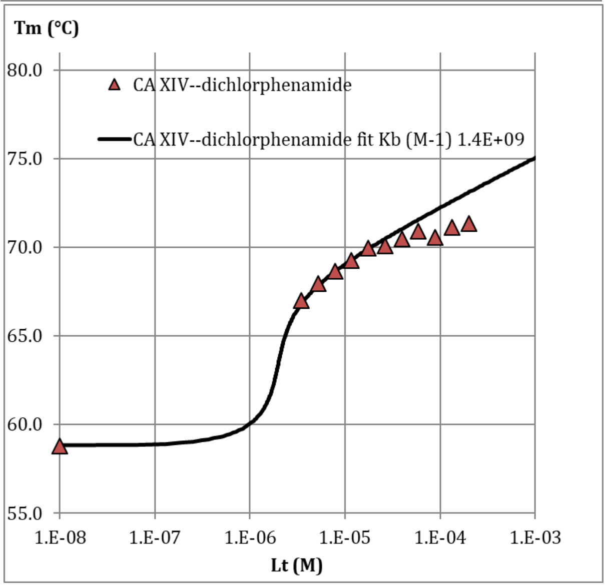
Tm (°C)
CA XIV--dichlorphenamide
CA XIV--dichlorphenamide fit Kb (M-1) 1.4E+09
Lt (M)

Isothermal titration calorimetry CAII – dichlorphenamide

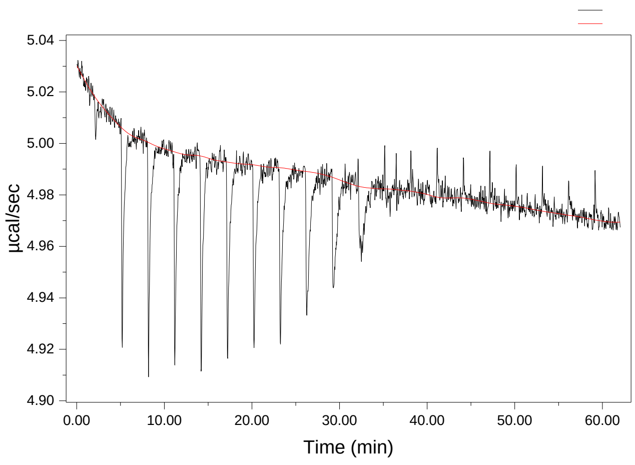

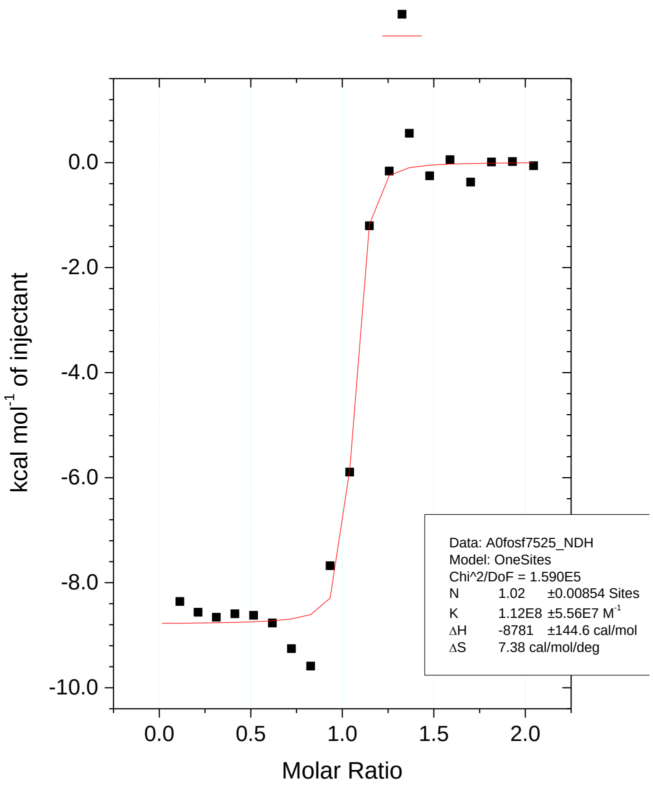

CAII – dichlorphenamide

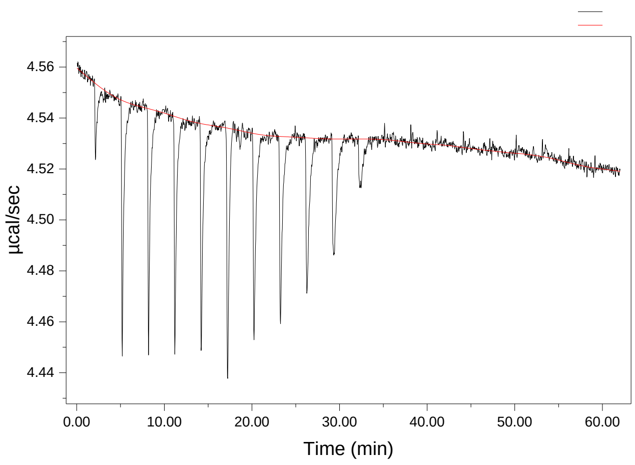

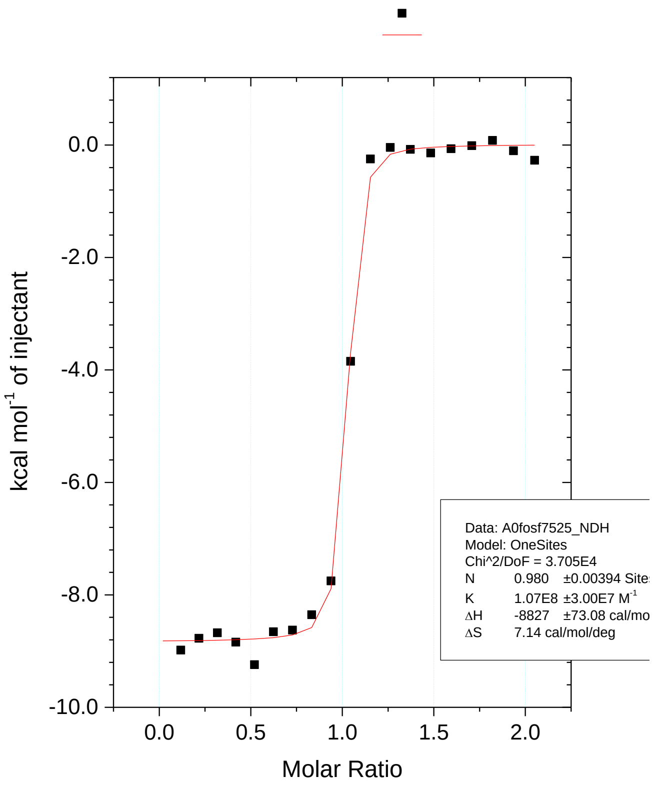

3. Chlorothiazide

Fluorecence-based thermal shift assay

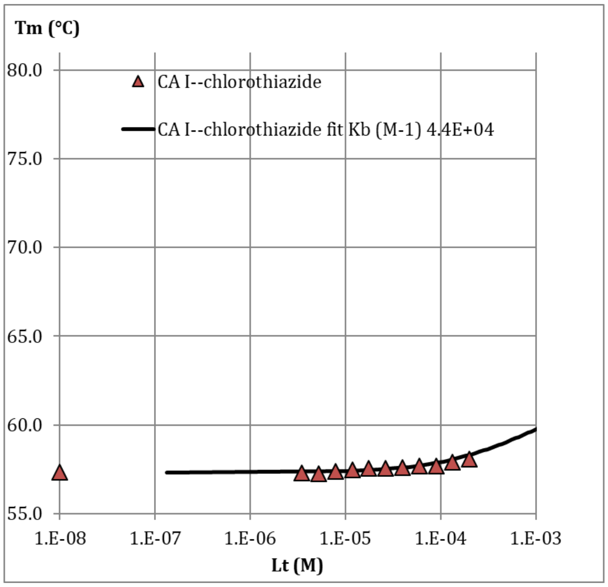

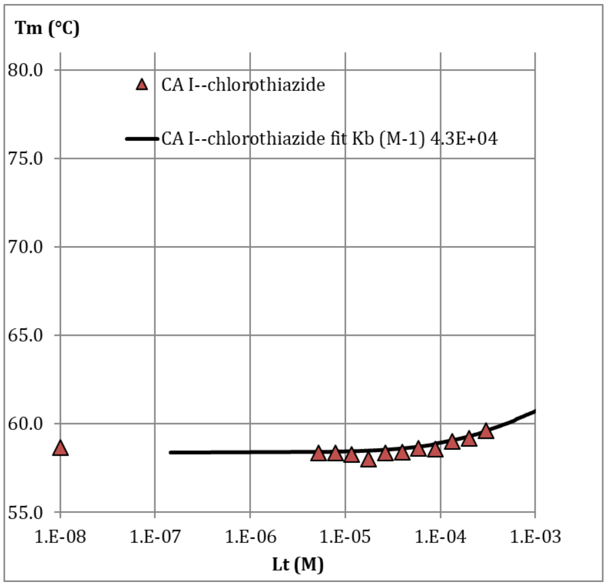

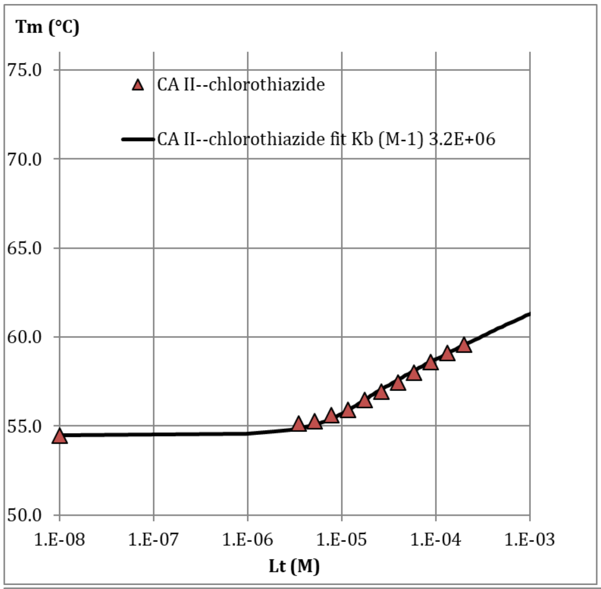

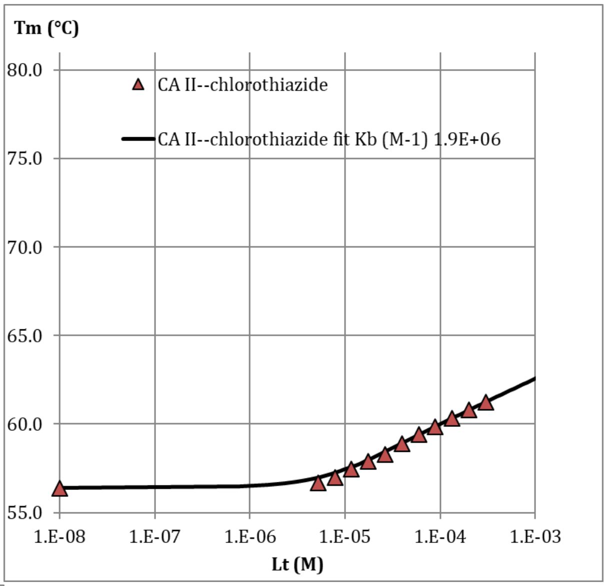

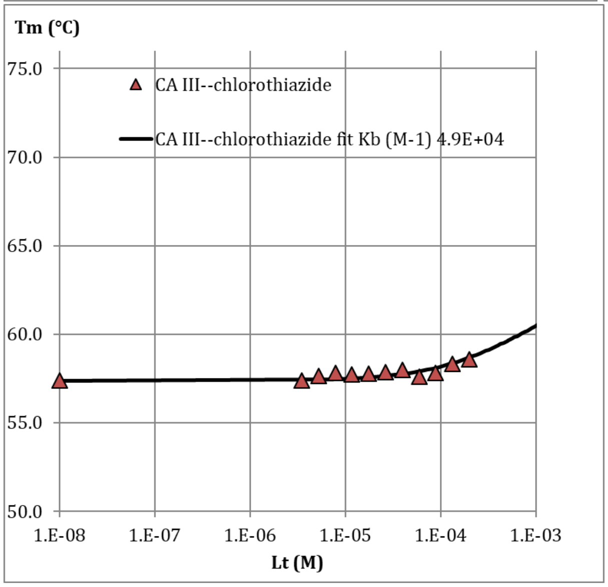

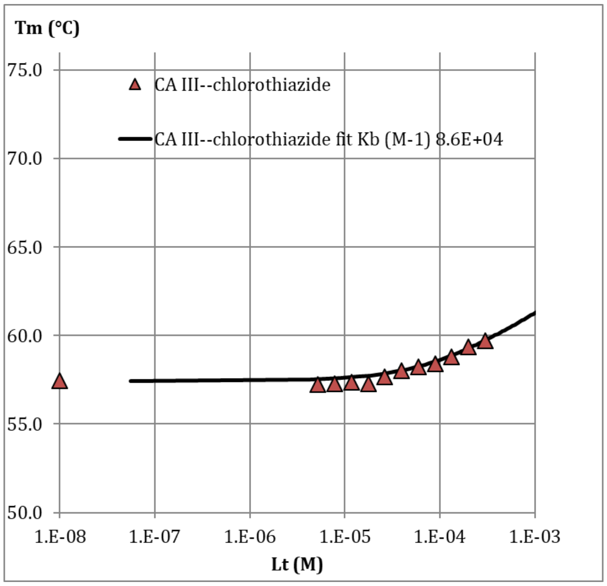

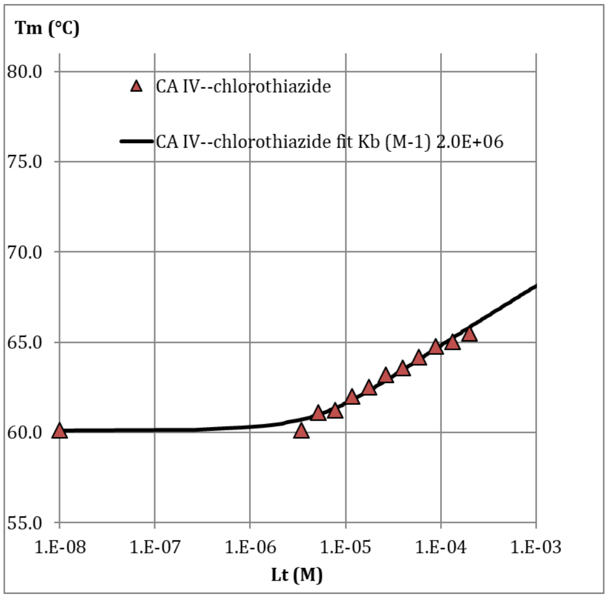
Tm (°C)
80.0
75.0
70.0
65.0
60.0
55.0
CA IV--chlorothiazide
CA IV--chlorothiazide fit Kb (M-1) 2.0E+06
1.E-08
1.E-07
1.E-06
1.E-05
1.E-04
1.E-03
Lt (M)

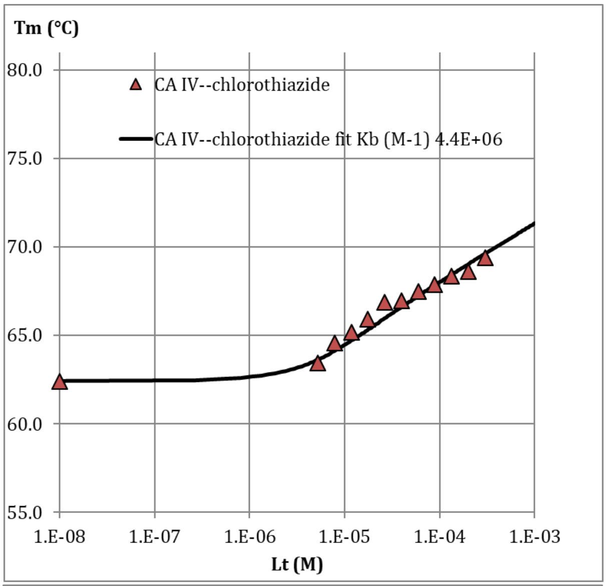
Tm (°C)
80.0
75.0
70.0
65.0
60.0
55.0
CA IV--chlorothiazide
CA IV--chlorothiazide fit Kb (M-1) 4.4E+06
1.E-08
1.E-07
1.E-06
1.E-05
1.E-04
1.E-03
Lt (M)

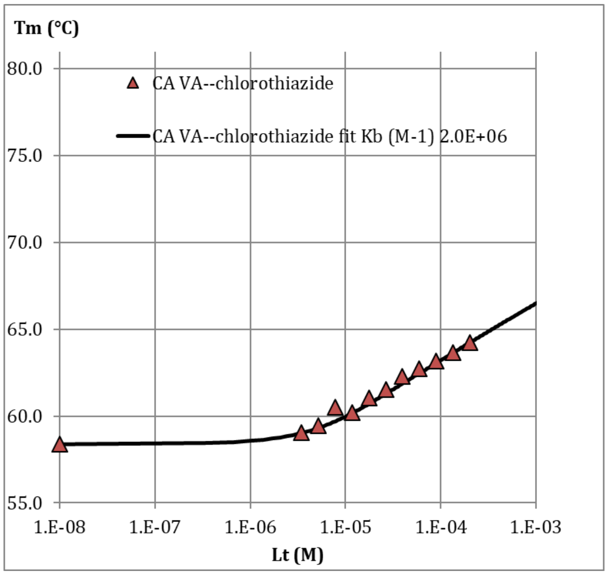
Tm (°C)
80.0
75.0
70.0
65.0
60.0
55.0
CA VA--chlorothiazide
CA VA--chlorothiazide fit Kb (M-1) 2.0E+06
1.E-08
1.E-07
1.E-06
1.E-05
1.E-04
1.E-03
Lt (M)

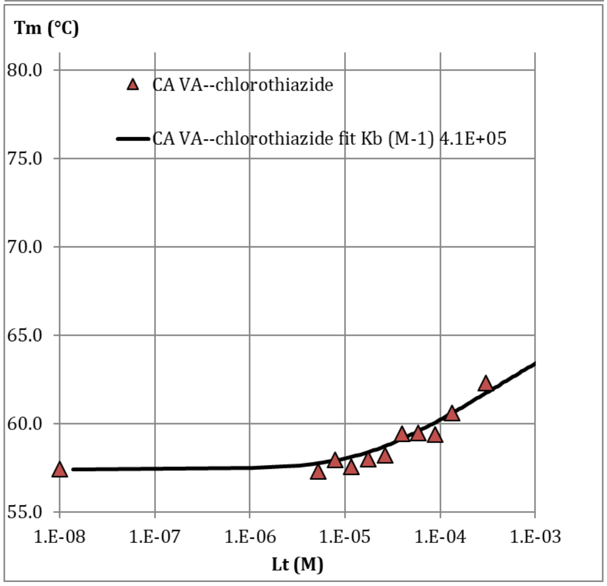
Tm (°C)
80.0
75.0
70.0
65.0
60.0
55.0
CA VA--chlorothiazide
CA VA--chlorothiazide fit Kb (M-1) 4.1E+05
1.E-08
1.E-07
1.E-06
1.E-05
1.E-04
1.E-03
Lt (M)

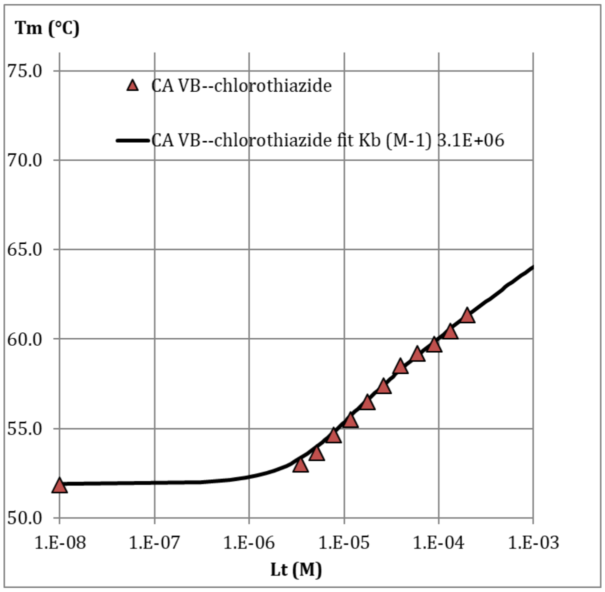
Tm (°C)
75.0
70.0
65.0
60.0
55.0
50.0
CA VB--chlorothiazide
CA VB--chlorothiazide fit Kb (M-1) 3.1E+06
1.E-08
1.E-07
1.E-06
1.E-05
1.E-04
1.E-03
Lt (M)

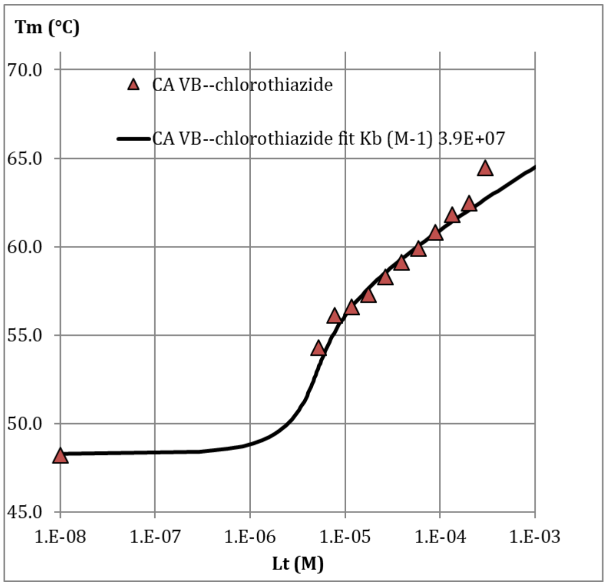
Tm (°C)
70.0
65.0
60.0
55.0
50.0
45.0
CA VB--chlorothiazide
CA VB--chlorothiazide fit Kb (M-1) 3.9E+07
1.E-08
1.E-07
1.E-06
1.E-05
1.E-04
1.E-03
Lt (M)

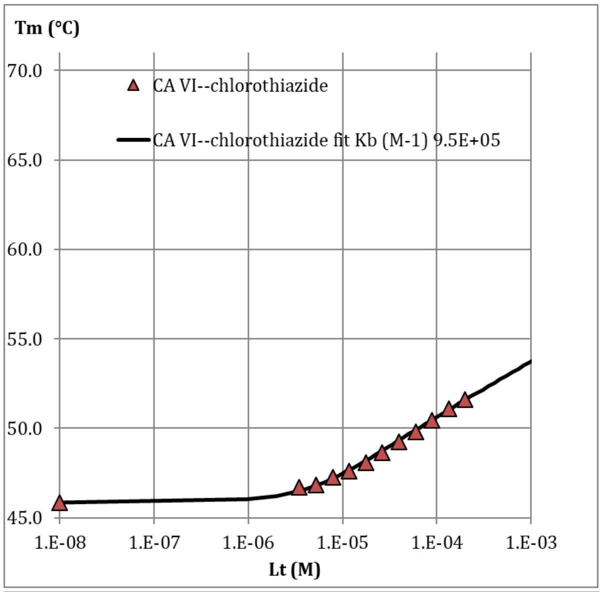
Tm (°C)
70.0
65.0
60.0
55.0
50.0
45.0
CA VI--chlorothiazide
CA VI--chlorothiazide fit Kb (M-1) 9.5E+05
1.E-08
1.E-07
1.E-06
1.E-05
1.E-04
1.E-03
Lt (M)

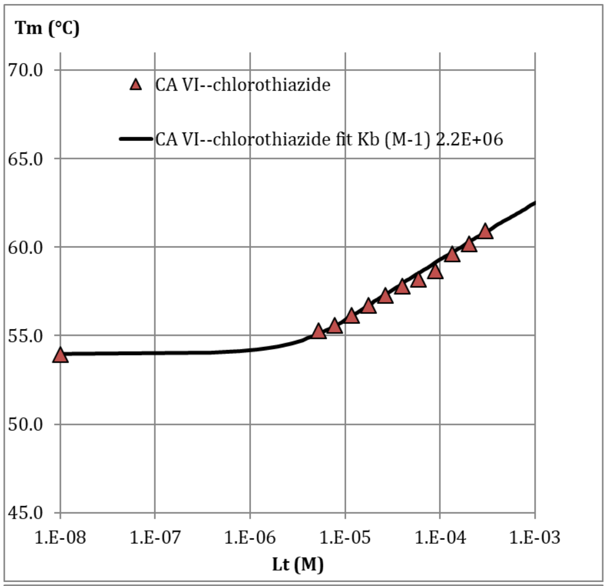
Tm (°C)
70.0
65.0
60.0
55.0
50.0
45.0
CA VI--chlorothiazide
CA VI--chlorothiazide fit Kb (M-1) 2.2E+06
1.E-08
1.E-07
1.E-06
1.E-05
1.E-04
1.E-03
Lt (M)

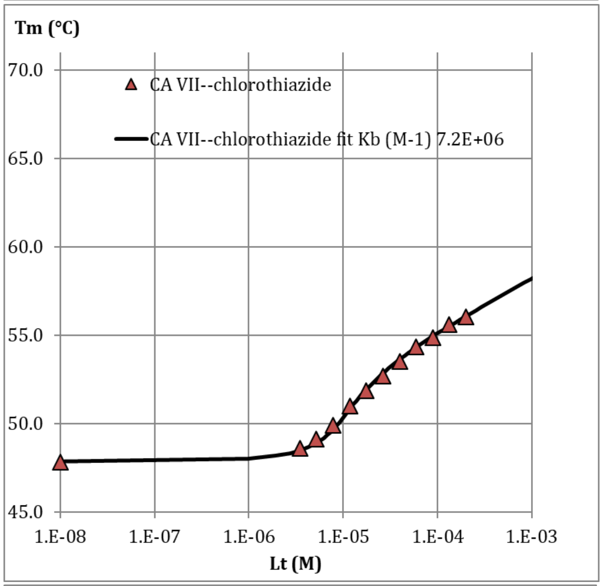
Tm (°C)
70.0
65.0
60.0
55.0
50.0
45.0
CA VII--chlorothiazide
CA VII--chlorothiazide fit Kb (M-1) 7.2E+06
1.E-08
1.E-07
1.E-06
1.E-05
1.E-04
1.E-03
Lt (M)

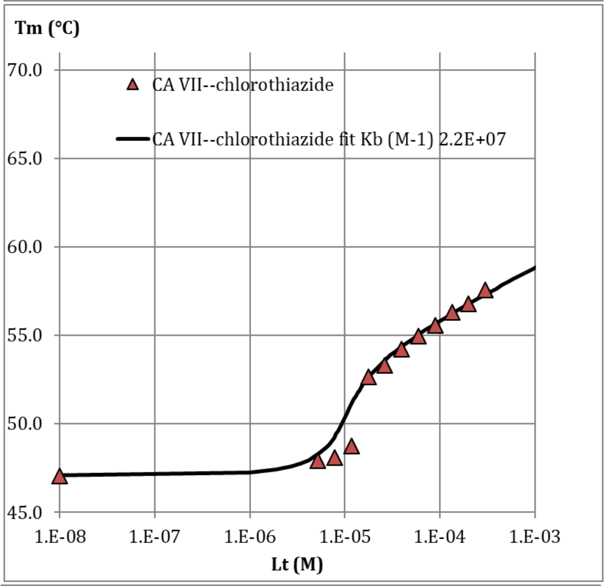
Tm (°C)
70.0
65.0
60.0
55.0
50.0
45.0
CA VII--chlorothiazide
CA VII--chlorothiazide fit Kb (M-1) 2.2E+07
1.E-08
1.E-07
1.E-06
1.E-05
1.E-04
1.E-03
Lt (M)

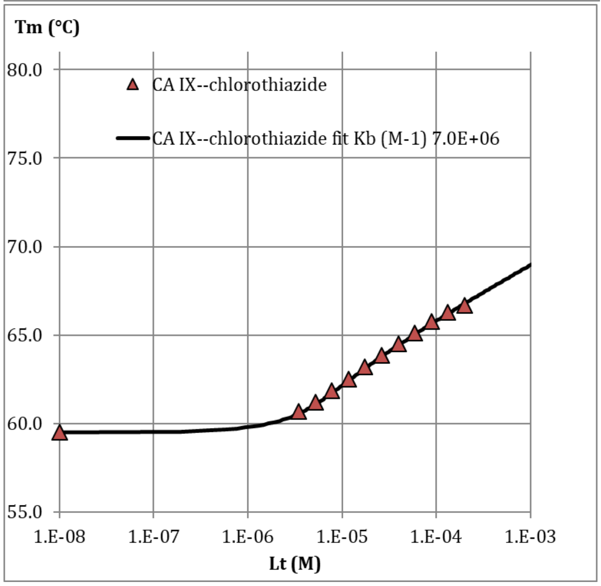
Tm (°C)
80.0
75.0
70.0
65.0
60.0
55.0
CA IX--chlorothiazide
CA IX--chlorothiazide fit Kb (M-1) 7.0E+06
1.E-08
1.E-07
1.E-06
1.E-05
1.E-04
1.E-03
Lt (M)

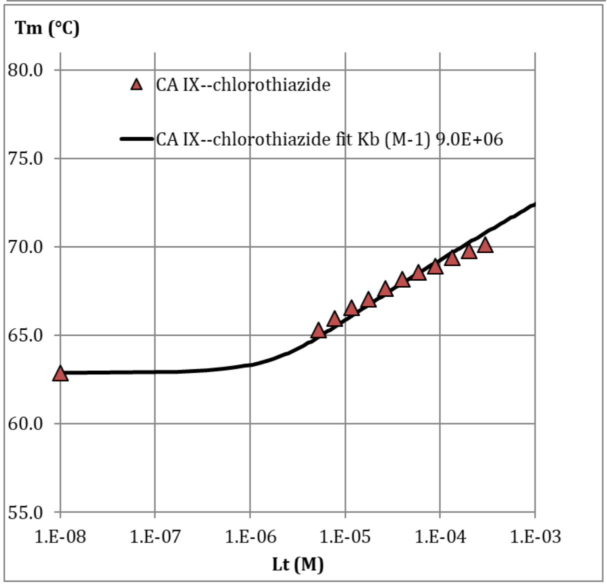
Tm (°C)
80.0
75.0
70.0
65.0
60.0
55.0
CA IX--chlorothiazide
CA IX--chlorothiazide fit Kb (M-1) 9.0E+06
1.E-08
1.E-07
1.E-06
1.E-05
1.E-04
1.E-03
Lt (M)

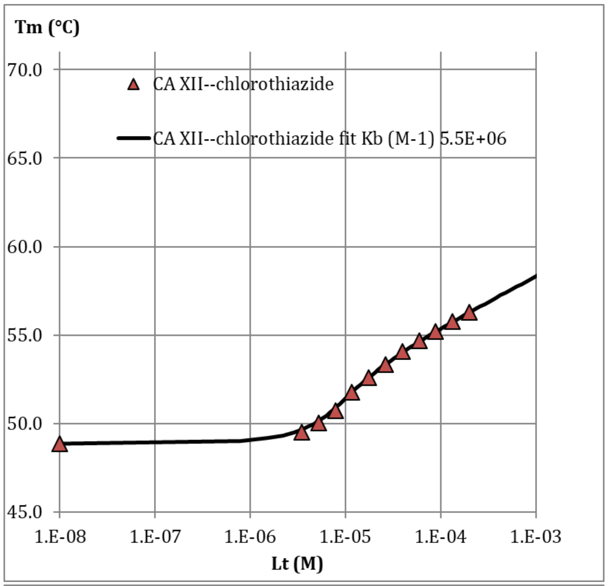
Tm (°C)
CA XII--chlorothiazide
CA XII--chlorothiazide fit Kb (M-1) 5.5E+06
Lt (M)

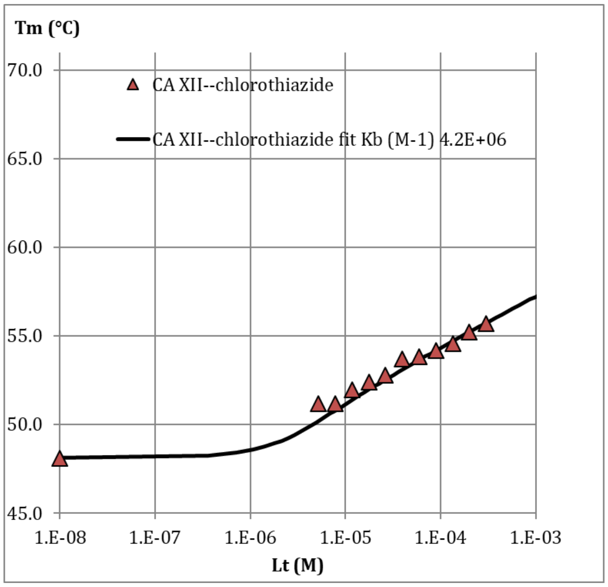
Tm (°C)
CA XII--chlorothiazide
CA XII--chlorothiazide fit Kb (M-1) 4.2E+06
Lt (M)

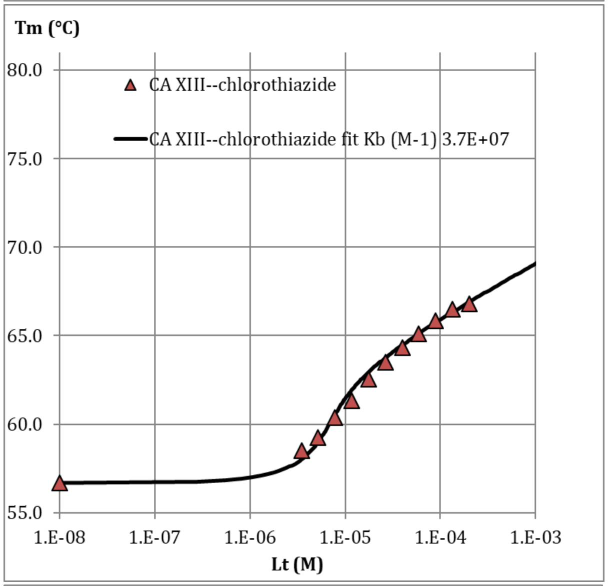
Tm (°C)
CA XIII--chlorothiazide
CA XIII--chlorothiazide fit Kb (M-1) 3.7E+07
Lt (M)

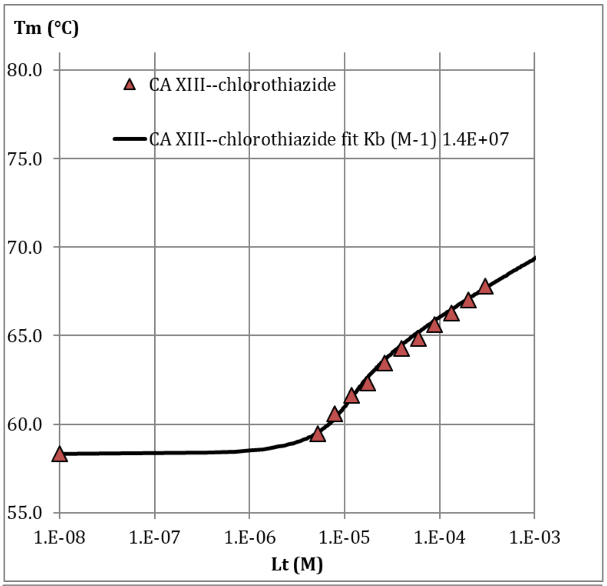
Tm (°C)
CA XIII--chlorothiazide
CA XIII--chlorothiazide fit Kb (M-1) 1.4E+07
Lt (M)

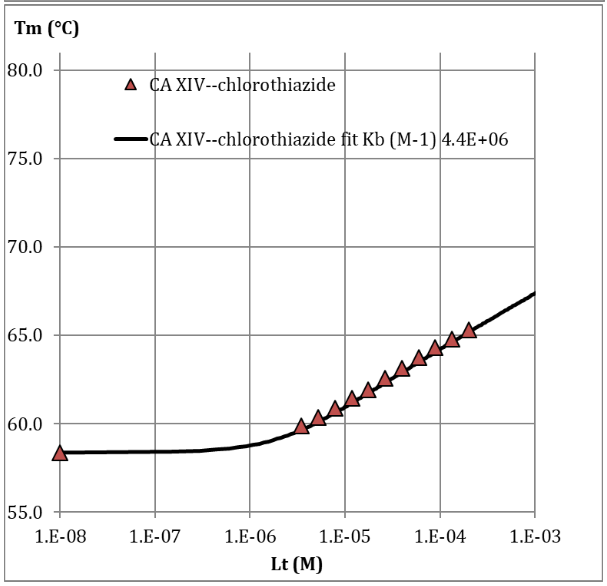
Tm (°C)
CA XIV--chlorothiazide
CA XIV--chlorothiazide fit Kb (M-1) 4.4E+06
Lt (M)

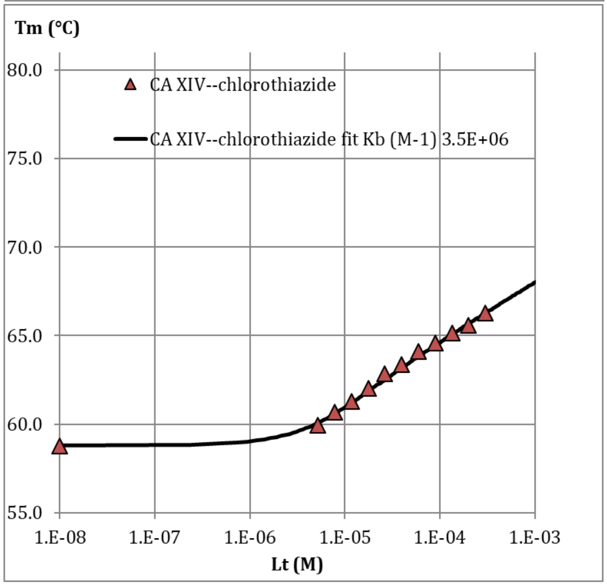
Tm (°C)
CA XIV--chlorothiazide
CA XIV--chlorothiazide fit Kb (M-1) 3.5E+06
Lt (M)

CO_2 hydration activity inhibition:

CA II - chlorothiazide

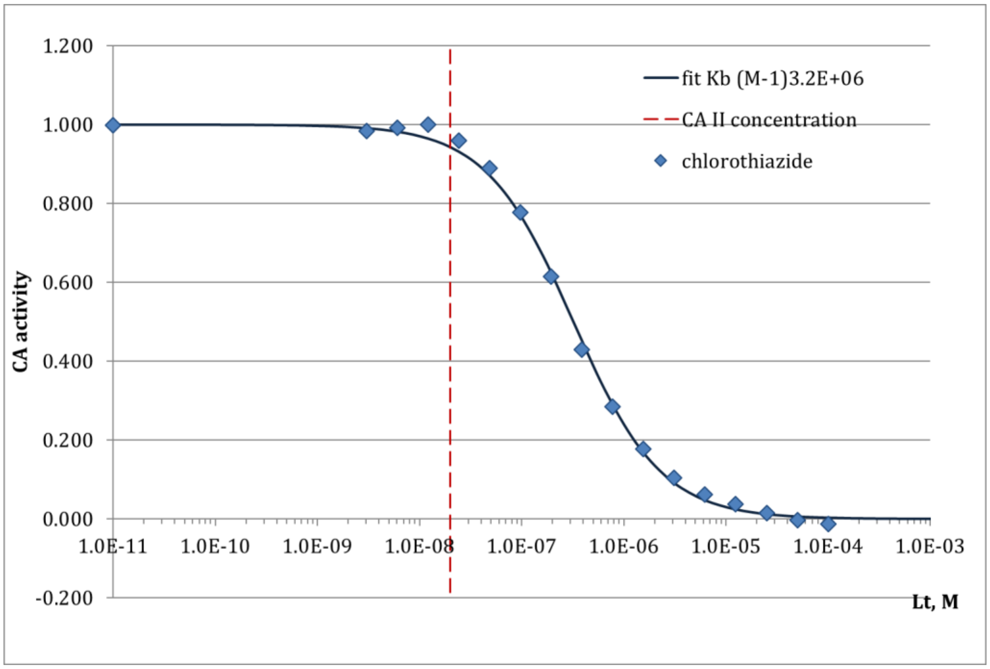

CA IV - chlorothiazide

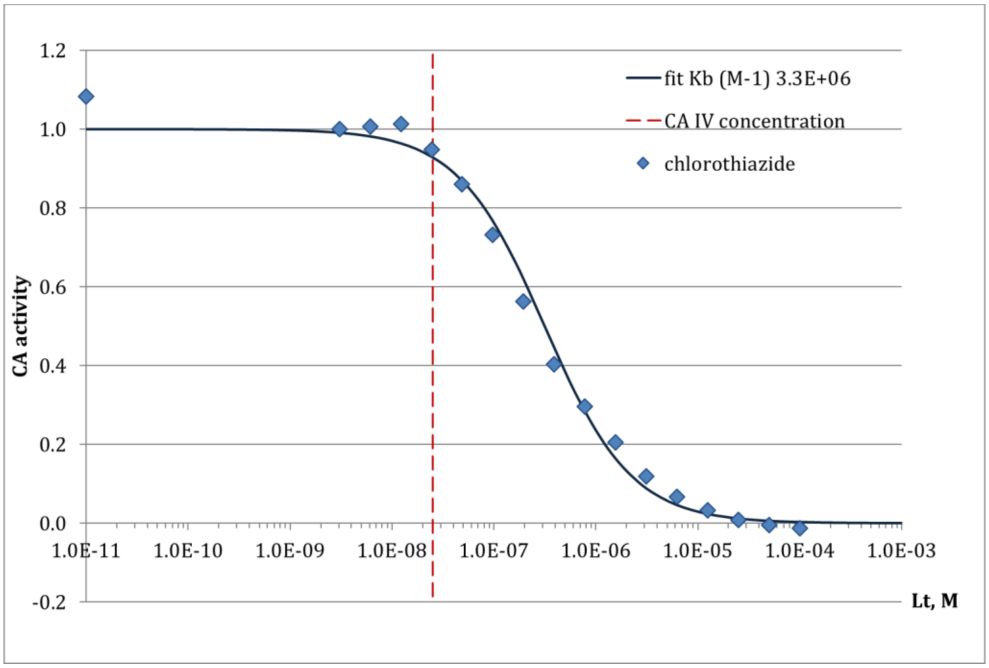

Isothermal titration calorimetry

CAII – chlorothiazide

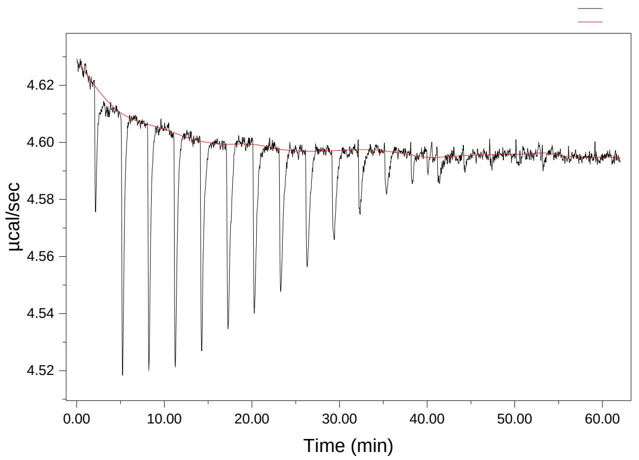

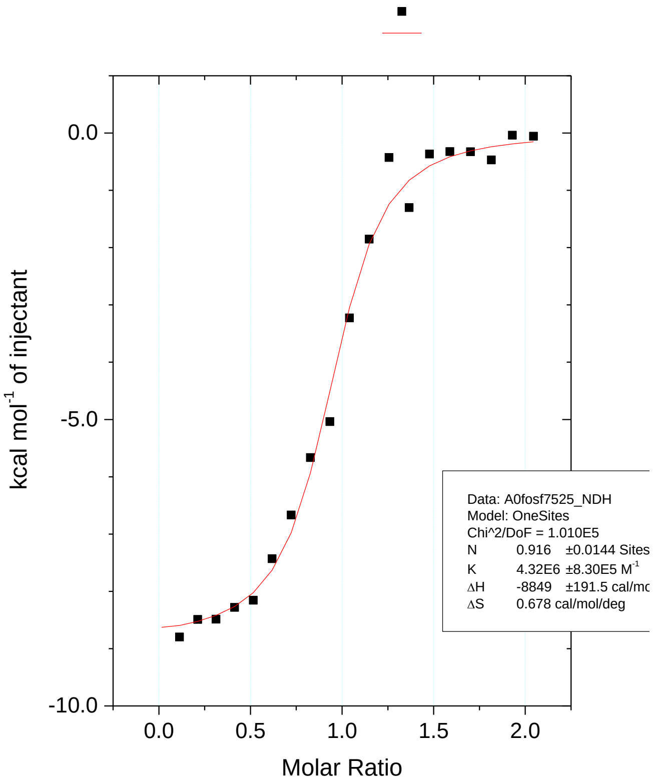

CAII – chlorothiazide

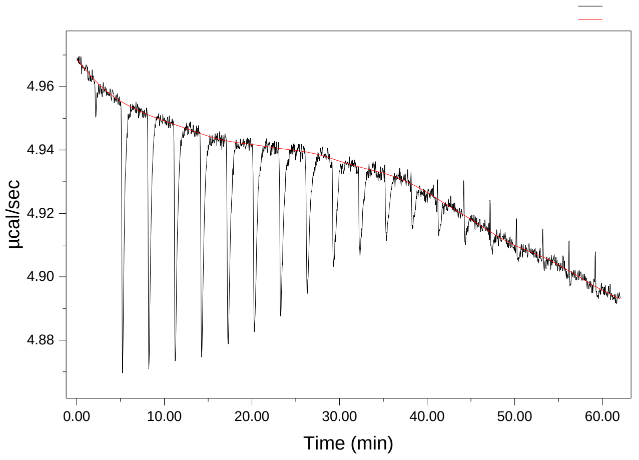

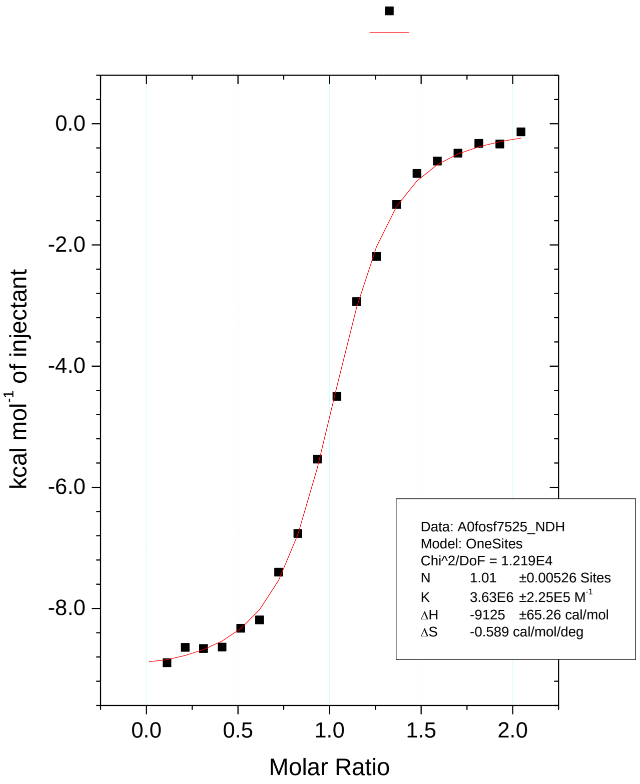

4. Hydrochlorothiazide

Fluorecence-based thermal shift assay

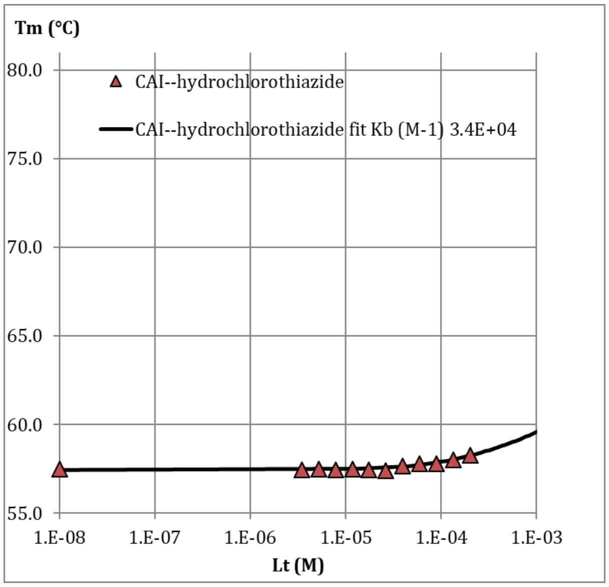

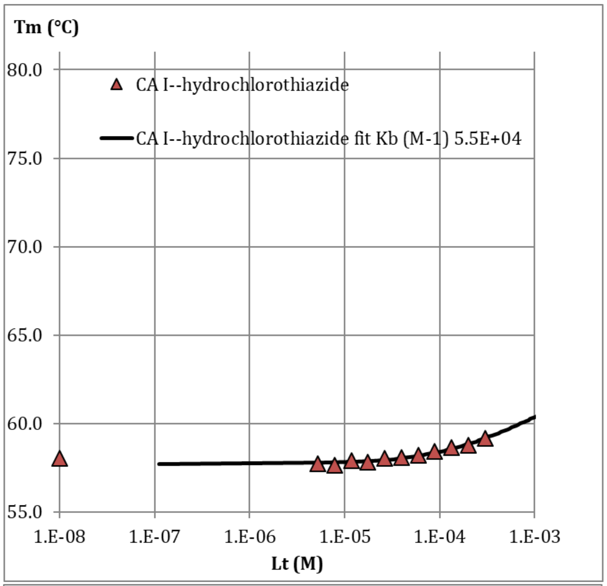

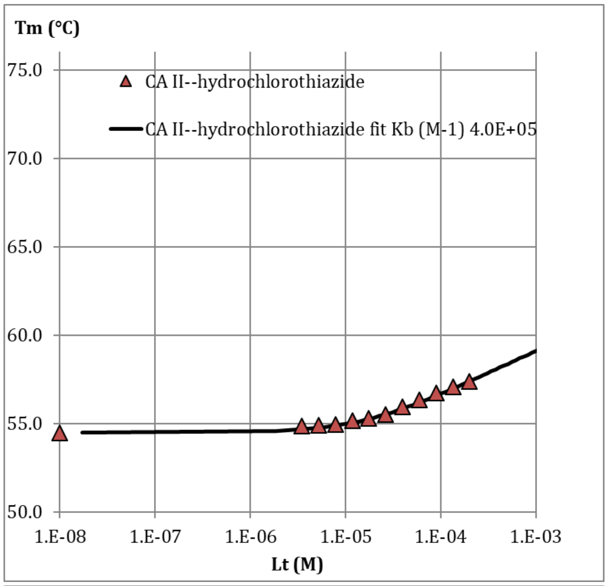

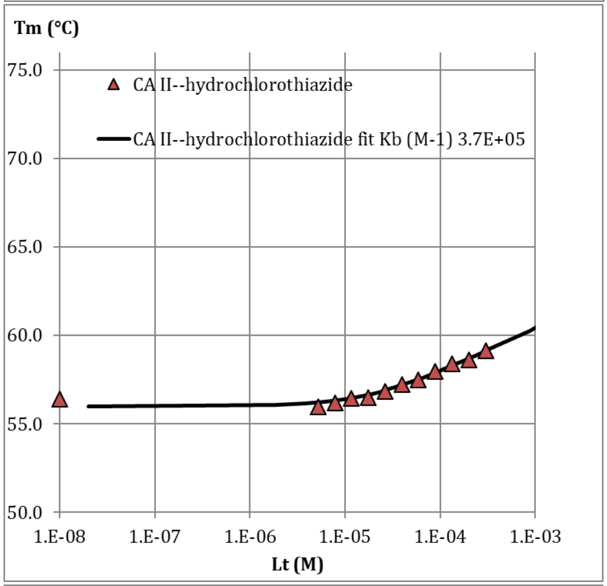

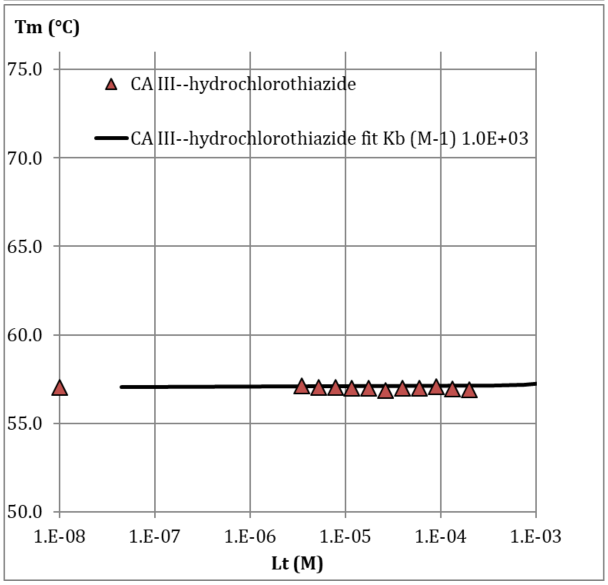

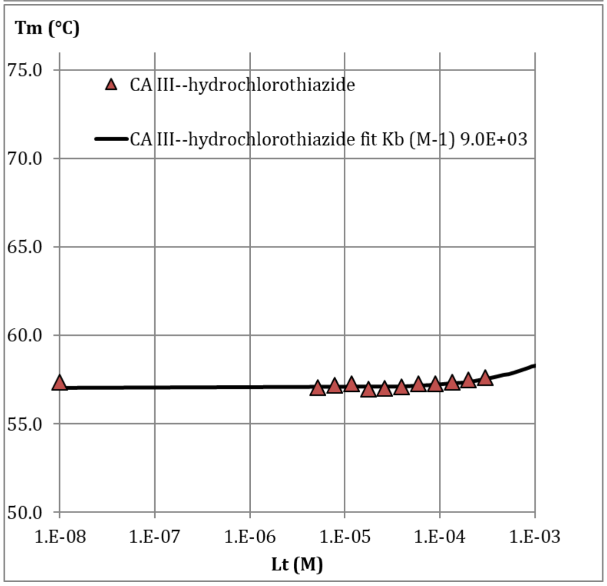

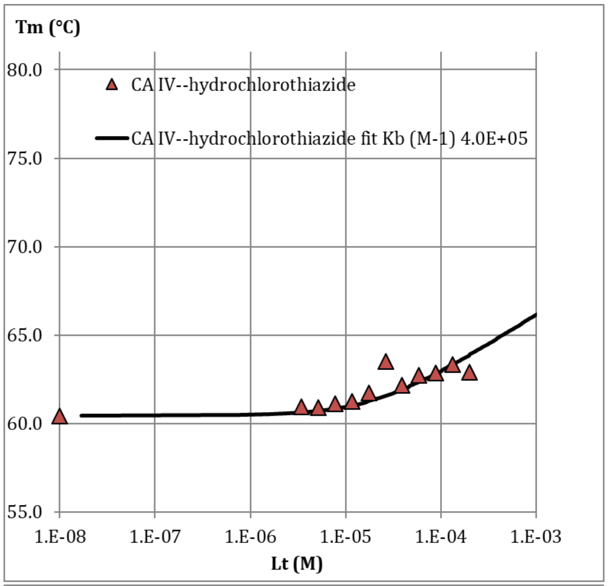
Tm (°C)
80.0
75.0
70.0
65.0
60.0
55.0
CA IV--hydrochlorothiazide
CA IV--hydrochlorothiazide fit Kb (M-1) 4.0E+05
1.E-08
1.E-07
1.E-06
1.E-05
1.E-04
1.E-03
Lt (M)

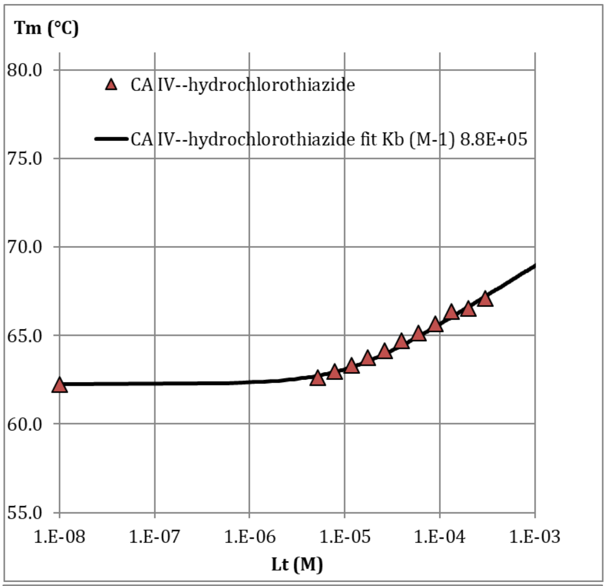
Tm (°C)
80.0
75.0
70.0
65.0
60.0
55.0
CA IV--hydrochlorothiazide
CA IV--hydrochlorothiazide fit Kb (M-1) 8.8E+05
1.E-08
1.E-07
1.E-06
1.E-05
1.E-04
1.E-03
Lt (M)

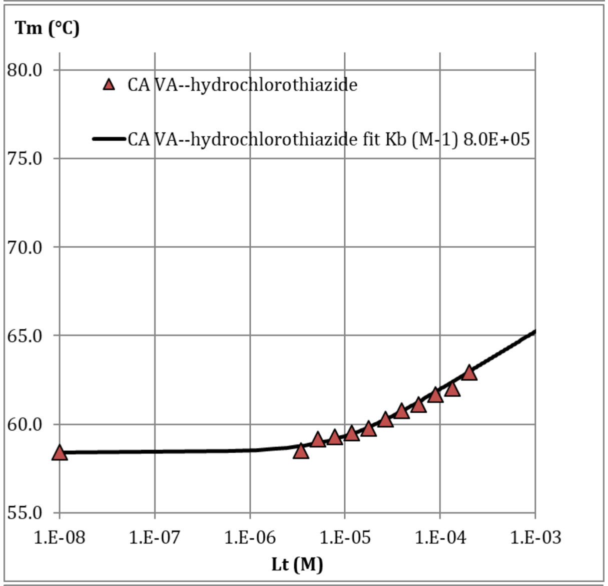
Tm (°C)
80.0
75.0
70.0
65.0
60.0
55.0
CA VA--hydrochlorothiazide
CA VA--hydrochlorothiazide fit Kb (M-1) 8.0E+05
1.E-08
1.E-07
1.E-06
1.E-05
1.E-04
1.E-03
Lt (M)

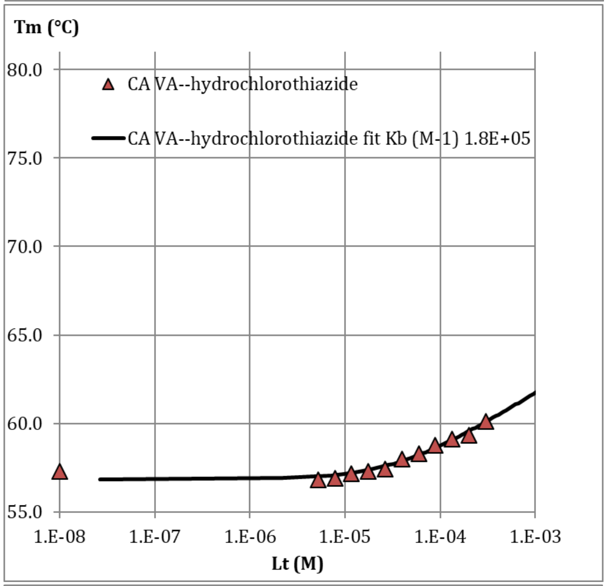
Tm (°C)
80.0
75.0
70.0
65.0
60.0
55.0
CA VA--hydrochlorothiazide
CA VA--hydrochlorothiazide fit Kb (M-1) 1.8E+05
1.E-08
1.E-07
1.E-06
1.E-05
1.E-04
1.E-03
Lt (M)

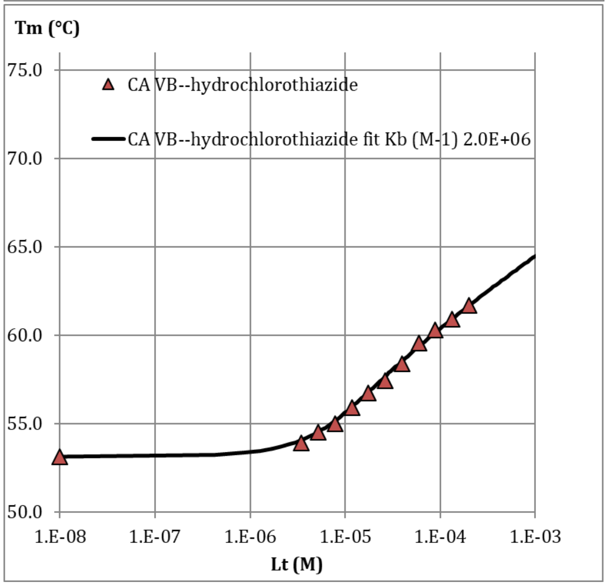
Tm (°C)
75.0
70.0
65.0
60.0
55.0
50.0
CA VB--hydrochlorothiazide
CA VB--hydrochlorothiazide fit Kb (M-1) 2.0E+06
1.E-08
1.E-07
1.E-06
1.E-05
1.E-04
1.E-03
Lt (M)

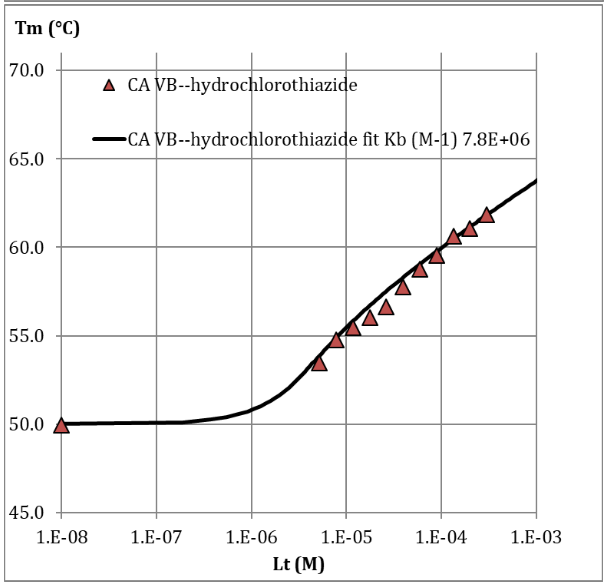
Tm (°C)
70.0
65.0
60.0
55.0
50.0
45.0
CA VB--hydrochlorothiazide
CA VB--hydrochlorothiazide fit Kb (M-1) 7.8E+06
1.E-08
1.E-07
1.E-06
1.E-05
1.E-04
1.E-03
Lt (M)

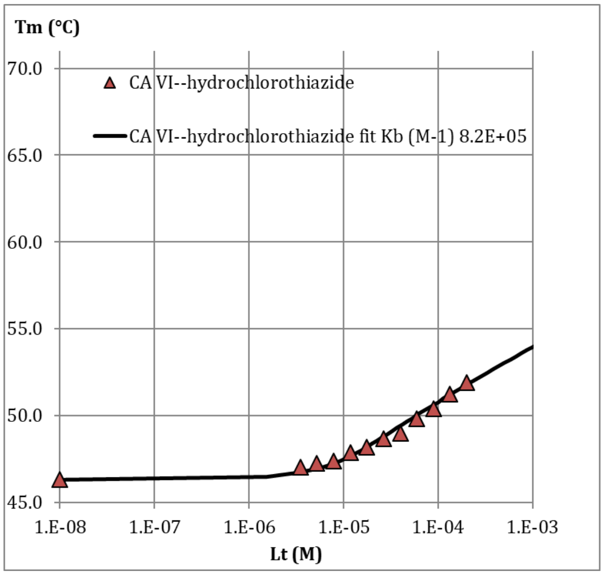
Tm (°C)
70.0
65.0
60.0
55.0
50.0
45.0
CA VI--hydrochlorothiazide
CA VI--hydrochlorothiazide fit Kb (M-1) 8.2E+05
1.E-08
1.E-07
1.E-06
1.E-05
1.E-04
1.E-03
Lt (M)

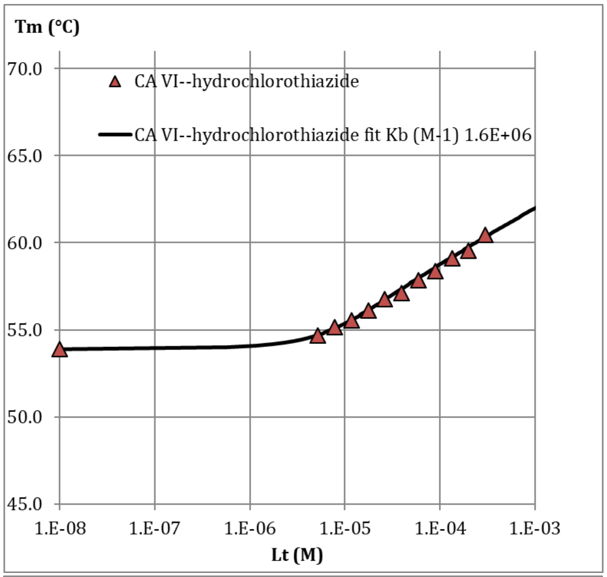
Tm (°C)
70.0
65.0
60.0
55.0
50.0
45.0
CA VI--hydrochlorothiazide
CA VI--hydrochlorothiazide fit Kb (M-1) 1.6E+06
1.E-08
1.E-07
1.E-06
1.E-05
1.E-04
1.E-03
Lt (M)

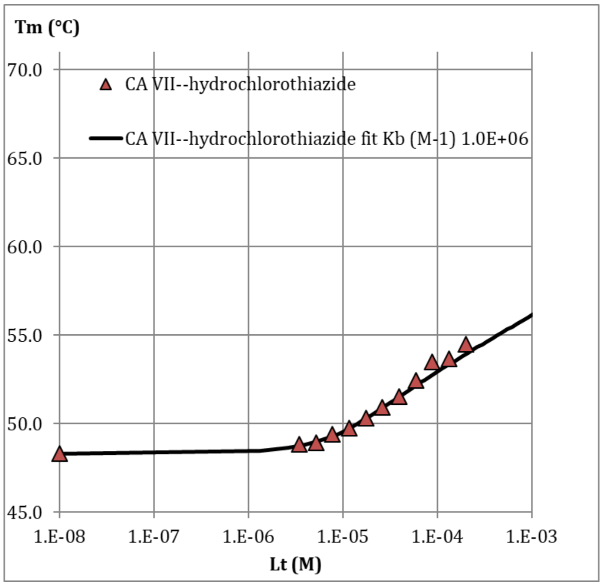
Tm (°C)
70.0
65.0
60.0
55.0
50.0
45.0
CA VII--hydrochlorothiazide
CA VII--hydrochlorothiazide fit Kb (M-1) 1.0E+06
1.E-08
1.E-07
1.E-06
1.E-05
1.E-04
1.E-03
Lt (M)

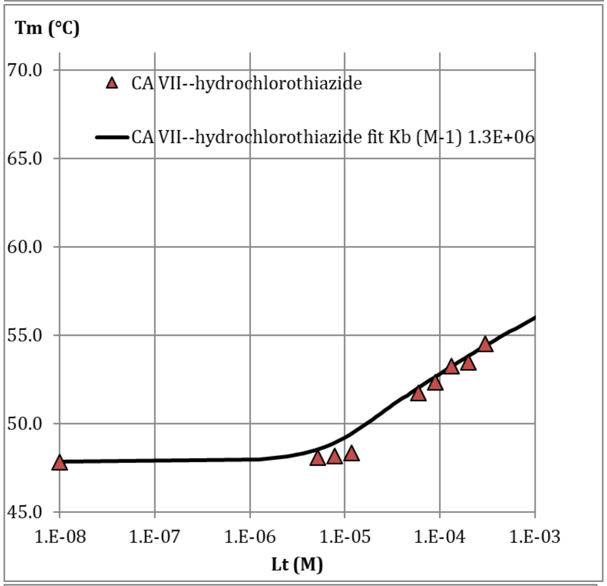
Tm (°C)
70.0
65.0
60.0
55.0
50.0
45.0
CA VII--hydrochlorothiazide
CA VII--hydrochlorothiazide fit Kb (M-1) 1.3E+06
1.E-08
1.E-07
1.E-06
1.E-05
1.E-04
1.E-03
Lt (M)

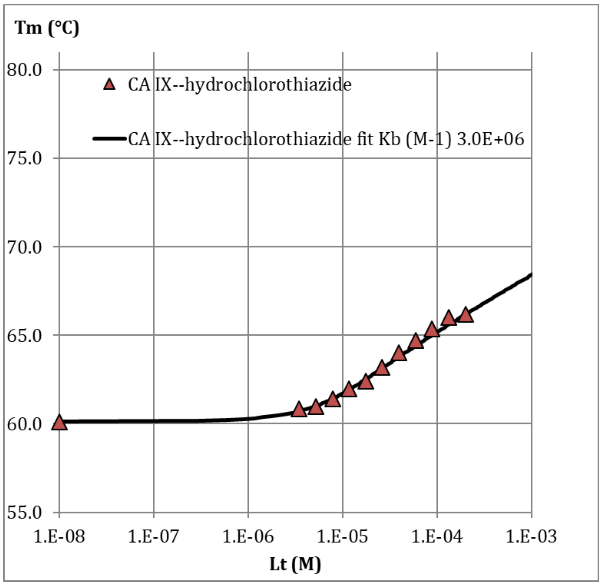
Tm (°C)
80.0
75.0
70.0
65.0
60.0
55.0
CA IX--hydrochlorothiazide
CA IX--hydrochlorothiazide fit Kb (M-1) 3.0E+06
1.E-08
1.E-07
1.E-06
1.E-05
1.E-04
1.E-03
Lt (M)

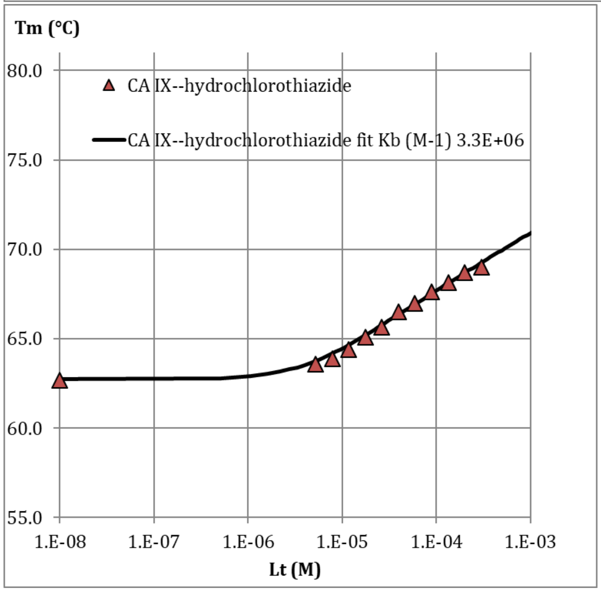
Tm (°C)
80.0
75.0
70.0
65.0
60.0
55.0
CA IX--hydrochlorothiazide
CA IX--hydrochlorothiazide fit Kb (M-1) 3.3E+06
1.E-08
1.E-07
1.E-06
1.E-05
1.E-04
1.E-03
Lt (M)

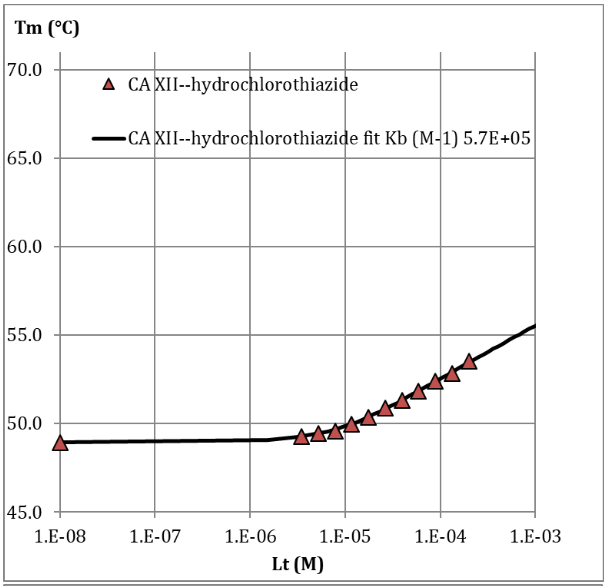
Tm (°C)
CA XII--hydrochlorothiazide
CA XII--hydrochlorothiazide fit Kb (M-1) 5.7E+05
70.0
65.0
60.0
55.0
50.0
45.0
1.E-08
1.E-07
1.E-06
1.E-05
1.E-04
1.E-03
Lt (M)

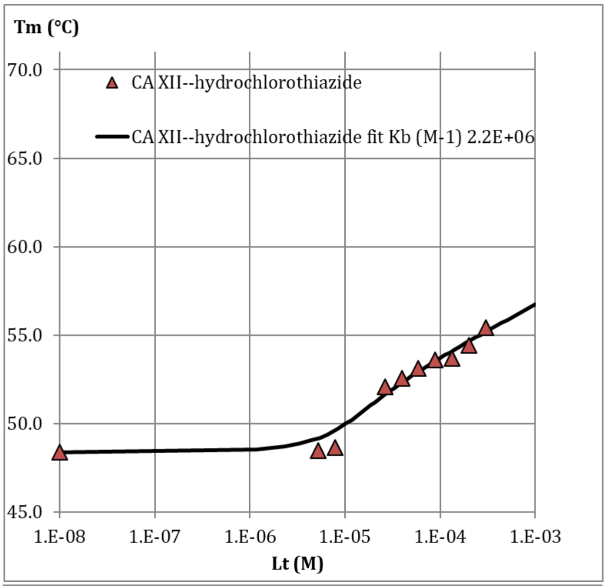
Tm (°C)
CA XII--hydrochlorothiazide
CA XII--hydrochlorothiazide fit Kb (M-1) 2.2E+06
70.0
65.0
60.0
55.0
50.0
45.0
1.E-08
1.E-07
1.E-06
1.E-05
1.E-04
1.E-03
Lt (M)

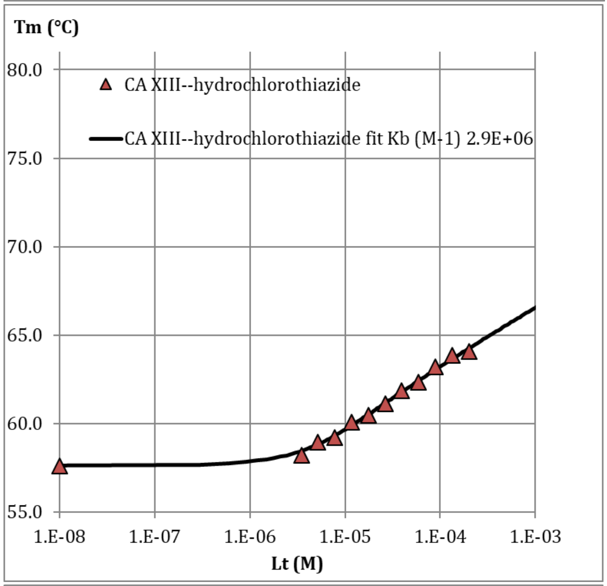
Tm (°C)
CA XIII--hydrochlorothiazide
CA XIII--hydrochlorothiazide fit Kb (M-1) 2.9E+06
80.0
75.0
70.0
65.0
60.0
55.0
1.E-08
1.E-07
1.E-06
1.E-05
1.E-04
1.E-03
Lt (M)

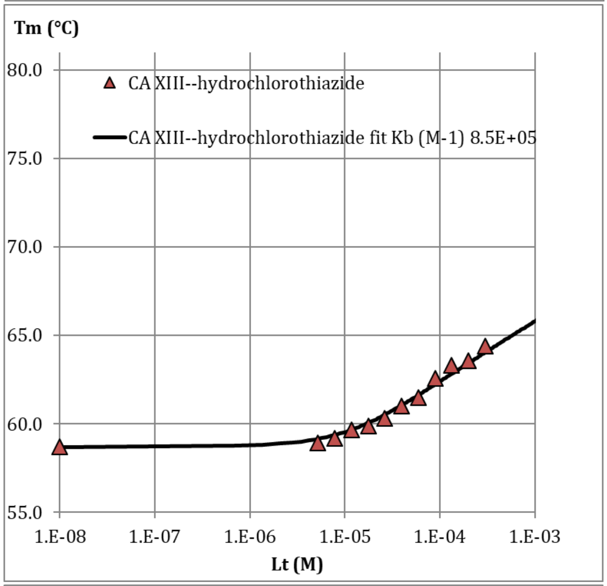
Tm (°C)
CA XIII--hydrochlorothiazide
CA XIII--hydrochlorothiazide fit Kb (M-1) 8.5E+05
80.0
75.0
70.0
65.0
60.0
55.0
1.E-08
1.E-07
1.E-06
1.E-05
1.E-04
1.E-03
Lt (M)

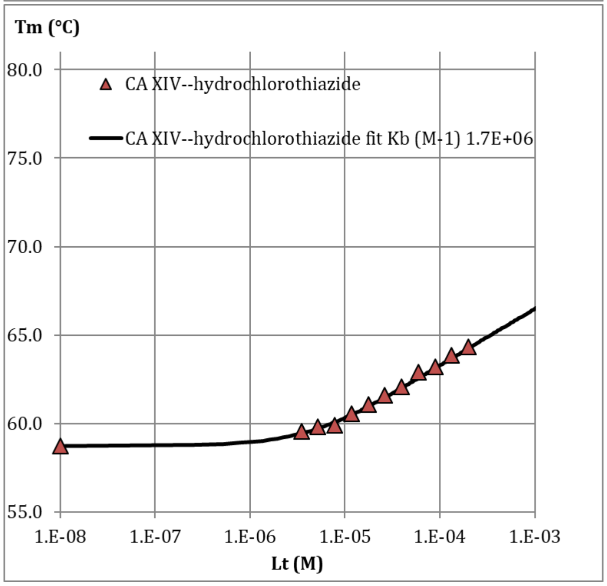
Tm (°C)
CA XIV--hydrochlorothiazide
CA XIV--hydrochlorothiazide fit Kb (M-1) 1.7E+06
80.0
75.0
70.0
65.0
60.0
55.0
1.E-08
1.E-07
1.E-06
1.E-05
1.E-04
1.E-03
Lt (M)

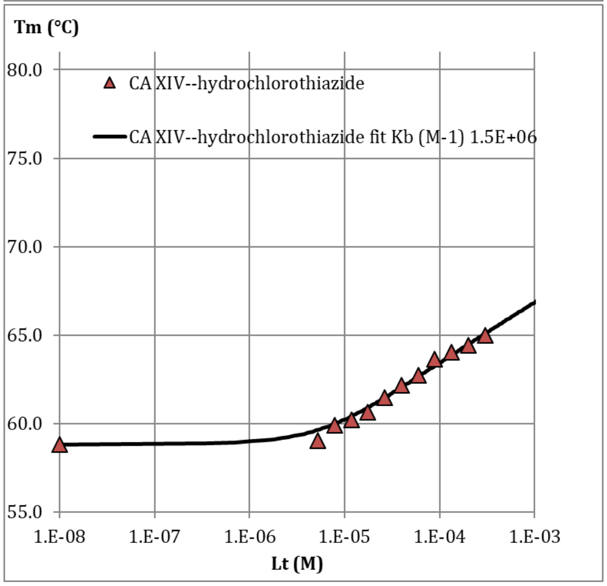
Tm (°C)
CA XIV--hydrochlorothiazide
CA XIV--hydrochlorothiazide fit Kb (M-1) 1.5E+06
80.0
75.0
70.0
65.0
60.0
55.0
1.E-08
1.E-07
1.E-06
1.E-05
1.E-04
1.E-03
Lt (M)

CO_2 hydration activity inhibition:

CA II- hydrochlorothiazide

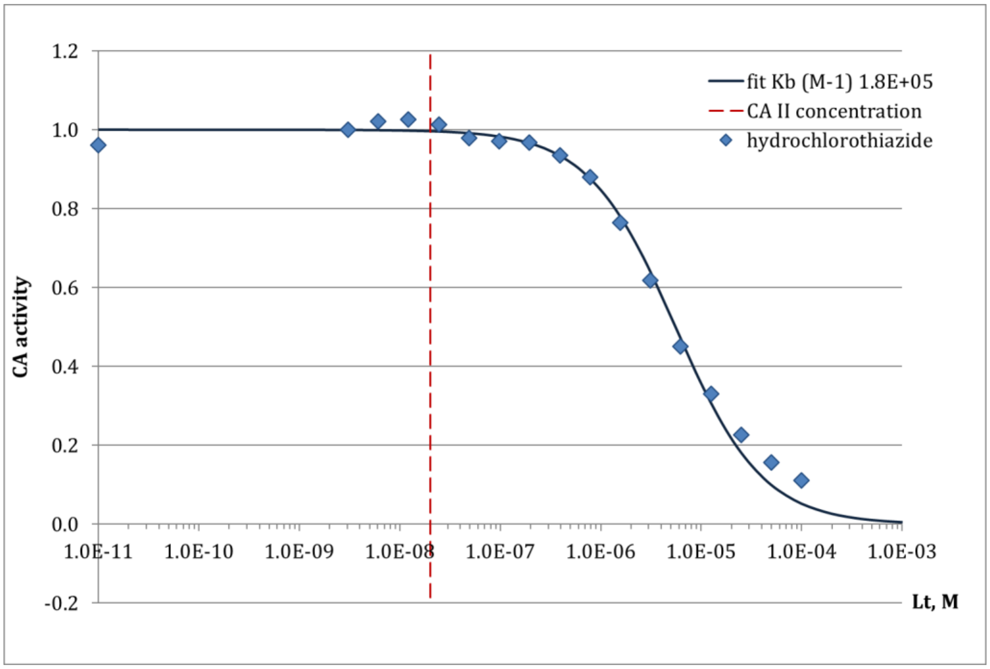

CA IV - hydrochlorothiazide

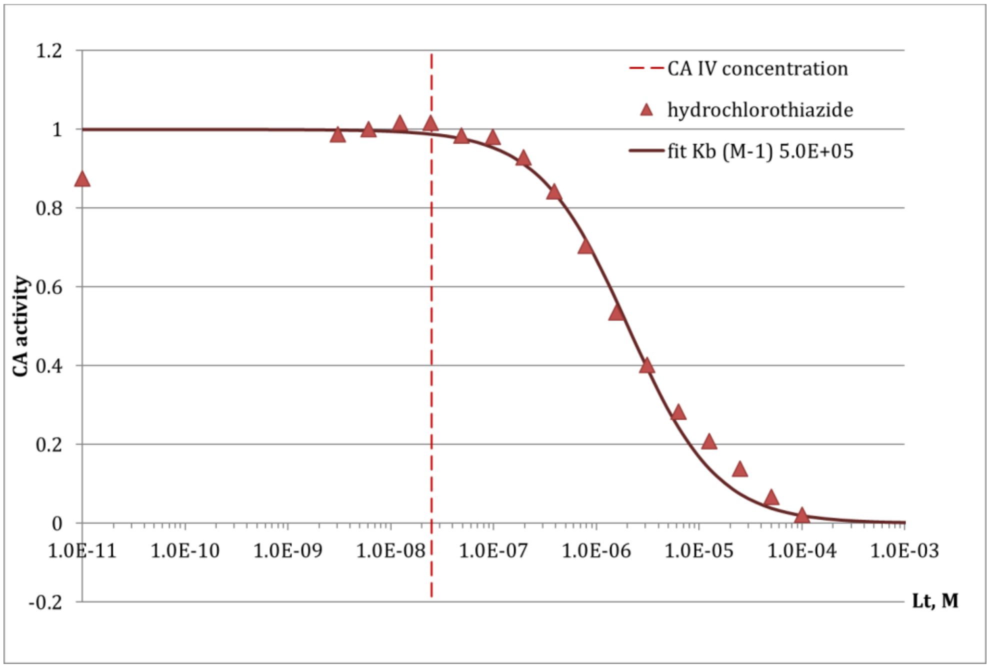

Isothermal titration calorimetry

CA II – hydrochlorothiazide

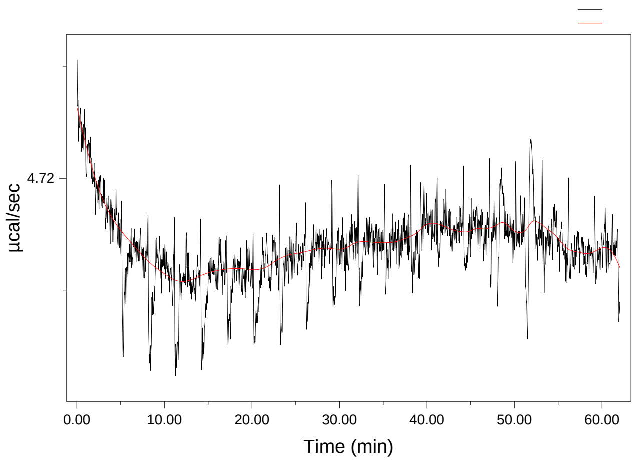

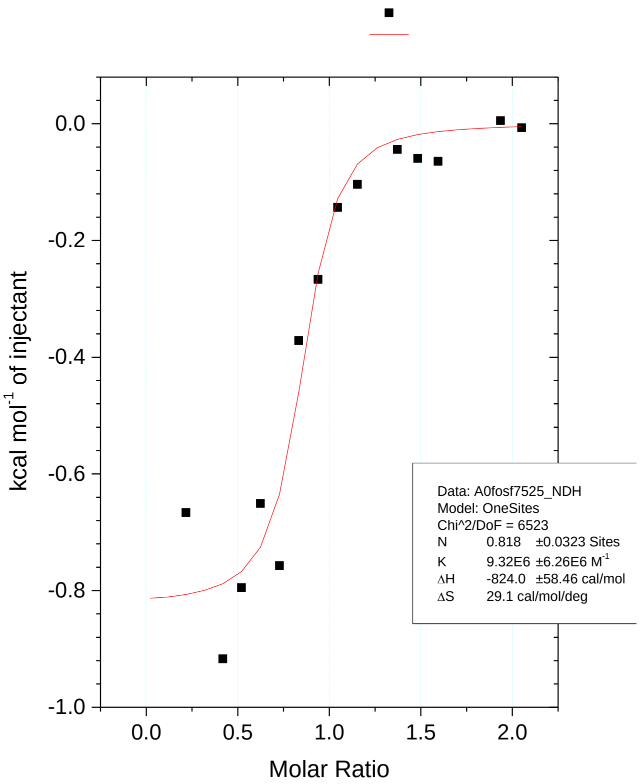

5. Trichloromethiazide

Fluorecence-based thermal shift assay

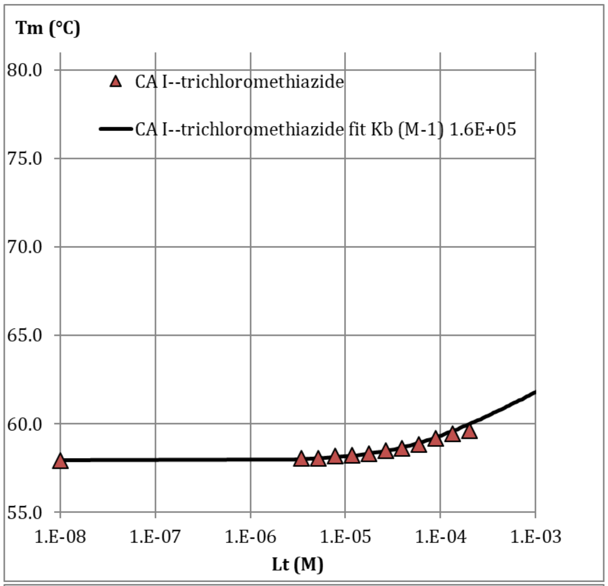

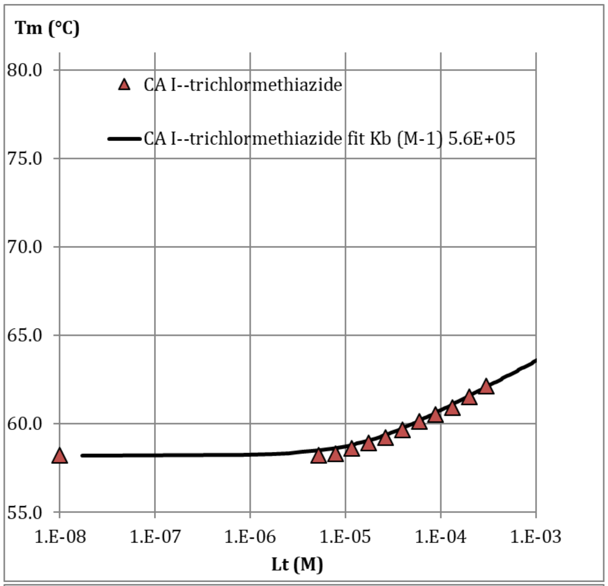

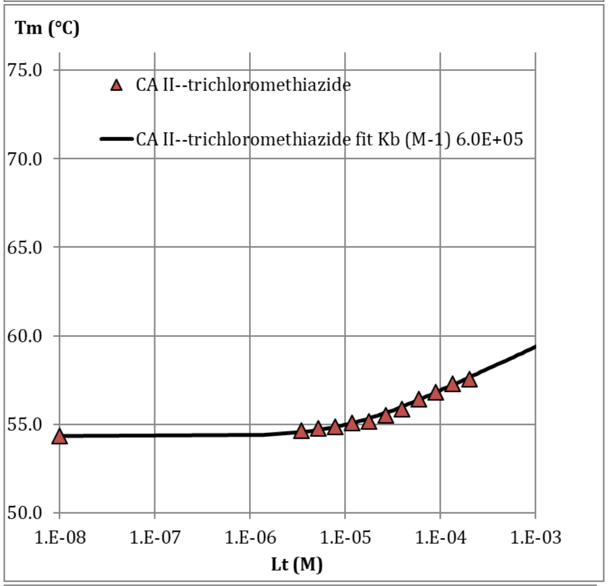

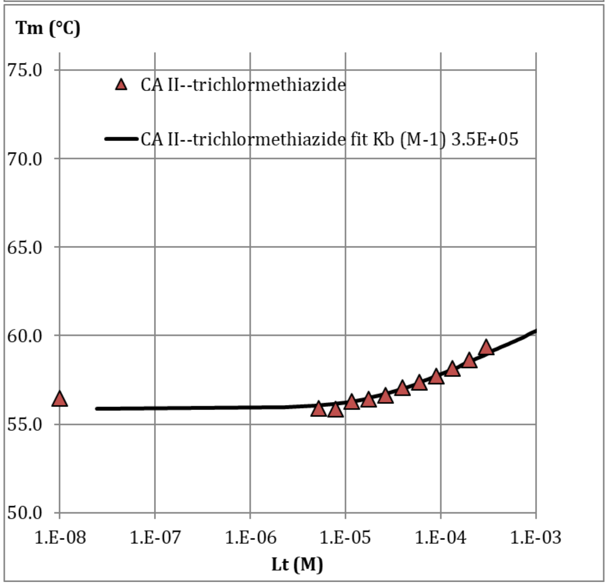

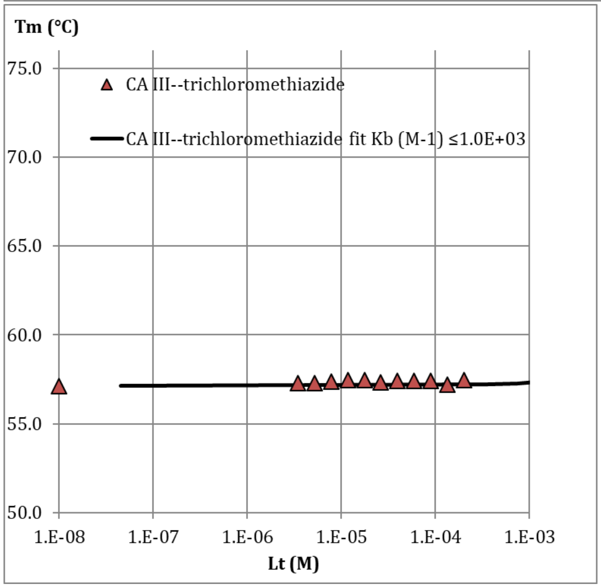

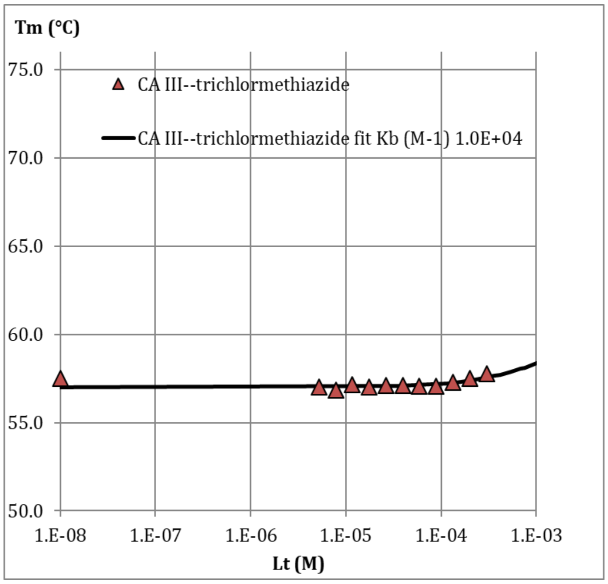

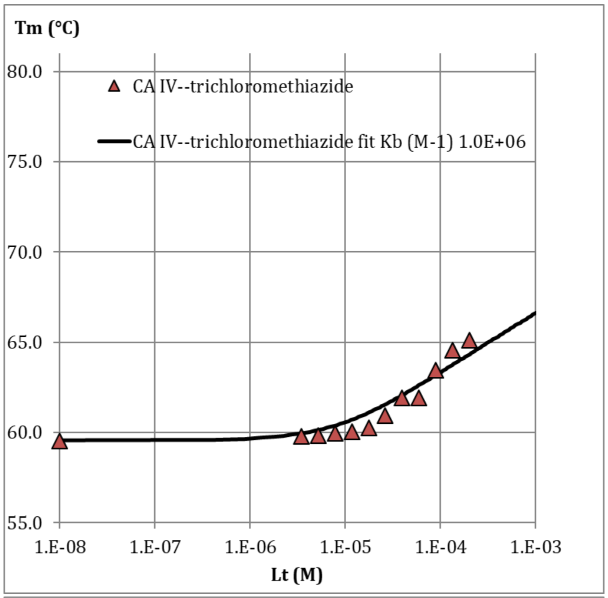
Tm (°C)
80.0
75.0
70.0
65.0
60.0
55.0
CA IV--trichloromethiazide
CA IV--trichloromethiazide fit Kb (M-1) 1.0E+06
1.E-08
1.E-07
1.E-06
1.E-05
1.E-04
1.E-03
Lt (M)

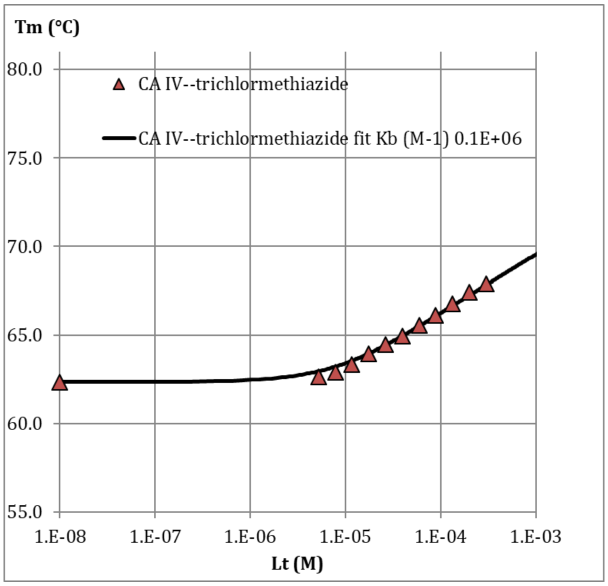
Tm (°C)
80.0
75.0
70.0
65.0
60.0
55.0
CA IV--trichlormethiazide
CA IV--trichlormethiazide fit Kb (M-1) 0.1E+06
1.E-08
1.E-07
1.E-06
1.E-05
1.E-04
1.E-03
Lt (M)

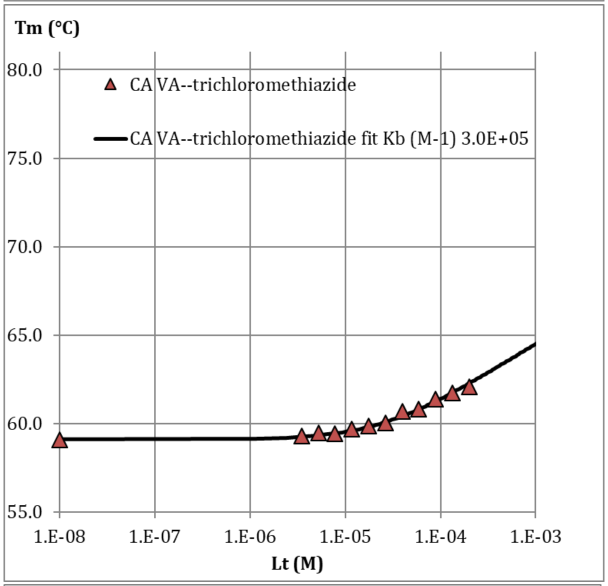
Tm (°C)
80.0
75.0
70.0
65.0
60.0
55.0
CA VA--trichloromethiazide
CA VA--trichloromethiazide fit Kb (M-1) 3.0E+05
1.E-08
1.E-07
1.E-06
1.E-05
1.E-04
1.E-03
Lt (M)

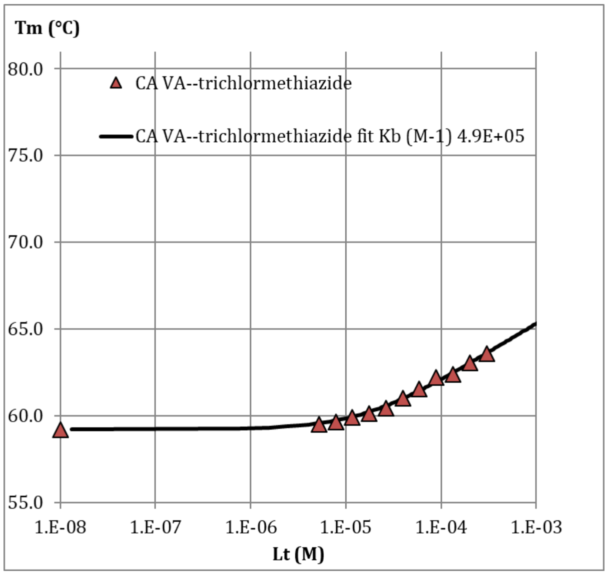
Tm (°C)
80.0
75.0
70.0
65.0
60.0
55.0
CA VA--trichlormethiazide
CA VA--trichlormethiazide fit Kb (M-1) 4.9E+05
1.E-08
1.E-07
1.E-06
1.E-05
1.E-04
1.E-03
Lt (M)

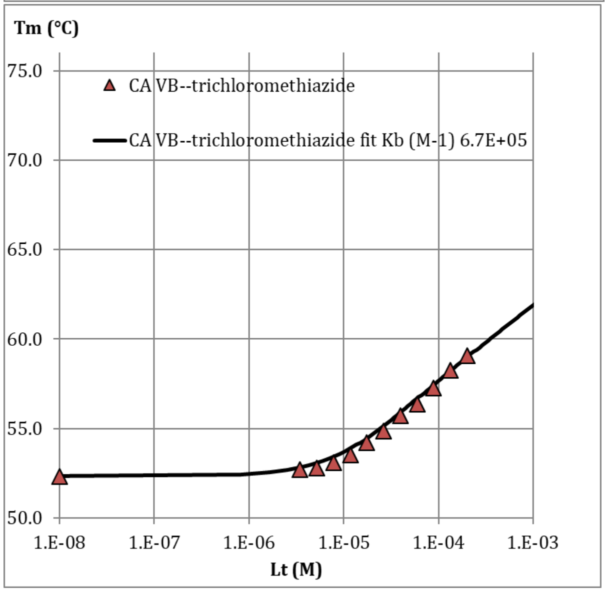
Tm (°C)
75.0
70.0
65.0
60.0
55.0
50.0
CA VB--trichloromethiazide
CA VB--trichloromethiazide fit Kb (M-1) 6.7E+05
1.E-08
1.E-07
1.E-06
1.E-05
1.E-04
1.E-03
Lt (M)

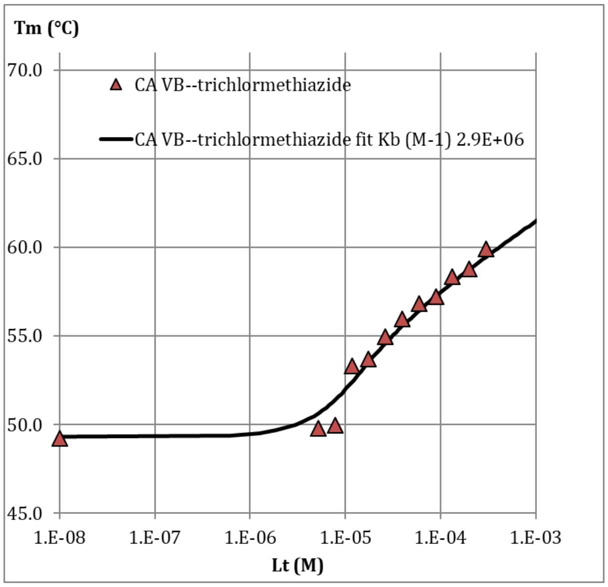
Tm (°C)
70.0
65.0
60.0
55.0
50.0
45.0
CA VB--trichlormethiazide
CA VB--trichlormethiazide fit Kb (M-1) 2.9E+06
1.E-08
1.E-07
1.E-06
1.E-05
1.E-04
1.E-03
Lt (M)

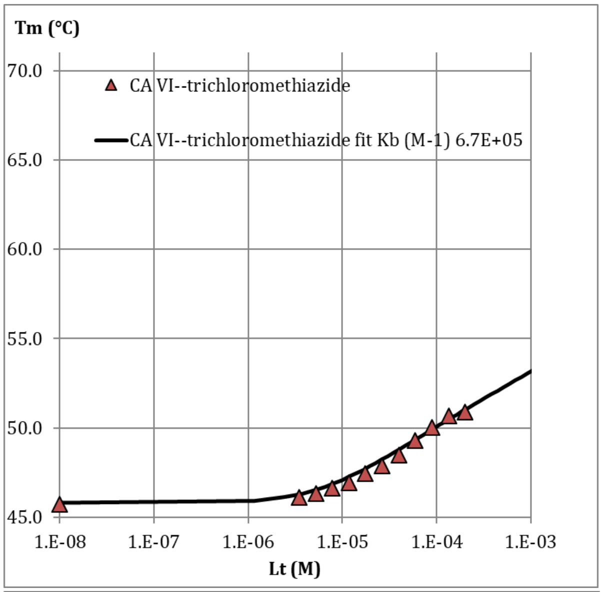
Tm (°C)
70.0
65.0
60.0
55.0
50.0
45.0
CA VI--trichloromethiazide
CA VI--trichloromethiazide fit Kb (M-1) 6.7E+05
1.E-08
1.E-07
1.E-06
1.E-05
1.E-04
1.E-03
Lt (M)

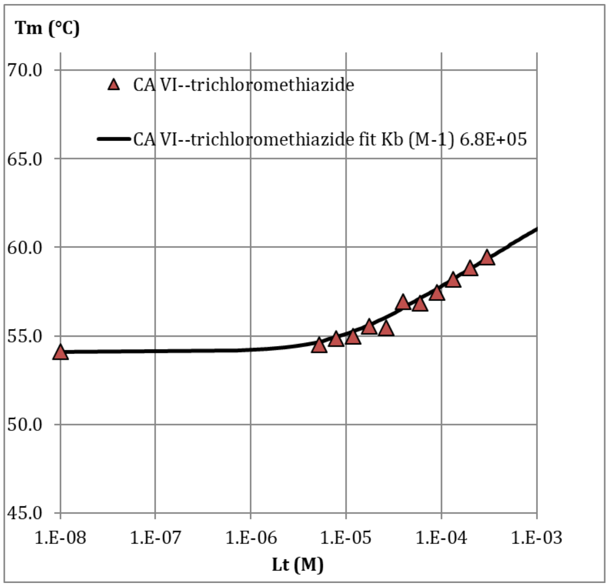
Tm (°C)
70.0
65.0
60.0
55.0
50.0
45.0
CA VI--trichloromethiazide
CA VI--trichloromethiazide fit Kb (M-1) 6.8E+05
1.E-08
1.E-07
1.E-06
1.E-05
1.E-04
1.E-03
Lt (M)

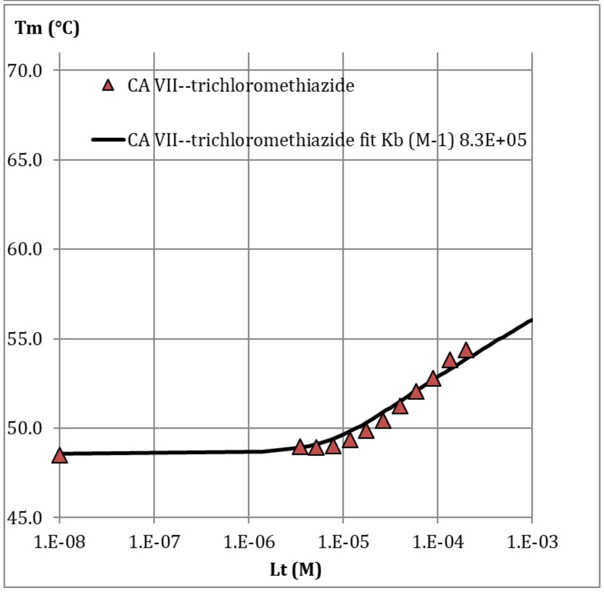
Tm (°C)
70.0
65.0
60.0
55.0
50.0
45.0
CA VII--trichloromethiazide
CA VII--trichloromethiazide fit Kb (M-1) 8.3E+05
1.E-08
1.E-07
1.E-06
1.E-05
1.E-04
1.E-03
Lt (M)

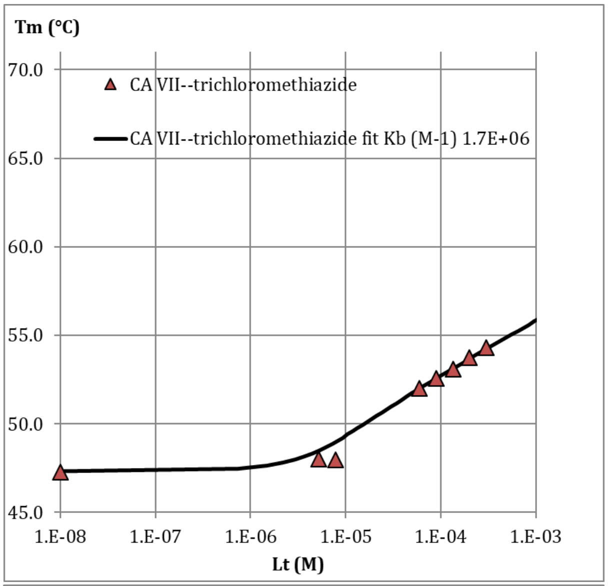
Tm (°C)
70.0
65.0
60.0
55.0
50.0
45.0
CA VII--trichloromethiazide
CA VII--trichloromethiazide fit Kb (M-1) 1.7E+06
1.E-08
1.E-07
1.E-06
1.E-05
1.E-04
1.E-03
Lt (M)

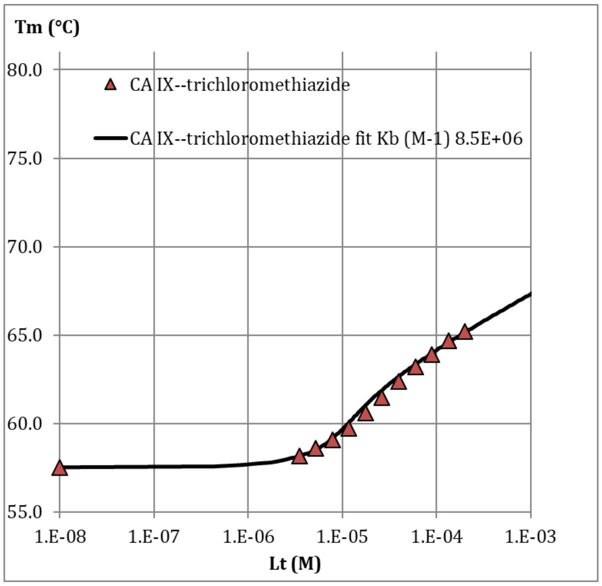
Tm (°C)
80.0
75.0
70.0
65.0
60.0
55.0
CA IX--trichloromethiazide
CA IX--trichloromethiazide fit Kb (M-1) 8.5E+06
1.E-08
1.E-07
1.E-06
1.E-05
1.E-04
1.E-03
Lt (M)

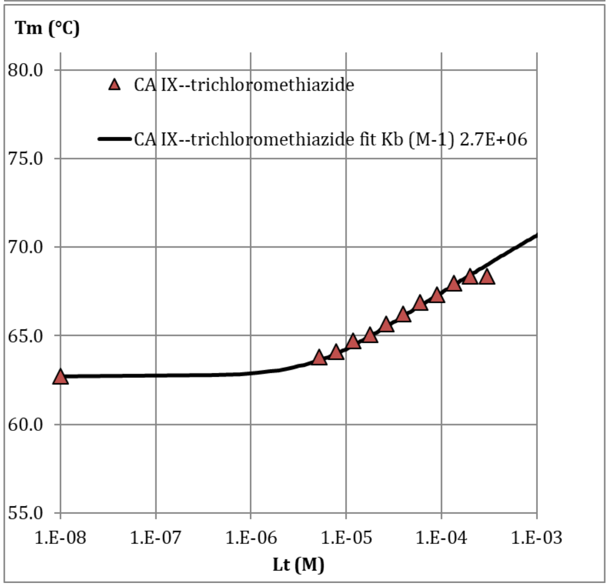
Tm (°C)
80.0
75.0
70.0
65.0
60.0
55.0
CA IX--trichloromethiazide
CA IX--trichloromethiazide fit Kb (M-1) 2.7E+06
1.E-08
1.E-07
1.E-06
1.E-05
1.E-04
1.E-03
Lt (M)

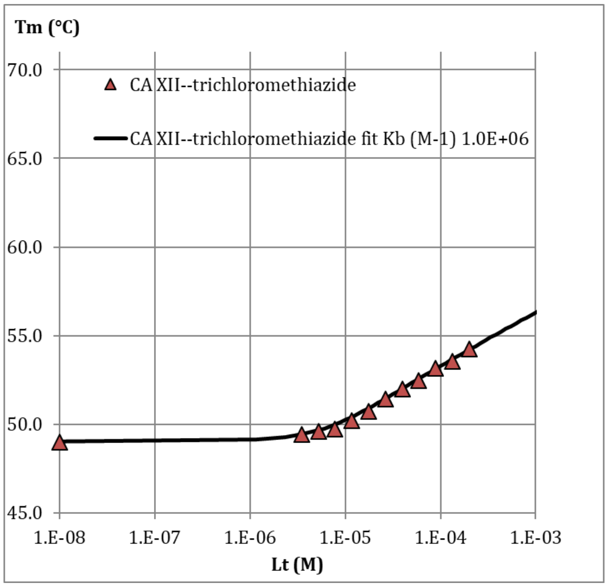
Tm (°C)
CA XII--trichloromethiazide
CA XII--trichloromethiazide fit Kb (M-1) 1.0E+06
Lt (M)

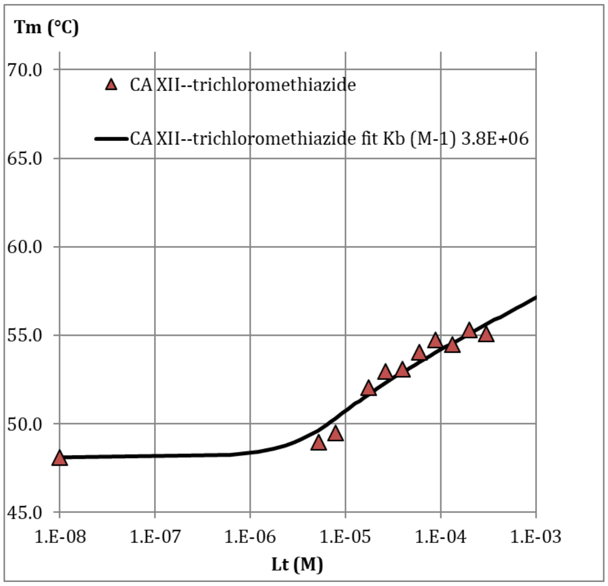
Tm (°C)
CA XII--trichloromethiazide
CA XII--trichloromethiazide fit Kb (M-1) 3.8E+06
Lt (M)

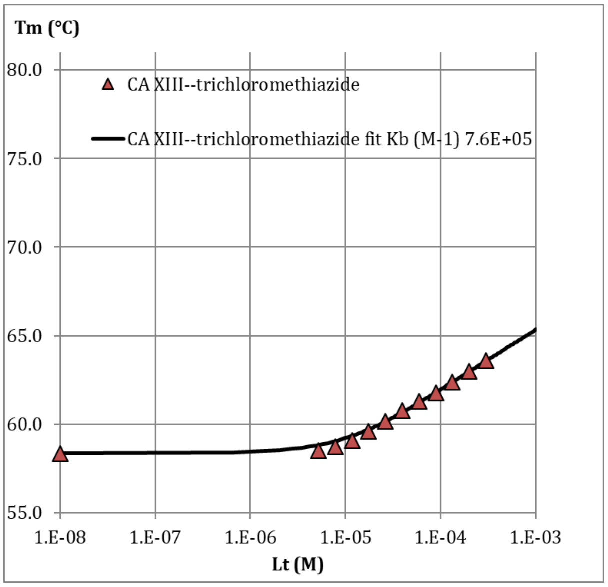
Tm (°C)
CA XIII--trichloromethiazide
CA XIII--trichloromethiazide fit Kb (M-1) 7.6E+05
Lt (M)

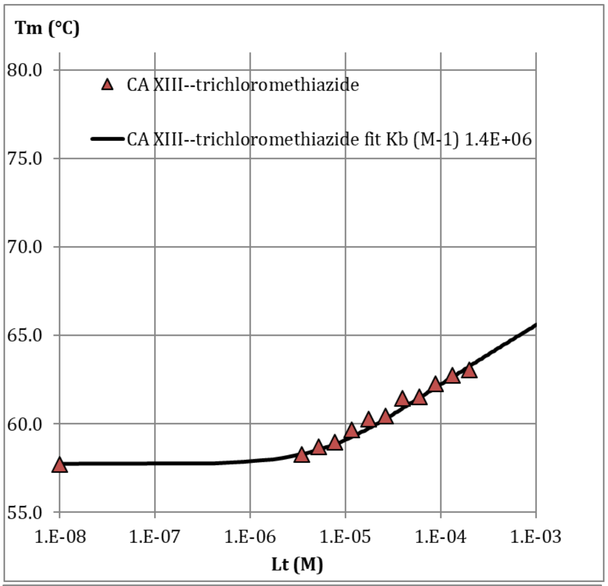
Tm (°C)
CA XIII--trichloromethiazide
CA XIII--trichloromethiazide fit Kb (M-1) 1.4E+06
Lt (M)

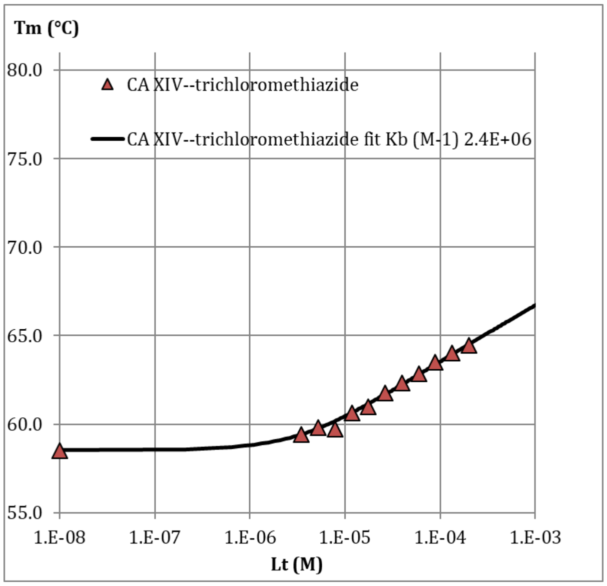
Tm (°C)
CA XIV--trichloromethiazide
CA XIV--trichloromethiazide fit Kb (M-1) 2.4E+06
Lt (M)

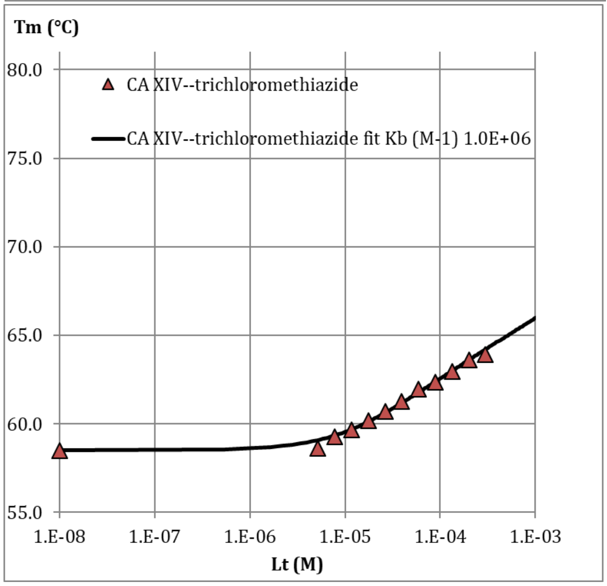
Tm (°C)
CA XIV--trichloromethiazide
CA XIV--trichloromethiazide fit Kb (M-1) 1.0E+06
Lt (M)

6. Cyclothiazide

Fluorecence-based thermal shift assay

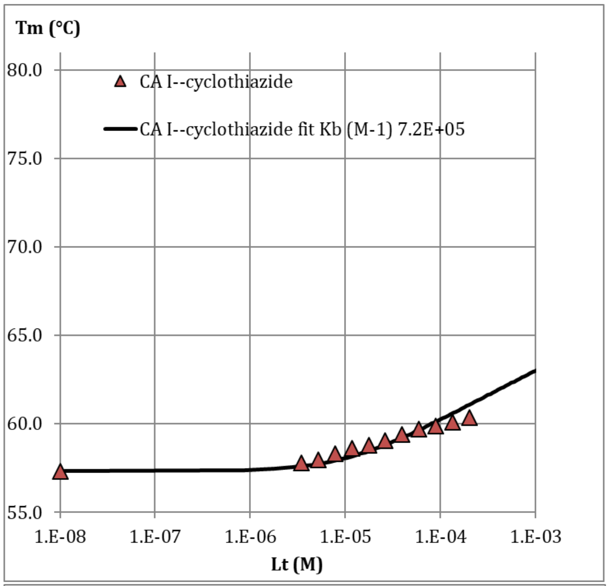

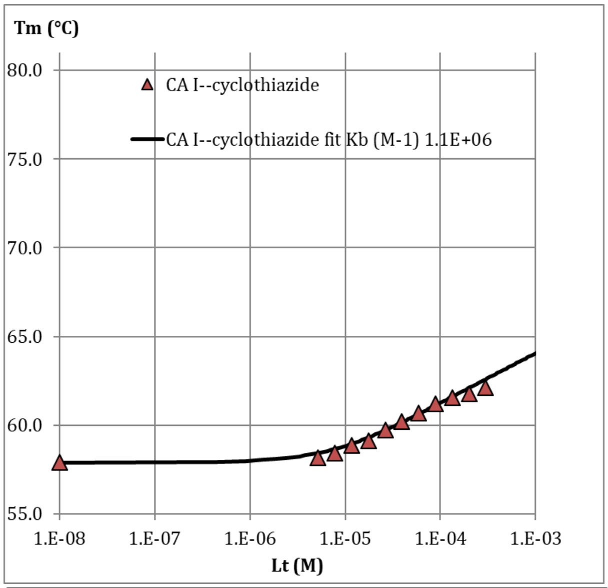

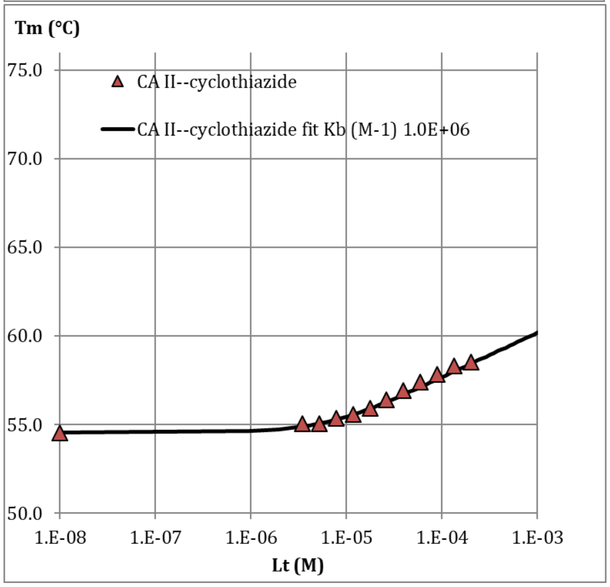

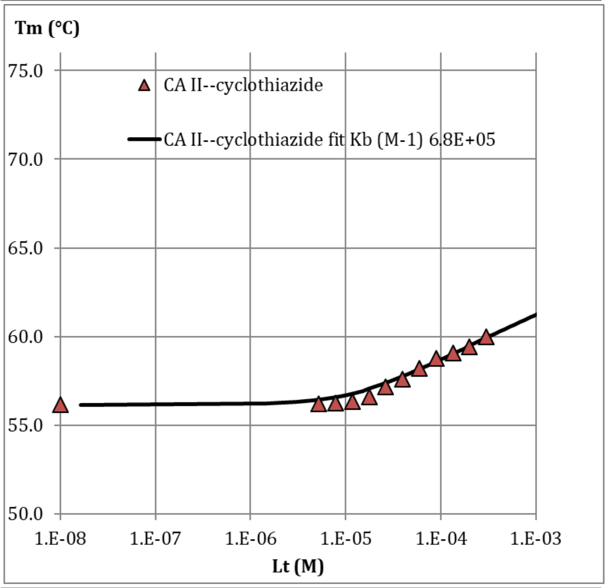

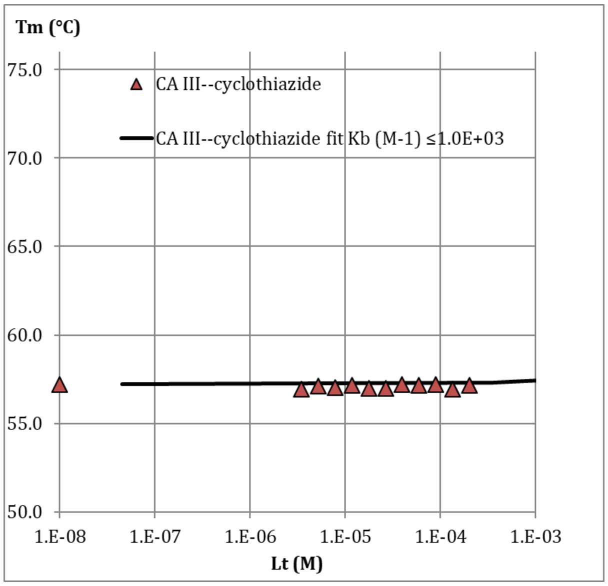

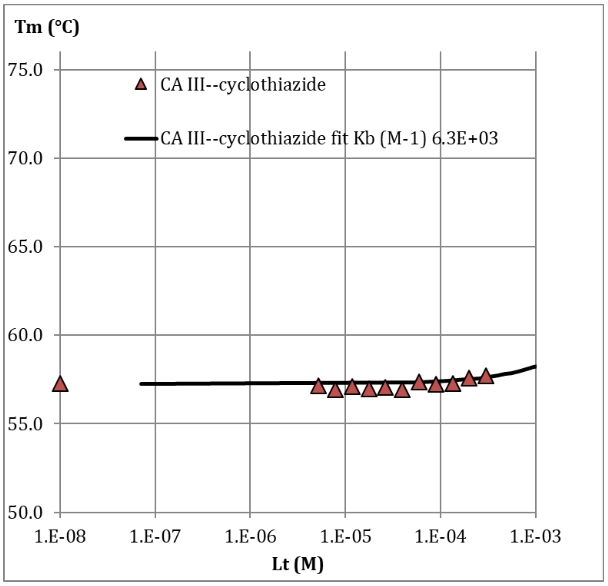

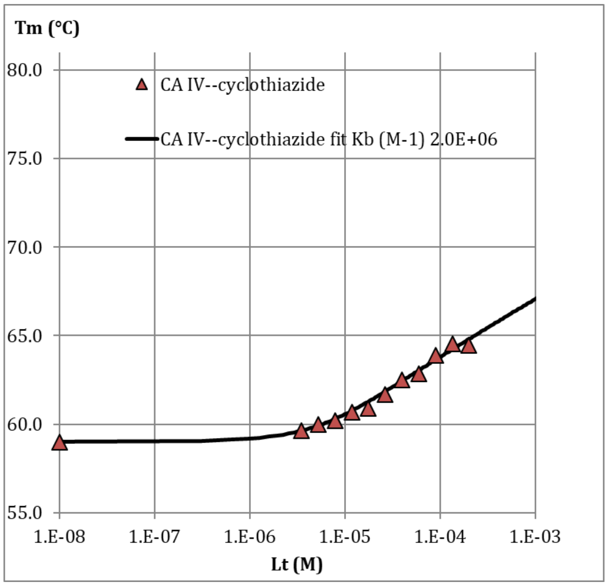
Tm (°C)
CA IV--cyclothiazide
CA IV--cyclothiazide fit Kb (M-1) 2.0E+06
Lt (M)

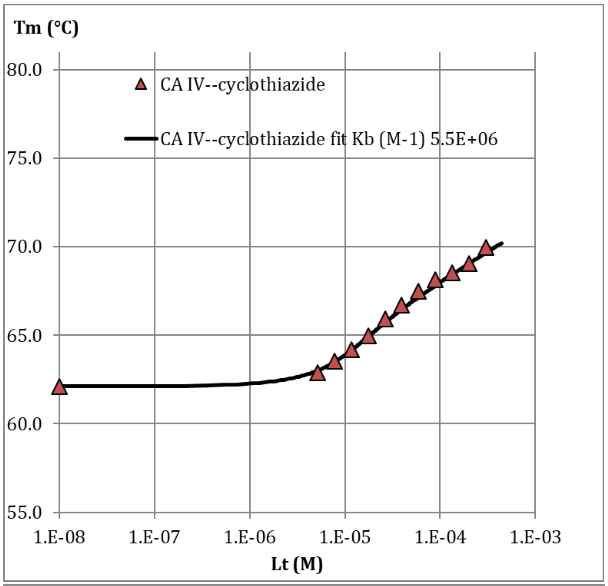
Tm (°C)
CA IV--cyclothiazide
CA IV--cyclothiazide fit Kb (M-1) 5.5E+06
Lt (M)

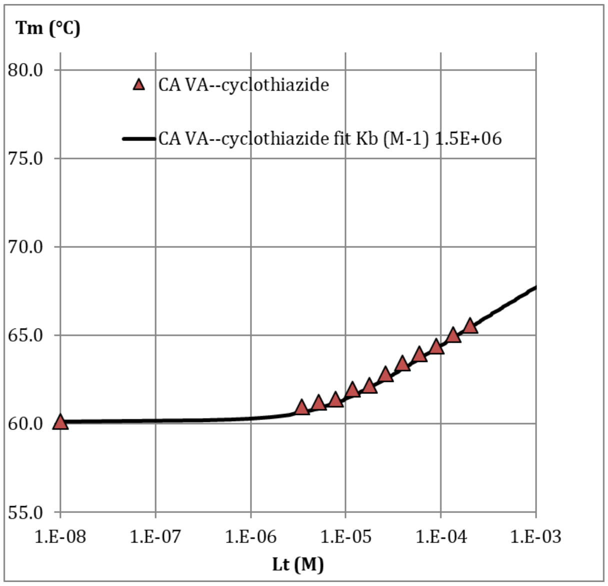
Tm (°C)
CA VA--cyclothiazide
CA VA--cyclothiazide fit Kb (M-1) 1.5E+06
Lt (M)

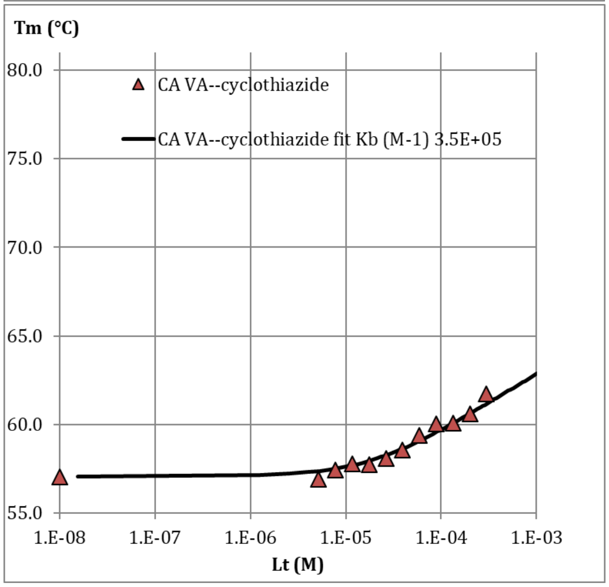
Tm (°C)
CA VA--cyclothiazide
CA VA--cyclothiazide fit Kb (M-1) 3.5E+05
Lt (M)

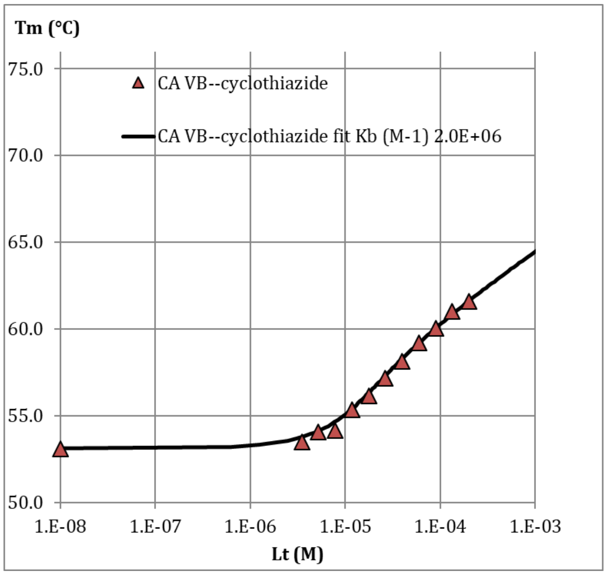
Tm (°C)
CA VB--cyclothiazide
CA VB--cyclothiazide fit Kb (M-1) 2.0E+06
Lt (M)

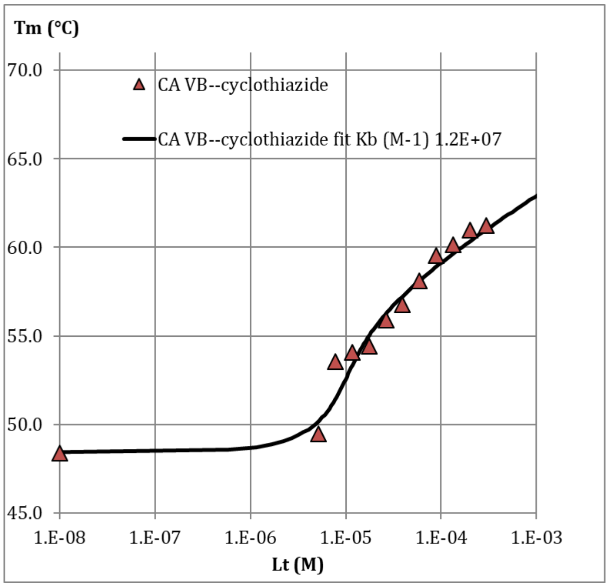
Tm (°C)
CA VB--cyclothiazide
CA VB--cyclothiazide fit Kb (M-1) 1.2E+07
Lt (M)

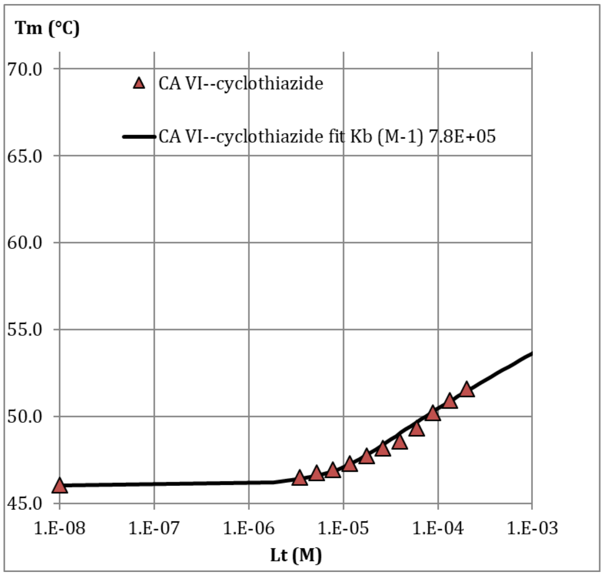
Tm (°C)
70.0
65.0
60.0
55.0
50.0
45.0
CA VI--cyclothiazide
CA VI--cyclothiazide fit Kb (M-1) 7.8E+05
1.E-08
1.E-07
1.E-06
1.E-05
1.E-04
1.E-03
Lt (M)

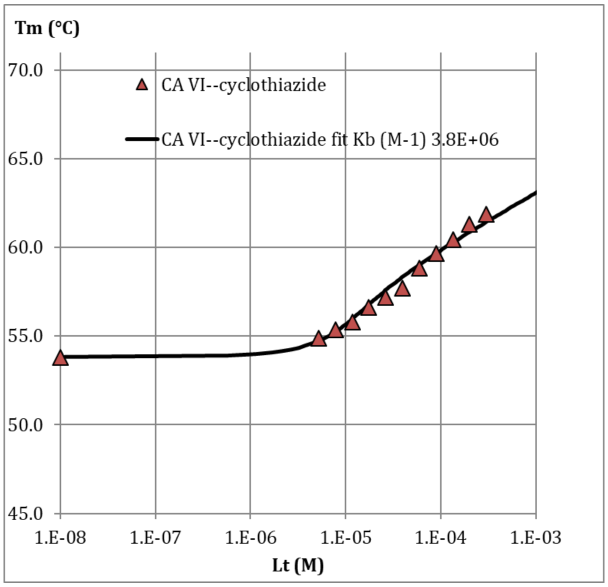
Tm (°C)
70.0
65.0
60.0
55.0
50.0
45.0
CA VI--cyclothiazide
CA VI--cyclothiazide fit Kb (M-1) 3.8E+06
1.E-08
1.E-07
1.E-06
1.E-05
1.E-04
1.E-03
Lt (M)

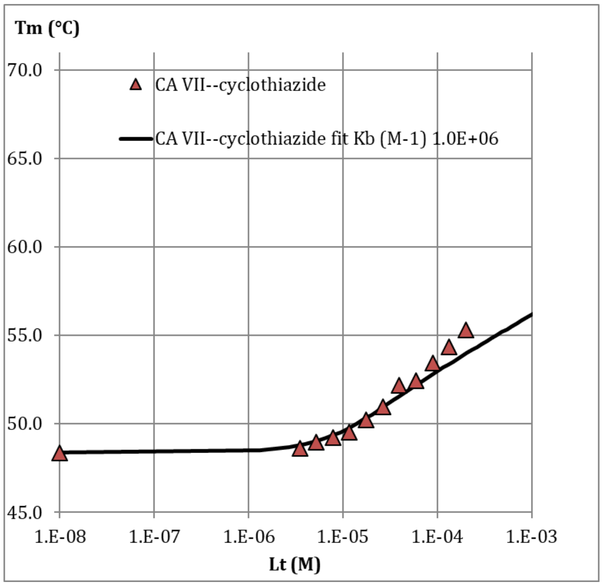
Tm (°C)
70.0
65.0
60.0
55.0
50.0
45.0
CA VII--cyclothiazide
CA VII--cyclothiazide fit Kb (M-1) 1.0E+06
1.E-08
1.E-07
1.E-06
1.E-05
1.E-04
1.E-03
Lt (M)

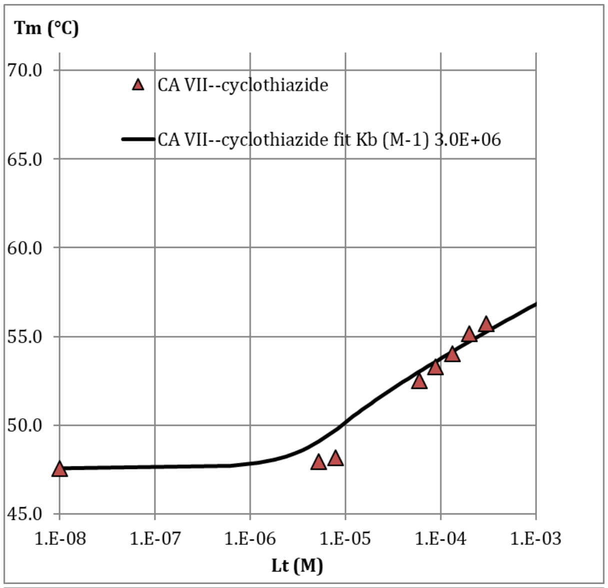
Tm (°C)
70.0
65.0
60.0
55.0
50.0
45.0
CA VII--cyclothiazide
CA VII--cyclothiazide fit Kb (M-1) 3.0E+06
1.E-08
1.E-07
1.E-06
1.E-05
1.E-04
1.E-03
Lt (M)

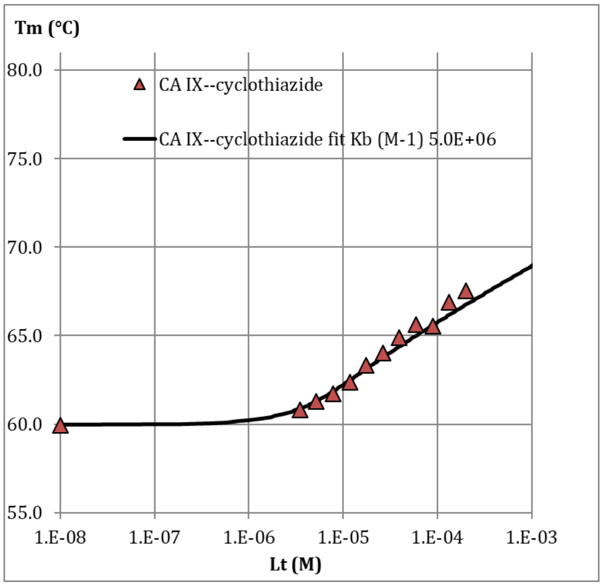
Tm (°C)
80.0
75.0
70.0
65.0
60.0
55.0
CA IX--cyclothiazide
CA IX--cyclothiazide fit Kb (M-1) 5.0E+06
1.E-08
1.E-07
1.E-06
1.E-05
1.E-04
1.E-03
Lt (M)

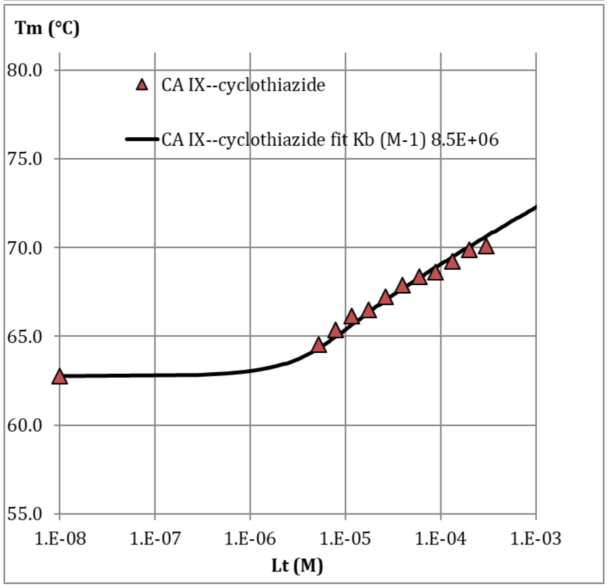
Tm (°C)
80.0
75.0
70.0
65.0
60.0
55.0
CA IX--cyclothiazide
CA IX--cyclothiazide fit Kb (M-1) 8.5E+06
1.E-08
1.E-07
1.E-06
1.E-05
1.E-04
1.E-03
Lt (M)

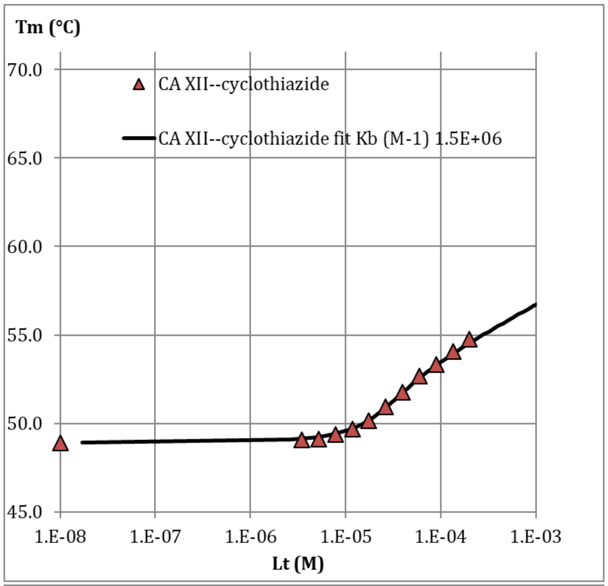
Tm (°C)
CA XII--cyclothiazide
CA XII--cyclothiazide fit Kb (M-1) 1.5E+06
Lt (M)

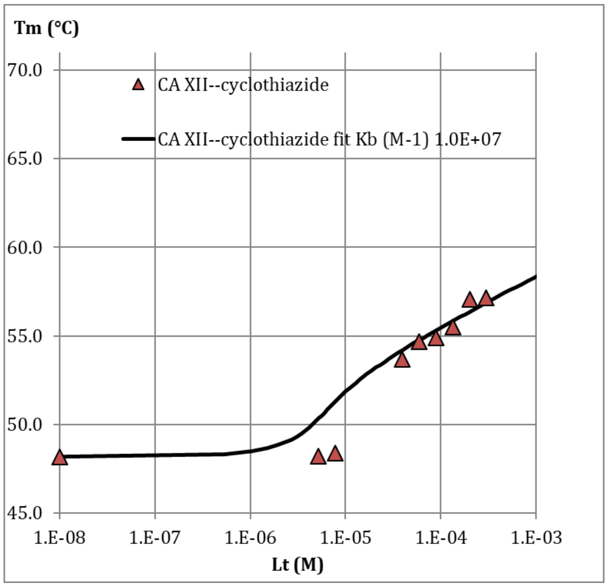
Tm (°C)
CA XII--cyclothiazide
CA XII--cyclothiazide fit Kb (M-1) 1.0E+07
Lt (M)

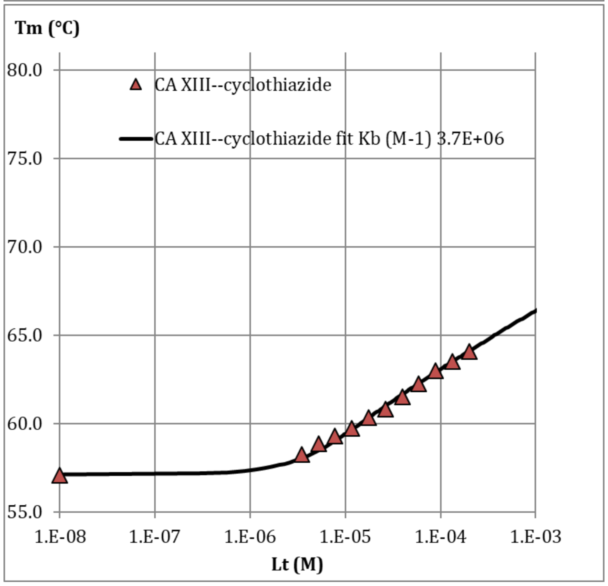
Tm (°C)
CA XIII--cyclothiazide
CA XIII--cyclothiazide fit Kb (M-1) 3.7E+06
Lt (M)

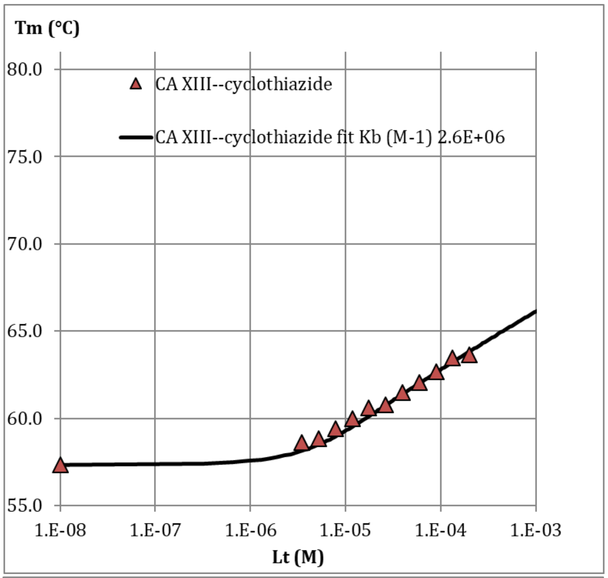
Tm (°C)
CA XIII--cyclothiazide
CA XIII--cyclothiazide fit Kb (M-1) 2.6E+06
Lt (M)

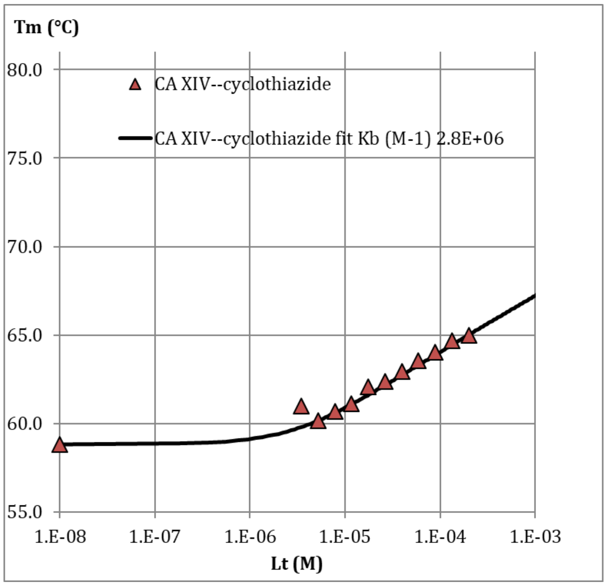
Tm (°C)
CA XIV--cyclothiazide
CA XIV--cyclothiazide fit Kb (M-1) 2.8E+06
Lt (M)

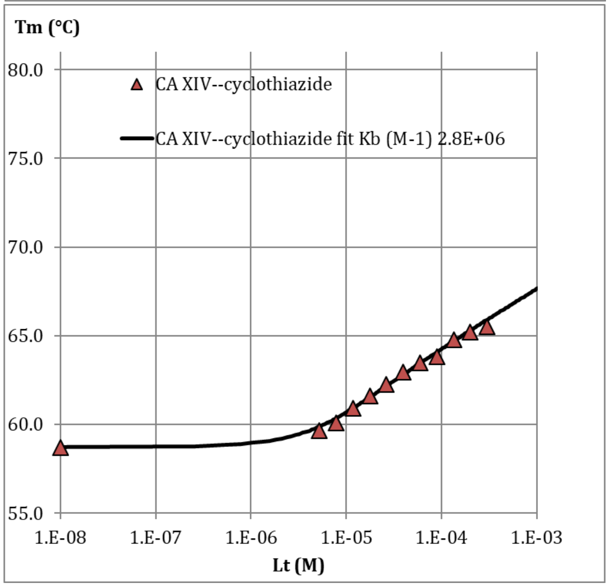
Tm (°C)
CA XIV--cyclothiazide
CA XIV--cyclothiazide fit Kb (M-1) 2.8E+06
Lt (M)

7. Polythiazide

Fluorecence-based thermal shift assay

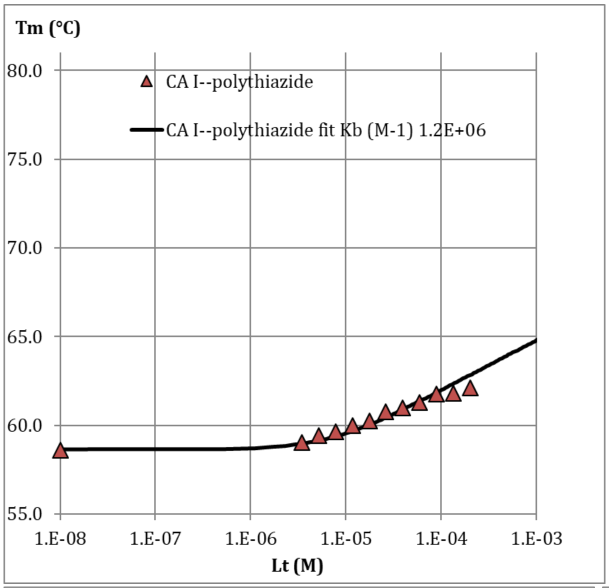

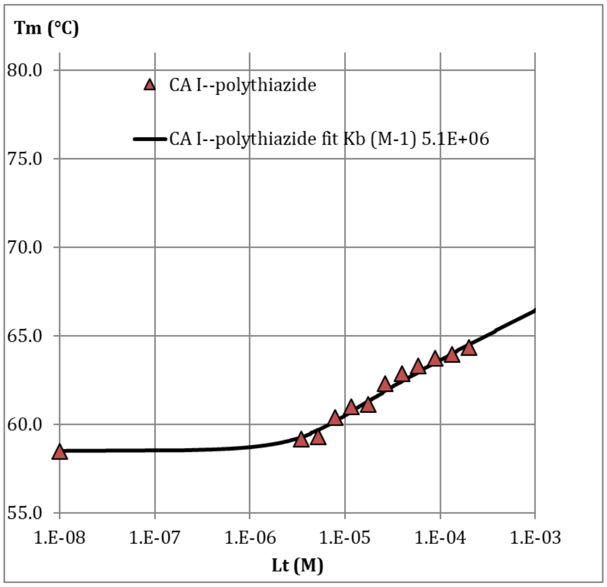

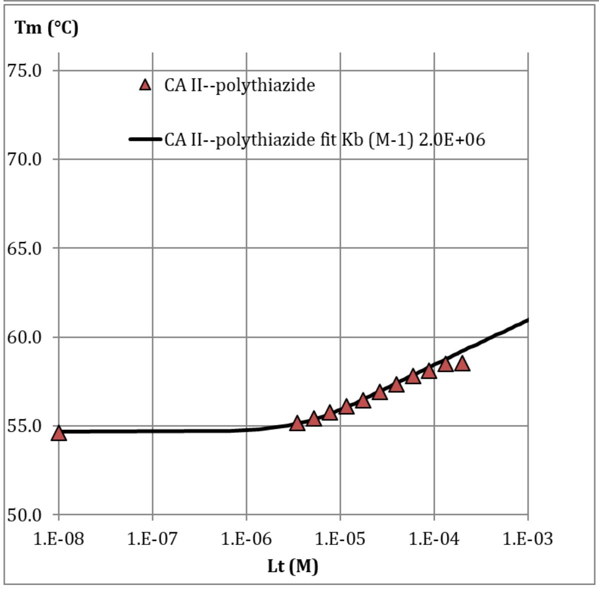

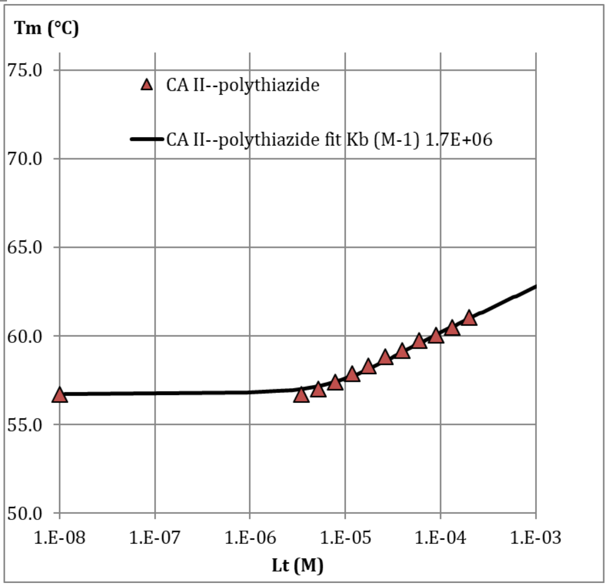

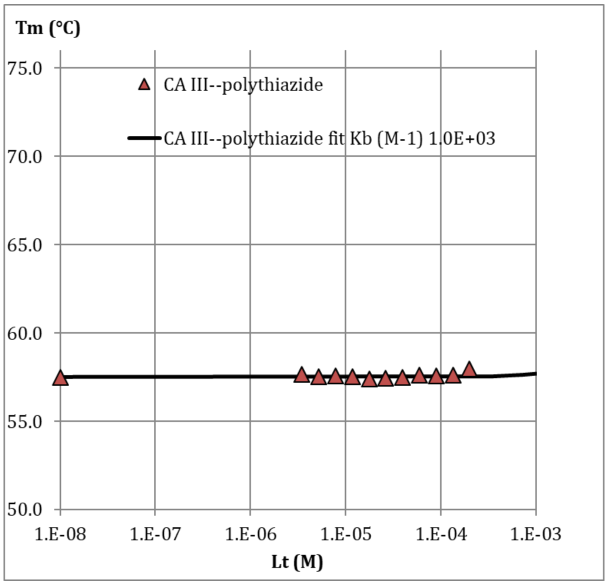

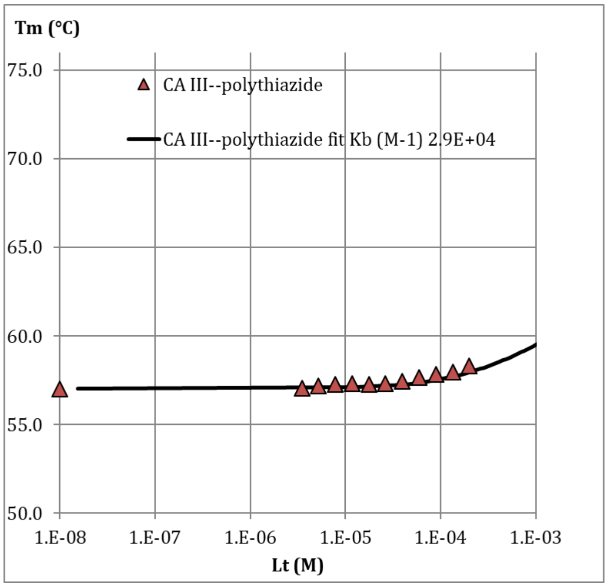

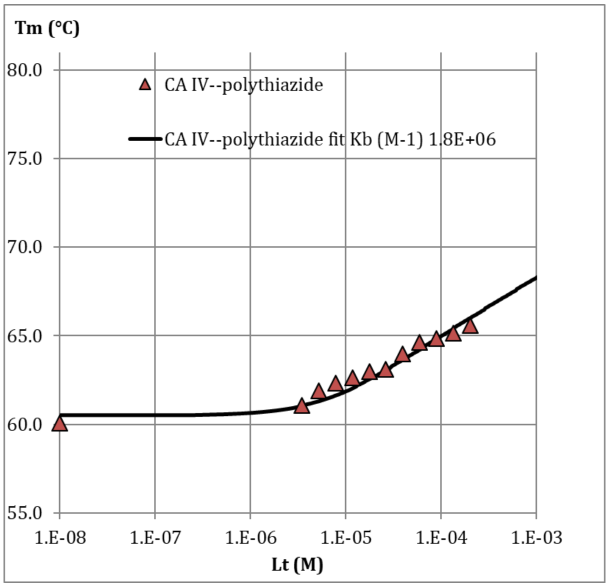
Tm (°C)
80.0
75.0
70.0
65.0
60.0
55.0
CA IV--polythiazide
CA IV--polythiazide fit Kb (M-1) 1.8E+06
1.E-08
1.E-07
1.E-06
1.E-05
1.E-04
1.E-03
Lt (M)

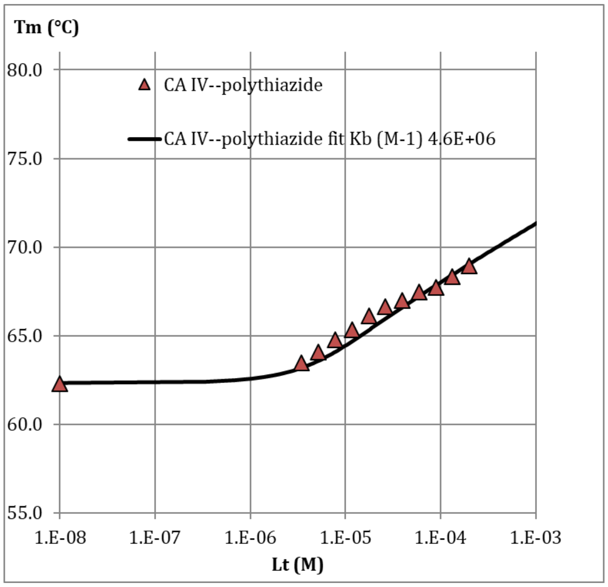
Tm (°C)
80.0
75.0
70.0
65.0
60.0
55.0
CA IV--polythiazide
CA IV--polythiazide fit Kb (M-1) 4.6E+06
1.E-08
1.E-07
1.E-06
1.E-05
1.E-04
1.E-03
Lt (M)

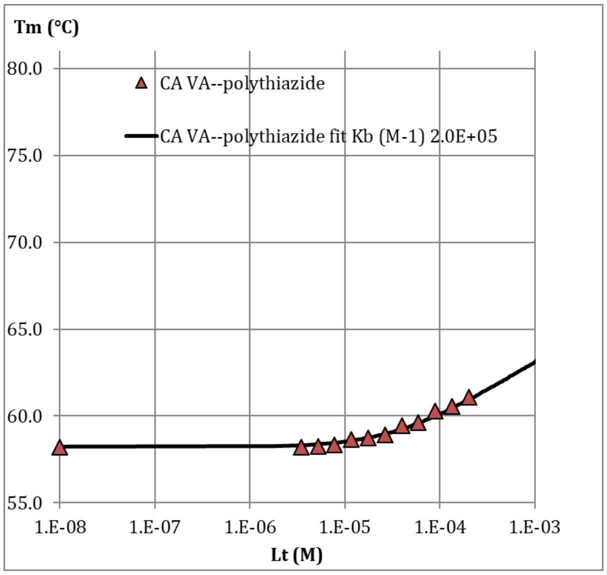
Tm (°C)
80.0
75.0
70.0
65.0
60.0
55.0
CA VA--polythiazide
CA VA--polythiazide fit Kb (M-1) 2.0E+05
1.E-08
1.E-07
1.E-06
1.E-05
1.E-04
1.E-03
Lt (M)

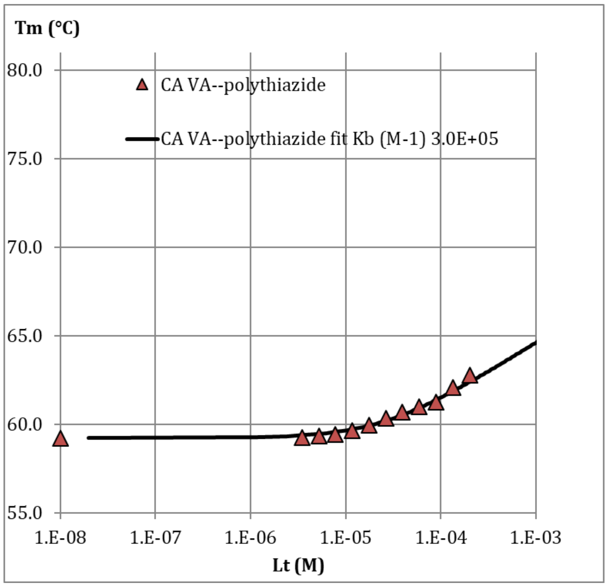
Tm (°C)
80.0
75.0
70.0
65.0
60.0
55.0
CA VA--polythiazide
CA VA--polythiazide fit Kb (M-1) 3.0E+05
1.E-08
1.E-07
1.E-06
1.E-05
1.E-04
1.E-03
Lt (M)

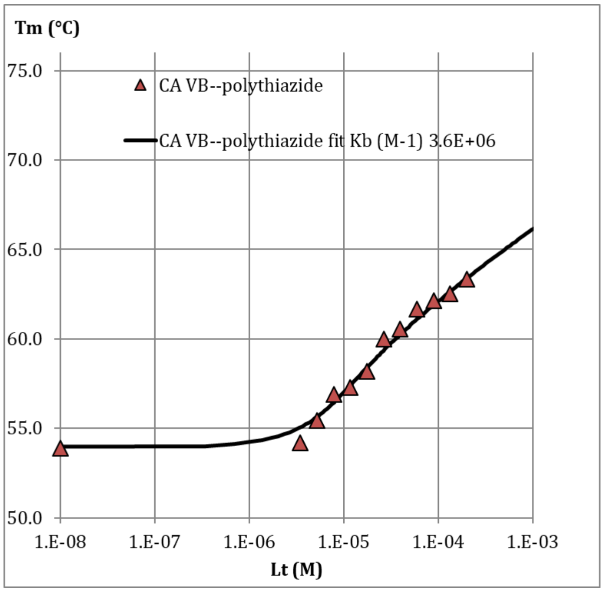
Tm (°C)
75.0
70.0
65.0
60.0
55.0
50.0
CA VB--polythiazide
CA VB--polythiazide fit Kb (M-1) 3.6E+06
1.E-08
1.E-07
1.E-06
1.E-05
1.E-04
1.E-03
Lt (M)

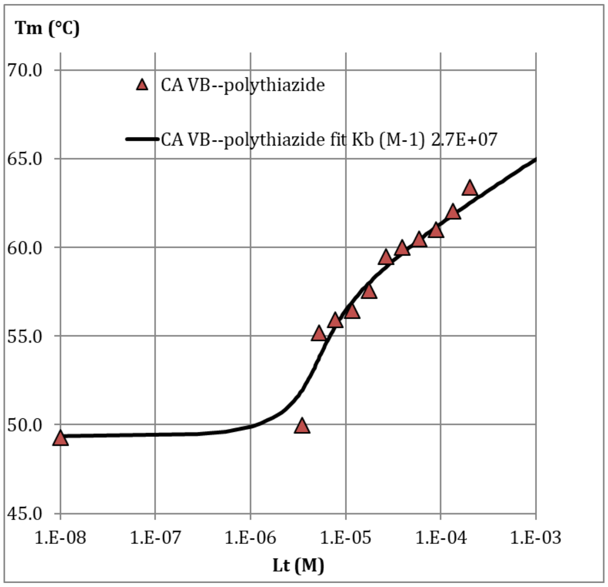
Tm (°C)
70.0
65.0
60.0
55.0
50.0
45.0
CA VB--polythiazide
CA VB--polythiazide fit Kb (M-1) 2.7E+07
1.E-08
1.E-07
1.E-06
1.E-05
1.E-04
1.E-03
Lt (M)

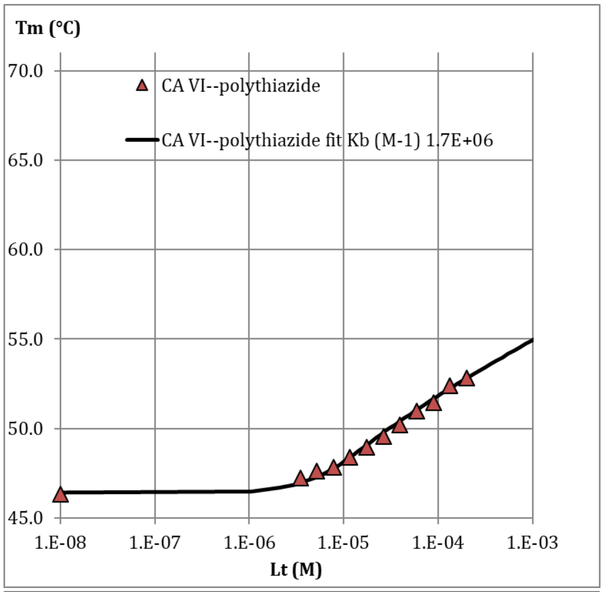
Tm (°C)
CA VI--polythiazide
CA VI--polythiazide fit Kb (M-1) 1.7E+06
70.0
65.0
60.0
55.0
50.0
45.0
1.E-08
1.E-07
1.E-06
1.E-05
1.E-04
1.E-03
Lt (M)

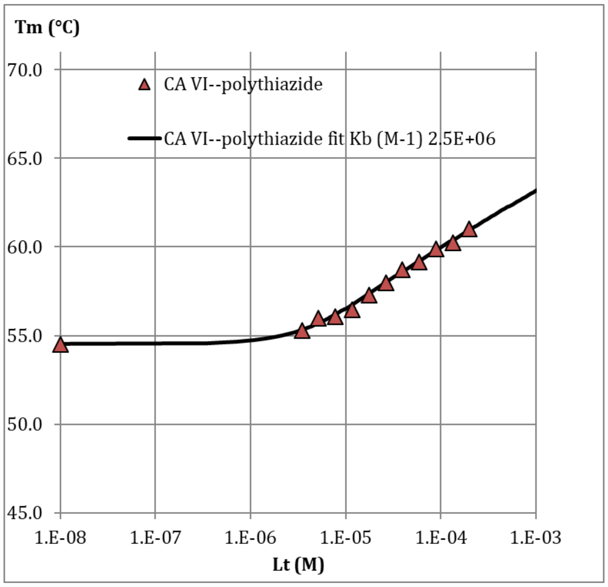
Tm (°C)
CA VI--polythiazide
CA VI--polythiazide fit Kb (M-1) 2.5E+06
70.0
65.0
60.0
55.0
50.0
45.0
1.E-08
1.E-07
1.E-06
1.E-05
1.E-04
1.E-03
Lt (M)

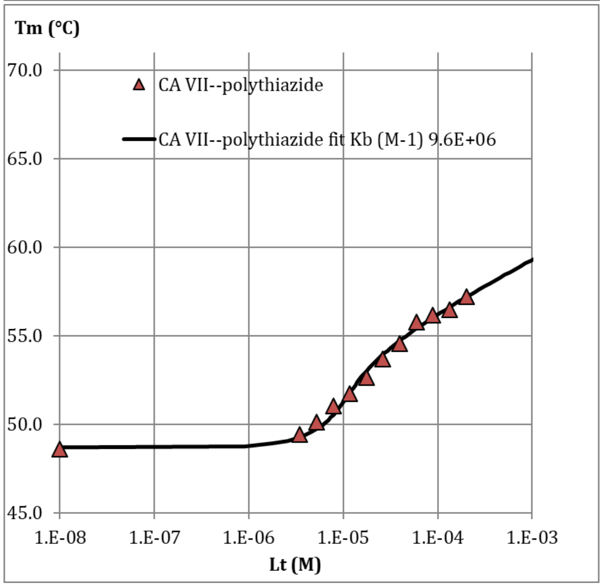
Tm (°C)
CA VII--polythiazide
CA VII--polythiazide fit Kb (M-1) 9.6E+06
70.0
65.0
60.0
55.0
50.0
45.0
1.E-08
1.E-07
1.E-06
1.E-05
1.E-04
1.E-03
Lt (M)

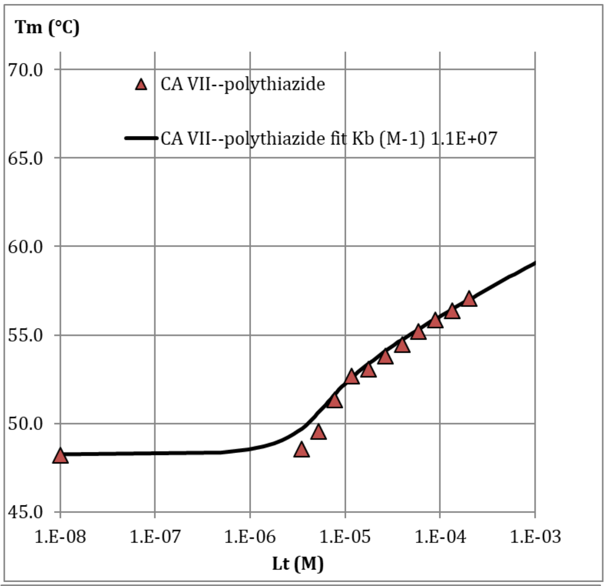
Tm (°C)
CA VII--polythiazide
CA VII--polythiazide fit Kb (M-1) 1.1E+07
70.0
65.0
60.0
55.0
50.0
45.0
1.E-08
1.E-07
1.E-06
1.E-05
1.E-04
1.E-03
Lt (M)

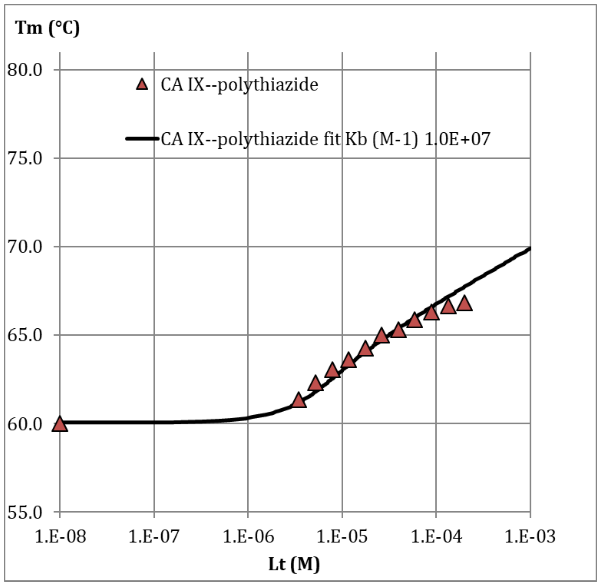
Tm (°C)
CA IX--polythiazide
CA IX--polythiazide fit Kb (M-1) 1.0E+07
80.0
75.0
70.0
65.0
60.0
55.0
1.E-08
1.E-07
1.E-06
1.E-05
1.E-04
1.E-03
Lt (M)

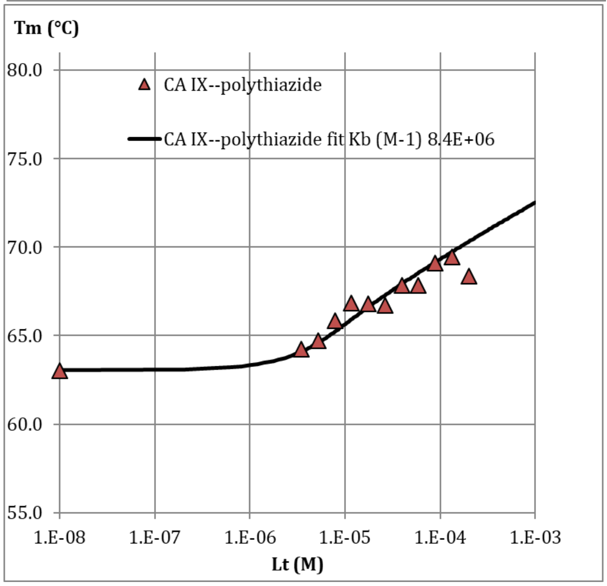
Tm (°C)
CA IX--polythiazide
CA IX--polythiazide fit Kb (M-1) 8.4E+06
80.0
75.0
70.0
65.0
60.0
55.0
1.E-08
1.E-07
1.E-06
1.E-05
1.E-04
1.E-03
Lt (M)

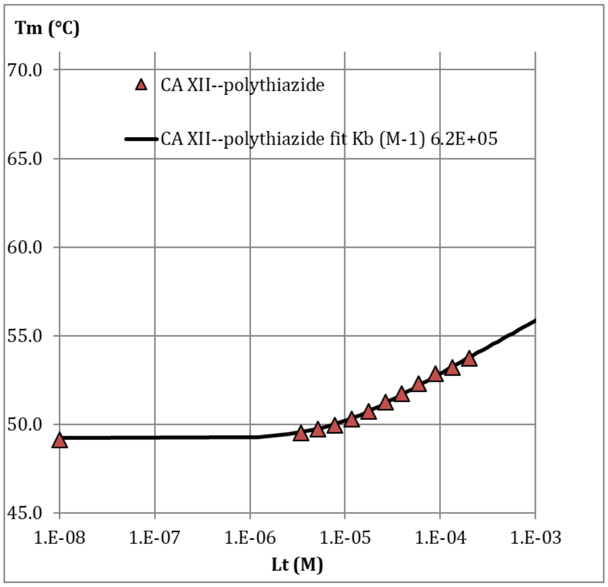
Tm (°C)
CA XII--polythiazide
CA XII--polythiazide fit Kb (M-1) 6.2E+05
Lt (M)

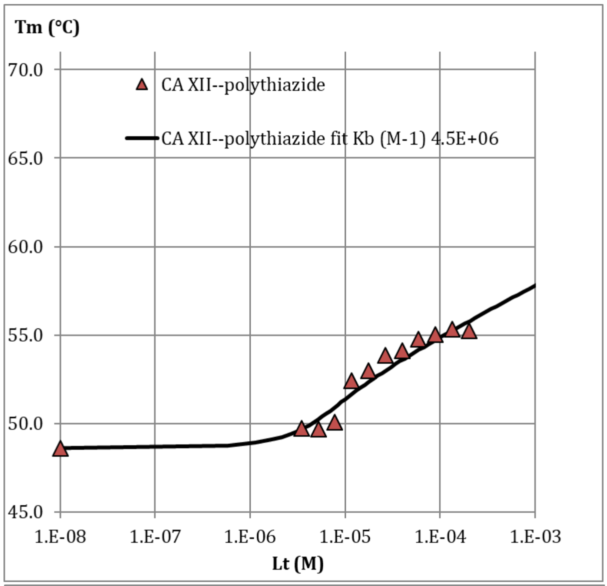
Tm (°C)
CA XII--polythiazide
CA XII--polythiazide fit Kb (M-1) 4.5E+06
Lt (M)

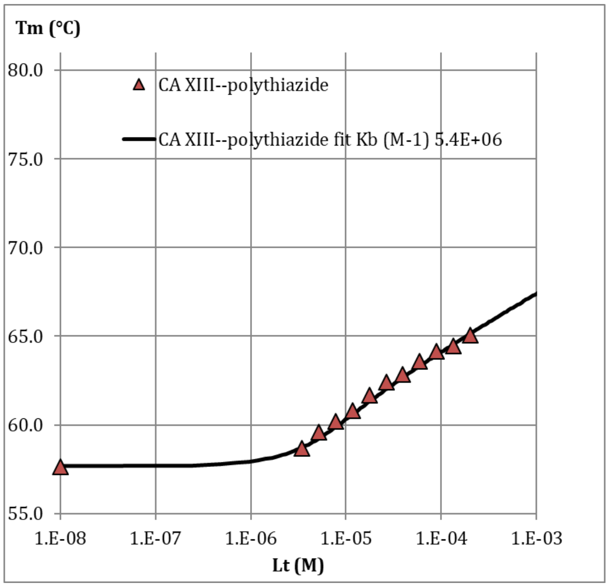
Tm (°C)
CA XIII--polythiazide
CA XIII--polythiazide fit Kb (M-1) 5.4E+06
Lt (M)

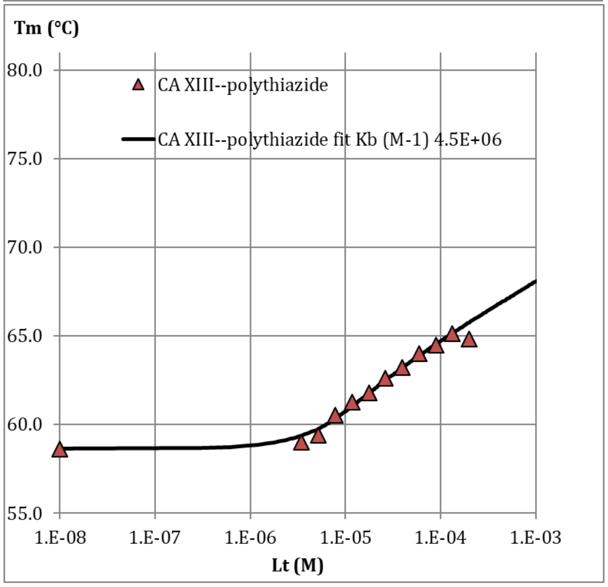
Tm (°C)
CA XIII--polythiazide
CA XIII--polythiazide fit Kb (M-1) 4.5E+06
Lt (M)

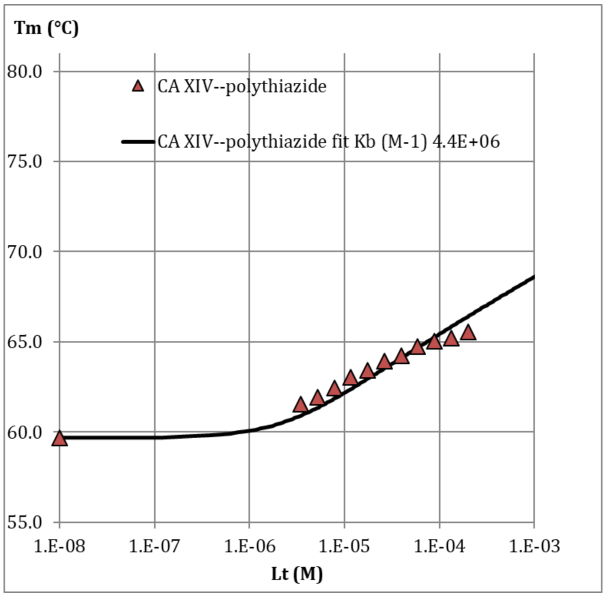
Tm (°C)
CA XIV--polythiazide
CA XIV--polythiazide fit Kb (M-1) 4.4E+06
Lt (M)

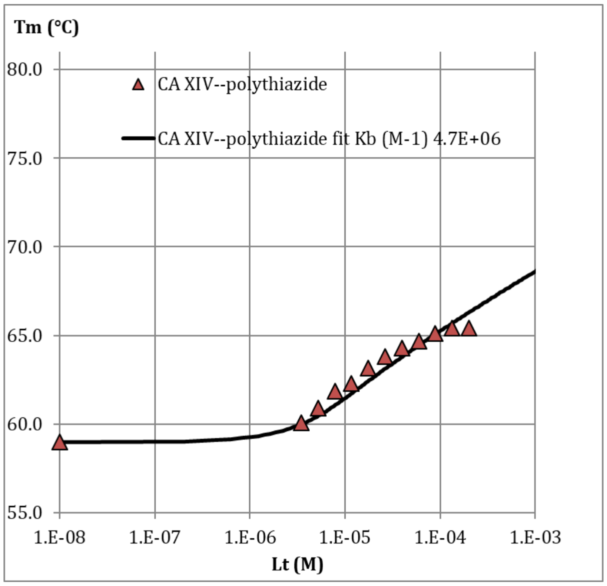
Tm (°C)
CA XIV--polythiazide
CA XIV--polythiazide fit Kb (M-1) 4.7E+06
Lt (M)

8. Benzthiazide

Fluorecence-based thermal shift assay

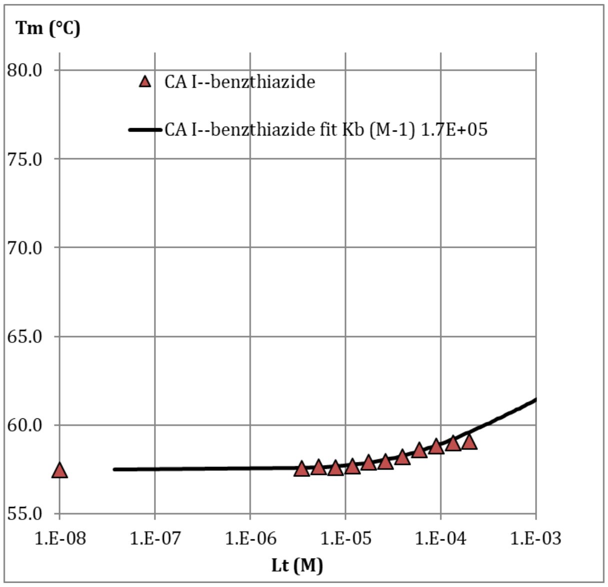

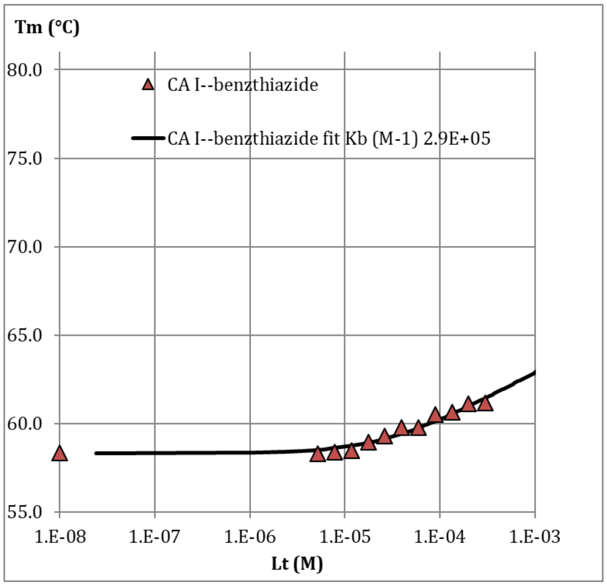

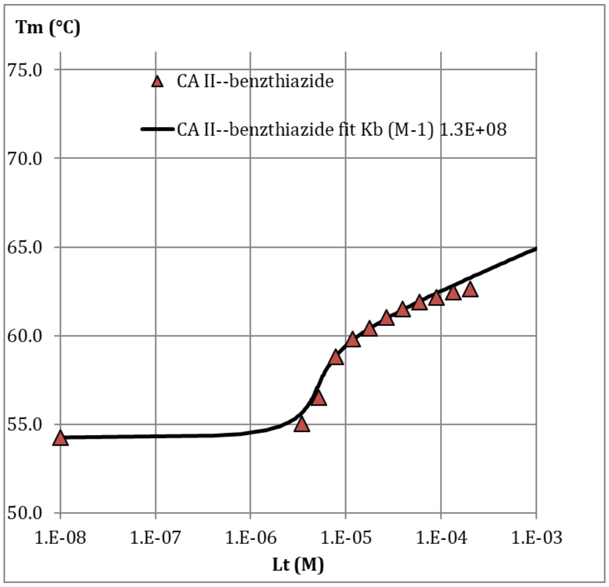

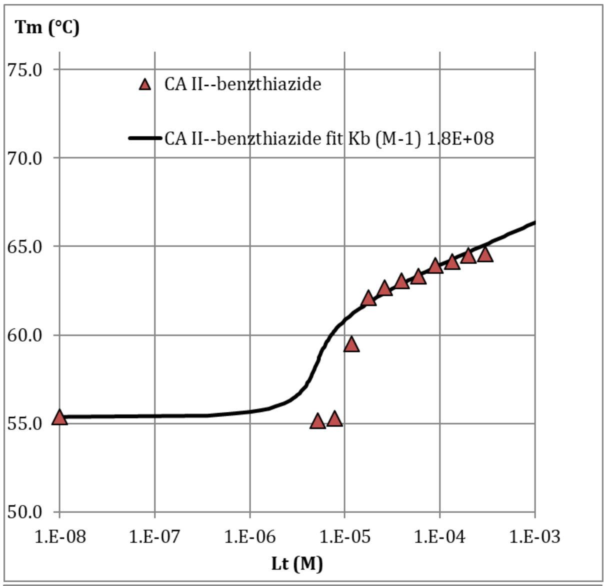

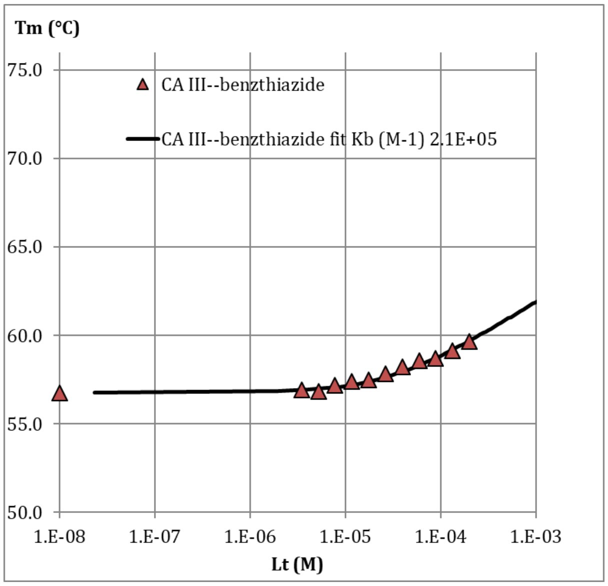

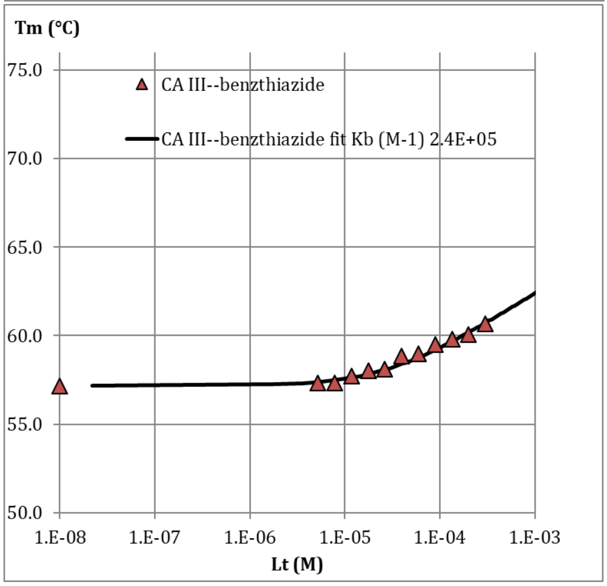

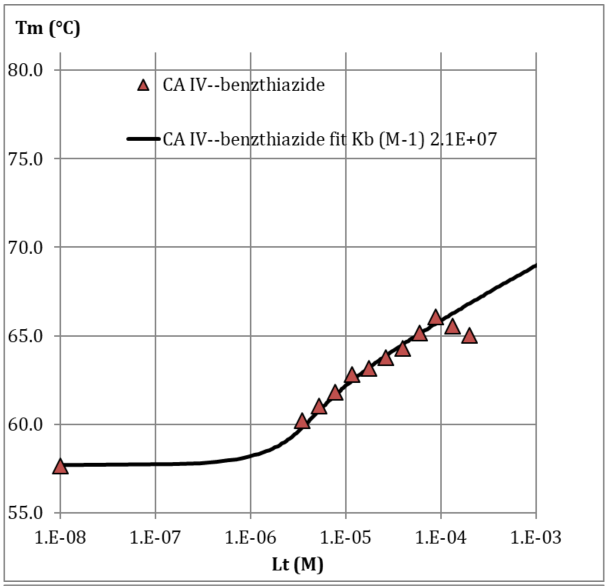
Tm (°C)
80.0
75.0
70.0
65.0
60.0
55.0
CA IV--benzthiazide
CA IV--benzthiazide fit Kb (M-1) 2.1E+07
1.E-08
1.E-07
1.E-06
1.E-05
1.E-04
1.E-03
Lt (M)

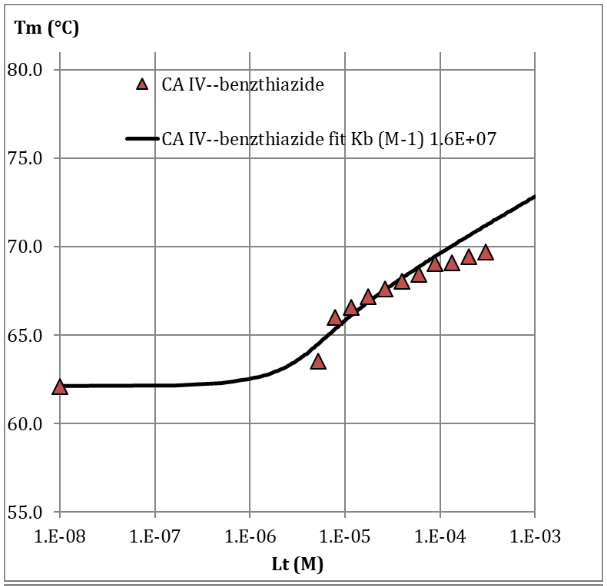
Tm (°C)
80.0
75.0
70.0
65.0
60.0
55.0
CA IV--benzthiazide
CA IV--benzthiazide fit Kb (M-1) 1.6E+07
1.E-08
1.E-07
1.E-06
1.E-05
1.E-04
1.E-03
Lt (M)

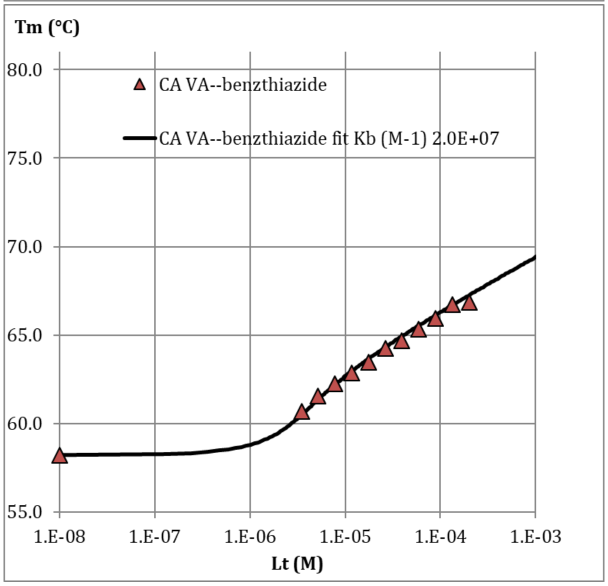
Tm (°C)
80.0
75.0
70.0
65.0
60.0
55.0
CA VA--benzthiazide
CA VA--benzthiazide fit Kb (M-1) 2.0E+07
1.E-08
1.E-07
1.E-06
1.E-05
1.E-04
1.E-03
Lt (M)

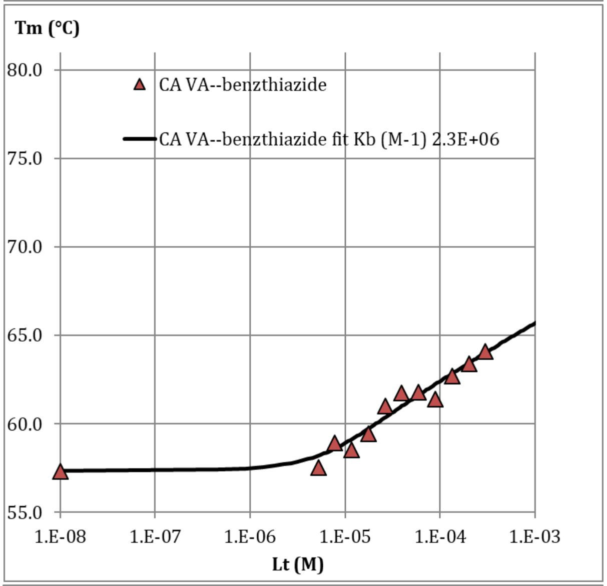
Tm (°C)
80.0
75.0
70.0
65.0
60.0
55.0
CA VA--benzthiazide
CA VA--benzthiazide fit Kb (M-1) 2.3E+06
1.E-08
1.E-07
1.E-06
1.E-05
1.E-04
1.E-03
Lt (M)

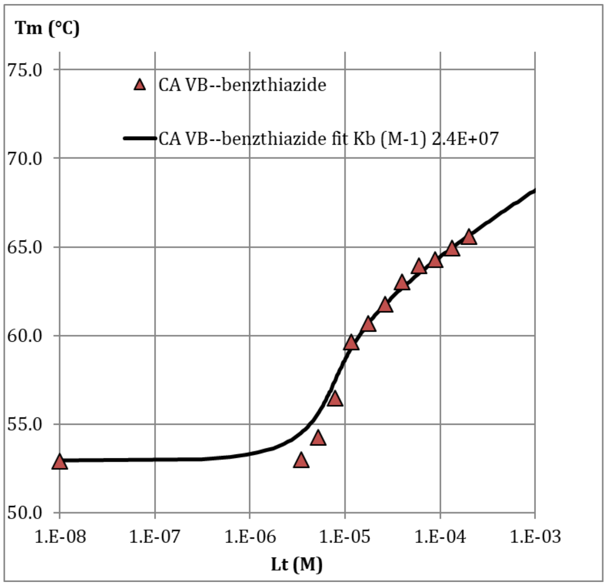
Tm (°C)
75.0
70.0
65.0
60.0
55.0
50.0
CA VB--benzthiazide
CA VB--benzthiazide fit Kb (M-1) 2.4E+07
1.E-08
1.E-07
1.E-06
1.E-05
1.E-04
1.E-03
Lt (M)

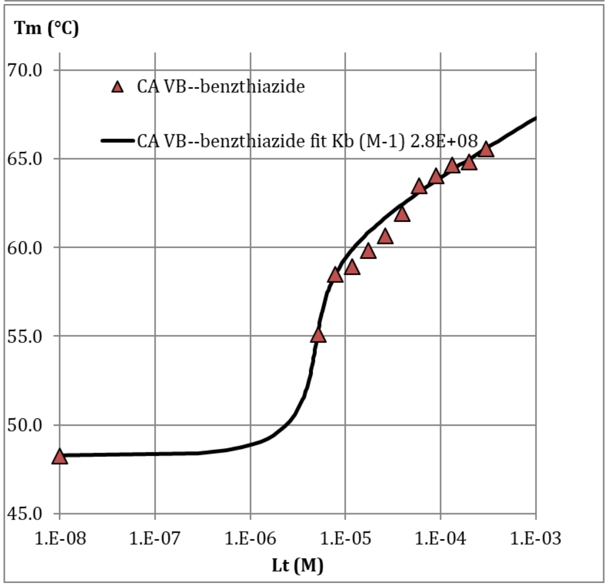
Tm (°C)
70.0
65.0
60.0
55.0
50.0
45.0
CA VB--benzthiazide
CA VB--benzthiazide fit Kb (M-1) 2.8E+08
1.E-08
1.E-07
1.E-06
1.E-05
1.E-04
1.E-03
Lt (M)

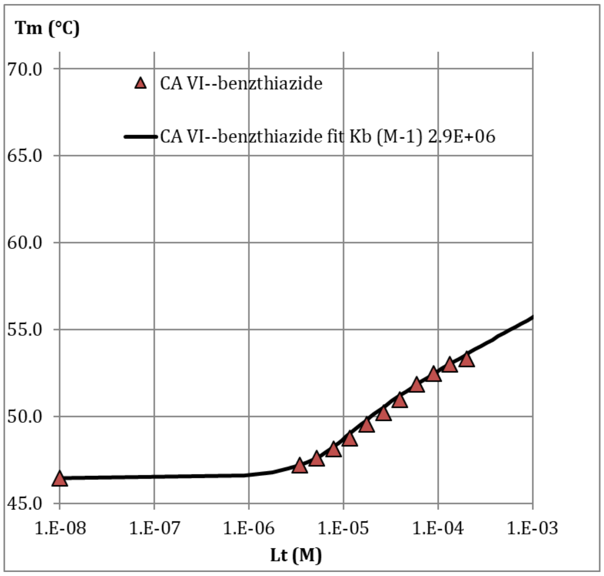
Tm (°C)
70.0
65.0
60.0
55.0
50.0
45.0
CA VI--benzthiazide
CA VI--benzthiazide fit Kb (M-1) 2.9E+06
1.E-08
1.E-07
1.E-06
1.E-05
1.E-04
1.E-03
Lt (M)

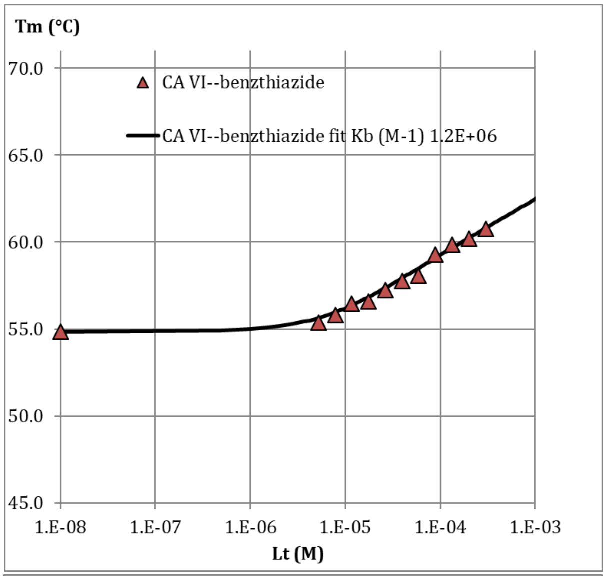
Tm (°C)
70.0
65.0
60.0
55.0
50.0
45.0
CA VI--benzthiazide
CA VI--benzthiazide fit Kb (M-1) 1.2E+06
1.E-08
1.E-07
1.E-06
1.E-05
1.E-04
1.E-03
Lt (M)

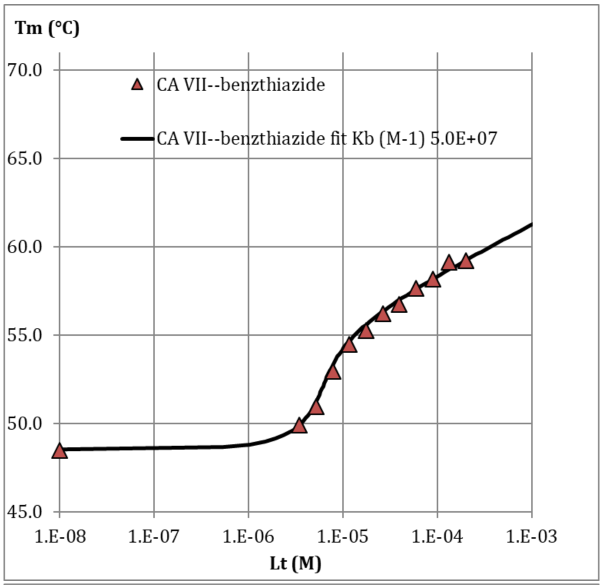
Tm (°C)
70.0
65.0
60.0
55.0
50.0
45.0
CA VII--benzthiazide
CA VII--benzthiazide fit Kb (M-1) 5.0E+07
1.E-08
1.E-07
1.E-06
1.E-05
1.E-04
1.E-03
Lt (M)

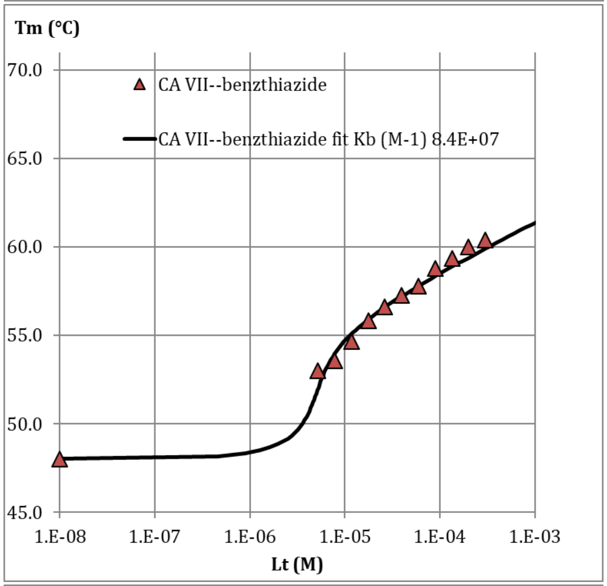
Tm (°C)
70.0
65.0
60.0
55.0
50.0
45.0
CA VII--benzthiazide
CA VII--benzthiazide fit Kb (M-1) 8.4E+07
1.E-08
1.E-07
1.E-06
1.E-05
1.E-04
1.E-03
Lt (M)

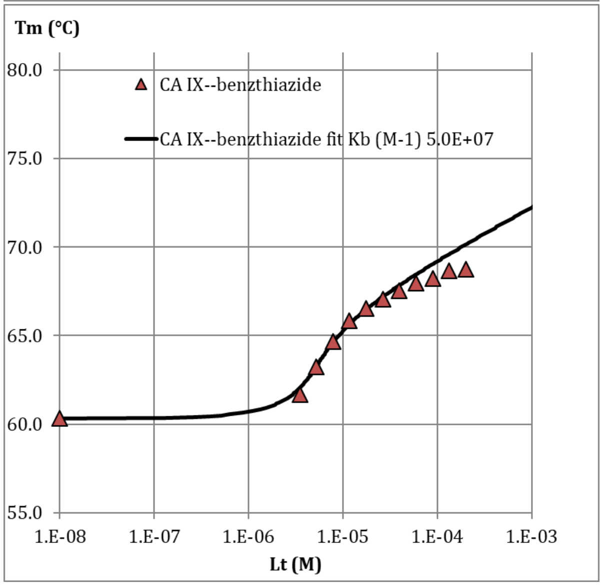
Tm (°C)
80.0
75.0
70.0
65.0
60.0
55.0
CA IX--benzthiazide
CA IX--benzthiazide fit Kb (M-1) 5.0E+07
1.E-08
1.E-07
1.E-06
1.E-05
1.E-04
1.E-03
Lt (M)

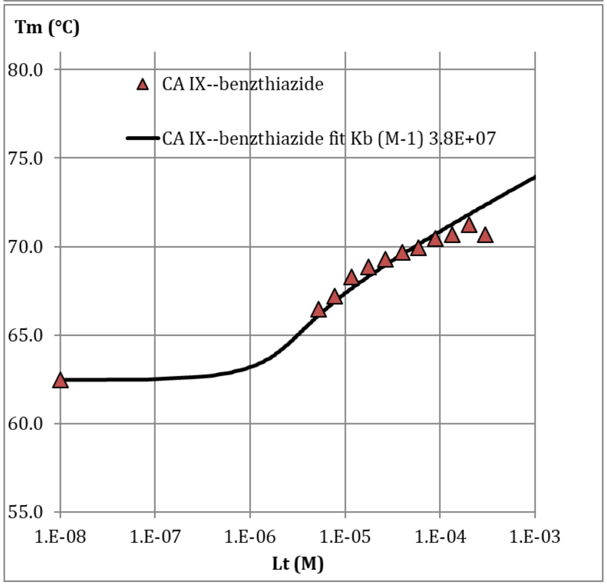
Tm (°C)
80.0
75.0
70.0
65.0
60.0
55.0
CA IX--benzthiazide
CA IX--benzthiazide fit Kb (M-1) 3.8E+07
1.E-08
1.E-07
1.E-06
1.E-05
1.E-04
1.E-03
Lt (M)

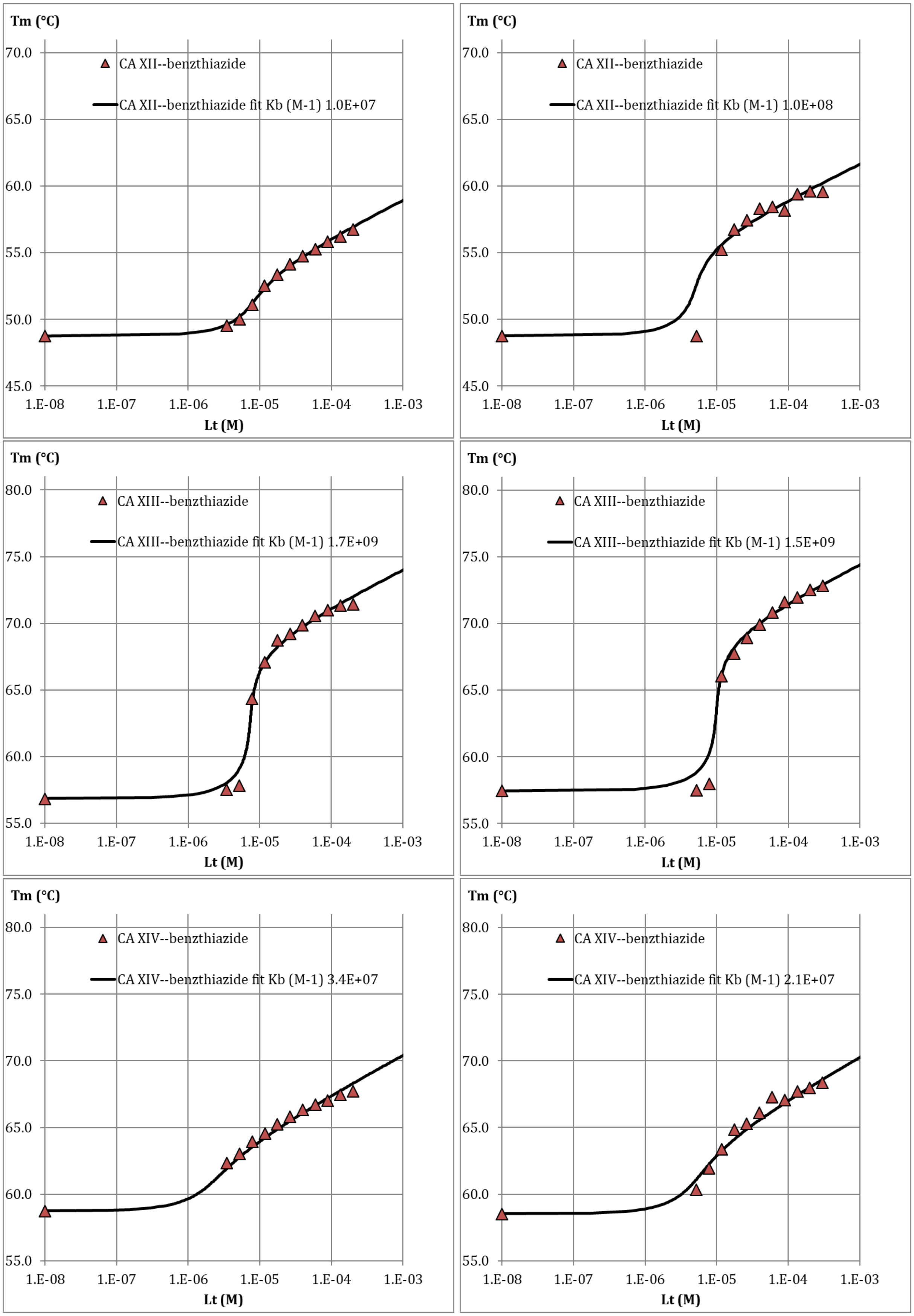
Tm (°C)
CA XII--benzthiazide
CA XII--benzthiazide fit Kb (M-1) 1.0E+07
Lt (M)
Tm (°C)
CA XII--benzthiazide
CA XII--benzthiazide fit Kb (M-1) 1.0E+08
Lt (M)
Tm (°C)
CA XIII--benzthiazide
CA XIII--benzthiazide fit Kb (M-1) 1.7E+09
Lt (M)
Tm (°C)
CA XIII--benzthiazide
CA XIII--benzthiazide fit Kb (M-1) 1.5E+09
Lt (M)
Tm (°C)
CA XIV--benzthiazide
CA XIV--benzthiazide fit Kb (M-1) 3.4E+07
Lt (M)
Tm (°C)
CA XIV--benzthiazide
CA XIV--benzthiazide fit Kb (M-1) 2.1E+07
Lt (M)

Isothermal titration calorimetry

CA II – benzthiazide

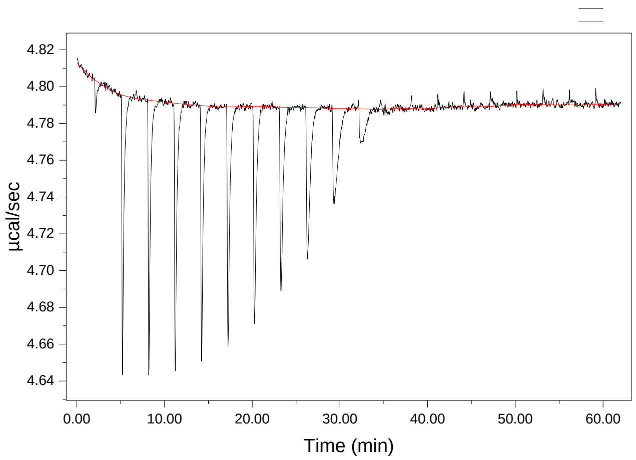

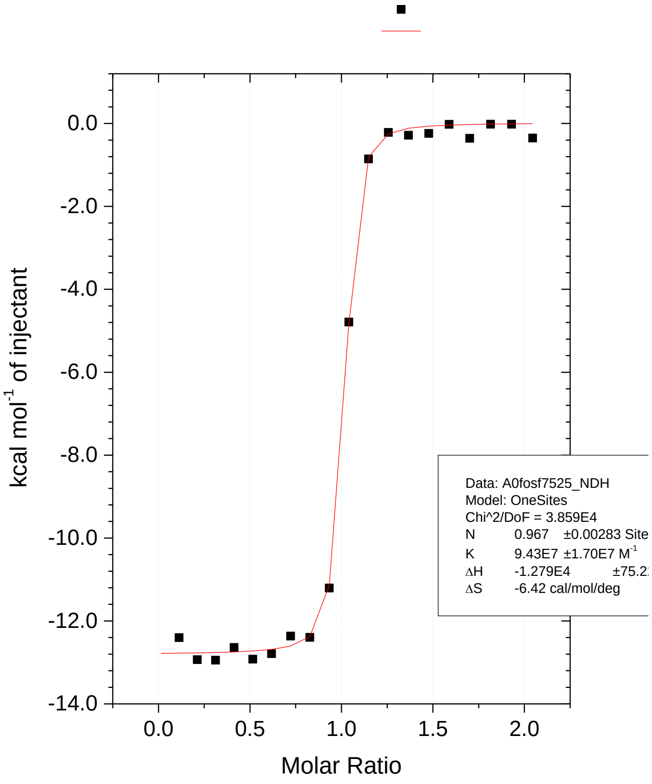

CA II – benzthiazide

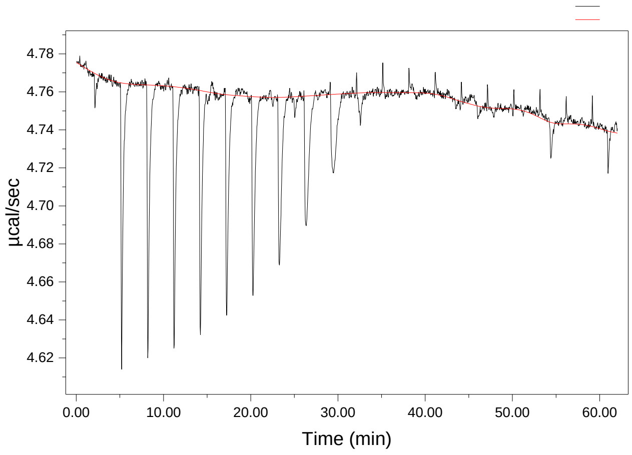

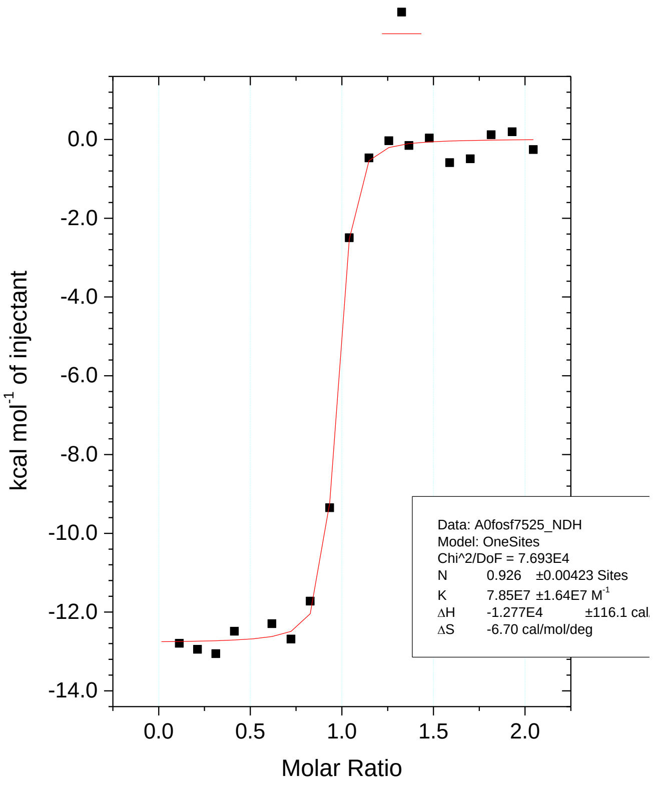

9. Athiazide

Fluorecence-based thermal shift assay

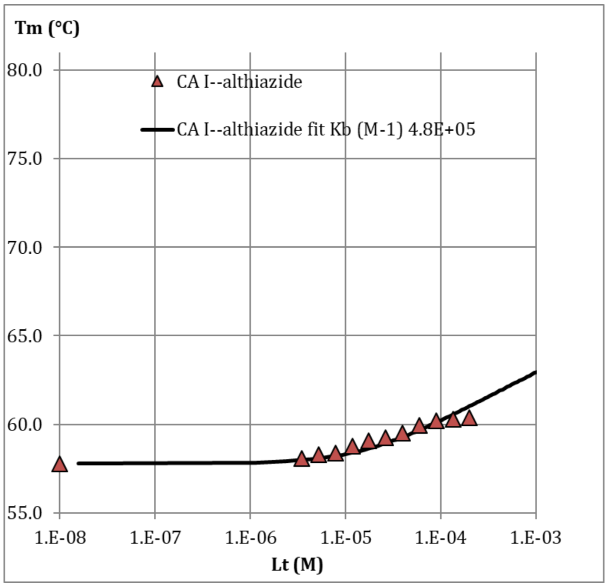

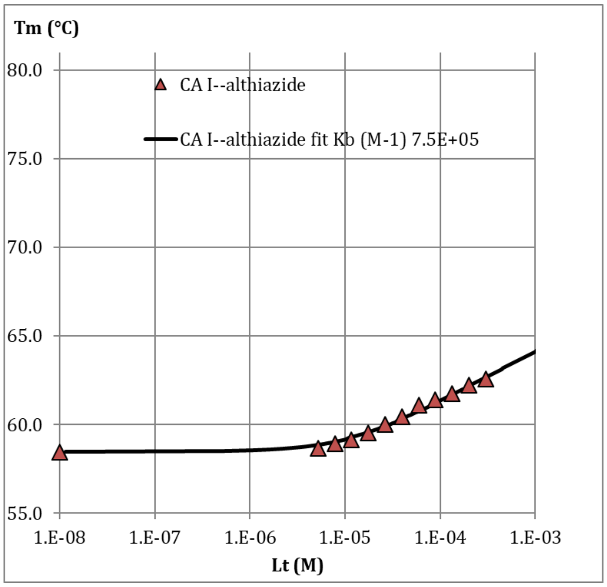

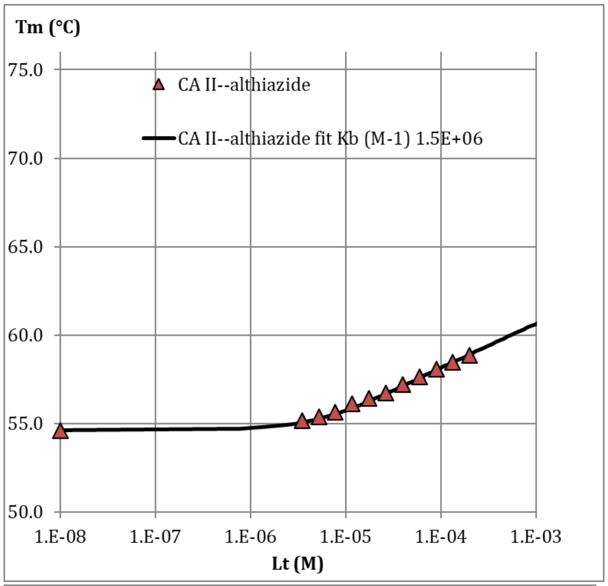

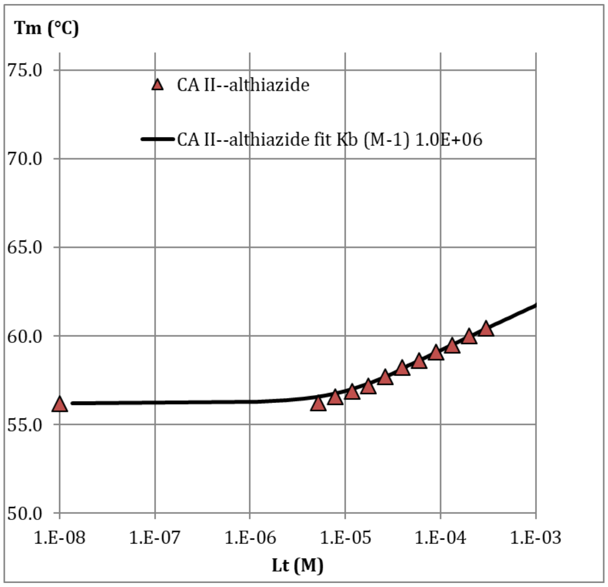

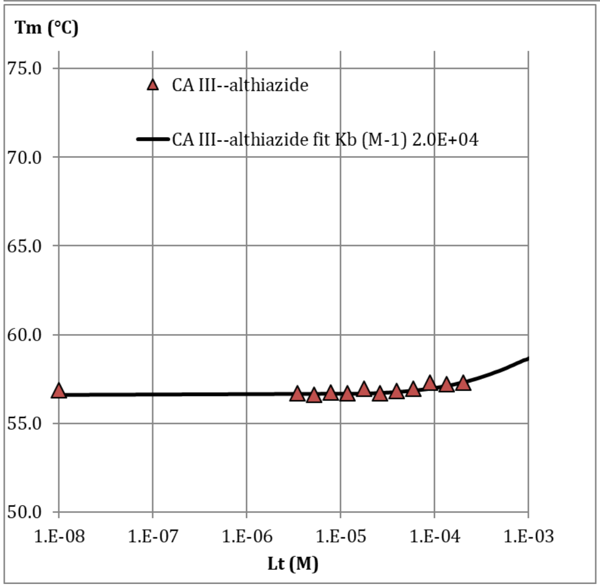

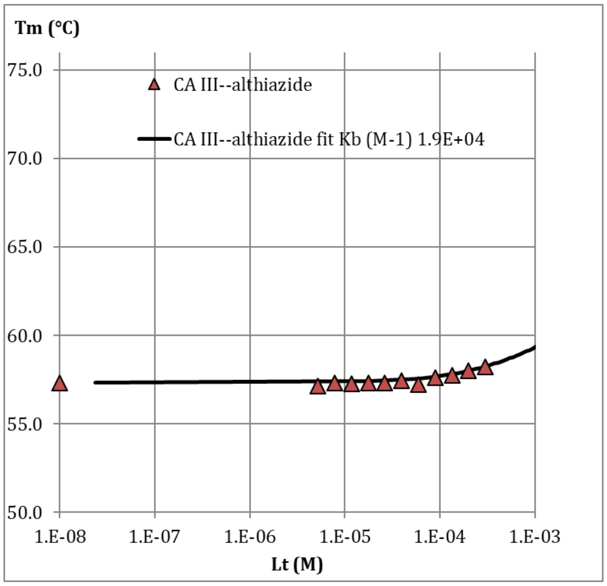

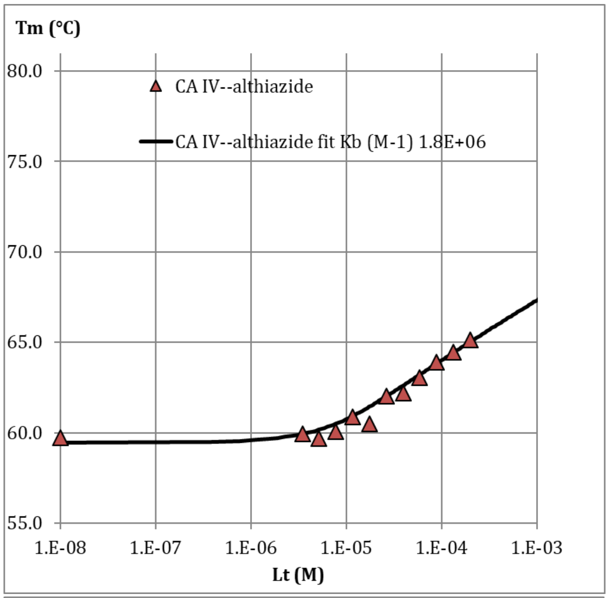
Tm (°C)
CA IV--althiazide
CA IV--althiazide fit Kb (M-1) 1.8E+06
80.0
75.0
70.0
65.0
60.0
55.0
1.E-08
1.E-07
1.E-06
1.E-05
1.E-04
1.E-03
Lt (M)

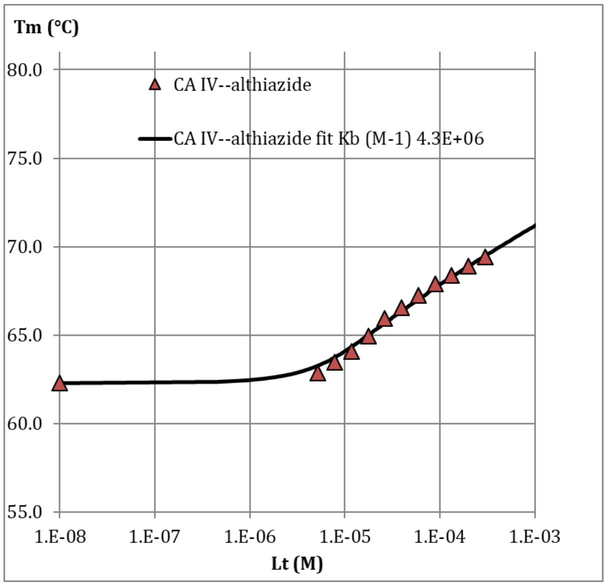
Tm (°C)
CA IV--althiazide
CA IV--althiazide fit Kb (M-1) 4.3E+06
80.0
75.0
70.0
65.0
60.0
55.0
1.E-08
1.E-07
1.E-06
1.E-05
1.E-04
1.E-03
Lt (M)

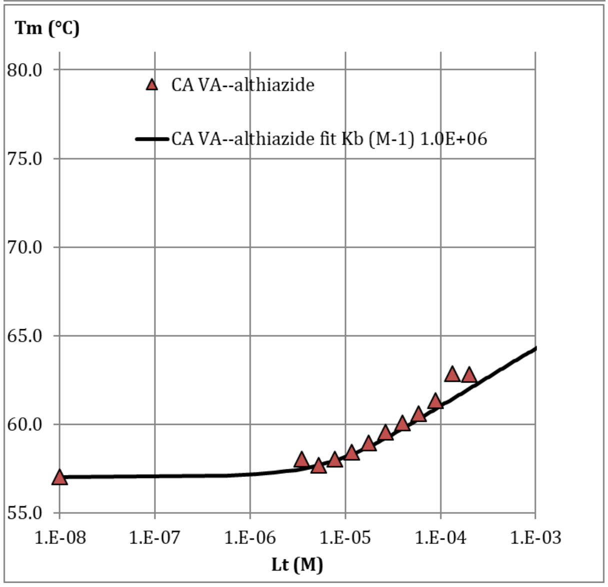
Tm (°C)
CA VA--althiazide
CA VA--althiazide fit Kb (M-1) 1.0E+06
80.0
75.0
70.0
65.0
60.0
55.0
1.E-08
1.E-07
1.E-06
1.E-05
1.E-04
1.E-03
Lt (M)

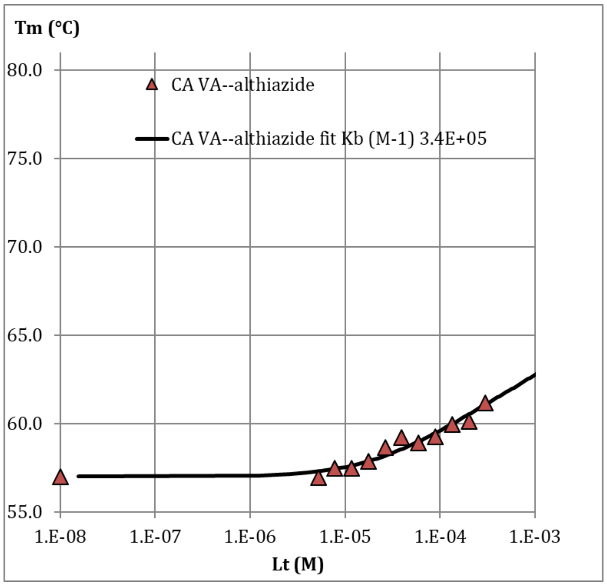
Tm (°C)
CA VA--althiazide
CA VA--althiazide fit Kb (M-1) 3.4E+05
80.0
75.0
70.0
65.0
60.0
55.0
1.E-08
1.E-07
1.E-06
1.E-05
1.E-04
1.E-03
Lt (M)

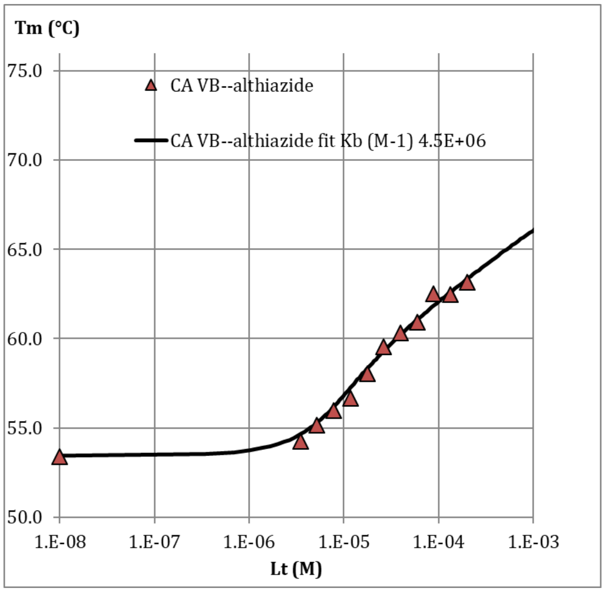
Tm (°C)
CA VB--althiazide
CA VB--althiazide fit Kb (M-1) 4.5E+06
75.0
70.0
65.0
60.0
55.0
50.0
1.E-08
1.E-07
1.E-06
1.E-05
1.E-04
1.E-03
Lt (M)

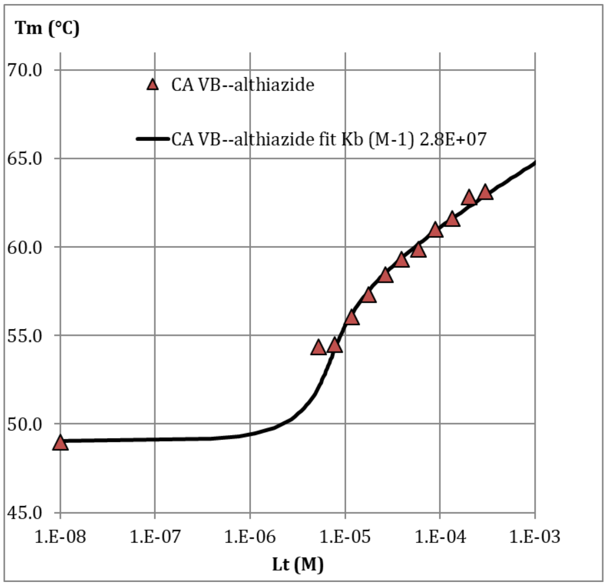
Tm (°C)
CA VB--althiazide
CA VB--althiazide fit Kb (M-1) 2.8E+07
70.0
65.0
60.0
55.0
50.0
45.0
1.E-08
1.E-07
1.E-06
1.E-05
1.E-04
1.E-03
Lt (M)

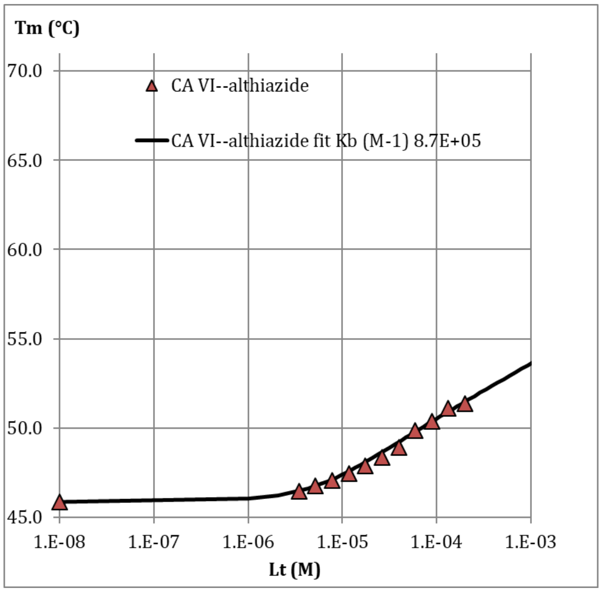
Tm (°C)
CA VI--althiazide
CA VI--althiazide fit Kb (M-1) 8.7E+05
70.0
65.0
60.0
55.0
50.0
45.0
1.E-08 1.E-07 1.E-06 1.E-05 1.E-04 1.E-03
Lt (M)

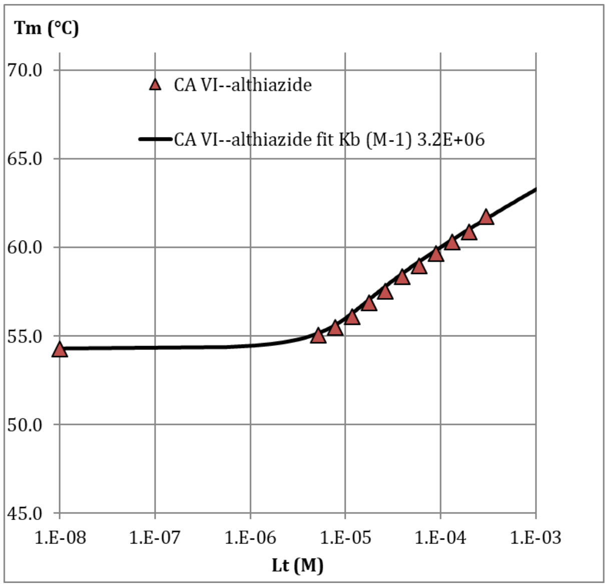
Tm (°C)
CA VI--althiazide
CA VI--althiazide fit Kb (M-1) 3.2E+06
70.0
65.0
60.0
55.0
50.0
45.0
1.E-08 1.E-07 1.E-06 1.E-05 1.E-04 1.E-03
Lt (M)

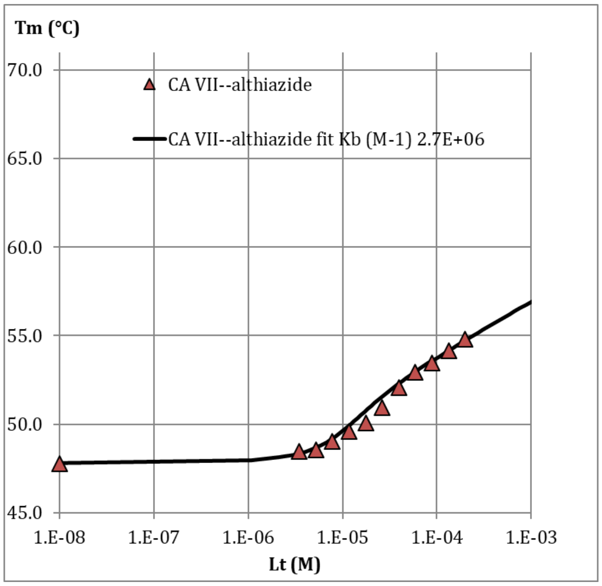
Tm (°C)
CA VII--althiazide
CA VII--althiazide fit Kb (M-1) 2.7E+06
70.0
65.0
60.0
55.0
50.0
45.0
1.E-08 1.E-07 1.E-06 1.E-05 1.E-04 1.E-03
Lt (M)

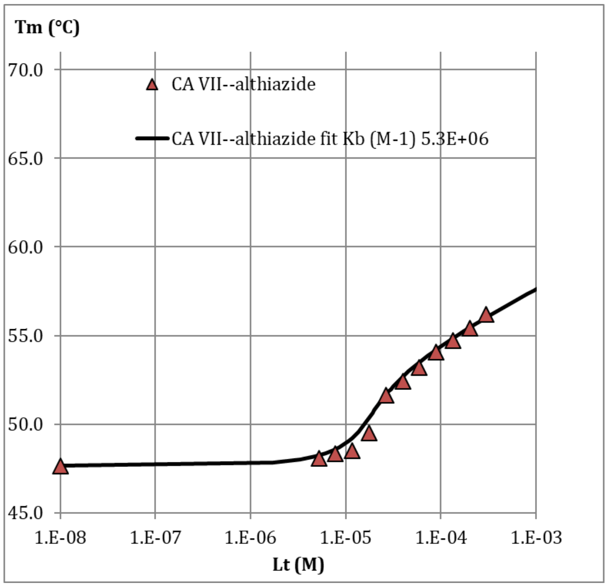
Tm (°C)
CA VII--althiazide
CA VII--althiazide fit Kb (M-1) 5.3E+06
70.0
65.0
60.0
55.0
50.0
45.0
1.E-08 1.E-07 1.E-06 1.E-05 1.E-04 1.E-03
Lt (M)

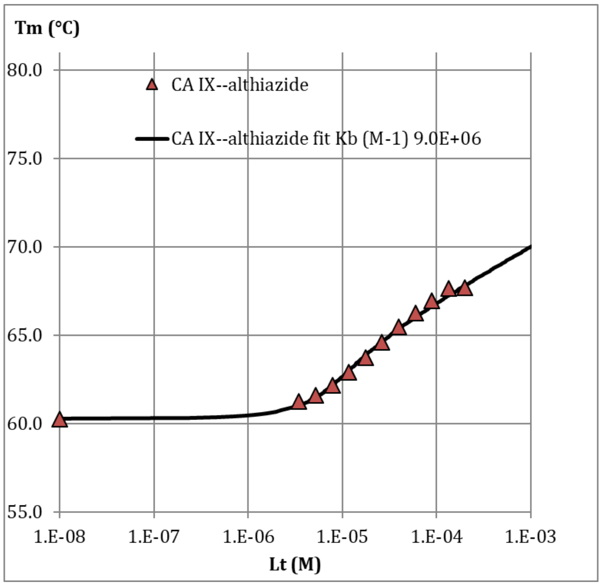
Tm (°C)
CA IX--althiazide
CA IX--althiazide fit Kb (M-1) 9.0E+06
80.0
75.0
70.0
65.0
60.0
55.0
1.E-08 1.E-07 1.E-06 1.E-05 1.E-04 1.E-03
Lt (M)

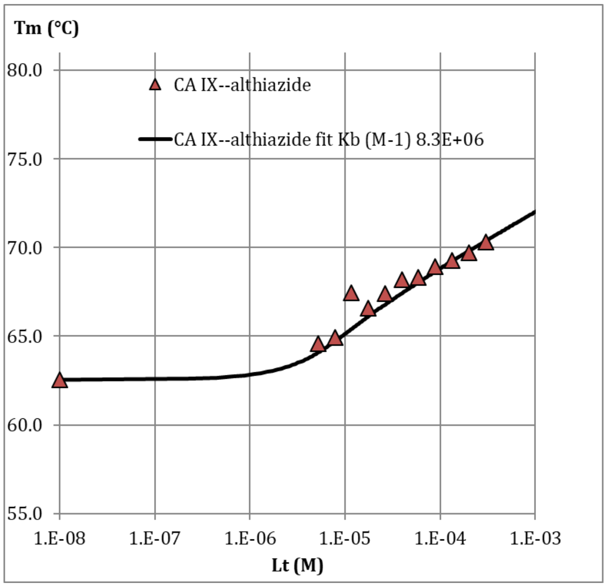
Tm (°C)
CA IX--althiazide
CA IX--althiazide fit Kb (M-1) 8.3E+06
80.0
75.0
70.0
65.0
60.0
55.0
1.E-08 1.E-07 1.E-06 1.E-05 1.E-04 1.E-03
Lt (M)

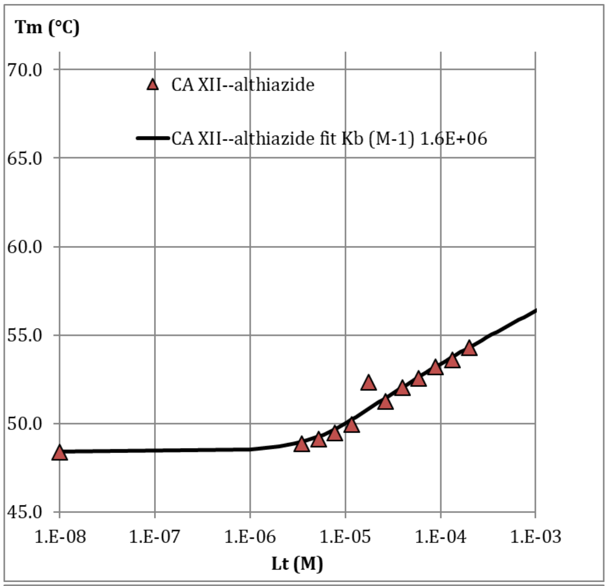
Tm (°C)
CA XII--althiazide
CA XII--althiazide fit Kb (M-1) 1.6E+06
70.0
65.0
60.0
55.0
50.0
45.0
1.E-08
1.E-07
1.E-06
1.E-05
1.E-04
1.E-03
Lt (M)

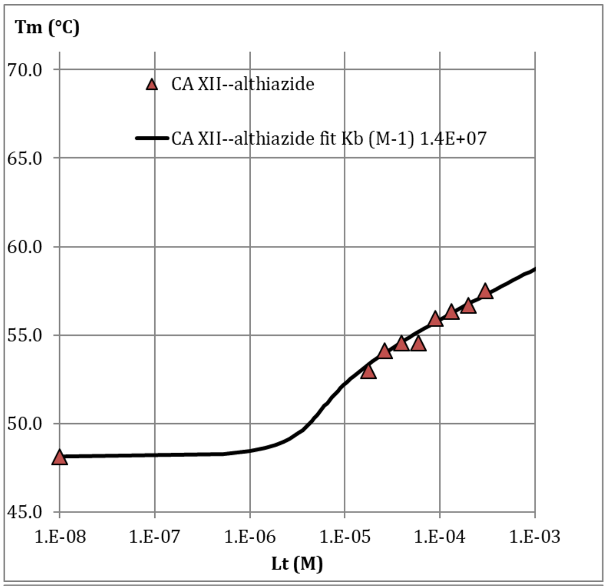
Tm (°C)
CA XII--althiazide
CA XII--althiazide fit Kb (M-1) 1.4E+07
70.0
65.0
60.0
55.0
50.0
45.0
1.E-08
1.E-07
1.E-06
1.E-05
1.E-04
1.E-03
Lt (M)

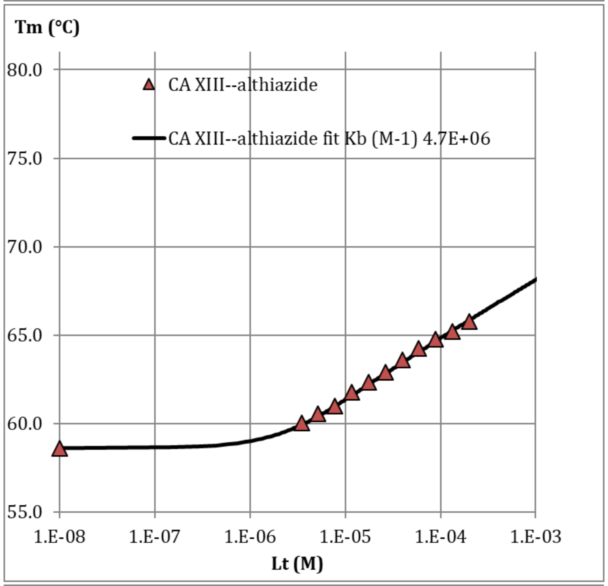
Tm (°C)
CA XIII--althiazide
CA XIII--althiazide fit Kb (M-1) 4.7E+06
80.0
75.0
70.0
65.0
60.0
55.0
1.E-08
1.E-07
1.E-06
1.E-05
1.E-04
1.E-03
Lt (M)

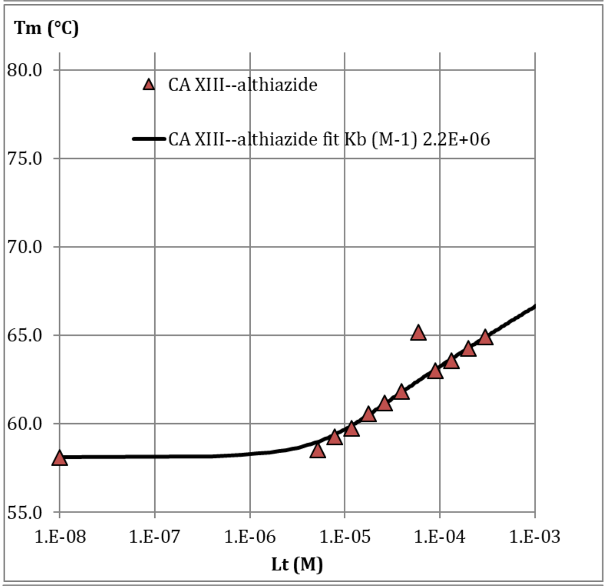
Tm (°C)
CA XIII--althiazide
CA XIII--althiazide fit Kb (M-1) 2.2E+06
80.0
75.0
70.0
65.0
60.0
55.0
1.E-08
1.E-07
1.E-06
1.E-05
1.E-04
1.E-03
Lt (M)

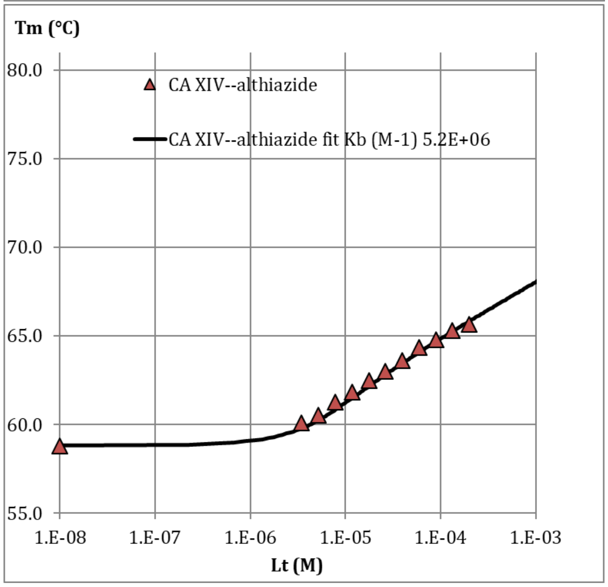
Tm (°C)
CA XIV--althiazide
CA XIV--althiazide fit Kb (M-1) 5.2E+06
80.0
75.0
70.0
65.0
60.0
55.0
1.E-08
1.E-07
1.E-06
1.E-05
1.E-04
1.E-03
Lt (M)

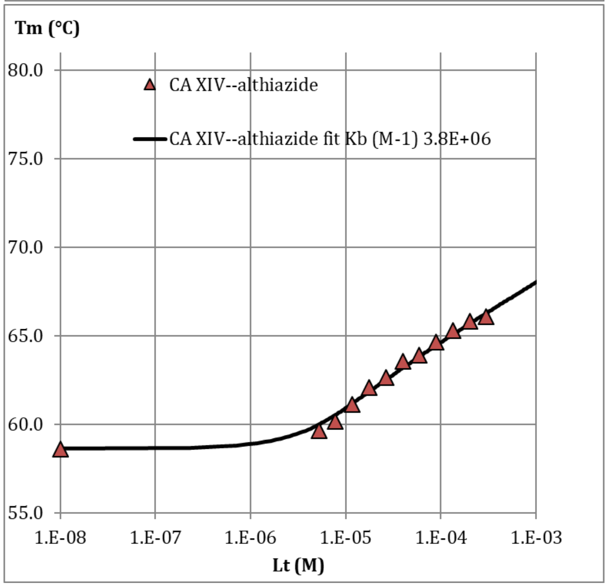
Tm (°C)
CA XIV--althiazide
CA XIV--althiazide fit Kb (M-1) 3.8E+06
80.0
75.0
70.0
65.0
60.0
55.0
1.E-08
1.E-07
1.E-06
1.E-05
1.E-04
1.E-03
Lt (M)

Isothermal titration calorimetry

CA II- althiazide

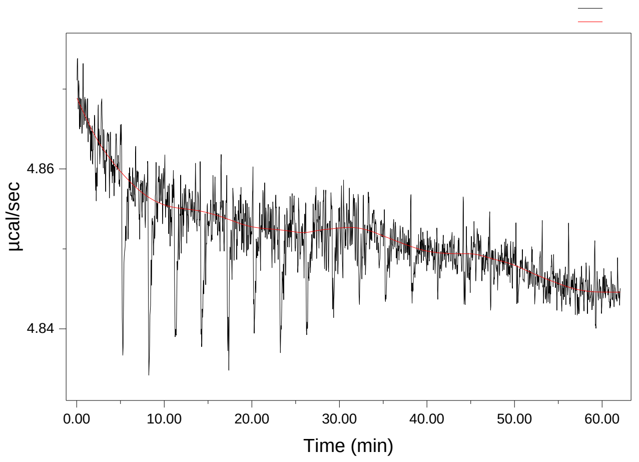

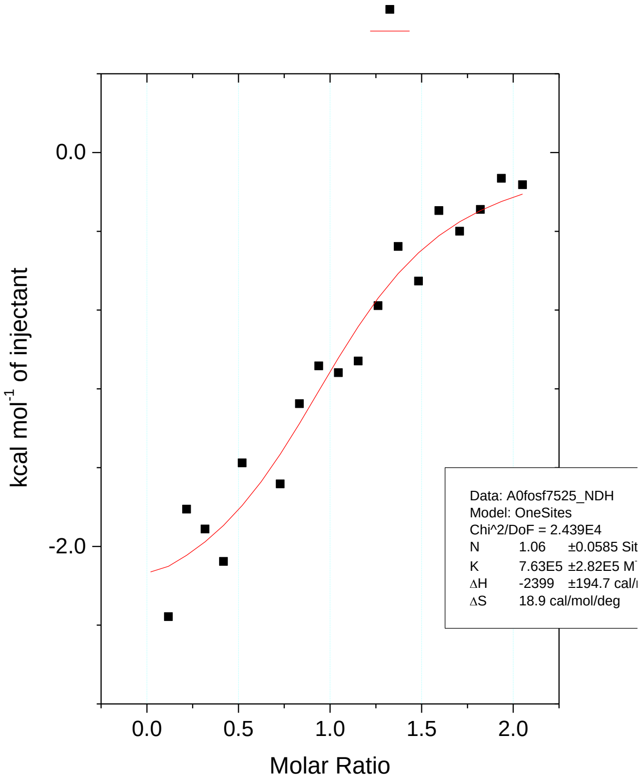

CA II- althiazide

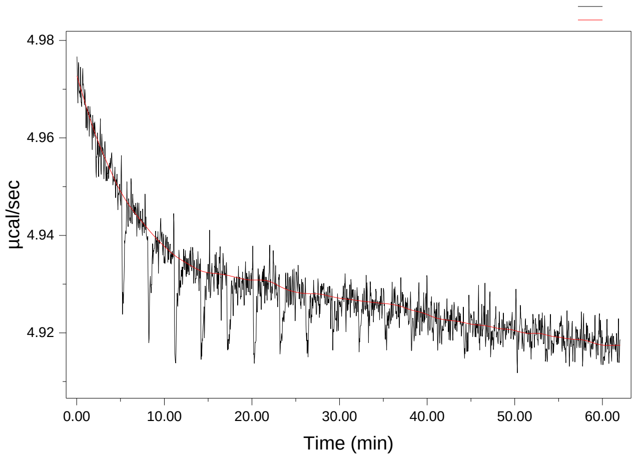

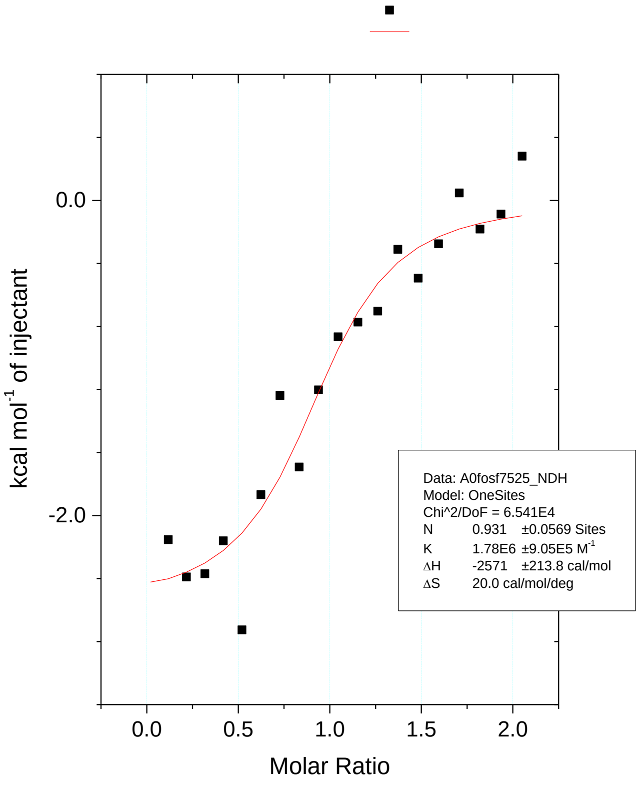

10. Hydroflumethiazide

Fluorecence-based thermal shift assay

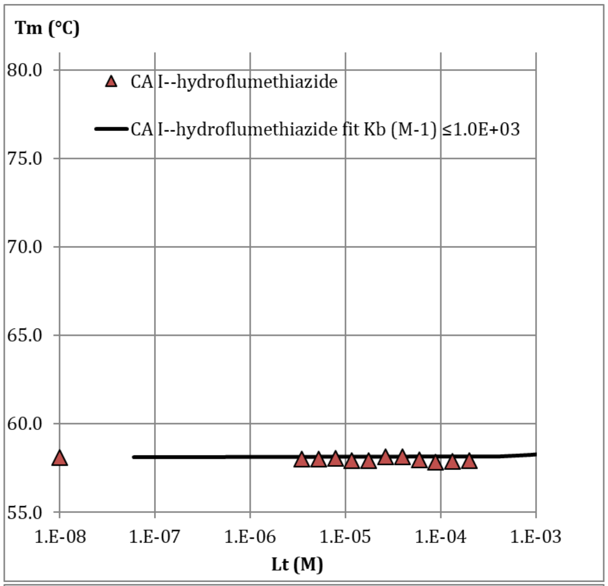

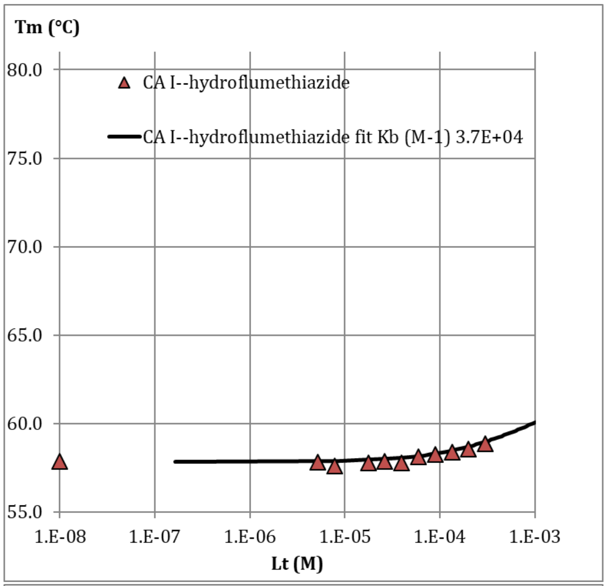

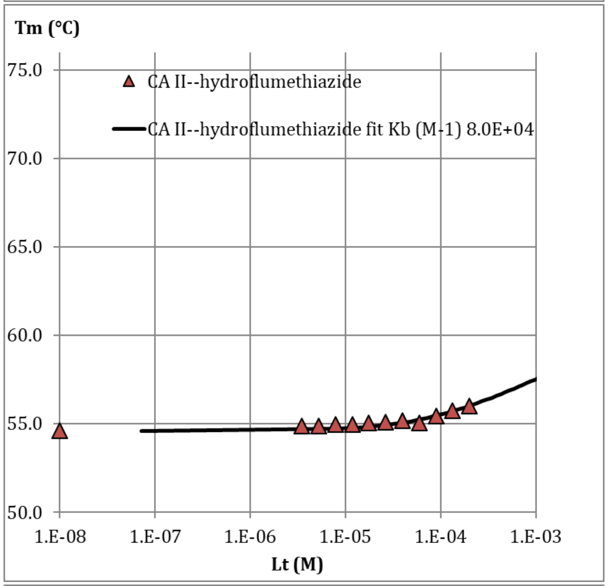

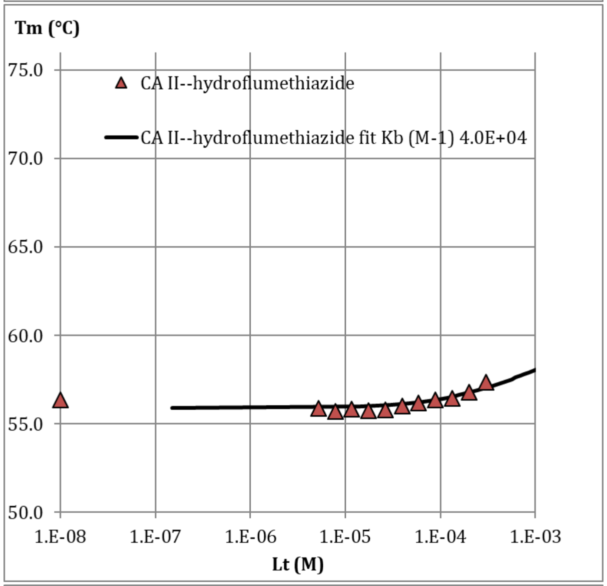

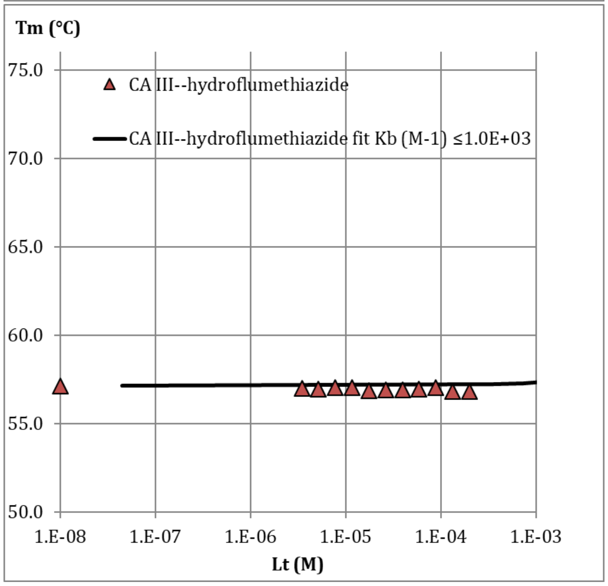

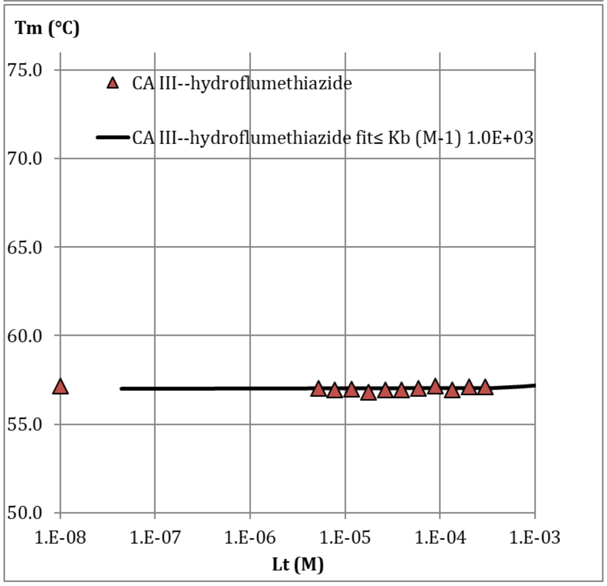

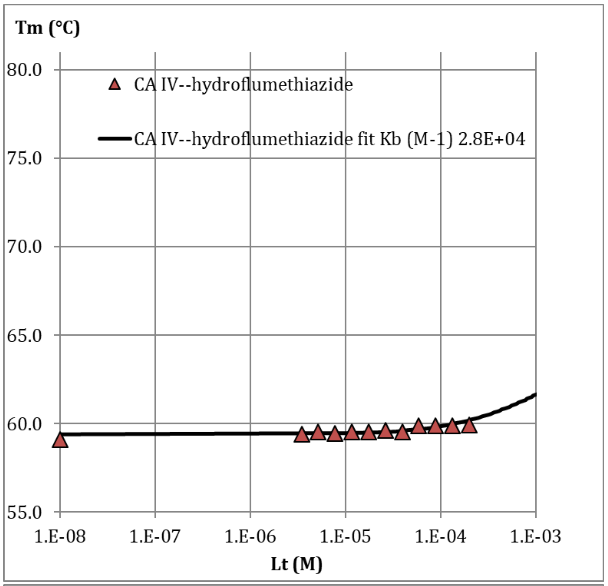
Tm (°C)
CA IV--hydroflumethiazide
CA IV--hydroflumethiazide fit Kb (M-1) 2.8E+04
80.0
75.0
70.0
65.0
60.0
55.0
1.E-08
1.E-07
1.E-06
1.E-05
1.E-04
1.E-03
Lt (M)

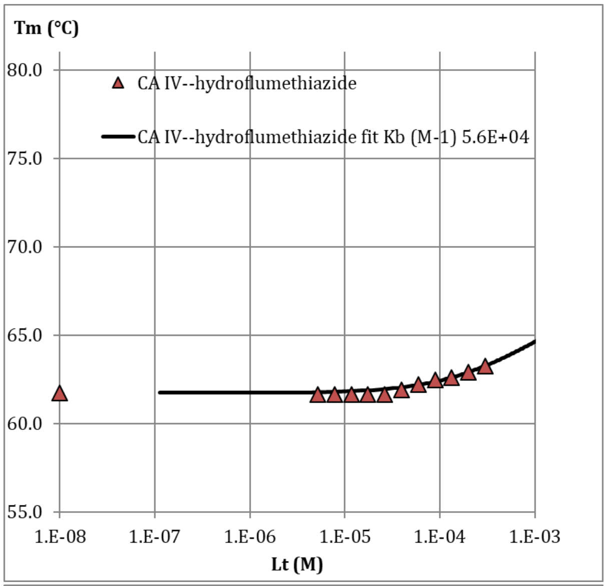
Tm (°C)
CA IV--hydroflumethiazide
CA IV--hydroflumethiazide fit Kb (M-1) 5.6E+04
80.0
75.0
70.0
65.0
60.0
55.0
1.E-08
1.E-07
1.E-06
1.E-05
1.E-04
1.E-03
Lt (M)

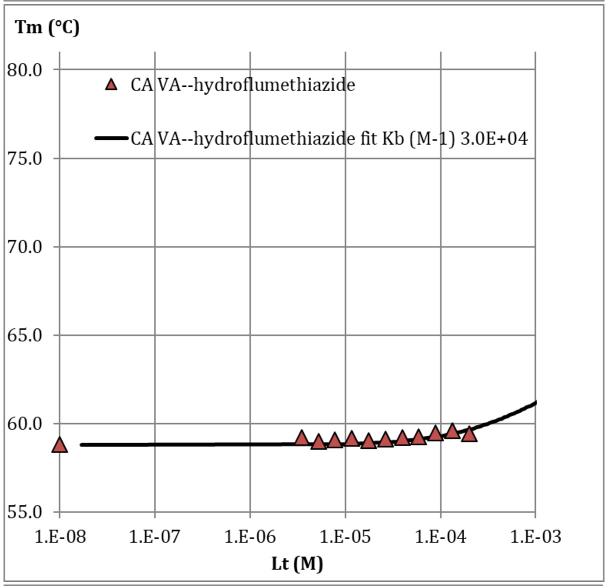
Tm (°C)
CA VA--hydroflumethiazide
CA VA--hydroflumethiazide fit Kb (M-1) 3.0E+04
80.0
75.0
70.0
65.0
60.0
55.0
1.E-08
1.E-07
1.E-06
1.E-05
1.E-04
1.E-03
Lt (M)

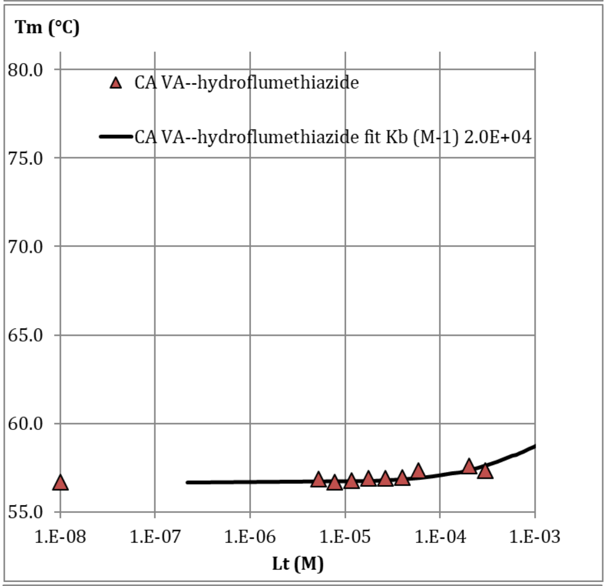
Tm (°C)
CA VA--hydroflumethiazide
CA VA--hydroflumethiazide fit Kb (M-1) 2.0E+04
80.0
75.0
70.0
65.0
60.0
55.0
1.E-08
1.E-07
1.E-06
1.E-05
1.E-04
1.E-03
Lt (M)

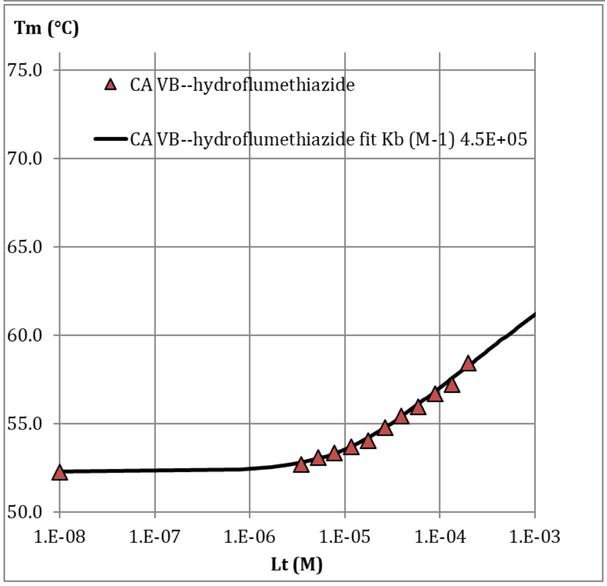
Tm (°C)
CA VB--hydroflumethiazide
CA VB--hydroflumethiazide fit Kb (M-1) 4.5E+05
75.0
70.0
65.0
60.0
55.0
50.0
1.E-08
1.E-07
1.E-06
1.E-05
1.E-04
1.E-03
Lt (M)

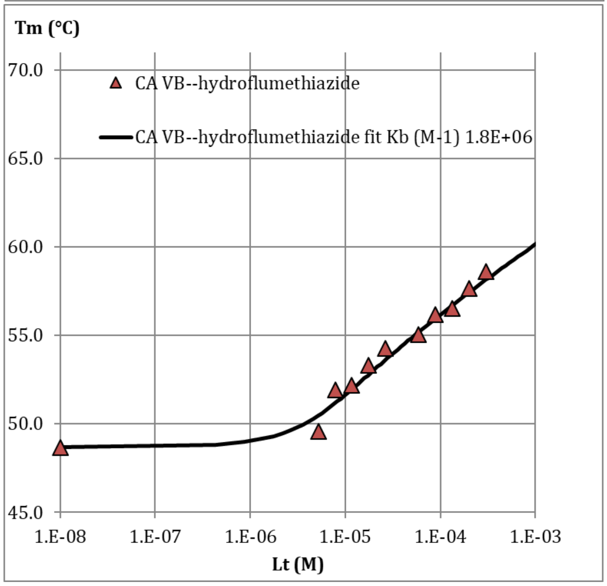
Tm (°C)
CA VB--hydroflumethiazide
CA VB--hydroflumethiazide fit Kb (M-1) 1.8E+06
70.0
65.0
60.0
55.0
50.0
45.0
1.E-08
1.E-07
1.E-06
1.E-05
1.E-04
1.E-03
Lt (M)

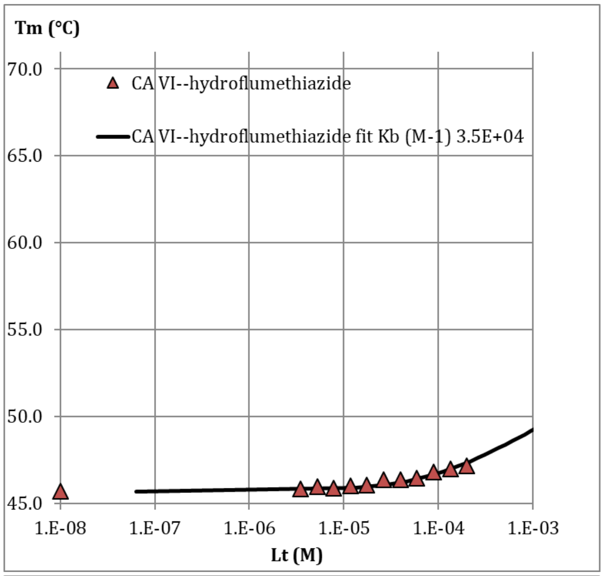
Tm (°C)
70.0
65.0
60.0
55.0
50.0
45.0
CA VI--hydroflumethiazide
CA VI--hydroflumethiazide fit Kb (M-1) 3.5E+04
1.E-08
1.E-07
1.E-06
1.E-05
1.E-04
1.E-03
Lt (M)

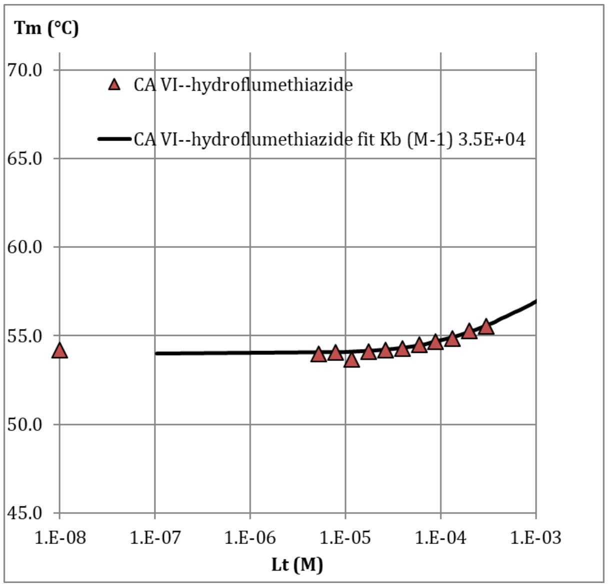
Tm (°C)
70.0
65.0
60.0
55.0
50.0
45.0
CA VI--hydroflumethiazide
CA VI--hydroflumethiazide fit Kb (M-1) 3.5E+04
1.E-08
1.E-07
1.E-06
1.E-05
1.E-04
1.E-03
Lt (M)

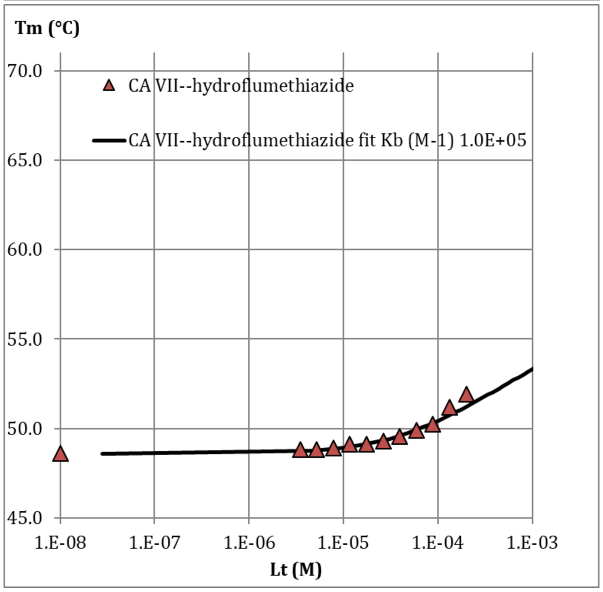
Tm (°C)
70.0
65.0
60.0
55.0
50.0
45.0
CA VII--hydroflumethiazide
CA VII--hydroflumethiazide fit Kb (M-1) 1.0E+05
1.E-08
1.E-07
1.E-06
1.E-05
1.E-04
1.E-03
Lt (M)

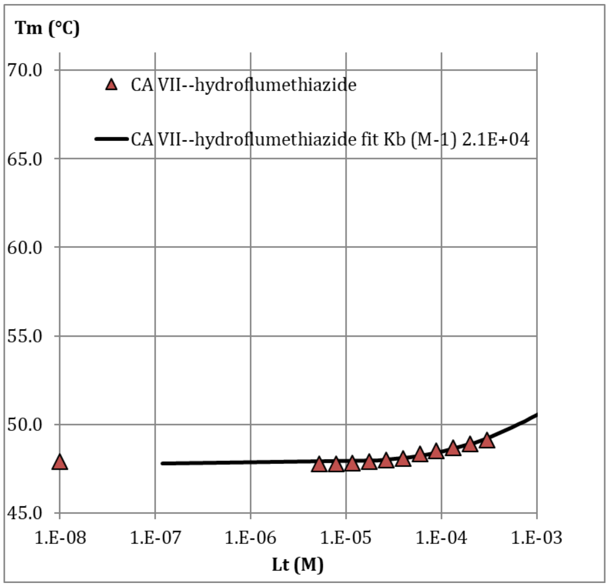
Tm (°C)
70.0
65.0
60.0
55.0
50.0
45.0
CA VII--hydroflumethiazide
CA VII--hydroflumethiazide fit Kb (M-1) 2.1E+04
1.E-08
1.E-07
1.E-06
1.E-05
1.E-04
1.E-03
Lt (M)

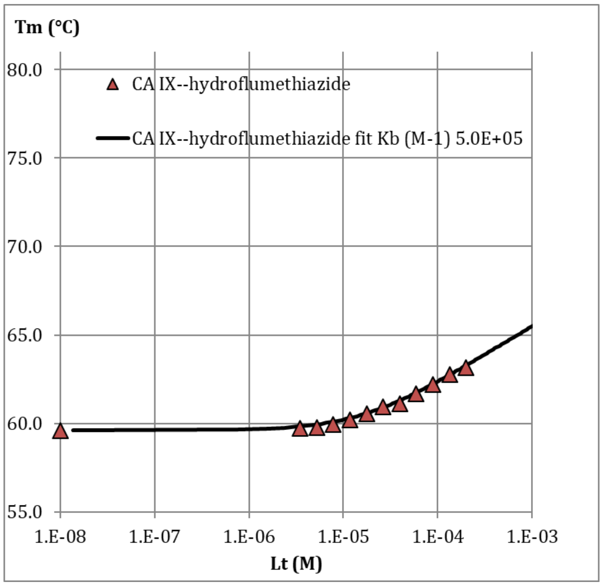
Tm (°C)
80.0
75.0
70.0
65.0
60.0
55.0
CA IX--hydroflumethiazide
CA IX--hydroflumethiazide fit Kb (M-1) 5.0E+05
1.E-08
1.E-07
1.E-06
1.E-05
1.E-04
1.E-03
Lt (M)

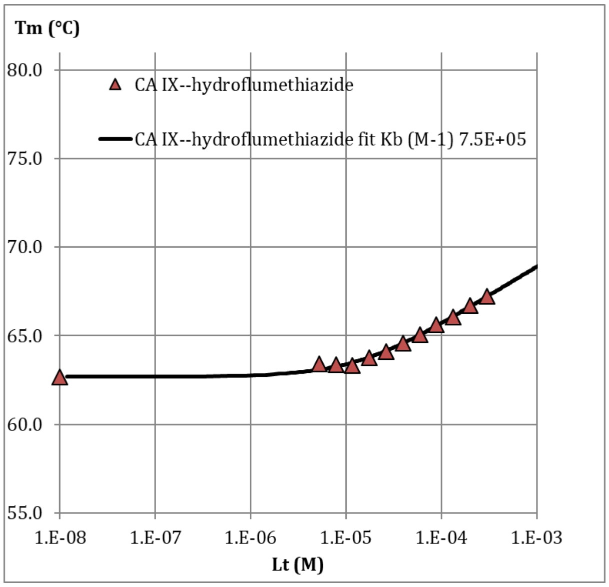
Tm (°C)
80.0
75.0
70.0
65.0
60.0
55.0
CA IX--hydroflumethiazide
CA IX--hydroflumethiazide fit Kb (M-1) 7.5E+05
1.E-08
1.E-07
1.E-06
1.E-05
1.E-04
1.E-03
Lt (M)

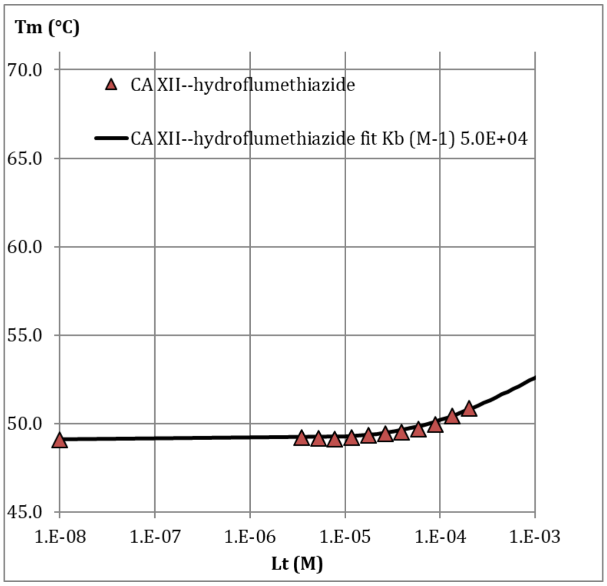

Tm (°C)
CA XII--hydroflumethiazide
CA XII--hydroflumethiazide fit Kb (M-1) 5.0E+04
Lt (M)

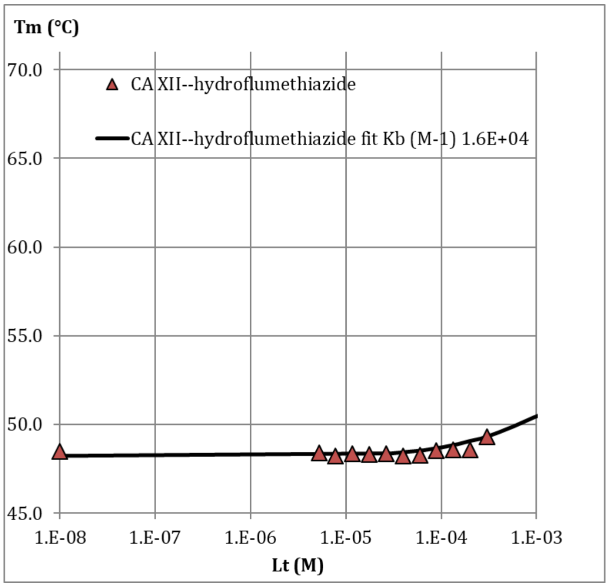

Tm (°C)
CA XII--hydroflumethiazide
CA XII--hydroflumethiazide fit Kb (M-1) 1.6E+04
Lt (M)

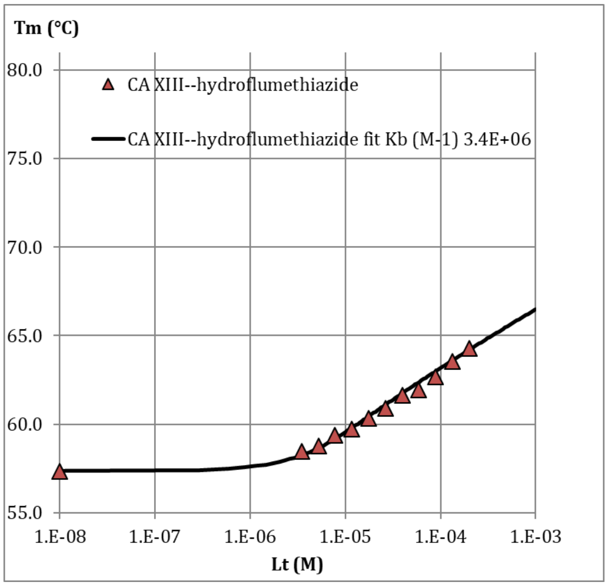

Tm (°C)
CA XIII--hydroflumethiazide
CA XIII--hydroflumethiazide fit Kb (M-1) 3.4E+06
Lt (M)

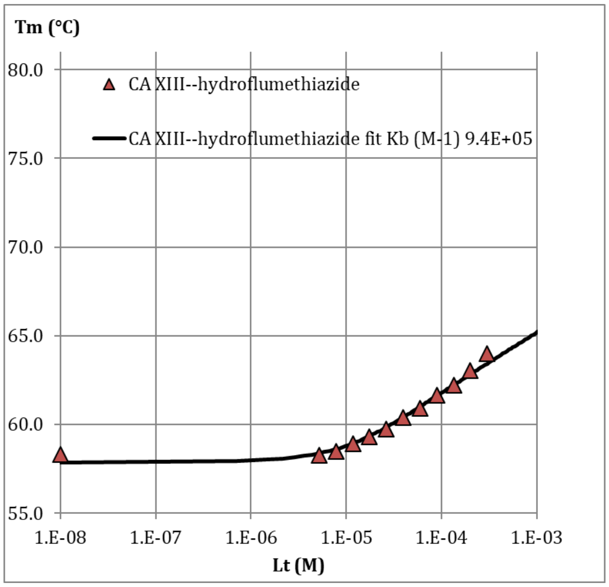

Tm (°C)
CA XIII--hydroflumethiazide
CA XIII--hydroflumethiazide fit Kb (M-1) 9.4E+05
Lt (M)

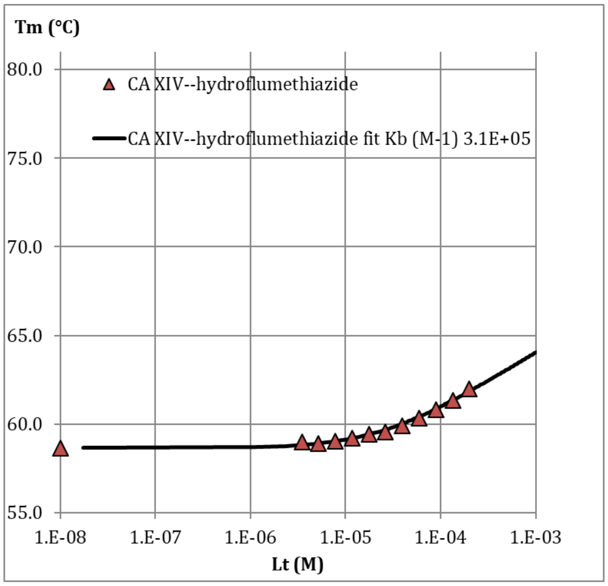

Tm (°C)
CA XIV--hydroflumethiazide
CA XIV--hydroflumethiazide fit Kb (M-1) 3.1E+05
Lt (M)

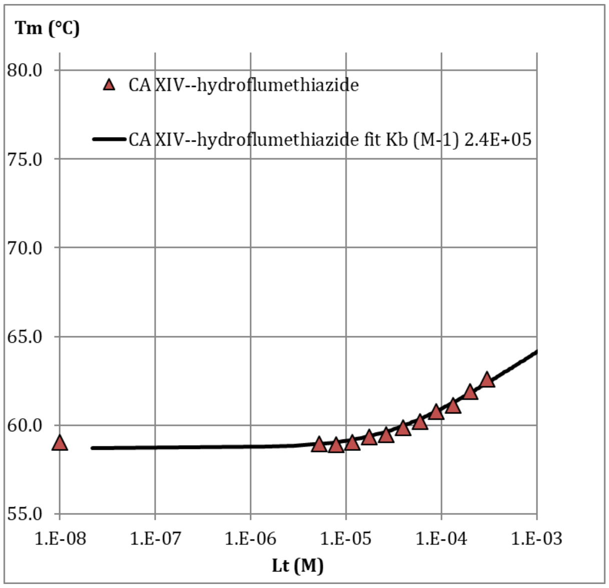

Tm (°C)
CA XIV--hydroflumethiazide
CA XIV--hydroflumethiazide fit Kb (M-1) 2.4E+05
Lt (M)

11. Bendroflumethiazide

Fluorecence-based thermal shift assay

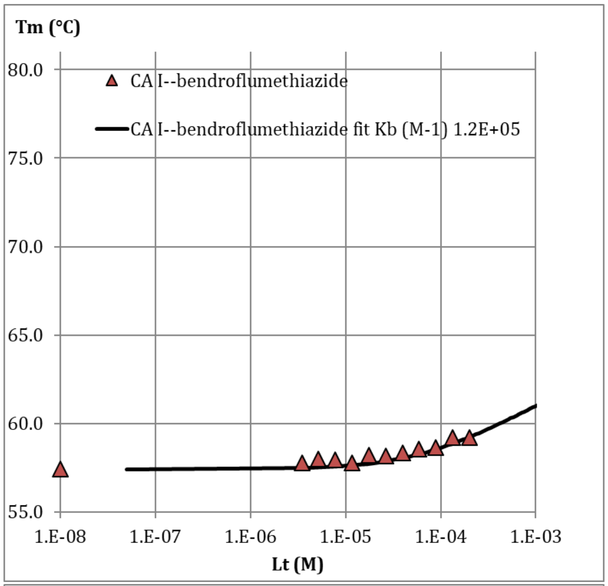

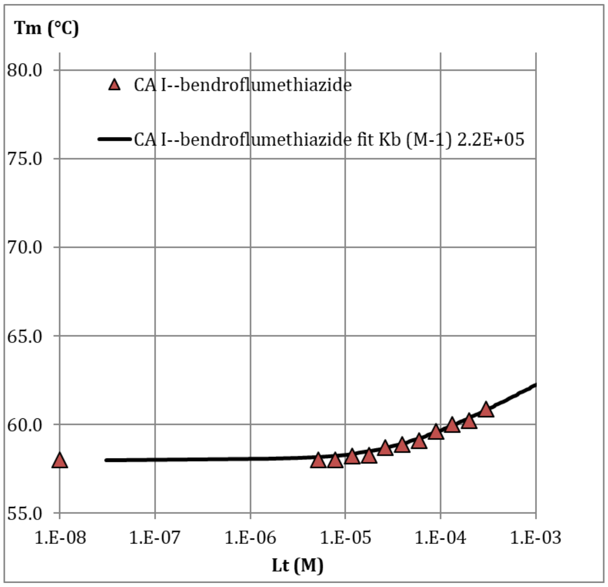

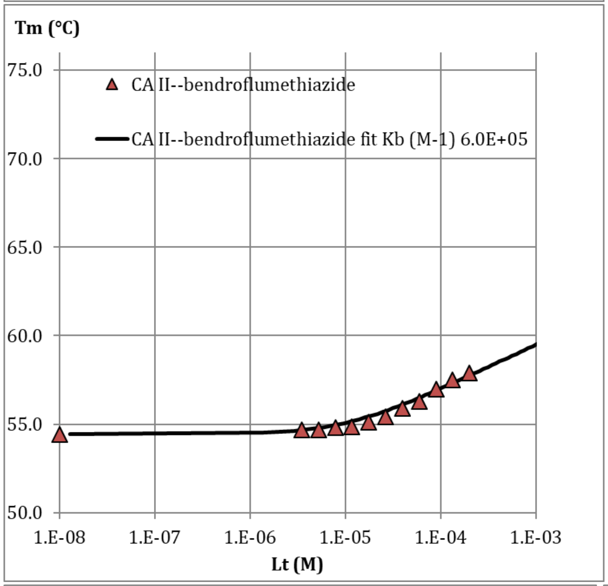

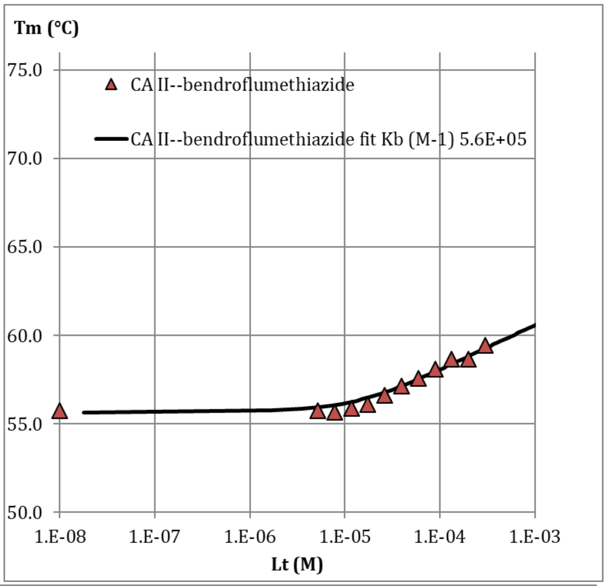

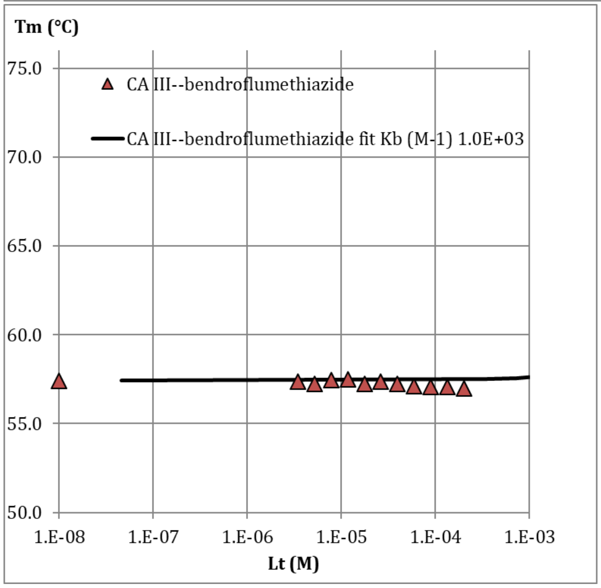

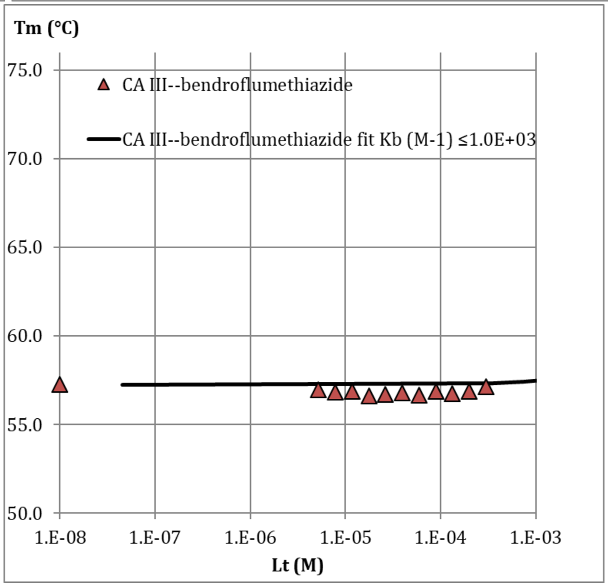

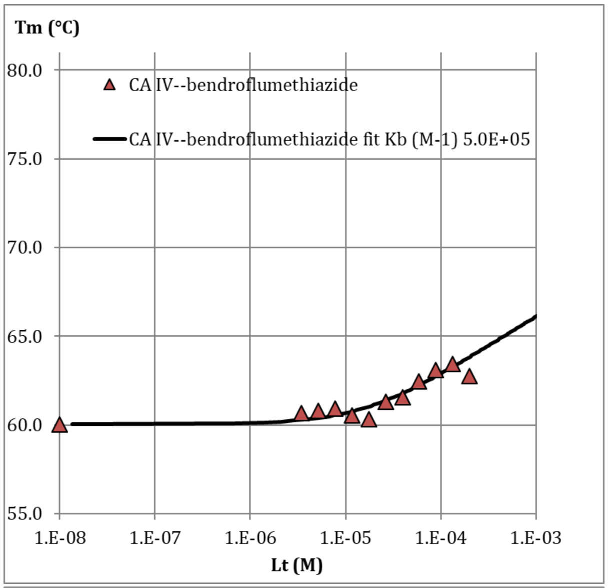
Tm (°C)
80.0
75.0
70.0
65.0
60.0
55.0
CA IV--bendroflumethiazide
CA IV--bendroflumethiazide fit Kb (M-1) 5.0E+05
1.E-08
1.E-07
1.E-06
1.E-05
1.E-04
1.E-03
Lt (M)

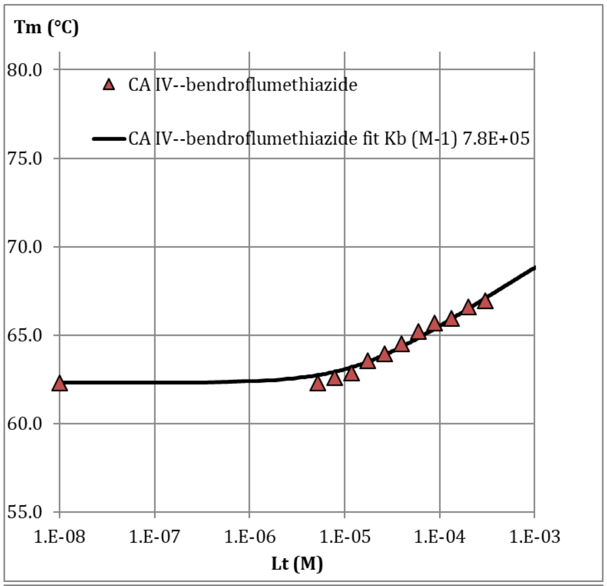
Tm (°C)
80.0
75.0
70.0
65.0
60.0
55.0
CA IV--bendroflumethiazide
CA IV--bendroflumethiazide fit Kb (M-1) 7.8E+05
1.E-08
1.E-07
1.E-06
1.E-05
1.E-04
1.E-03
Lt (M)

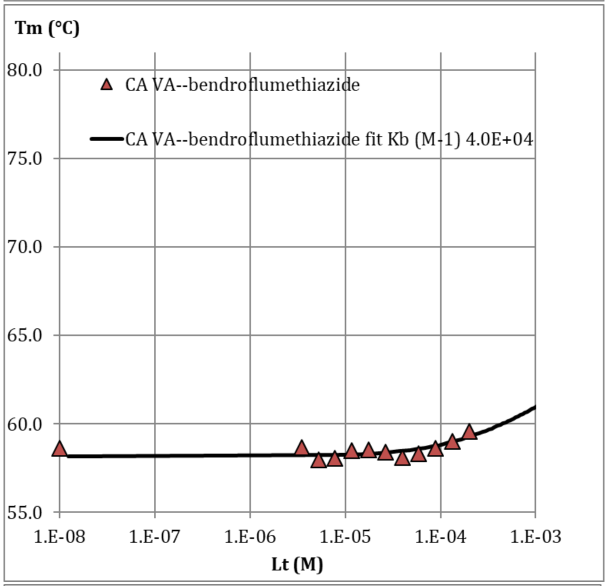
Tm (°C)
80.0
75.0
70.0
65.0
60.0
55.0
CA VA--bendroflumethiazide
CA VA--bendroflumethiazide fit Kb (M-1) 4.0E+04
1.E-08
1.E-07
1.E-06
1.E-05
1.E-04
1.E-03
Lt (M)

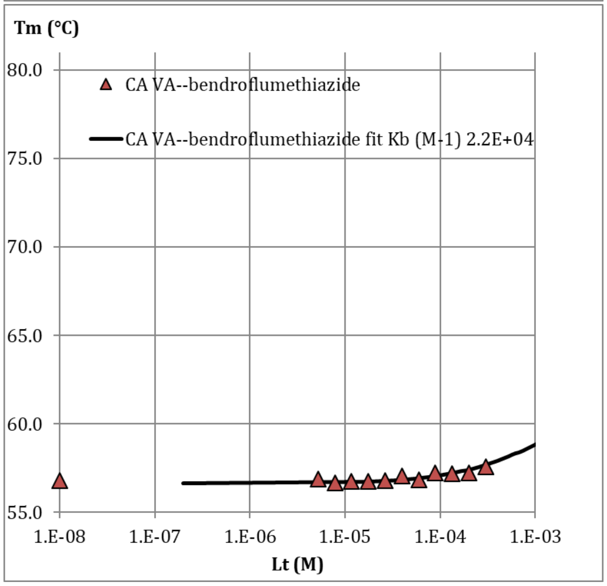
Tm (°C)
80.0
75.0
70.0
65.0
60.0
55.0
CA VA--bendroflumethiazide
CA VA--bendroflumethiazide fit Kb (M-1) 2.2E+04
1.E-08
1.E-07
1.E-06
1.E-05
1.E-04
1.E-03
Lt (M)

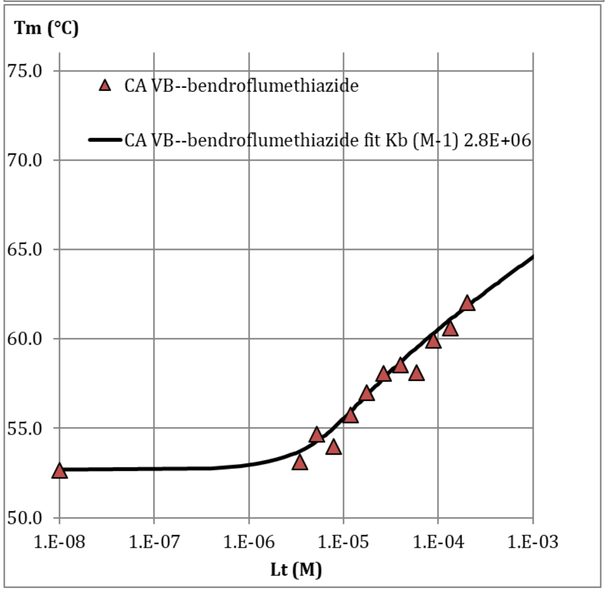
Tm (°C)
75.0
70.0
65.0
60.0
55.0
50.0
CA VB--bendroflumethiazide
CA VB--bendroflumethiazide fit Kb (M-1) 2.8E+06
1.E-08
1.E-07
1.E-06
1.E-05
1.E-04
1.E-03
Lt (M)

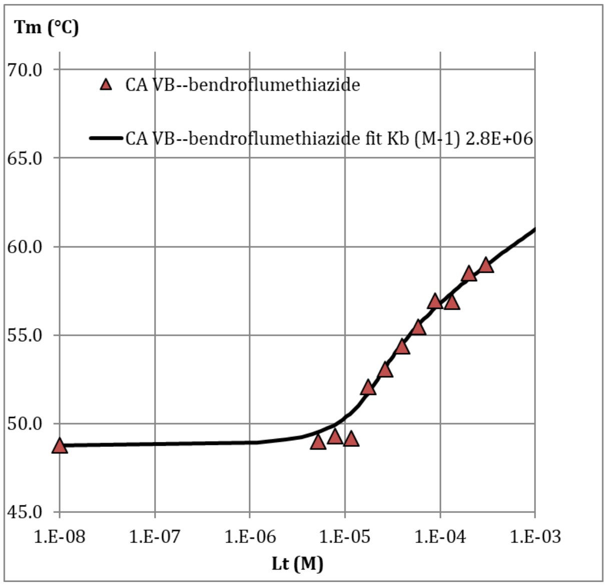
Tm (°C)
70.0
65.0
60.0
55.0
50.0
45.0
CA VB--bendroflumethiazide
CA VB--bendroflumethiazide fit Kb (M-1) 2.8E+06
1.E-08
1.E-07
1.E-06
1.E-05
1.E-04
1.E-03
Lt (M)

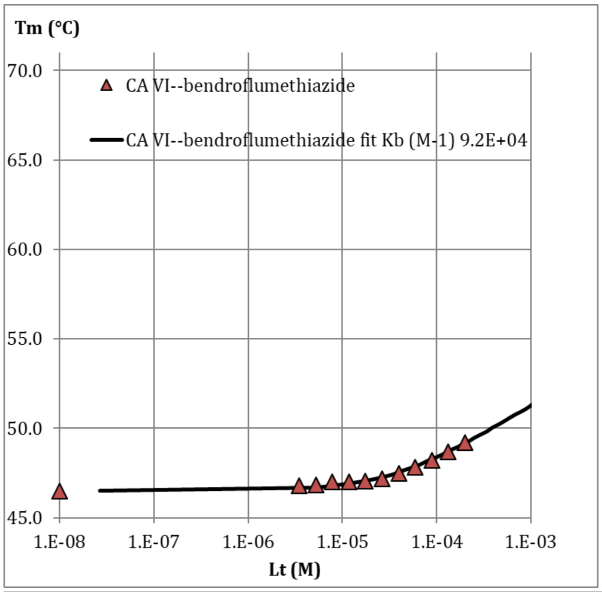
Tm (°C)
70.0
65.0
60.0
55.0
50.0
45.0
CA VI--bendroflumethiazide
CA VI--bendroflumethiazide fit Kb (M-1) 9.2E+04
1.E-08
1.E-07
1.E-06
1.E-05
1.E-04
1.E-03
Lt (M)

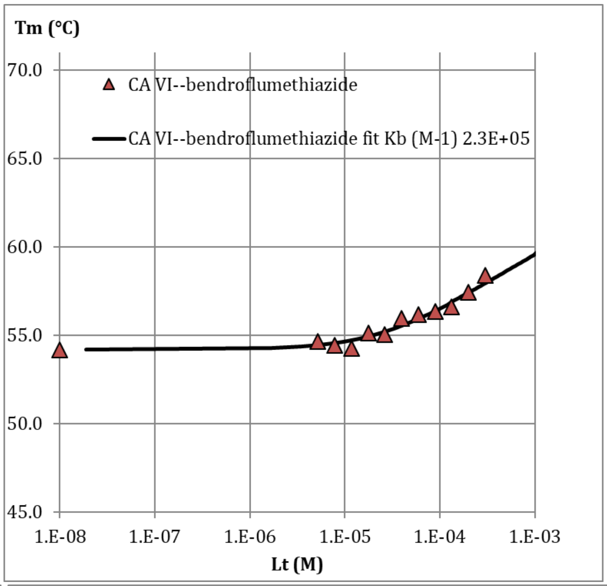
Tm (°C)
70.0
65.0
60.0
55.0
50.0
45.0
CA VI--bendroflumethiazide
CA VI--bendroflumethiazide fit Kb (M-1) 2.3E+05
1.E-08
1.E-07
1.E-06
1.E-05
1.E-04
1.E-03
Lt (M)

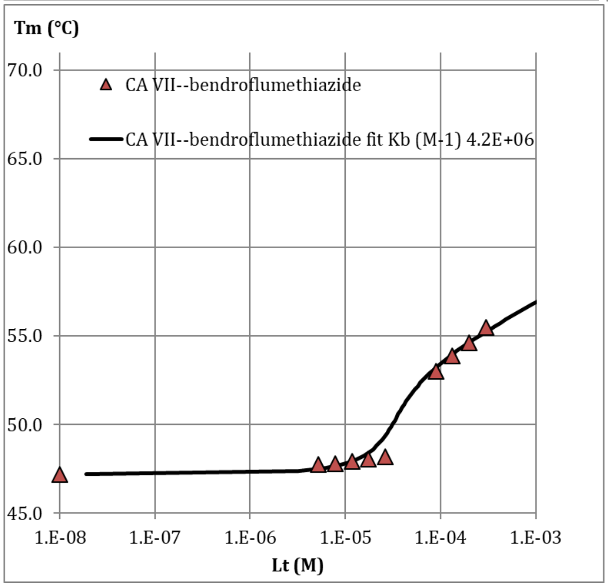
Tm (°C)
70.0
65.0
60.0
55.0
50.0
45.0
CA VII--bendroflumethiazide
CA VII--bendroflumethiazide fit Kb (M-1) 4.2E+06
1.E-08
1.E-07
1.E-06
1.E-05
1.E-04
1.E-03
Lt (M)

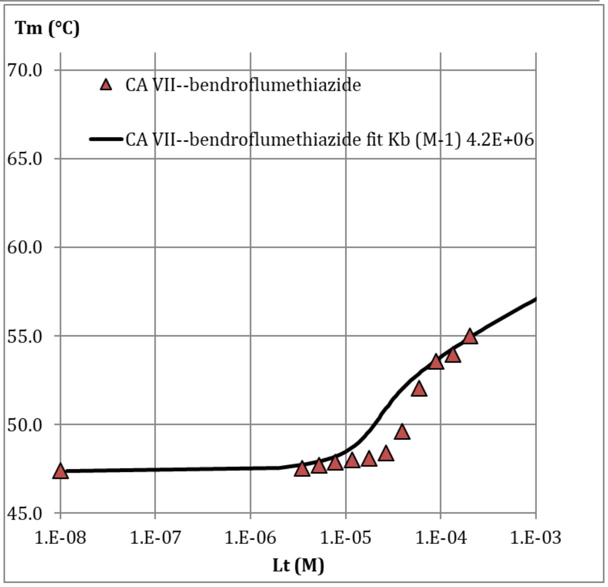
Tm (°C)
70.0
65.0
60.0
55.0
50.0
45.0
CA VII--bendroflumethiazide
CA VII--bendroflumethiazide fit Kb (M-1) 4.2E+06
1.E-08
1.E-07
1.E-06
1.E-05
1.E-04
1.E-03
Lt (M)

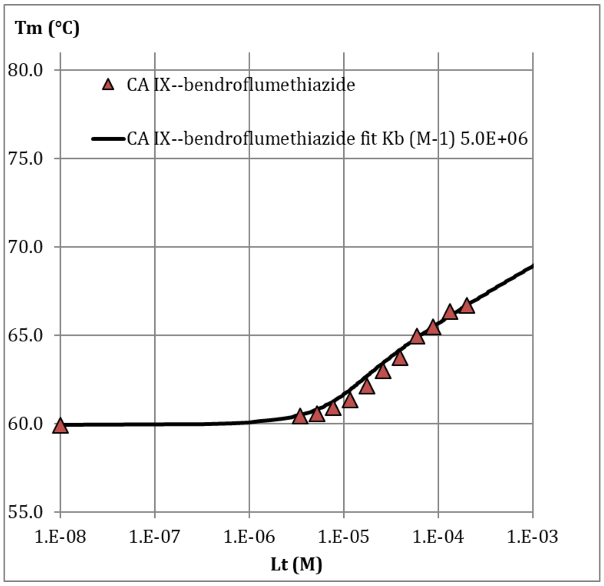
Tm (°C)
80.0
75.0
70.0
65.0
60.0
55.0
CA IX--bendroflumethiazide
CA IX--bendroflumethiazide fit Kb (M-1) 5.0E+06
1.E-08
1.E-07
1.E-06
1.E-05
1.E-04
1.E-03
Lt (M)

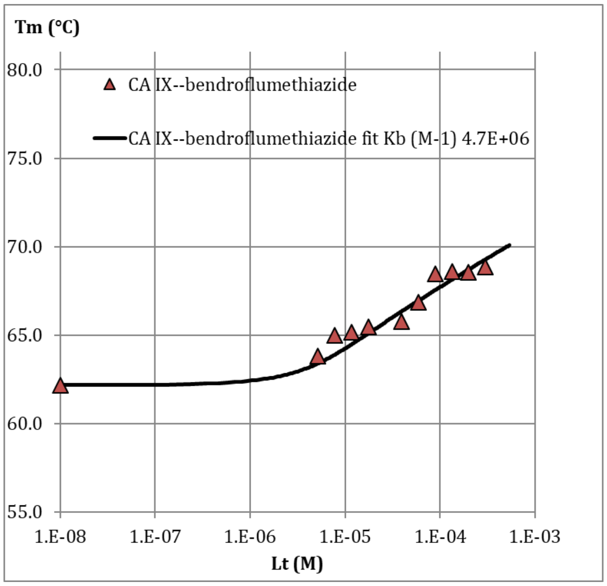
Tm (°C)
80.0
75.0
70.0
65.0
60.0
55.0
CA IX--bendroflumethiazide
CA IX--bendroflumethiazide fit Kb (M-1) 4.7E+06
1.E-08
1.E-07
1.E-06
1.E-05
1.E-04
1.E-03
Lt (M)

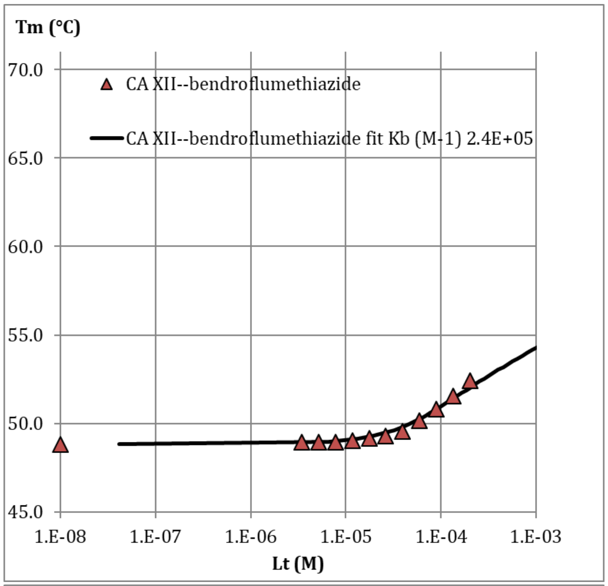
Tm (°C)
CA XII--bendroflumethiazide
CA XII--bendroflumethiazide fit Kb (M-1) 2.4E+05
70.0
65.0
60.0
55.0
50.0
45.0
1.E-08
1.E-07
1.E-06
1.E-05
1.E-04
1.E-03
Lt (M)

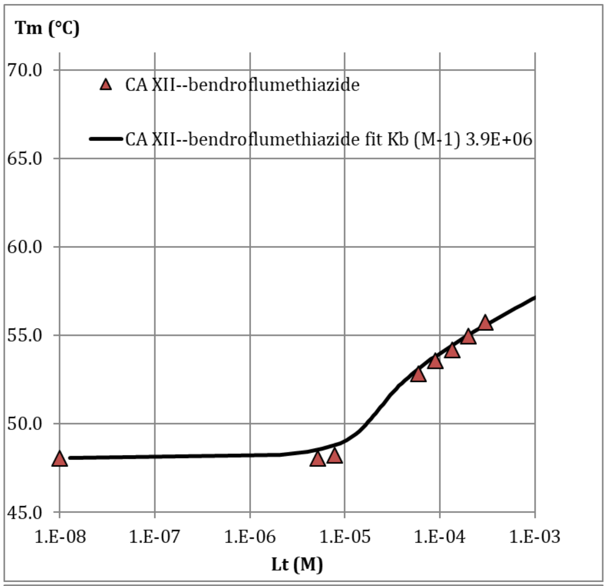
Tm (°C)
CA XII--bendroflumethiazide
CA XII--bendroflumethiazide fit Kb (M-1) 3.9E+06
70.0
65.0
60.0
55.0
50.0
45.0
1.E-08
1.E-07
1.E-06
1.E-05
1.E-04
1.E-03
Lt (M)

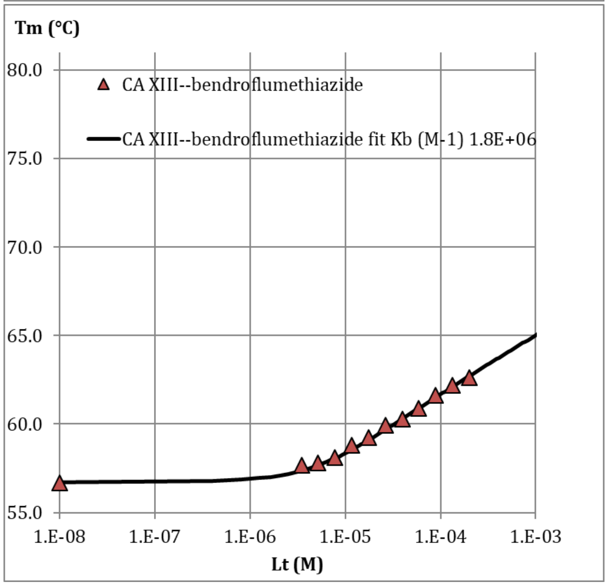
Tm (°C)
CA XIII--bendroflumethiazide
CA XIII--bendroflumethiazide fit Kb (M-1) 1.8E+06
80.0
75.0
70.0
65.0
60.0
55.0
1.E-08
1.E-07
1.E-06
1.E-05
1.E-04
1.E-03
Lt (M)

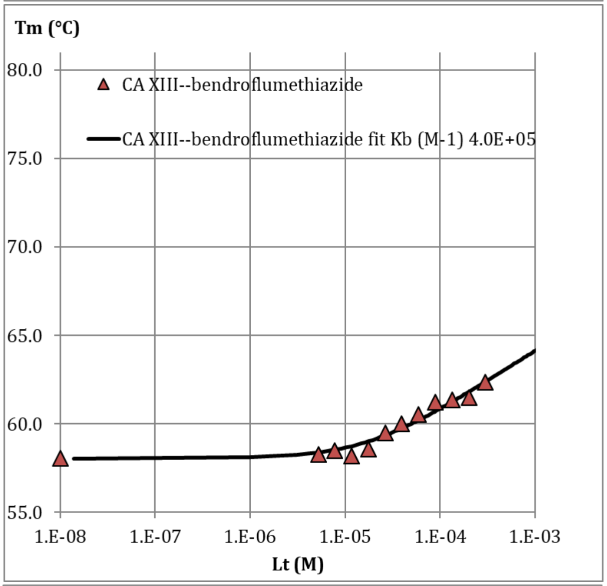
Tm (°C)
CA XIII--bendroflumethiazide
CA XIII--bendroflumethiazide fit Kb (M-1) 4.0E+05
80.0
75.0
70.0
65.0
60.0
55.0
1.E-08
1.E-07
1.E-06
1.E-05
1.E-04
1.E-03
Lt (M)

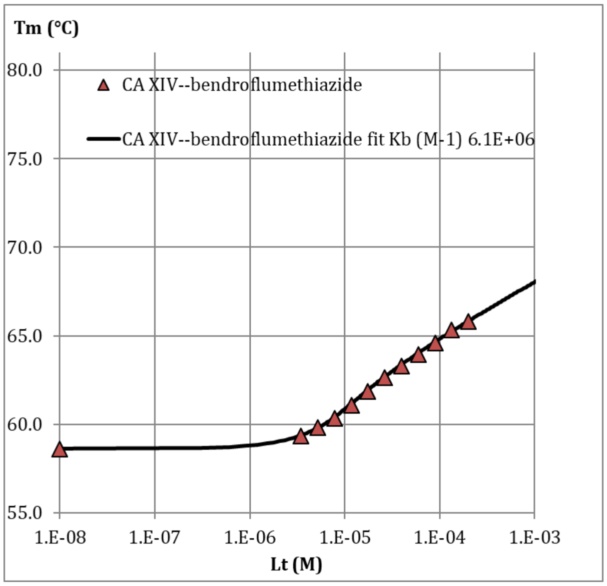
Tm (°C)
CA XIV--bendroflumethiazide
CA XIV--bendroflumethiazide fit Kb (M-1) 6.1E+06
80.0
75.0
70.0
65.0
60.0
55.0
1.E-08
1.E-07
1.E-06
1.E-05
1.E-04
1.E-03
Lt (M)

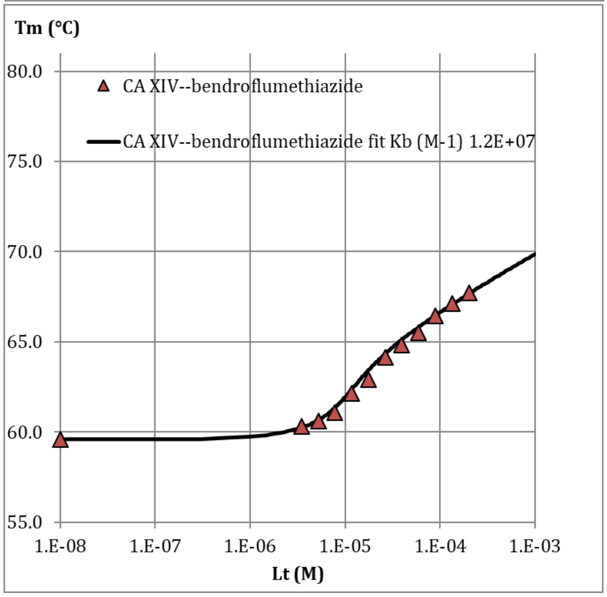
Tm (°C)
CA XIV--bendroflumethiazide
CA XIV--bendroflumethiazide fit Kb (M-1) 1.2E+07
80.0
75.0
70.0
65.0
60.0
55.0
1.E-08
1.E-07
1.E-06
1.E-05
1.E-04
1.E-03
Lt (M)

CO_2 hydration activity inhibition

CA IV - bendroflumethiazide

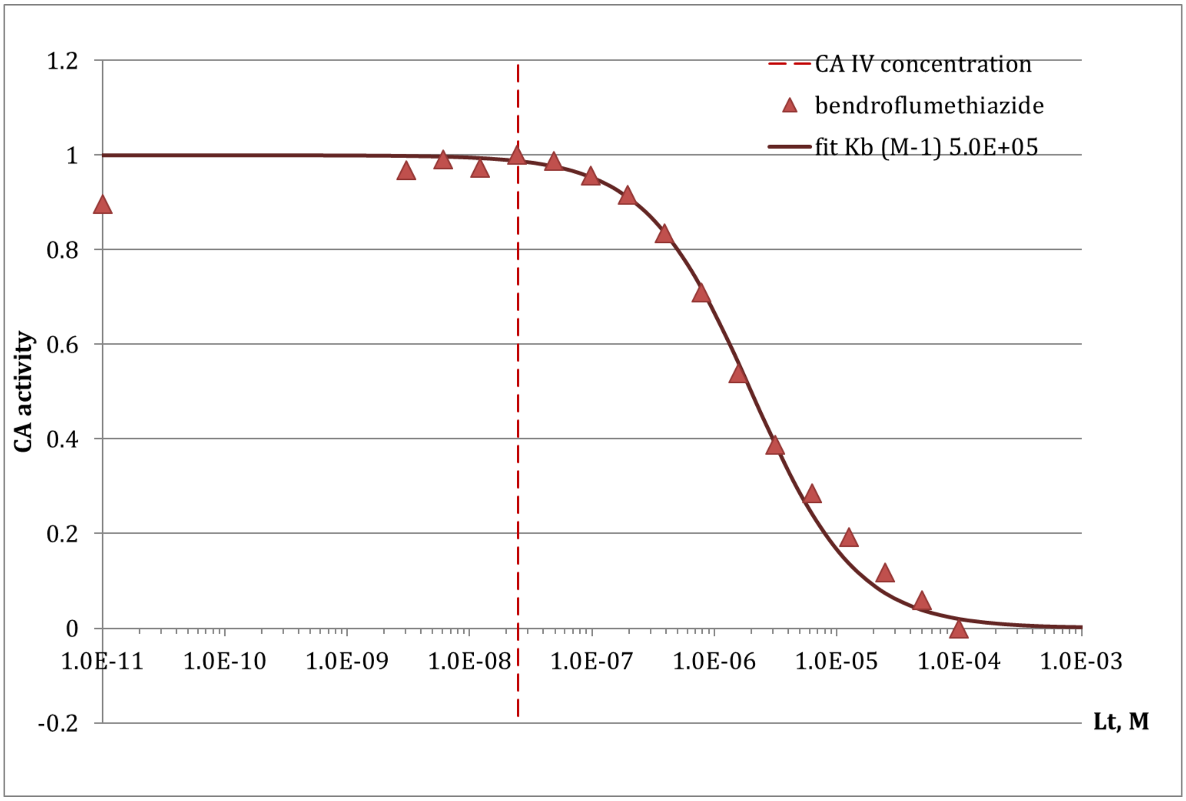

12. Indapamide

Fluorecence-based thermal shift assay

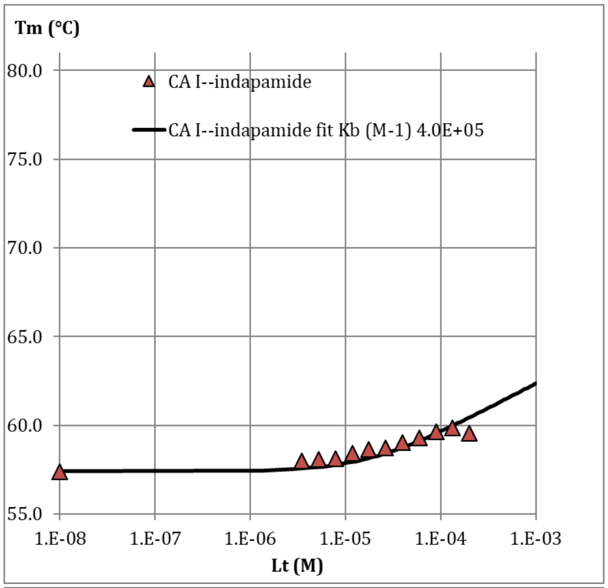

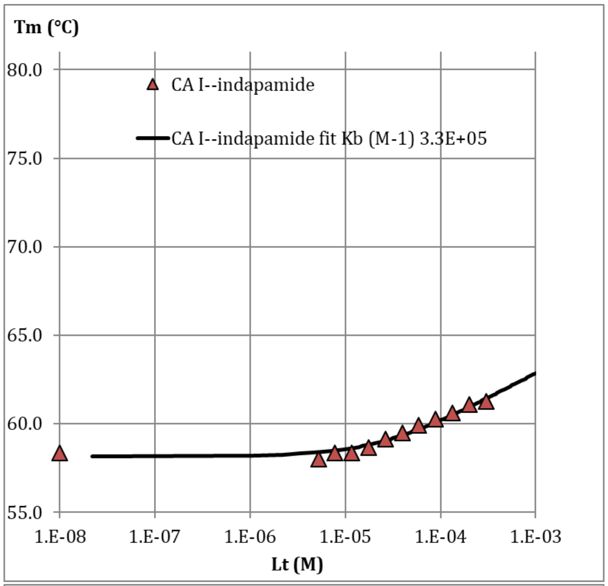

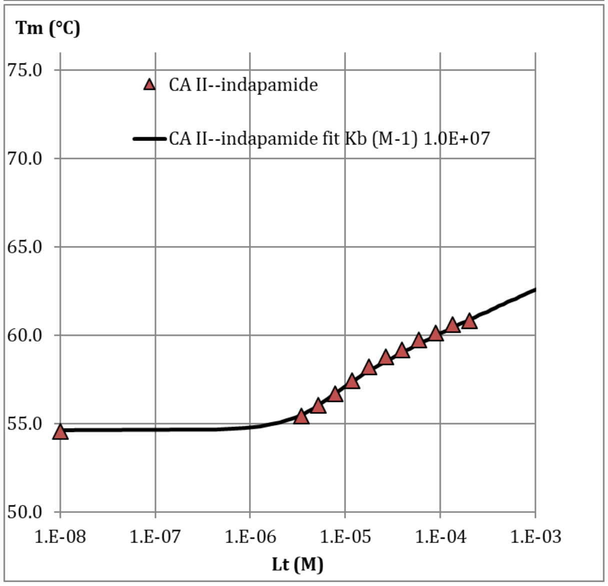

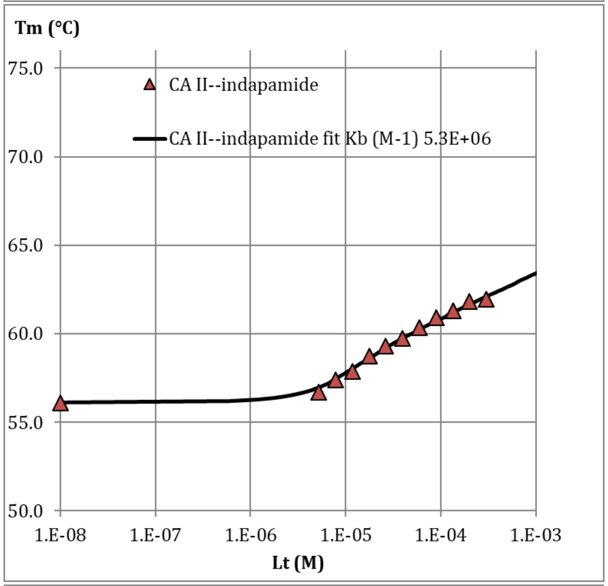

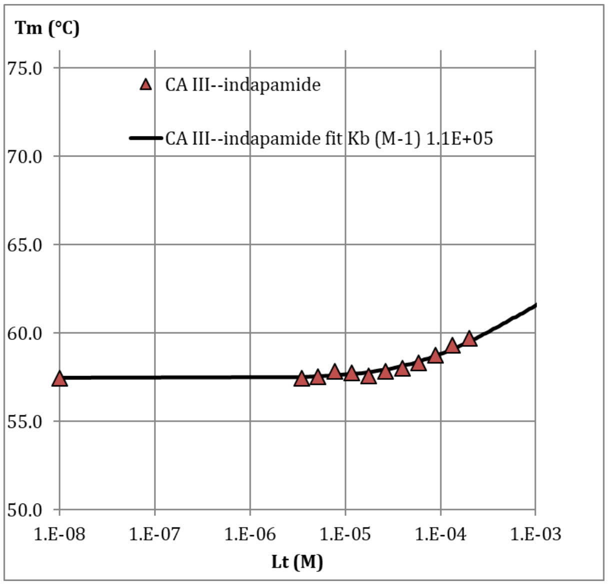

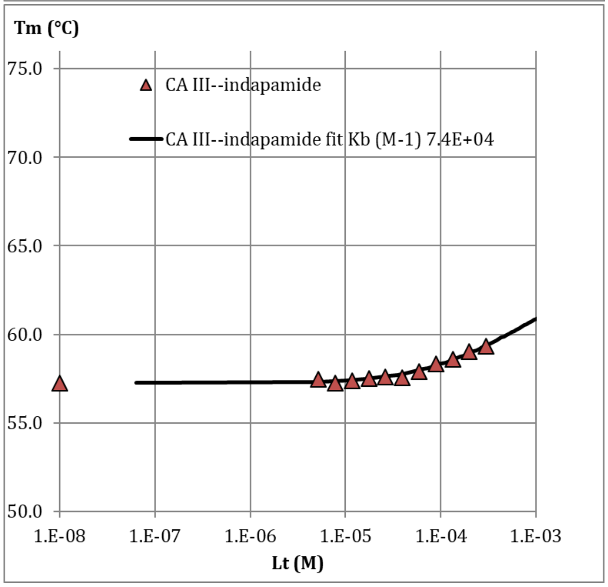

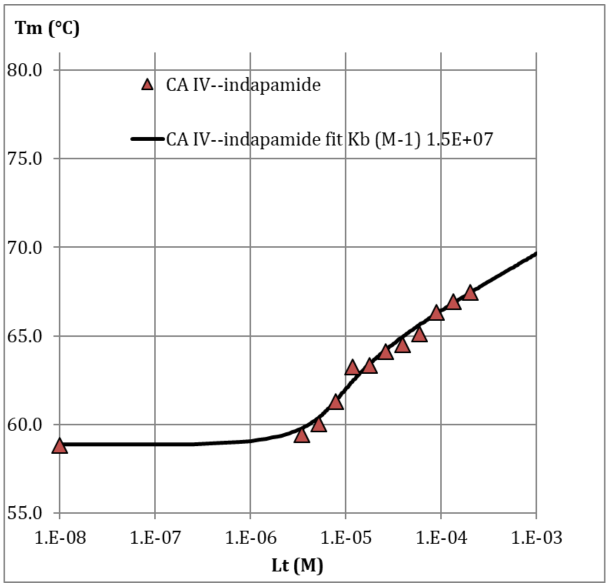
Tm (°C)
CA IV--indapamide
CA IV--indapamide fit Kb (M-1) 1.5E+07
80.0
75.0
70.0
65.0
60.0
55.0
1.E-08
1.E-07
1.E-06
1.E-05
1.E-04
1.E-03
Lt (M)

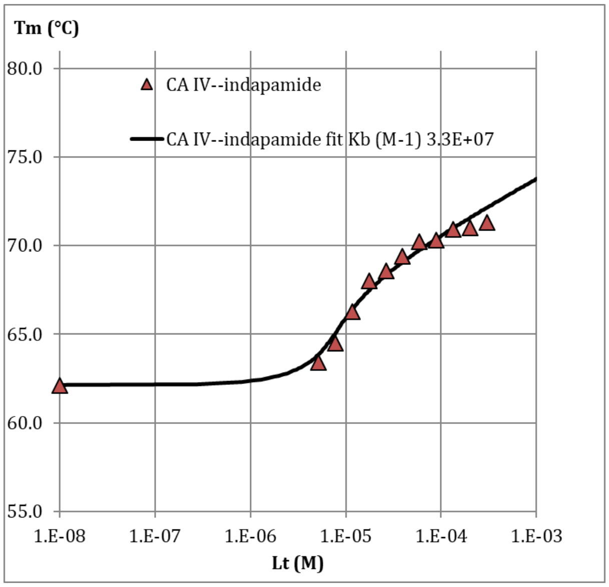
Tm (°C)
CA IV--indapamide
CA IV--indapamide fit Kb (M-1) 3.3E+07
80.0
75.0
70.0
65.0
60.0
55.0
1.E-08
1.E-07
1.E-06
1.E-05
1.E-04
1.E-03
Lt (M)

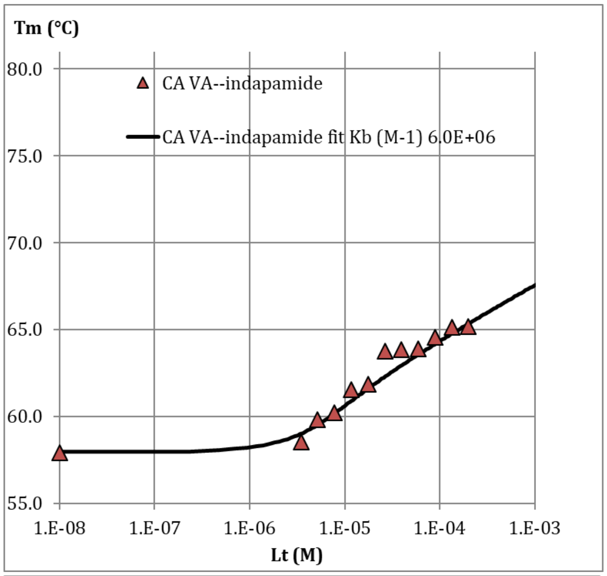
Tm (°C)
CA VA--indapamide
CA VA--indapamide fit Kb (M-1) 6.0E+06
80.0
75.0
70.0
65.0
60.0
55.0
1.E-08
1.E-07
1.E-06
1.E-05
1.E-04
1.E-03
Lt (M)

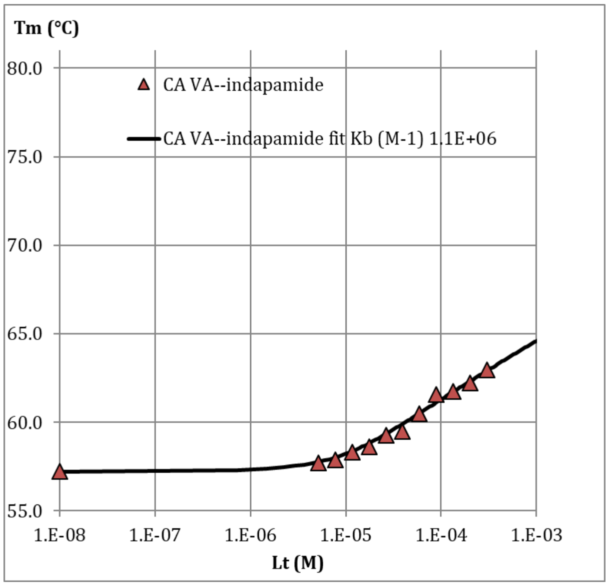
Tm (°C)
CA VA--indapamide
CA VA--indapamide fit Kb (M-1) 1.1E+06
80.0
75.0
70.0
65.0
60.0
55.0
1.E-08
1.E-07
1.E-06
1.E-05
1.E-04
1.E-03
Lt (M)

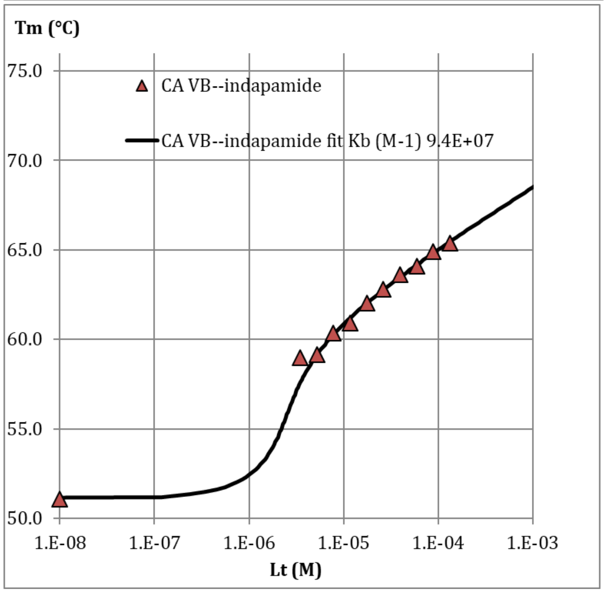
Tm (°C)
CA VB--indapamide
CA VB--indapamide fit Kb (M-1) 9.4E+07
75.0
70.0
65.0
60.0
55.0
50.0
1.E-08
1.E-07
1.E-06
1.E-05
1.E-04
1.E-03
Lt (M)

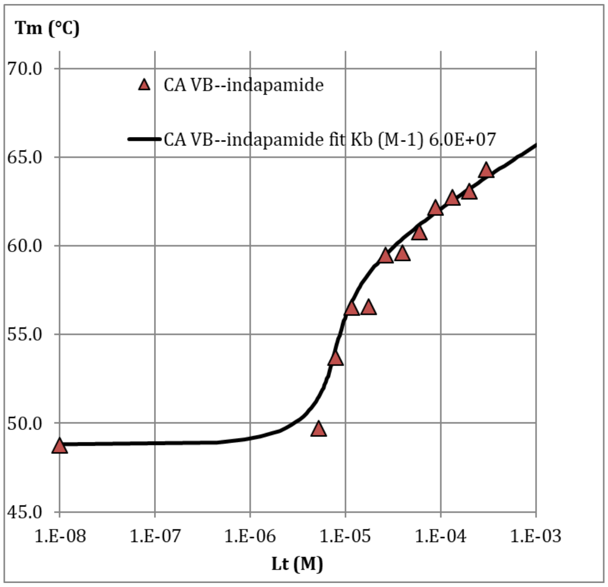
Tm (°C)
CA VB--indapamide
CA VB--indapamide fit Kb (M-1) 6.0E+07
70.0
65.0
60.0
55.0
50.0
45.0
1.E-08
1.E-07
1.E-06
1.E-05
1.E-04
1.E-03
Lt (M)

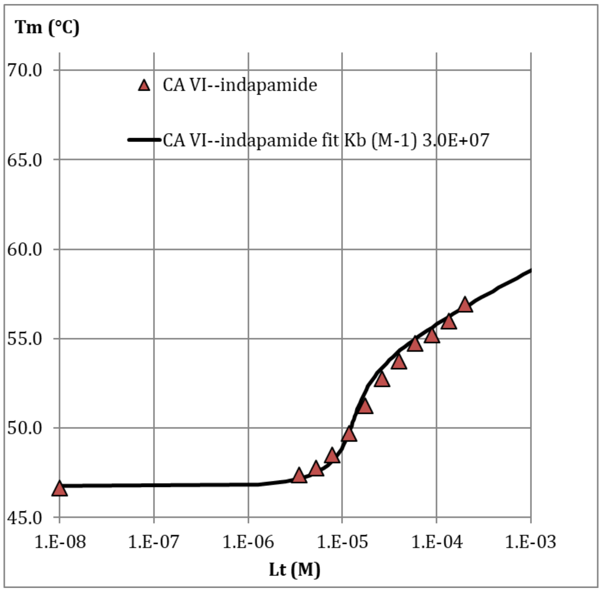
Tm (°C)
CA VI--indapamide
CA VI--indapamide fit Kb (M-1) 3.0E+07
Lt (M)

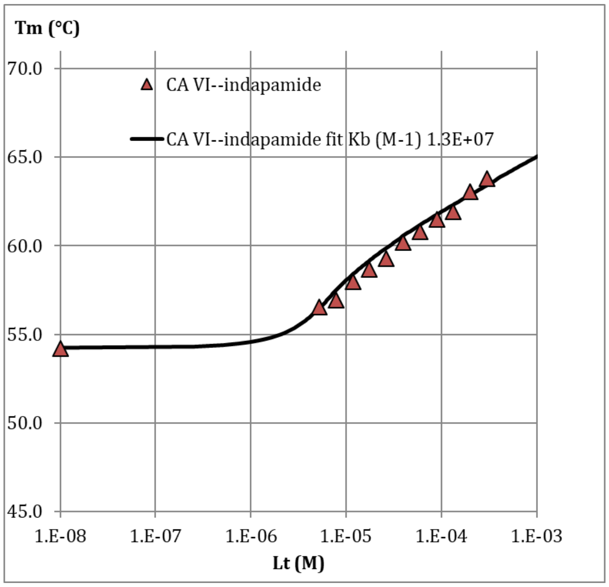
Tm (°C)
CA VI--indapamide
CA VI--indapamide fit Kb (M-1) 1.3E+07
Lt (M)

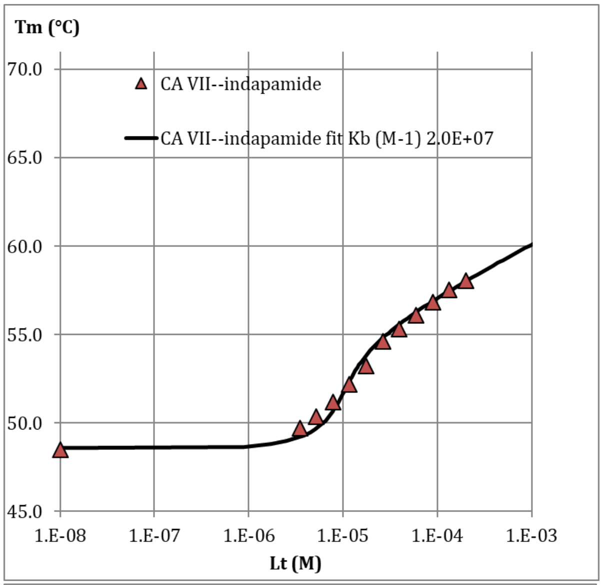
Tm (°C)
CA VII--indapamide
CA VII--indapamide fit Kb (M-1) 2.0E+07
Lt (M)

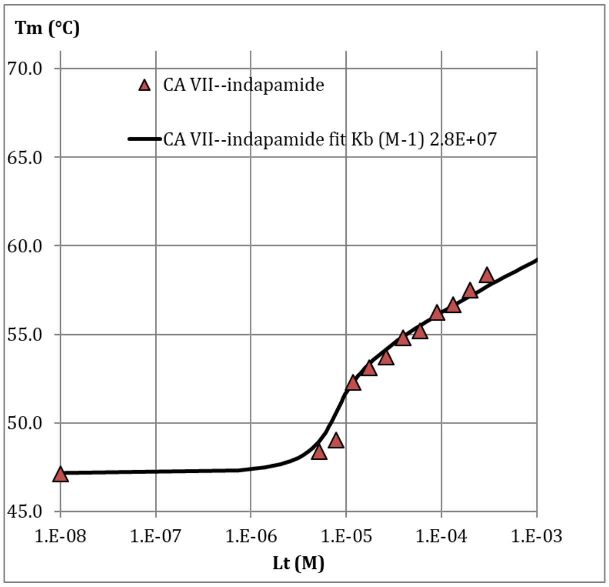
Tm (°C)
CA VII--indapamide
CA VII--indapamide fit Kb (M-1) 2.8E+07
Lt (M)

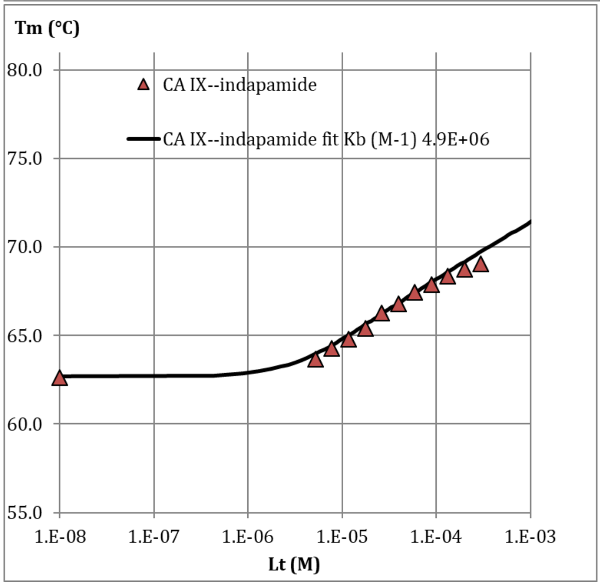
Tm (°C)
CA IX--indapamide
CA IX--indapamide fit Kb (M-1) 4.9E+06
Lt (M)

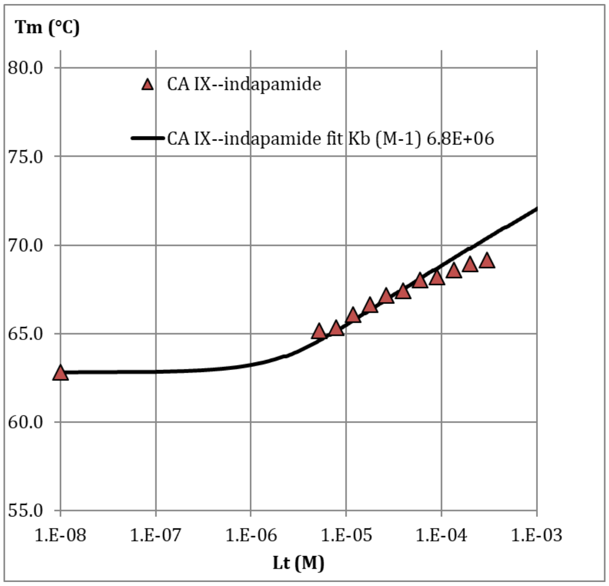
Tm (°C)
CA IX--indapamide
CA IX--indapamide fit Kb (M-1) 6.8E+06
Lt (M)

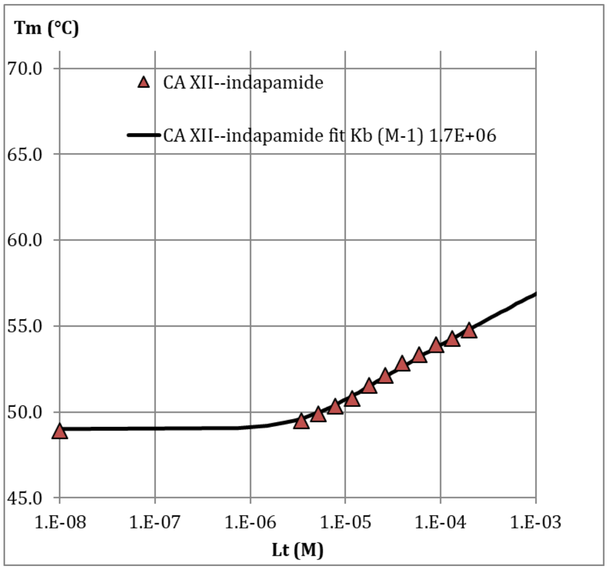

Tm (°C)
70.0
65.0
60.0
55.0
50.0
45.0
CA XII--indapamide
CA XII--indapamide fit Kb (M-1) 1.7E+06
1.E-08
1.E-07
1.E-06
1.E-05
1.E-04
1.E-03
Lt (M)

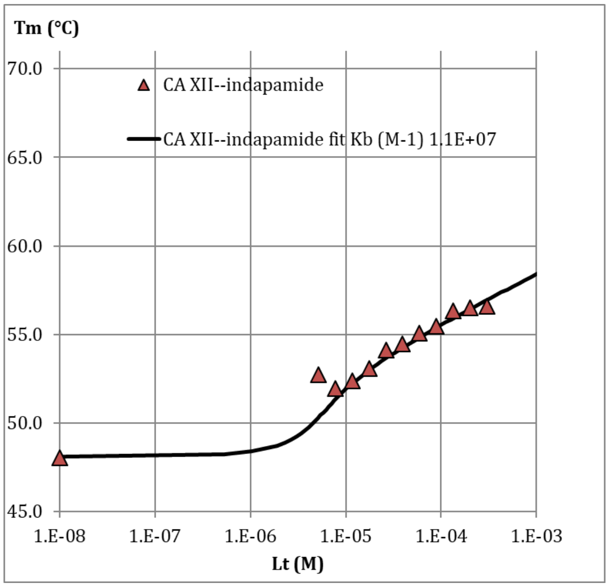

Tm (°C)
70.0
65.0
60.0
55.0
50.0
45.0
CA XII--indapamide
CA XII--indapamide fit Kb (M-1) 1.1E+07
1.E-08
1.E-07
1.E-06
1.E-05
1.E-04
1.E-03
Lt (M)

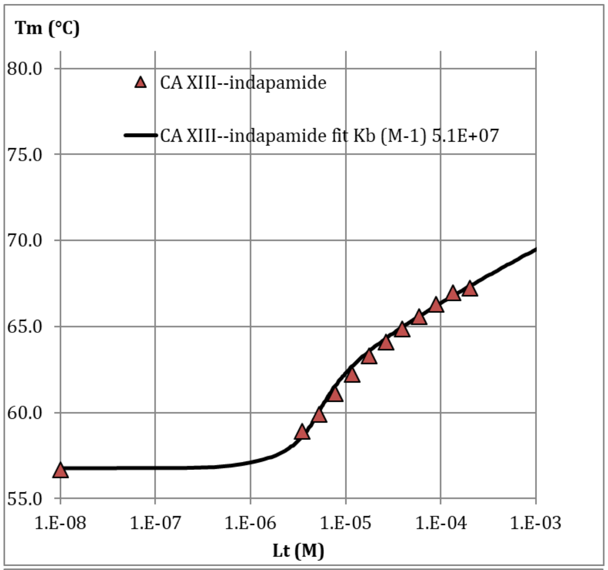

Tm (°C)
80.0
75.0
70.0
65.0
60.0
55.0
CA XIII--indapamide
CA XIII--indapamide fit Kb (M-1) 5.1E+07
1.E-08
1.E-07
1.E-06
1.E-05
1.E-04
1.E-03
Lt (M)

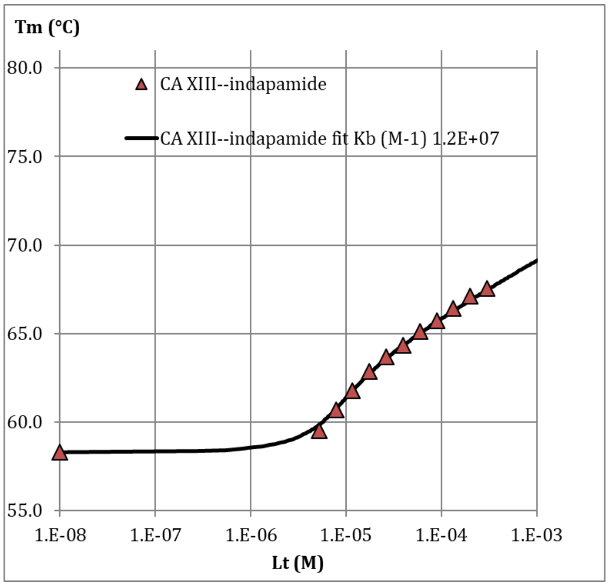

Tm (°C)
80.0
75.0
70.0
65.0
60.0
55.0
CA XIII--indapamide
CA XIII--indapamide fit Kb (M-1) 1.2E+07
1.E-08
1.E-07
1.E-06
1.E-05
1.E-04
1.E-03
Lt (M)

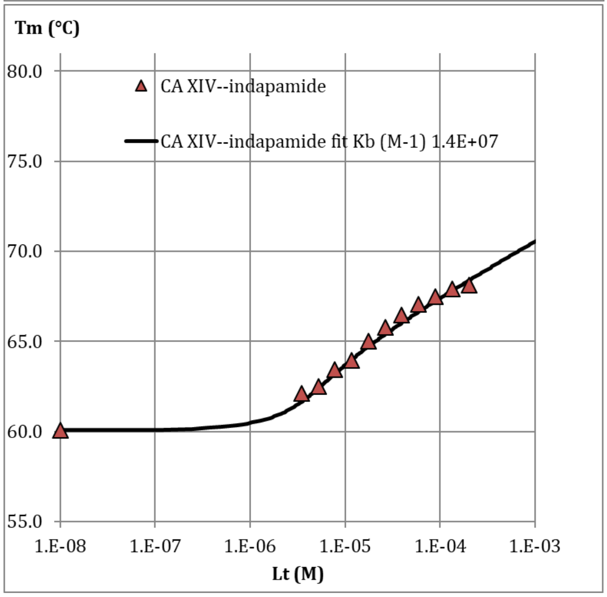

Tm (°C)
80.0
75.0
70.0
65.0
60.0
55.0
CA XIV--indapamide
CA XIV--indapamide fit Kb (M-1) 1.4E+07
1.E-08
1.E-07
1.E-06
1.E-05
1.E-04
1.E-03
Lt (M)

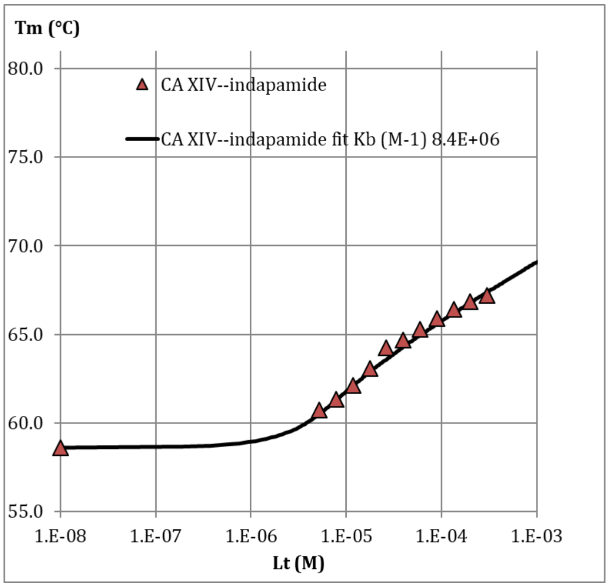

Tm (°C)
80.0
75.0
70.0
65.0
60.0
55.0
CA XIV--indapamide
CA XIV--indapamide fit Kb (M-1) 8.4E+06
1.E-08
1.E-07
1.E-06
1.E-05
1.E-04
1.E-03
Lt (M)

CO_2 hydration activity inhibition

CA IV - indapamide

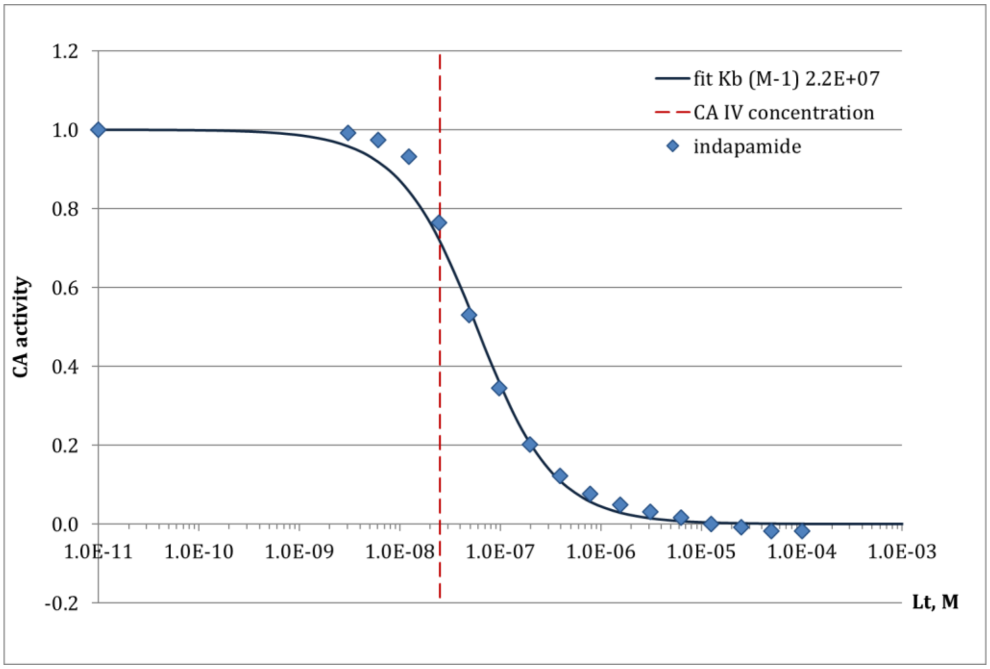

CA XII - indapamide

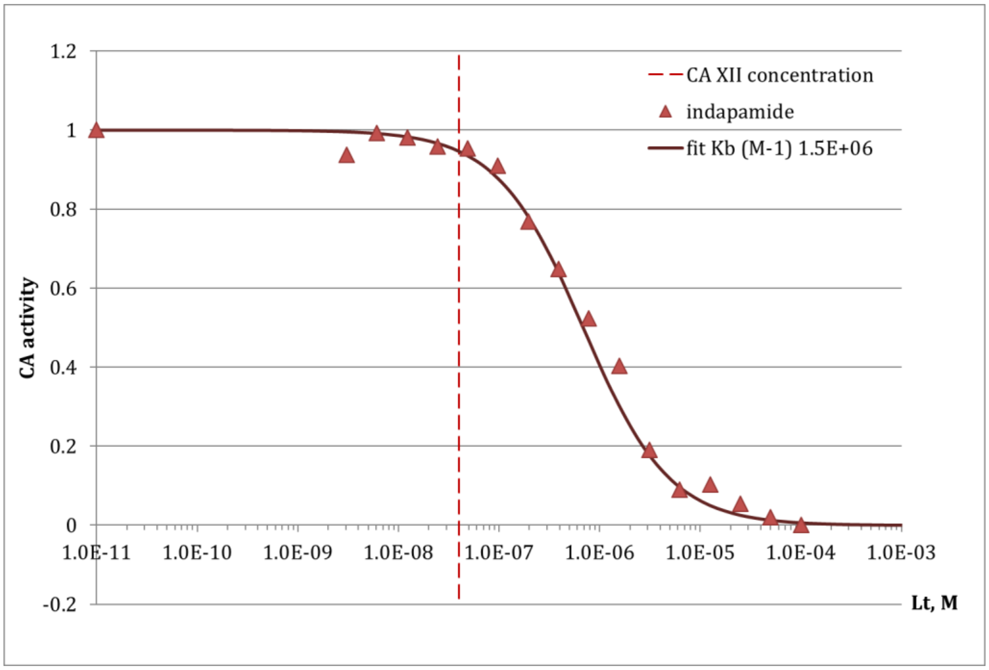

Isothermal titration calorimetry

CA II – indapamide

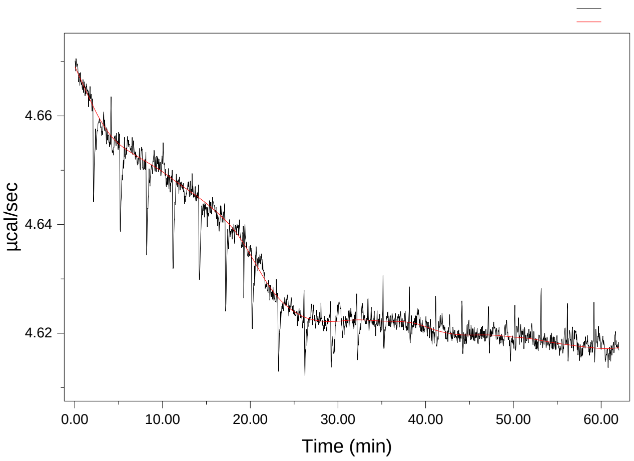

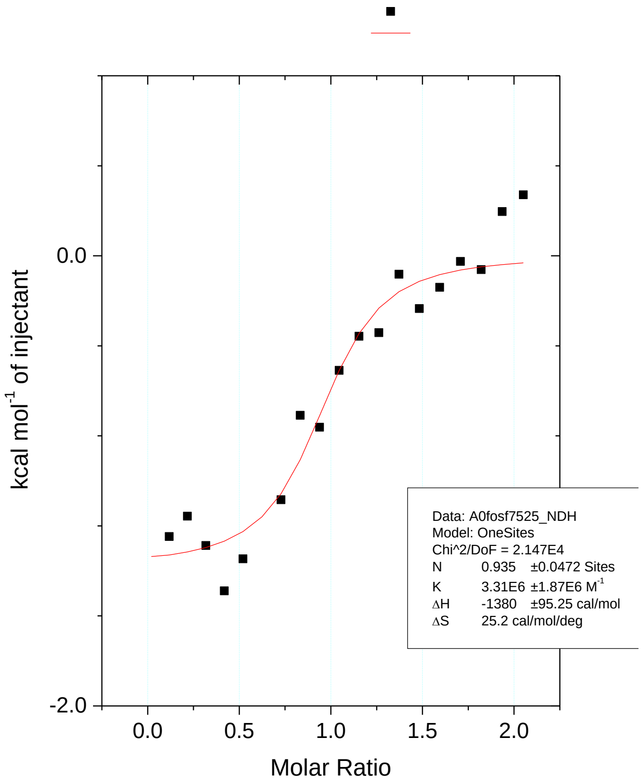

CA II – indapamide

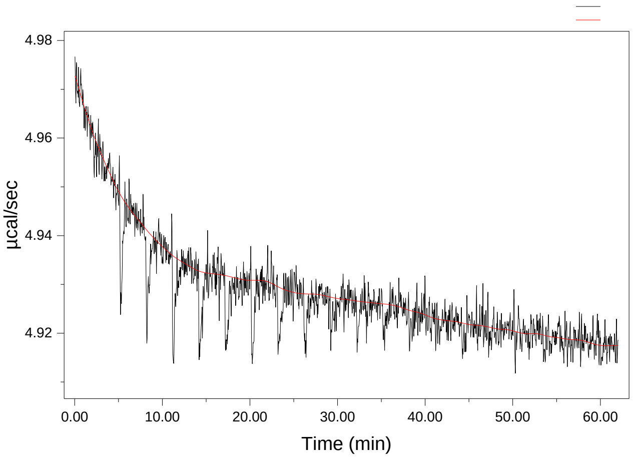

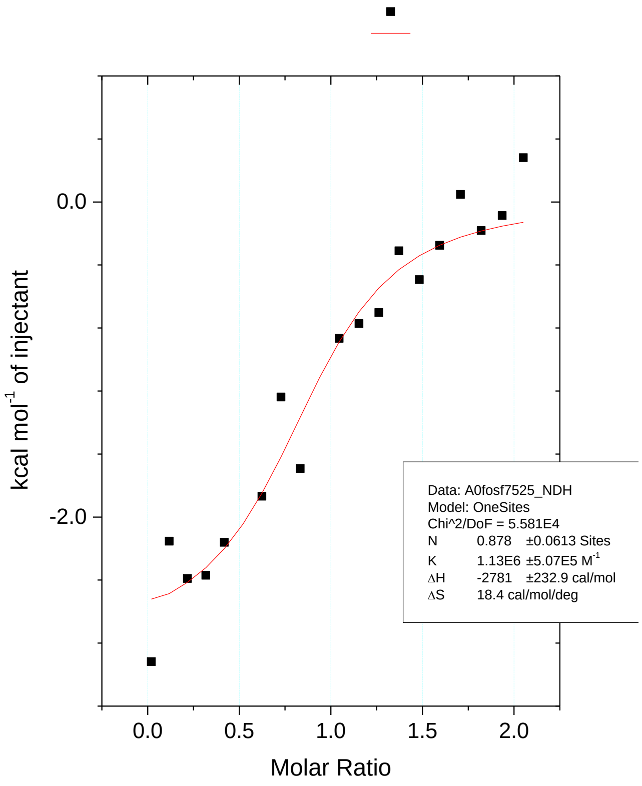

13. Clopamide

Fluorecence-based thermal shift assay

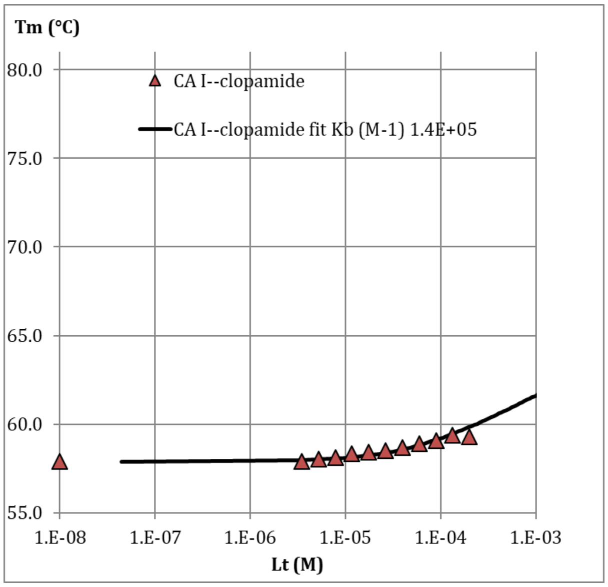

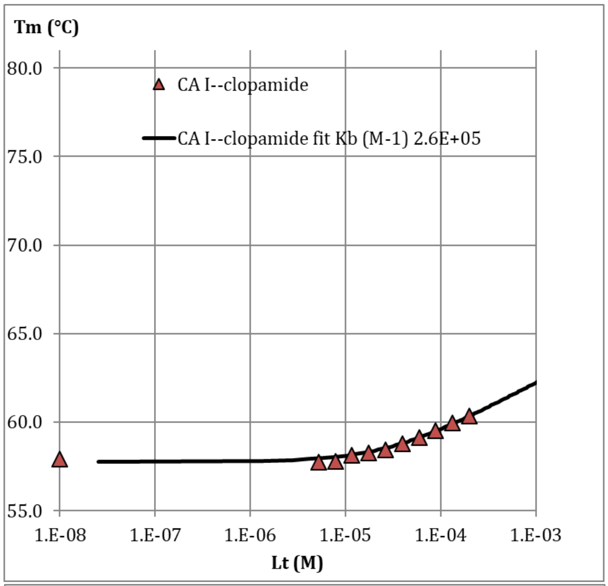

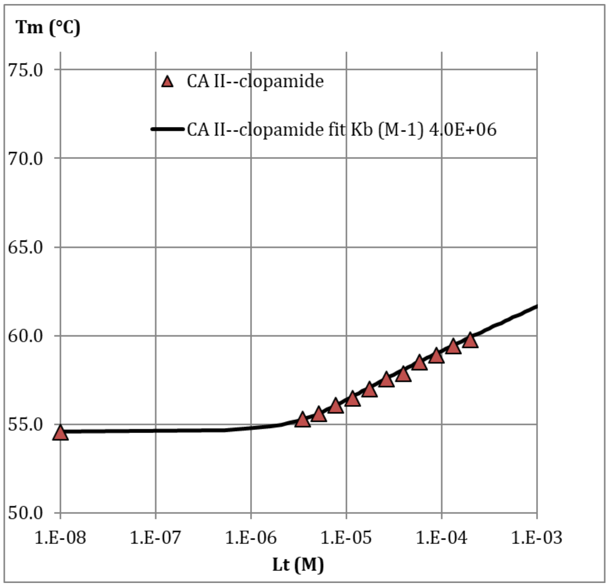

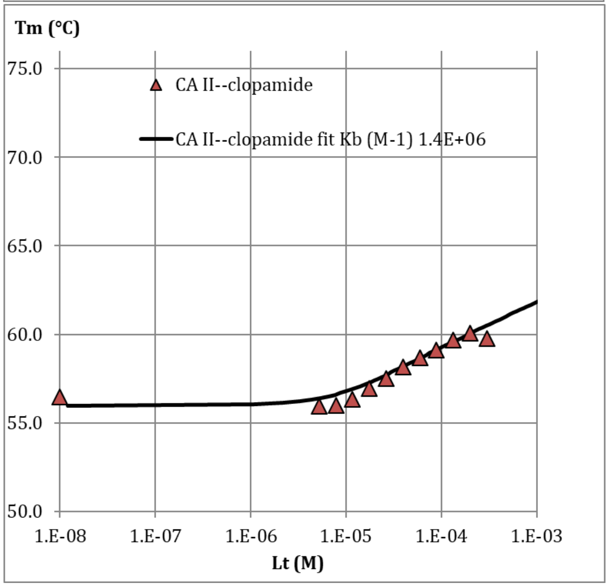

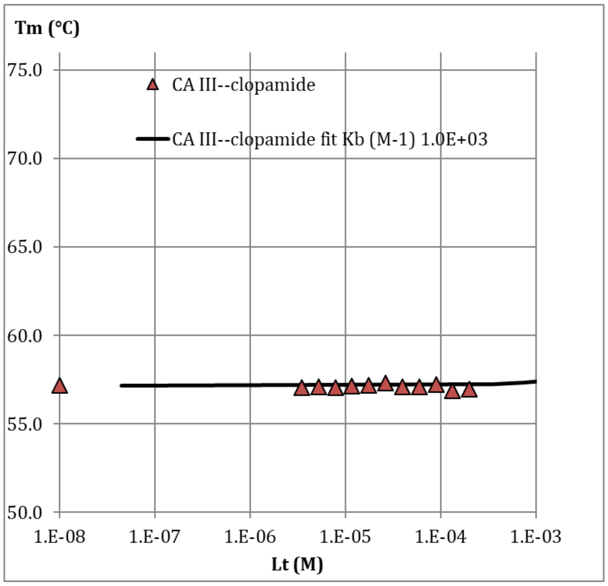

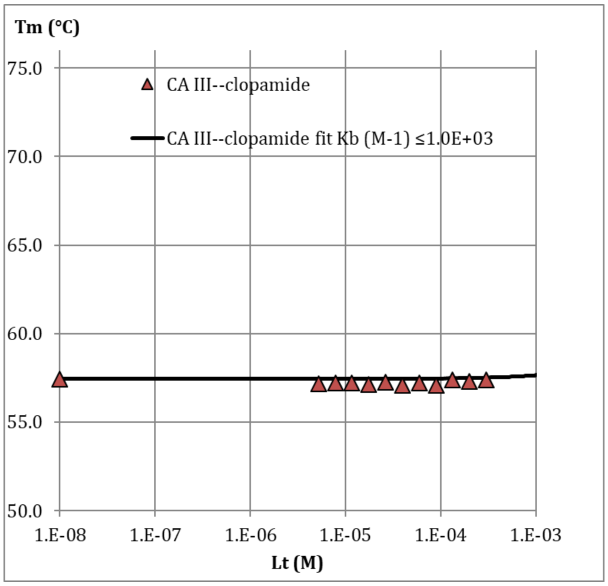

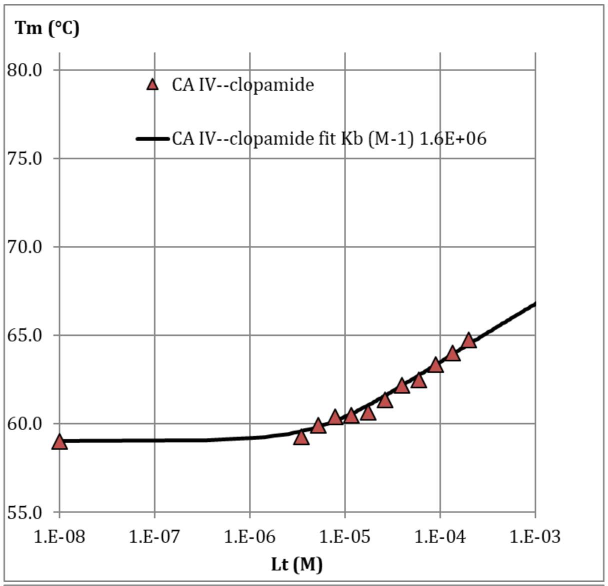
Tm (°C)
CA IV--clopamide
CA IV--clopamide fit Kb (M-1) 1.6E+06
Lt (M)

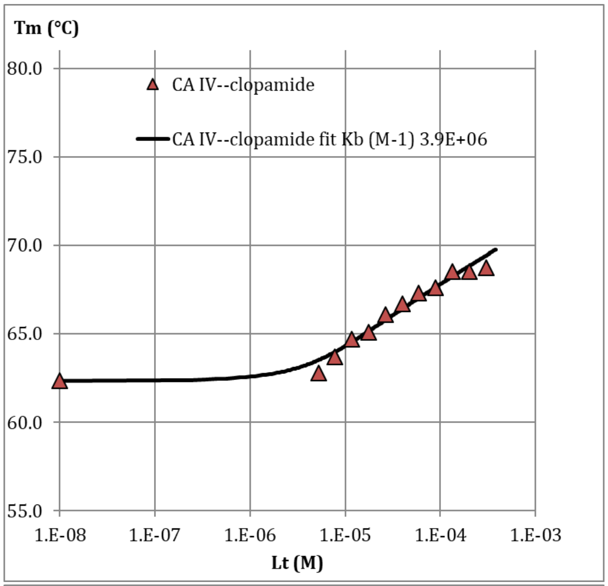
Tm (°C)
CA IV--clopamide
CA IV--clopamide fit Kb (M-1) 3.9E+06
Lt (M)

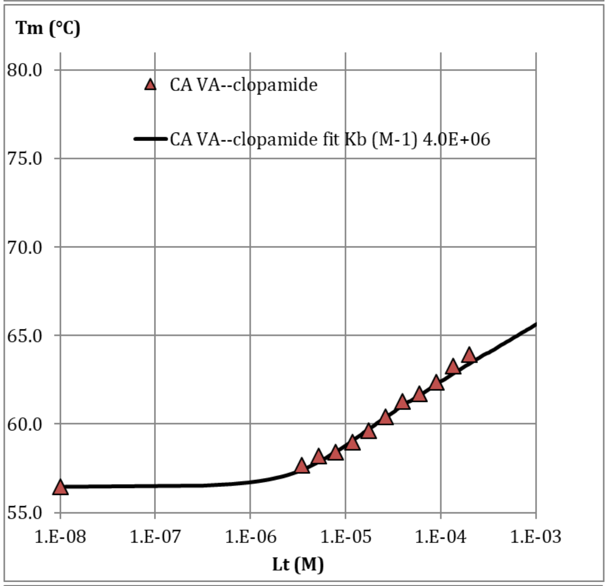
Tm (°C)
CA VA--clopamide
CA VA--clopamide fit Kb (M-1) 4.0E+06
Lt (M)

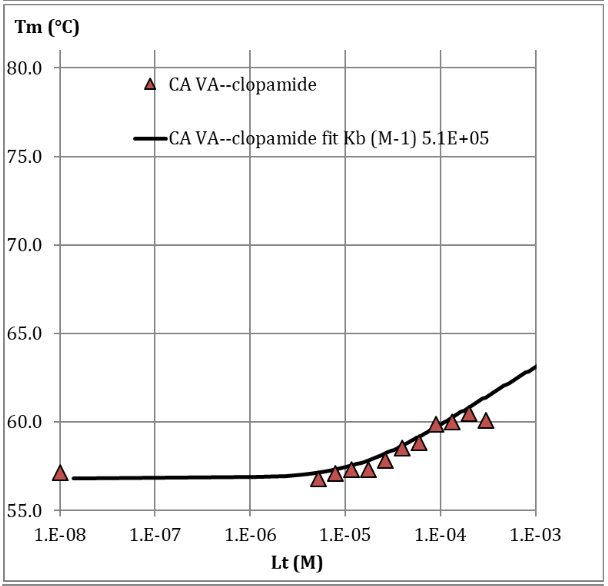
Tm (°C)
CA VA--clopamide
CA VA--clopamide fit Kb (M-1) 5.1E+05
Lt (M)

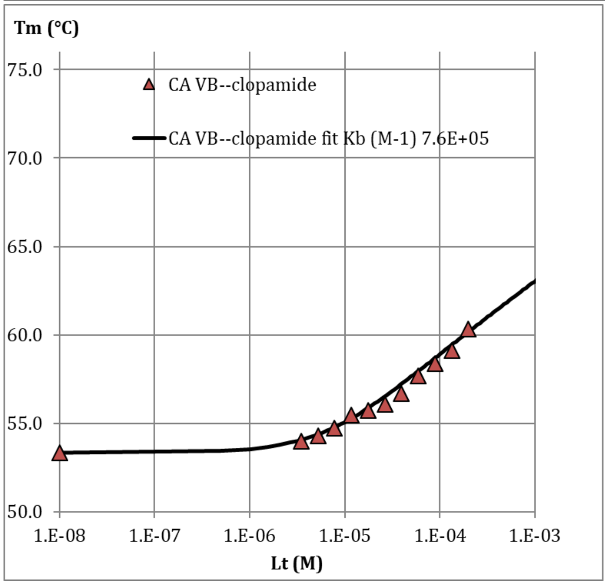
Tm (°C)
CA VB--clopamide
CA VB--clopamide fit Kb (M-1) 7.6E+05
Lt (M)

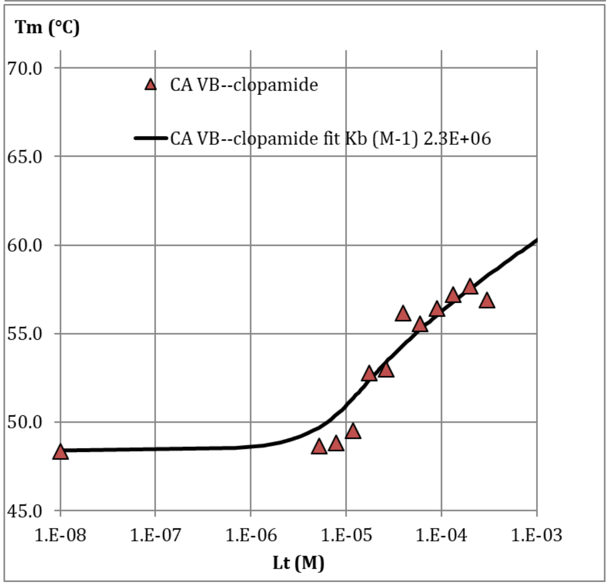
Tm (°C)
CA VB--clopamide
CA VB--clopamide fit Kb (M-1) 2.3E+06
Lt (M)

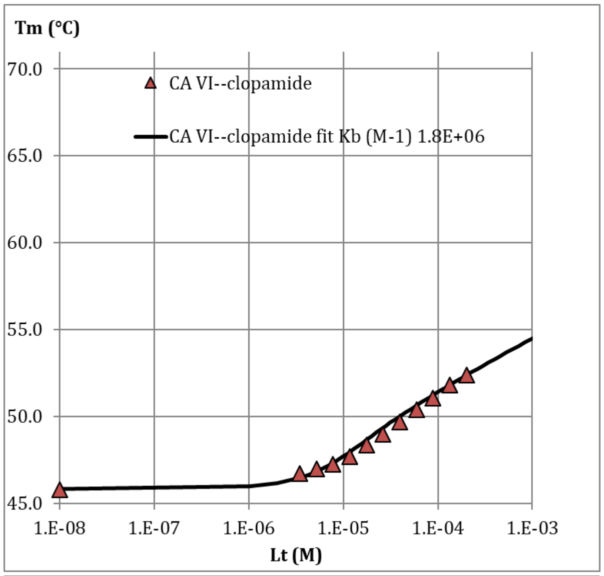
Tm (°C)
70.0
65.0
60.0
55.0
50.0
45.0
1.E-08
1.E-07
1.E-06
1.E-05
1.E-04
1.E-03
Lt (M)
CA VI--clopamide
CA VI--clopamide fit Kb (M-1) 1.8E+06

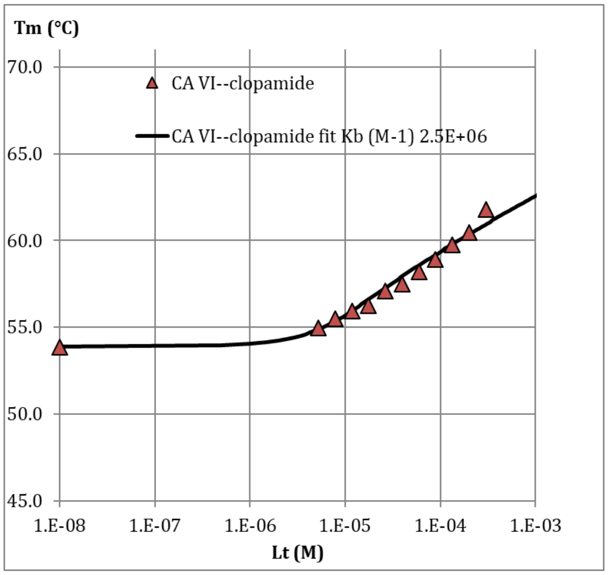
Tm (°C)
70.0
65.0
60.0
55.0
50.0
45.0
1.E-08
1.E-07
1.E-06
1.E-05
1.E-04
1.E-03
Lt (M)
CA VI--clopamide
CA VI--clopamide fit Kb (M-1) 2.5E+06

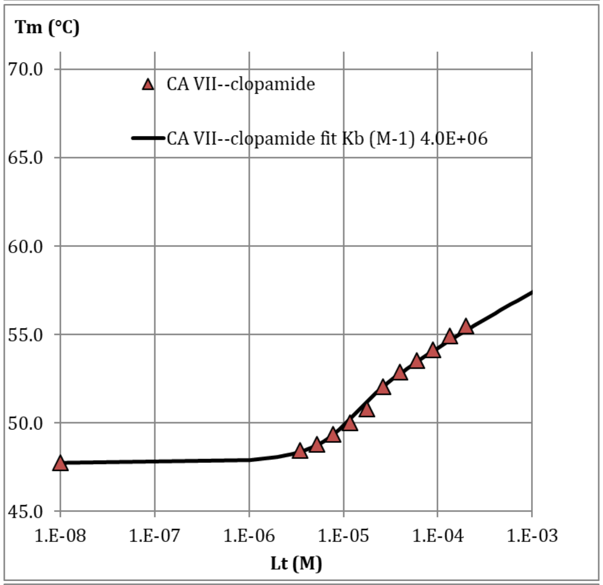
Tm (°C)
70.0
65.0
60.0
55.0
50.0
45.0
1.E-08
1.E-07
1.E-06
1.E-05
1.E-04
1.E-03
Lt (M)
CA VII--clopamide
CA VII--clopamide fit Kb (M-1) 4.0E+06

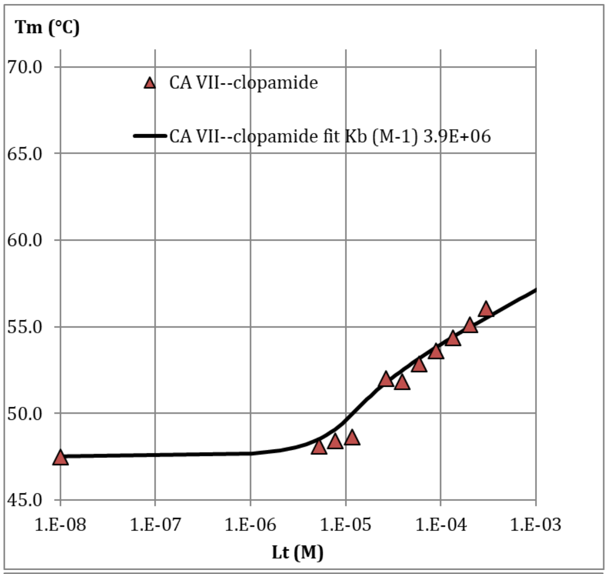
Tm (°C)
70.0
65.0
60.0
55.0
50.0
45.0
1.E-08
1.E-07
1.E-06
1.E-05
1.E-04
1.E-03
Lt (M)
CA VII--clopamide
CA VII--clopamide fit Kb (M-1) 3.9E+06

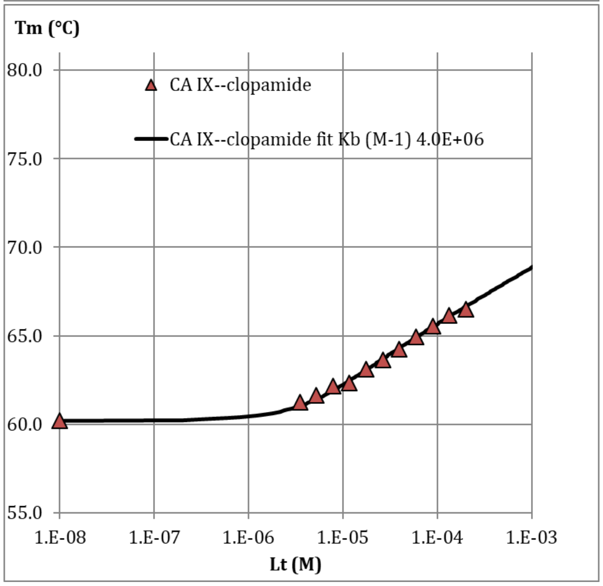
Tm (°C)
80.0
75.0
70.0
65.0
60.0
55.0
1.E-08
1.E-07
1.E-06
1.E-05
1.E-04
1.E-03
Lt (M)
CA IX--clopamide
CA IX--clopamide fit Kb (M-1) 4.0E+06

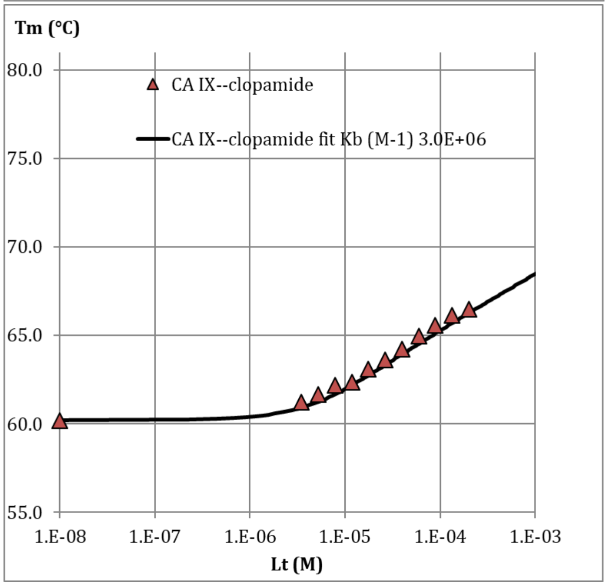
Tm (°C)
80.0
75.0
70.0
65.0
60.0
55.0
1.E-08
1.E-07
1.E-06
1.E-05
1.E-04
1.E-03
Lt (M)
CA IX--clopamide
CA IX--clopamide fit Kb (M-1) 3.0E+06

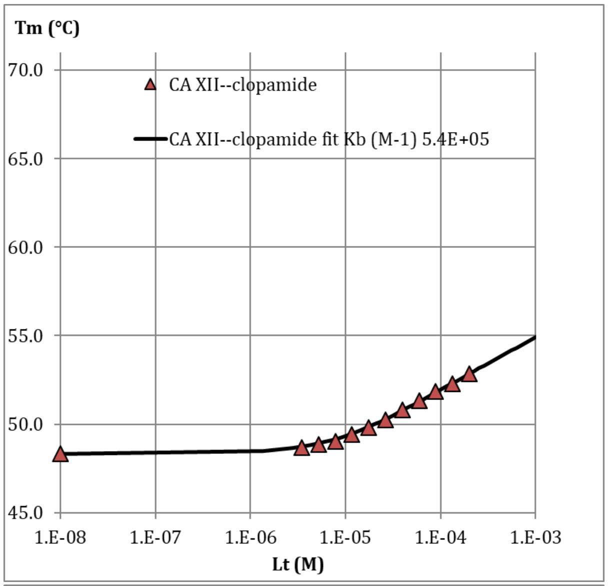
Tm (°C)
70.0
65.0
60.0
55.0
50.0
45.0
CA XII--clopamide
CA XII--clopamide fit Kb (M-1) 5.4E+05
1.E-08
1.E-07
1.E-06
1.E-05
1.E-04
1.E-03
Lt (M)

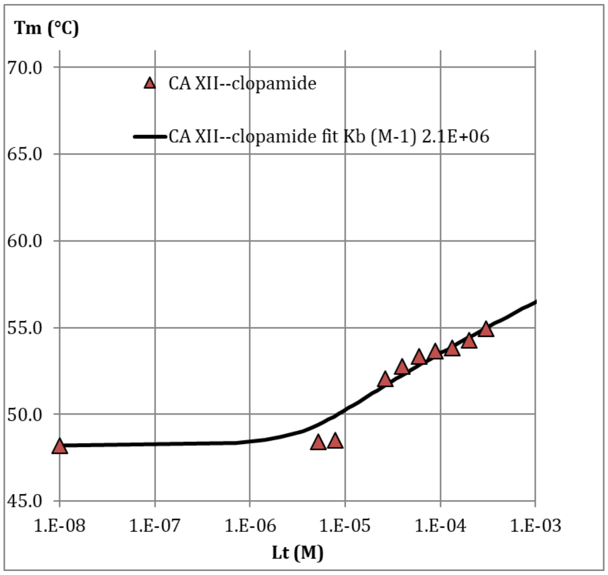
Tm (°C)
70.0
65.0
60.0
55.0
50.0
45.0
CA XII--clopamide
CA XII--clopamide fit Kb (M-1) 2.1E+06
1.E-08
1.E-07
1.E-06
1.E-05
1.E-04
1.E-03
Lt (M)

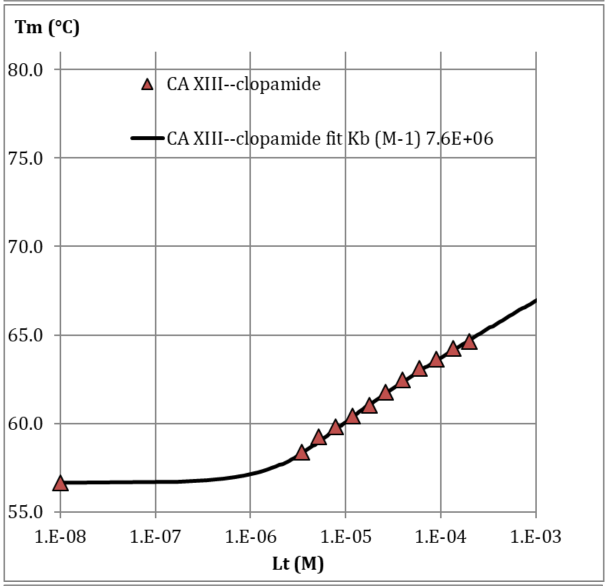
Tm (°C)
80.0
75.0
70.0
65.0
60.0
55.0
CA XIII--clopamide
CA XIII--clopamide fit Kb (M-1) 7.6E+06
1.E-08
1.E-07
1.E-06
1.E-05
1.E-04
1.E-03
Lt (M)

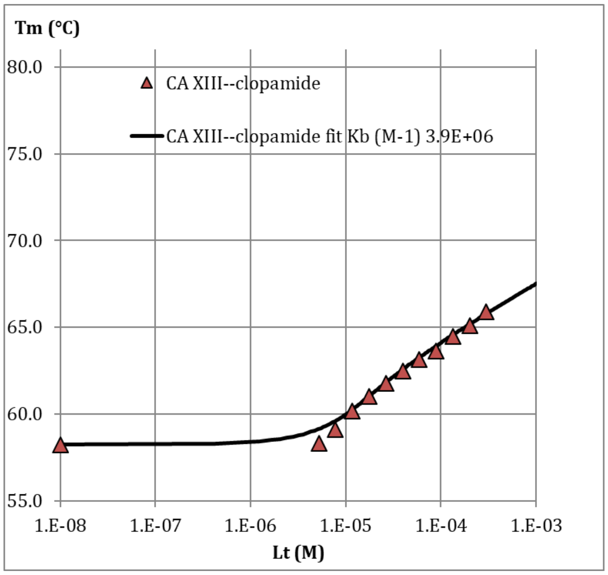
Tm (°C)
80.0
75.0
70.0
65.0
60.0
55.0
CA XIII--clopamide
CA XIII--clopamide fit Kb (M-1) 3.9E+06
1.E-08
1.E-07
1.E-06
1.E-05
1.E-04
1.E-03
Lt (M)

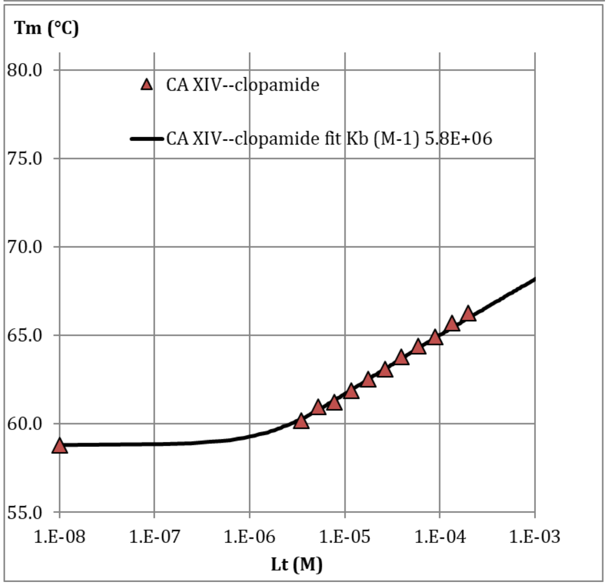
Tm (°C)
80.0
75.0
70.0
65.0
60.0
55.0
CA XIV--clopamide
CA XIV--clopamide fit Kb (M-1) 5.8E+06
1.E-08
1.E-07
1.E-06
1.E-05
1.E-04
1.E-03
Lt (M)

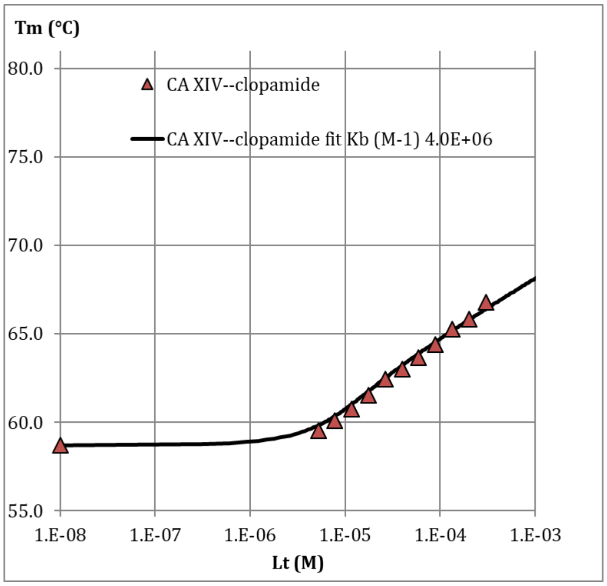
Tm (°C)
80.0
75.0
70.0
65.0
60.0
55.0
CA XIV--clopamide
CA XIV--clopamide fit Kb (M-1) 4.0E+06
1.E-08
1.E-07
1.E-06
1.E-05
1.E-04
1.E-03
Lt (M)

Isothermal titration calorimetry

CA II – clopamide

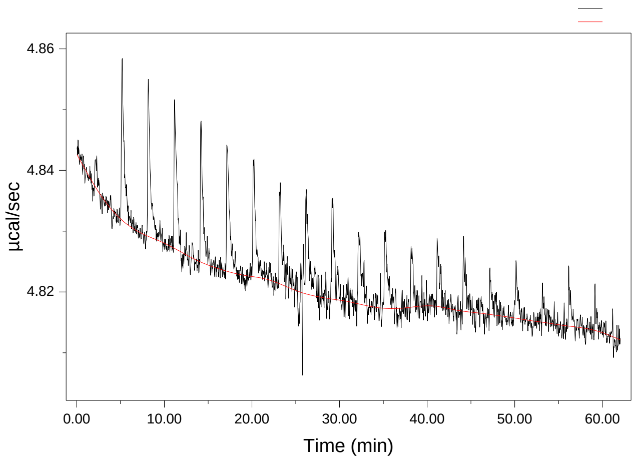

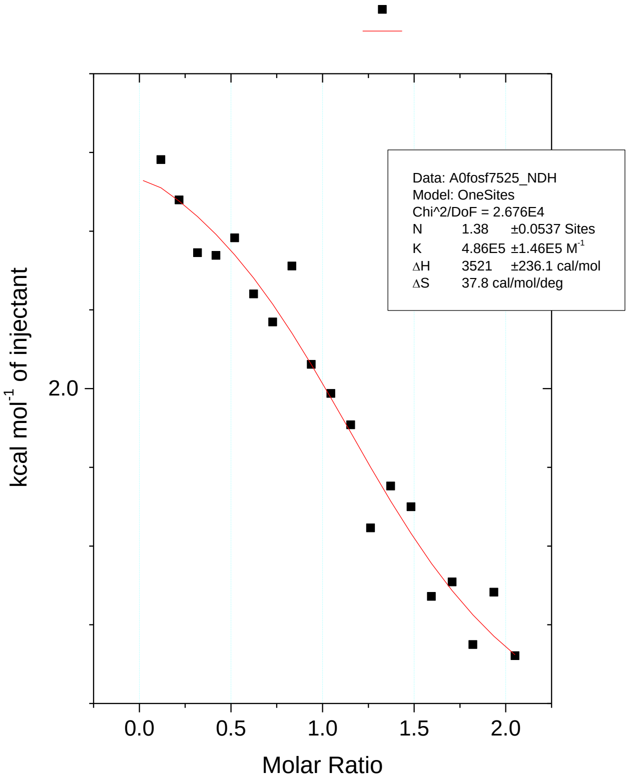

CA II – clopamide

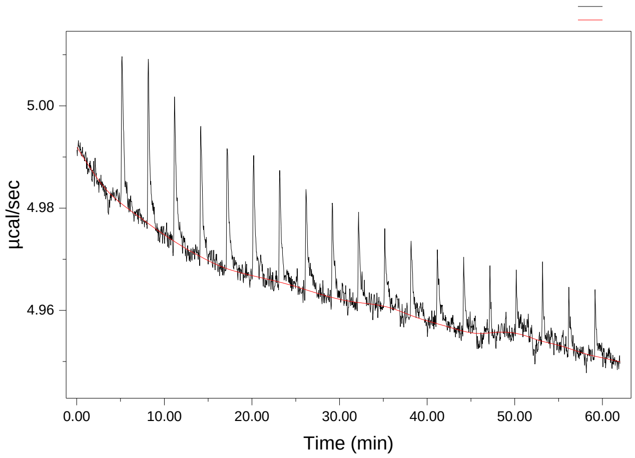

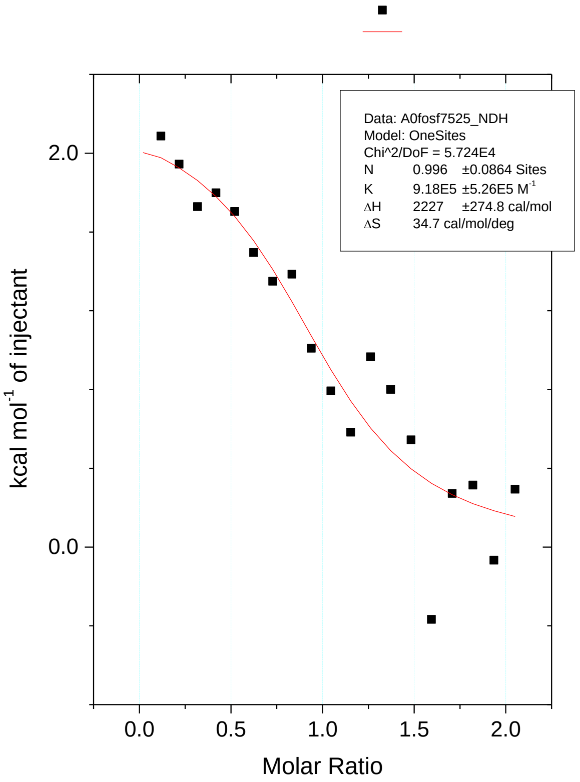

14. Xipamide

Fluorecence-based thermal shift assay

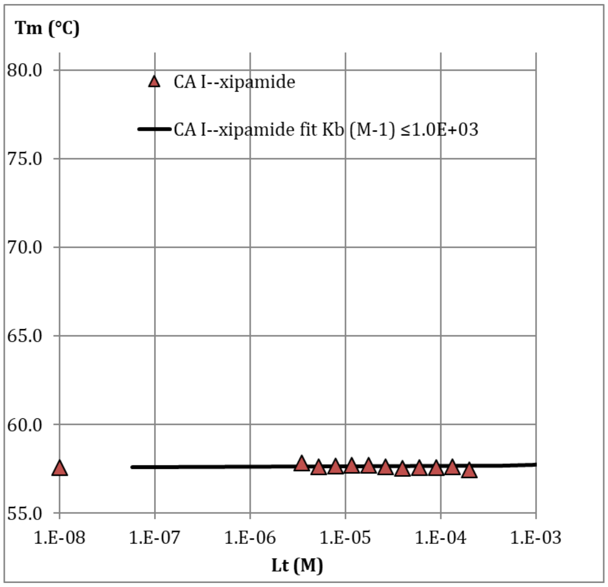

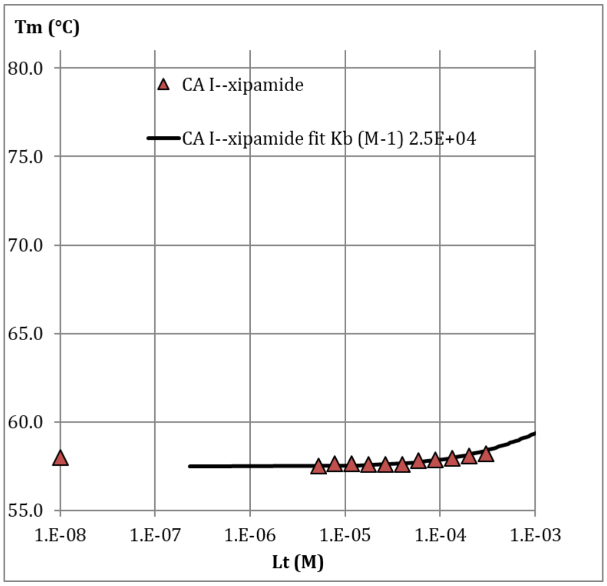

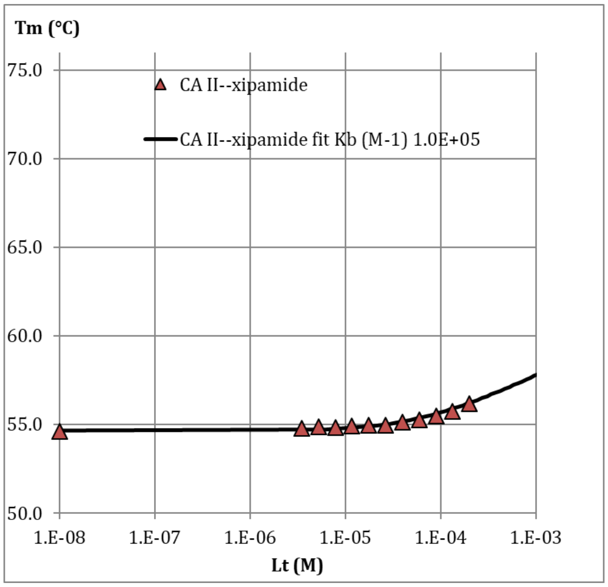

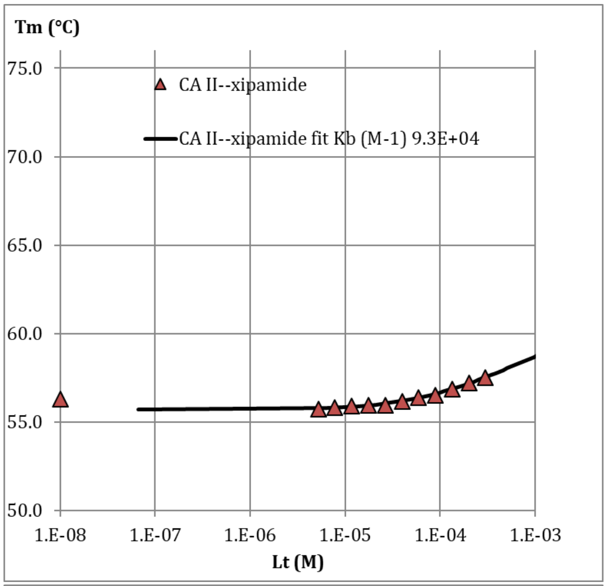

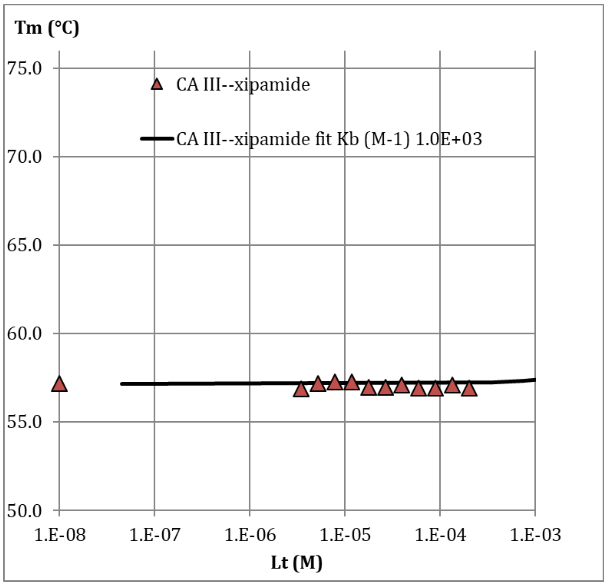

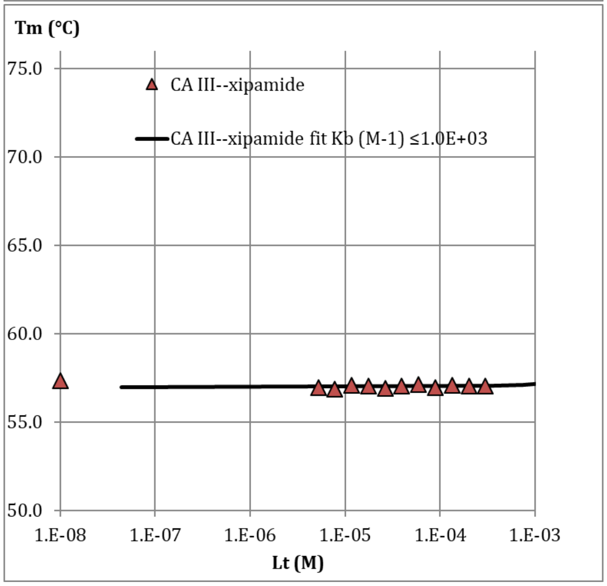

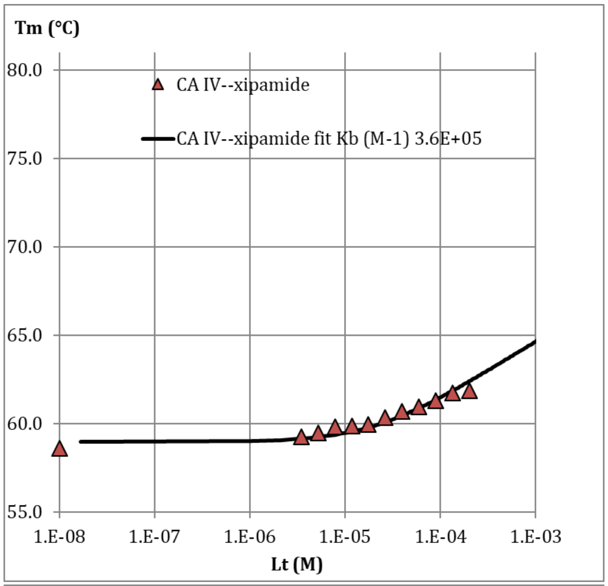
Tm (°C)
80.0
75.0
70.0
65.0
60.0
55.0
CA IV--xipamide
CA IV--xipamide fit Kb (M-1) 3.6E+05
1.E-08
1.E-07
1.E-06
1.E-05
1.E-04
1.E-03
Lt (M)

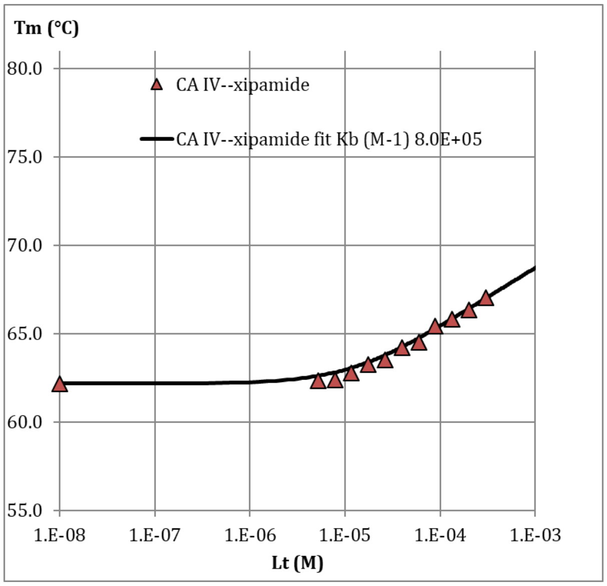
Tm (°C)
80.0
75.0
70.0
65.0
60.0
55.0
CA IV--xipamide
CA IV--xipamide fit Kb (M-1) 8.0E+05
1.E-08
1.E-07
1.E-06
1.E-05
1.E-04
1.E-03
Lt (M)

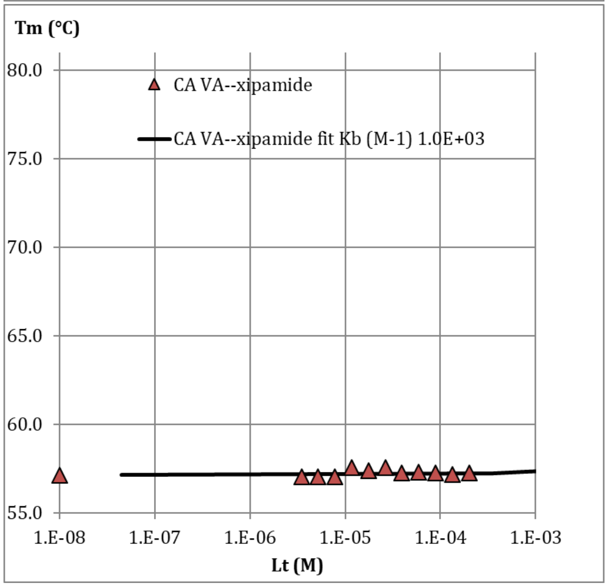
Tm (°C)
80.0
75.0
70.0
65.0
60.0
55.0
CA VA--xipamide
CA VA--xipamide fit Kb (M-1) 1.0E+03
1.E-08
1.E-07
1.E-06
1.E-05
1.E-04
1.E-03
Lt (M)

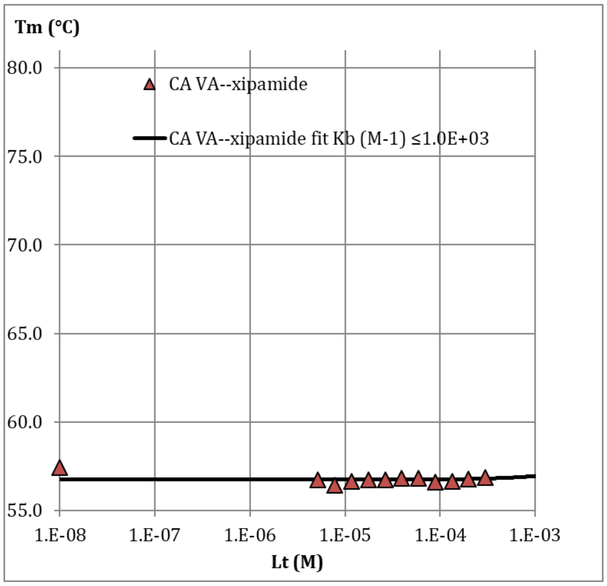
Tm (°C)
80.0
75.0
70.0
65.0
60.0
55.0
CA VA--xipamide
CA VA--xipamide fit Kb (M-1) ≤1.0E+03
1.E-08
1.E-07
1.E-06
1.E-05
1.E-04
1.E-03
Lt (M)

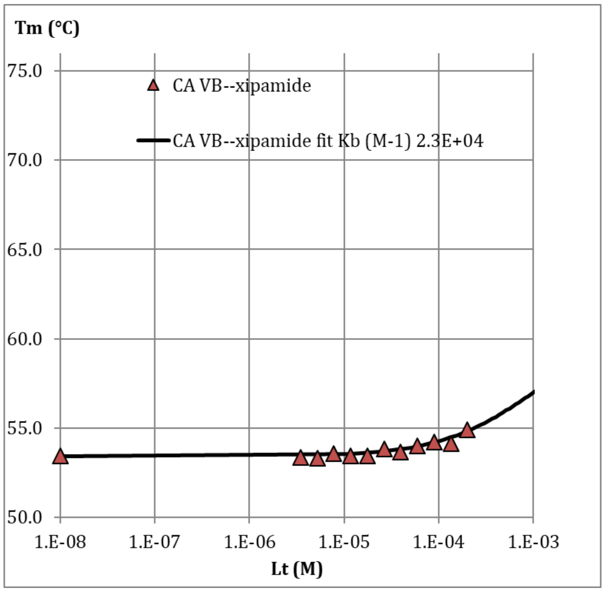
Tm (°C)
75.0
70.0
65.0
60.0
55.0
50.0
CA VB--xipamide
CA VB--xipamide fit Kb (M-1) 2.3E+04
1.E-08
1.E-07
1.E-06
1.E-05
1.E-04
1.E-03
Lt (M)

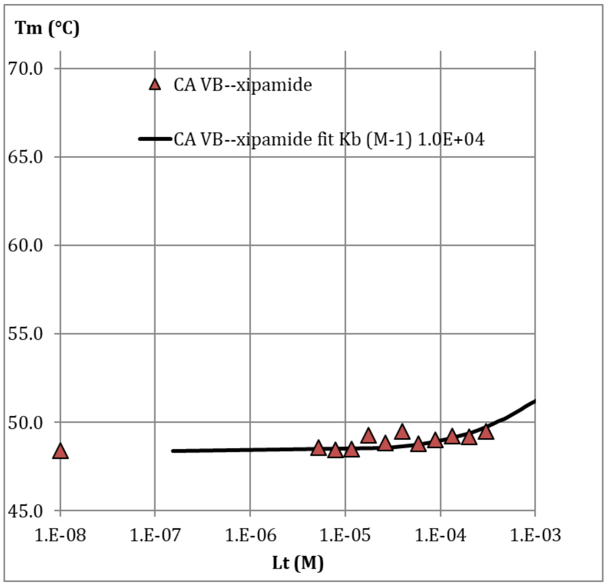
Tm (°C)
70.0
65.0
60.0
55.0
50.0
45.0
CA VB--xipamide
CA VB--xipamide fit Kb (M-1) 1.0E+04
1.E-08
1.E-07
1.E-06
1.E-05
1.E-04
1.E-03
Lt (M)

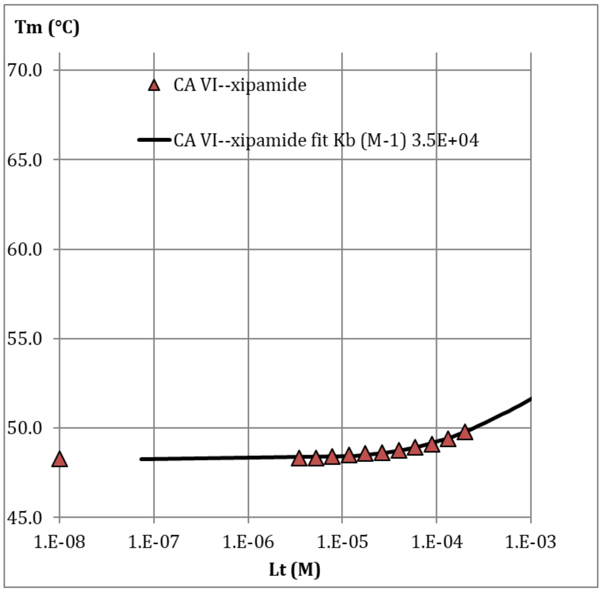
Tm (°C)
70.0
65.0
60.0
55.0
50.0
45.0
CA VI--xipamide
CA VI--xipamide fit Kb (M-1) 3.5E+04
1.E-08
1.E-07
1.E-06
1.E-05
1.E-04
1.E-03
Lt (M)

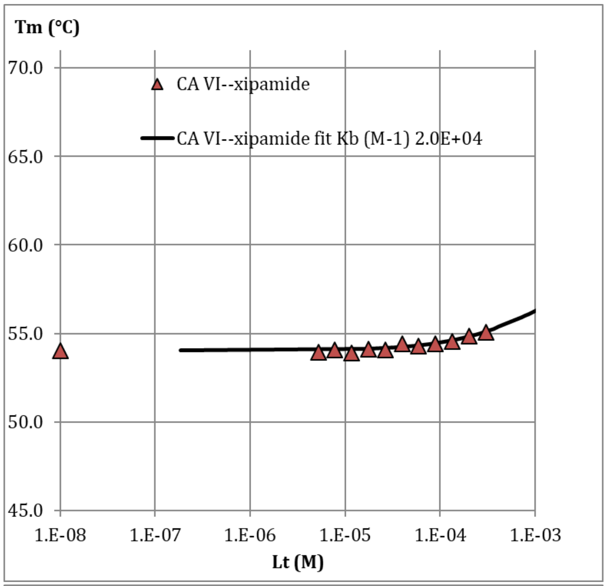
Tm (°C)
70.0
65.0
60.0
55.0
50.0
45.0
CA VI--xipamide
CA VI--xipamide fit Kb (M-1) 2.0E+04
1.E-08
1.E-07
1.E-06
1.E-05
1.E-04
1.E-03
Lt (M)

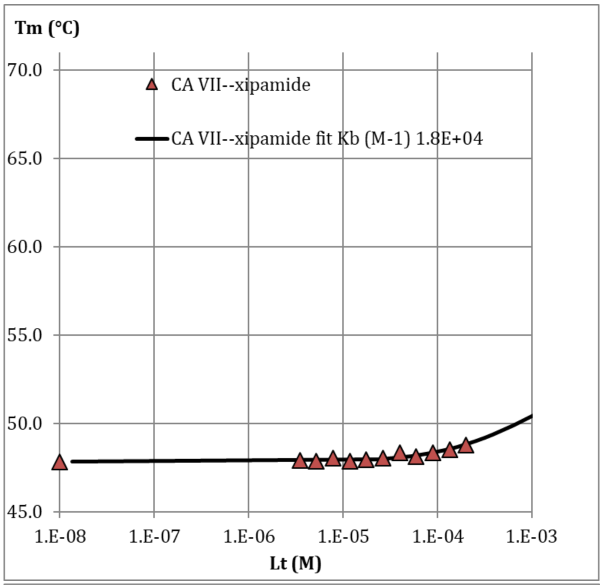
Tm (°C)
70.0
65.0
60.0
55.0
50.0
45.0
CA VII--xipamide
CA VII--xipamide fit Kb (M-1) 1.8E+04
1.E-08
1.E-07
1.E-06
1.E-05
1.E-04
1.E-03
Lt (M)

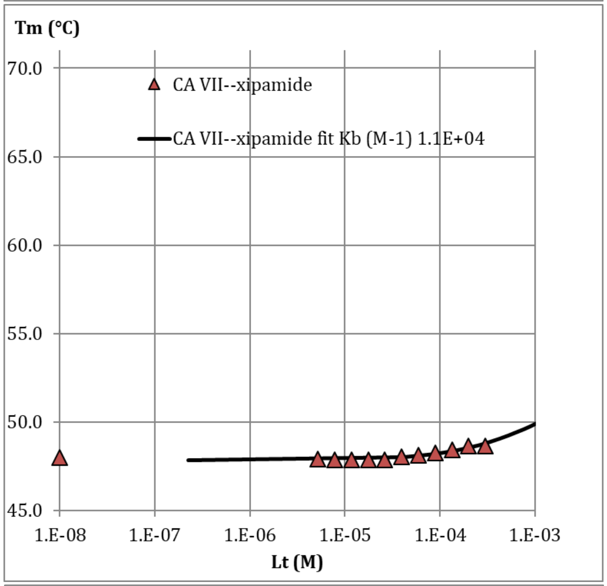
Tm (°C)
70.0
65.0
60.0
55.0
50.0
45.0
CA VII--xipamide
CA VII--xipamide fit Kb (M-1) 1.1E+04
1.E-08
1.E-07
1.E-06
1.E-05
1.E-04
1.E-03
Lt (M)

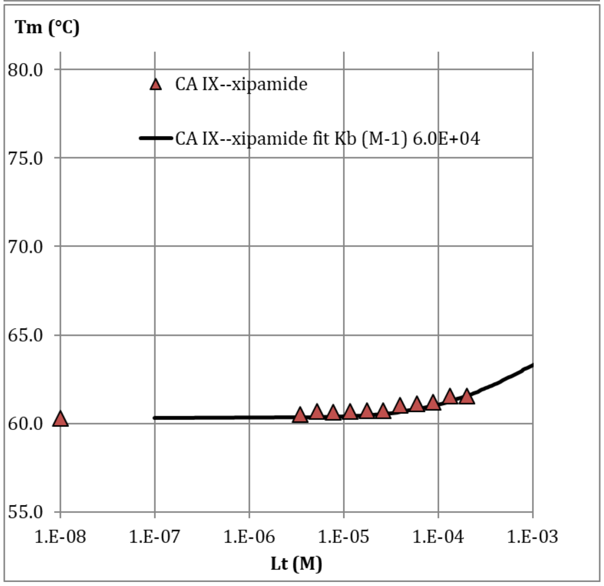
Tm (°C)
80.0
75.0
70.0
65.0
60.0
55.0
CA IX--xipamide
CA IX--xipamide fit Kb (M-1) 6.0E+04
1.E-08
1.E-07
1.E-06
1.E-05
1.E-04
1.E-03
Lt (M)

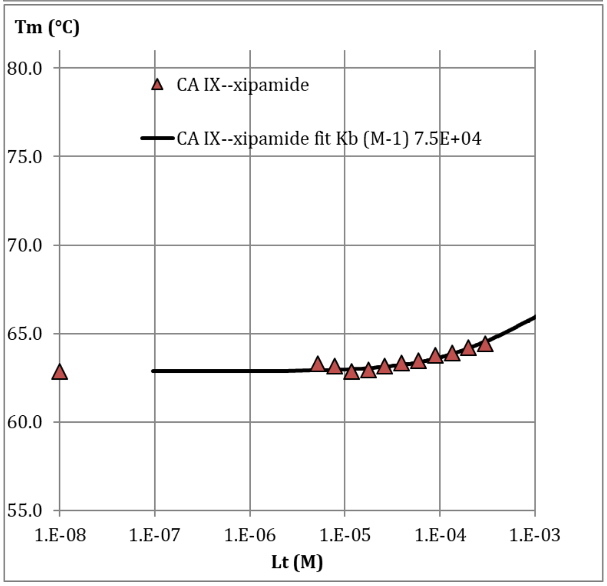
Tm (°C)
80.0
75.0
70.0
65.0
60.0
55.0
CA IX--xipamide
CA IX--xipamide fit Kb (M-1) 7.5E+04
1.E-08
1.E-07
1.E-06
1.E-05
1.E-04
1.E-03
Lt (M)

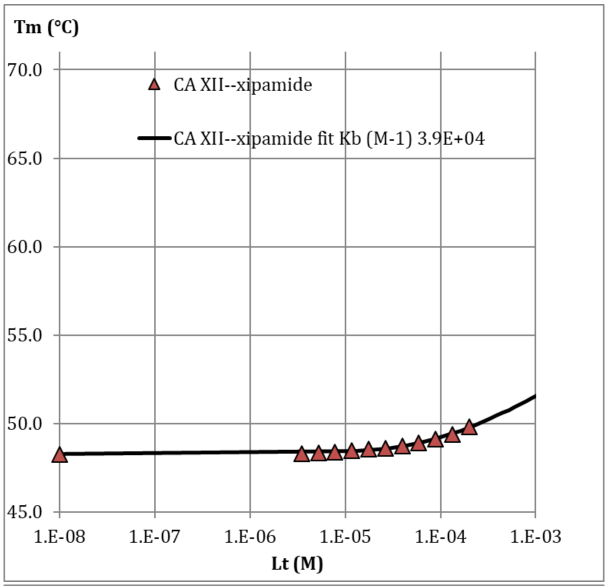
Tm (°C)
70.0
65.0
60.0
55.0
50.0
45.0
CA XII--xipamide
CA XII--xipamide fit Kb (M-1) 3.9E+04
1.E-08
1.E-07
1.E-06
1.E-05
1.E-04
1.E-03
Lt (M)

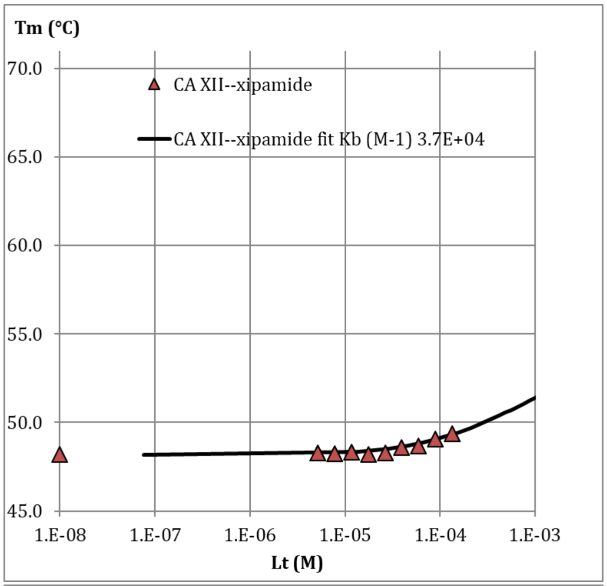
Tm (°C)
70.0
65.0
60.0
55.0
50.0
45.0
CA XII--xipamide
CA XII--xipamide fit Kb (M-1) 3.7E+04
1.E-08
1.E-07
1.E-06
1.E-05
1.E-04
1.E-03
Lt (M)

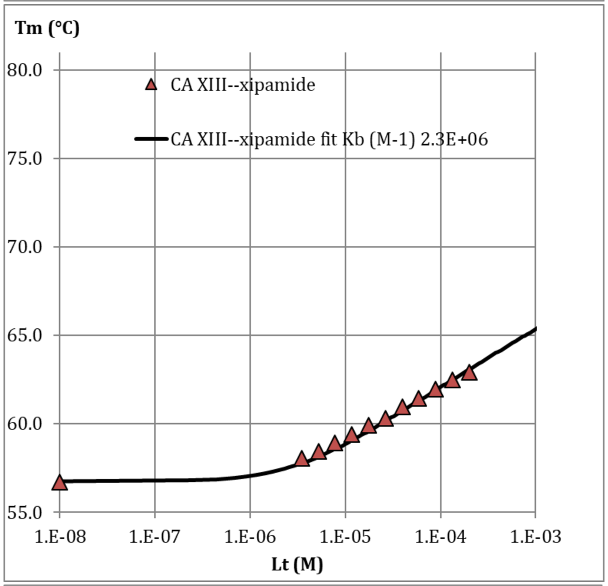
Tm (°C)
80.0
75.0
70.0
65.0
60.0
55.0
CA XIII--xipamide
CA XIII--xipamide fit Kb (M-1) 2.3E+06
1.E-08
1.E-07
1.E-06
1.E-05
1.E-04
1.E-03
Lt (M)

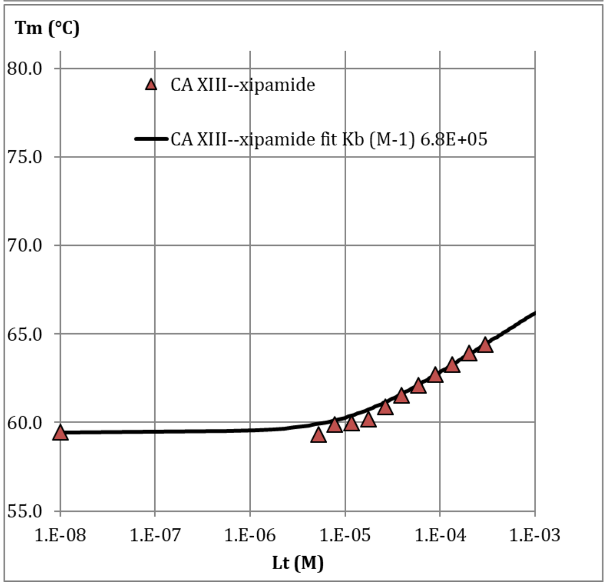
Tm (°C)
80.0
75.0
70.0
65.0
60.0
55.0
CA XIII--xipamide
CA XIII--xipamide fit Kb (M-1) 6.8E+05
1.E-08
1.E-07
1.E-06
1.E-05
1.E-04
1.E-03
Lt (M)

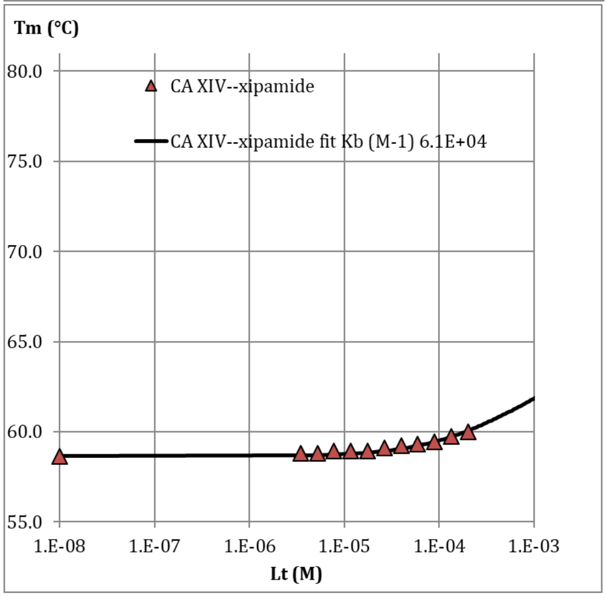
Tm (°C)
80.0
75.0
70.0
65.0
60.0
55.0
CA XIV--xipamide
CA XIV--xipamide fit Kb (M-1) 6.1E+04
1.E-08
1.E-07
1.E-06
1.E-05
1.E-04
1.E-03
Lt (M)

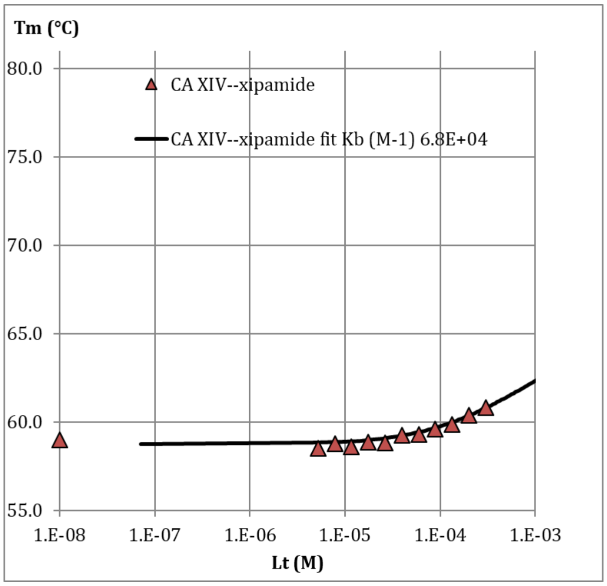
Tm (°C)
80.0
75.0
70.0
65.0
60.0
55.0
CA XIV--xipamide
CA XIV--xipamide fit Kb (M-1) 6.8E+04
1.E-08
1.E-07
1.E-06
1.E-05
1.E-04
1.E-03
Lt (M)

15. Furosemide

Fluorecence-based thermal shift assay

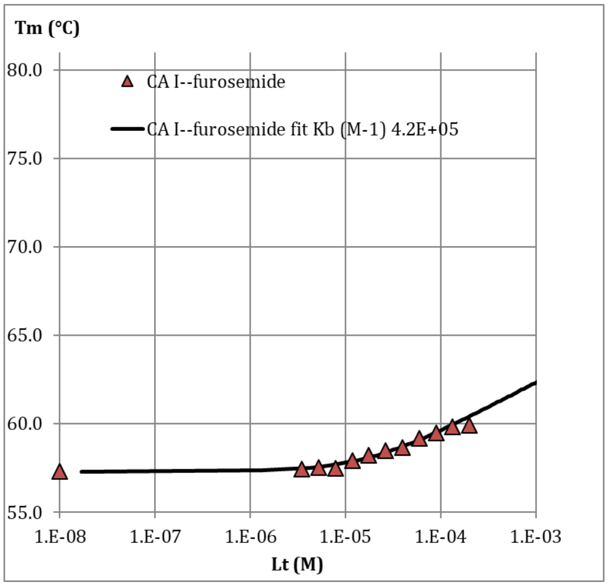

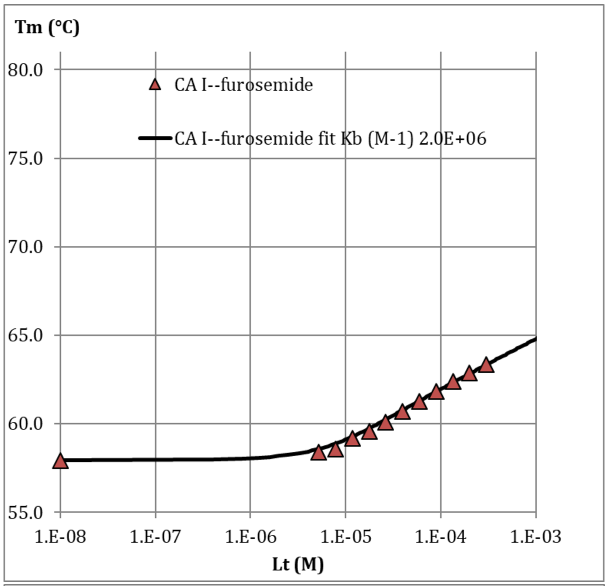

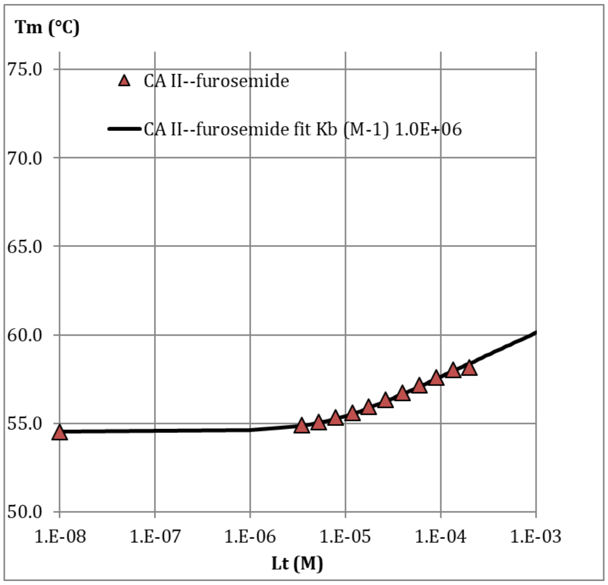

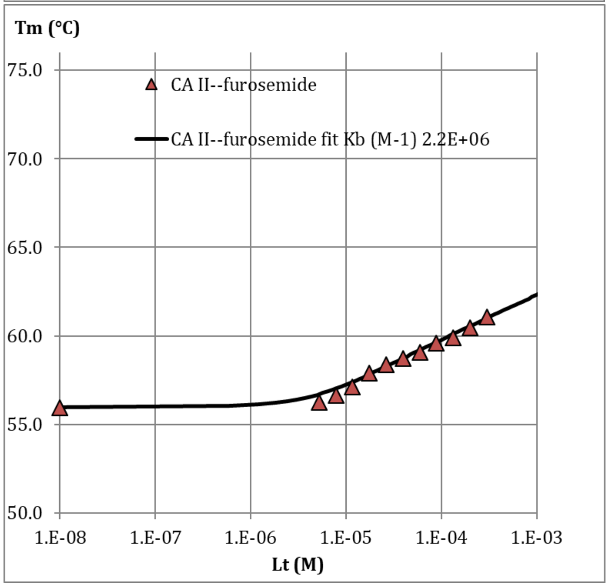

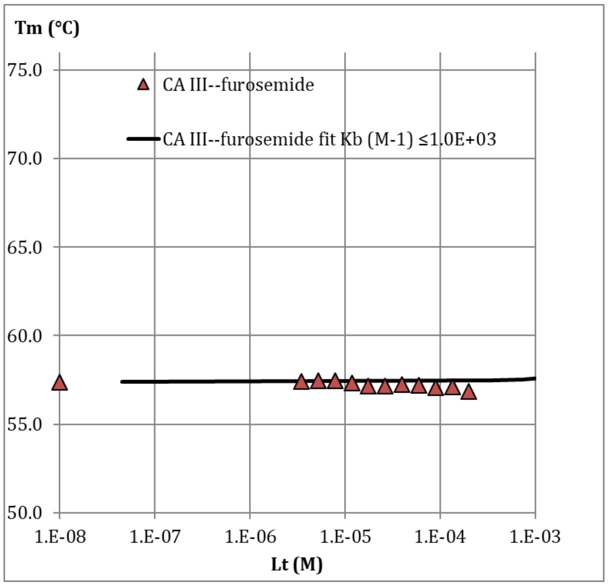

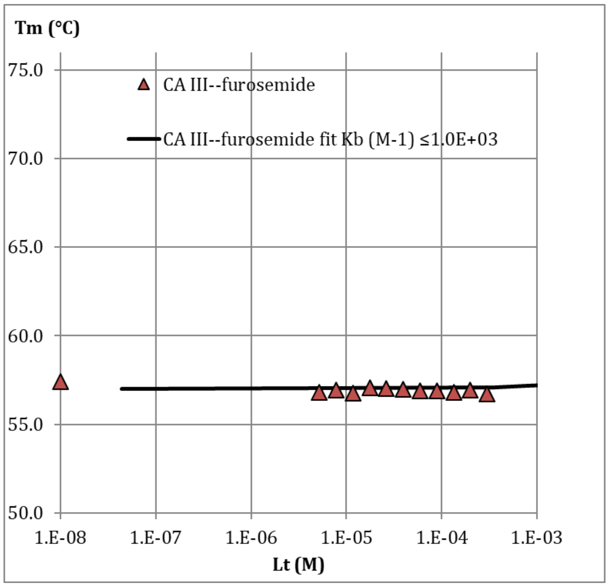

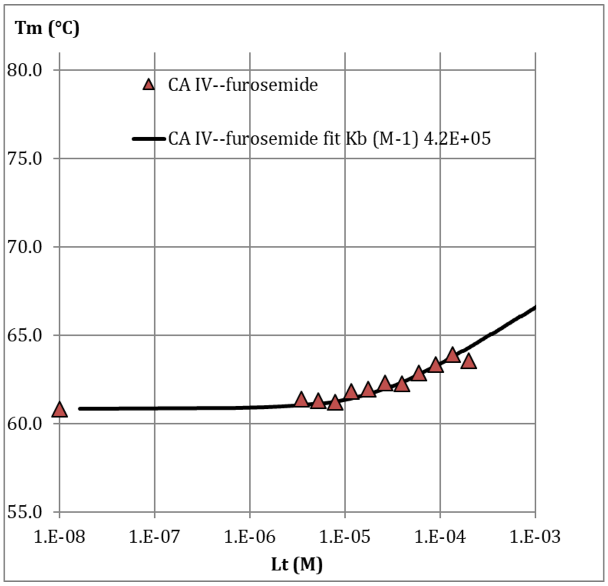
Tm (°C)
CA IV--furosemide
CA IV--furosemide fit Kb (M-1) 4.2E+05
80.0
75.0
70.0
65.0
60.0
55.0
1.E-08
1.E-07
1.E-06
1.E-05
1.E-04
1.E-03
Lt (M)

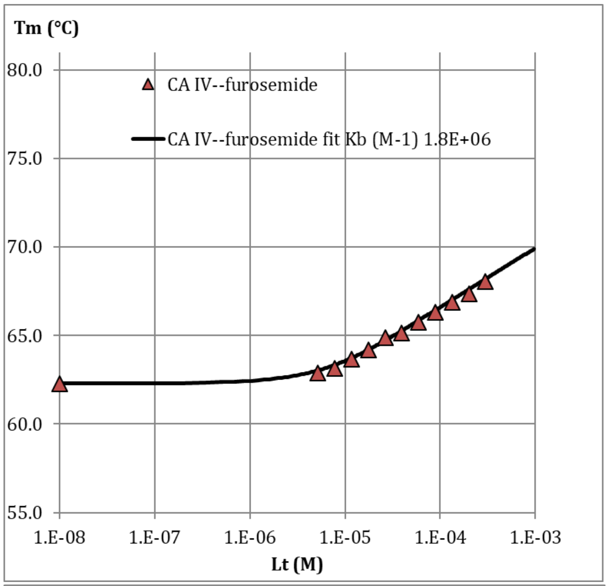
Tm (°C)
CA IV--furosemide
CA IV--furosemide fit Kb (M-1) 1.8E+06
80.0
75.0
70.0
65.0
60.0
55.0
1.E-08
1.E-07
1.E-06
1.E-05
1.E-04
1.E-03
Lt (M)

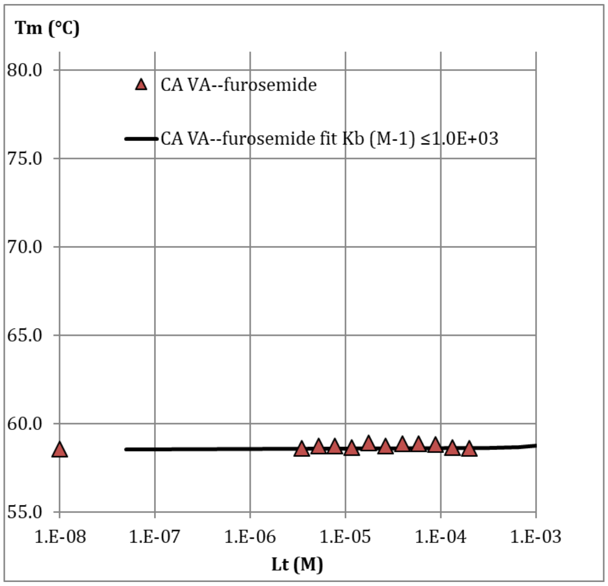
Tm (°C)
CA VA--furosemide
CA VA--furosemide fit Kb (M-1) ≤1.0E+03
80.0
75.0
70.0
65.0
60.0
55.0
1.E-08
1.E-07
1.E-06
1.E-05
1.E-04
1.E-03
Lt (M)

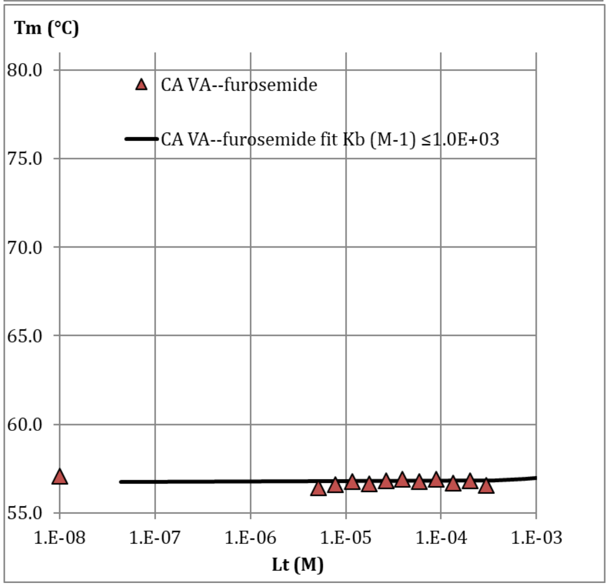
Tm (°C)
CA VA--furosemide
CA VA--furosemide fit Kb (M-1) ≤1.0E+03
80.0
75.0
70.0
65.0
60.0
55.0
1.E-08
1.E-07
1.E-06
1.E-05
1.E-04
1.E-03
Lt (M)

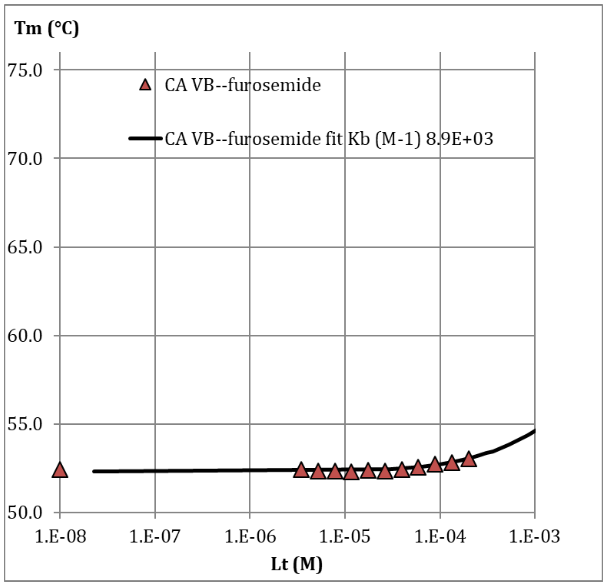
Tm (°C)
CA VB--furosemide
CA VB--furosemide fit Kb (M-1) 8.9E+03
75.0
70.0
65.0
60.0
55.0
50.0
1.E-08
1.E-07
1.E-06
1.E-05
1.E-04
1.E-03
Lt (M)

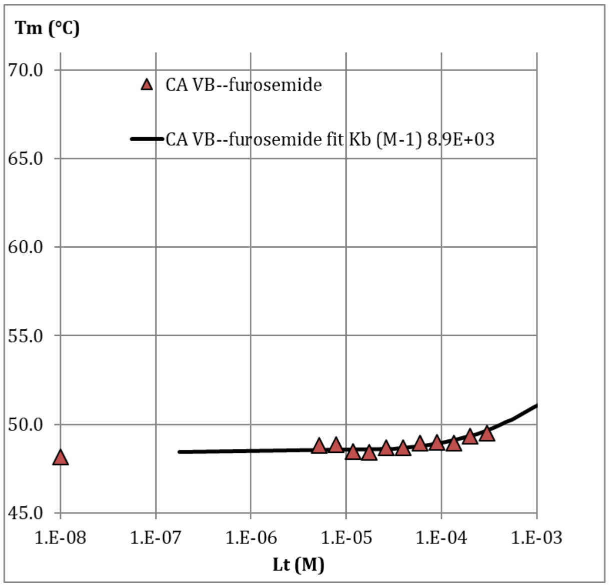
Tm (°C)
CA VB--furosemide
CA VB--furosemide fit Kb (M-1) 8.9E+03
70.0
65.0
60.0
55.0
50.0
45.0
1.E-08
1.E-07
1.E-06
1.E-05
1.E-04
1.E-03
Lt (M)

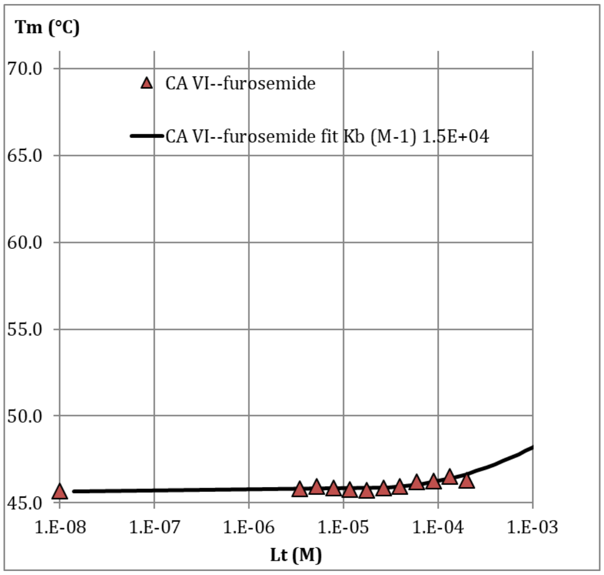

Tm (°C)
70.0
65.0
60.0
55.0
50.0
45.0
CA VI--furosemide
CA VI--furosemide fit Kb (M-1) 1.5E+04
1.E-08
1.E-07
1.E-06
1.E-05
1.E-04
1.E-03
Lt (M)

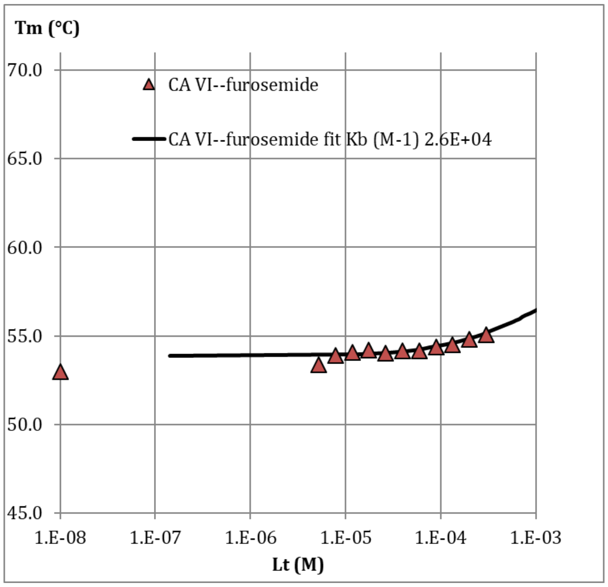

Tm (°C)
70.0
65.0
60.0
55.0
50.0
45.0
CA VI--furosemide
CA VI--furosemide fit Kb (M-1) 2.6E+04
1.E-08
1.E-07
1.E-06
1.E-05
1.E-04
1.E-03
Lt (M)

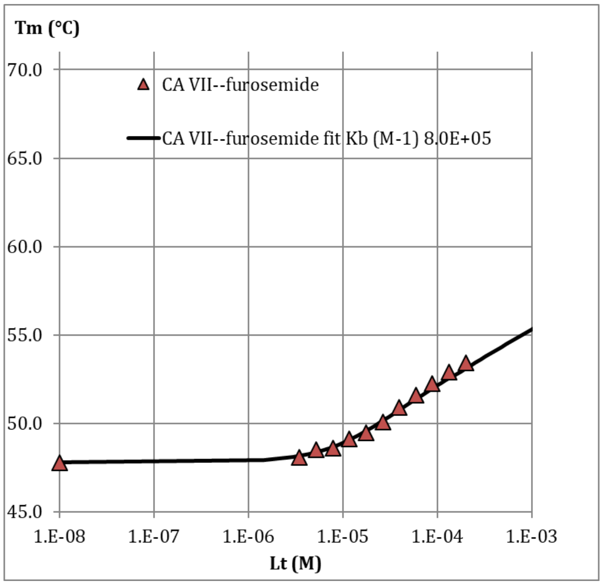

Tm (°C)
70.0
65.0
60.0
55.0
50.0
45.0
CA VII--furosemide
CA VII--furosemide fit Kb (M-1) 8.0E+05
1.E-08
1.E-07
1.E-06
1.E-05
1.E-04
1.E-03
Lt (M)

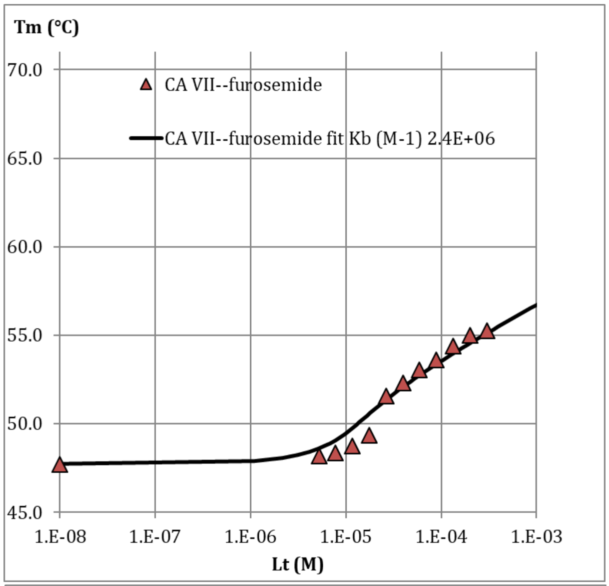

Tm (°C)
70.0
65.0
60.0
55.0
50.0
45.0
CA VII--furosemide
CA VII--furosemide fit Kb (M-1) 2.4E+06
1.E-08
1.E-07
1.E-06
1.E-05
1.E-04
1.E-03
Lt (M)

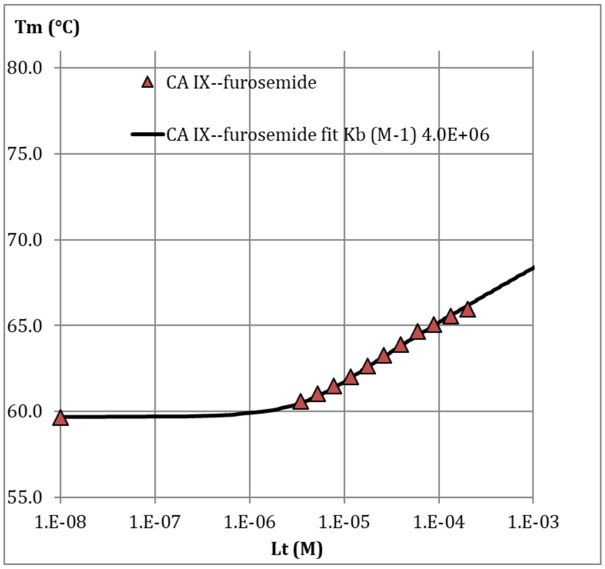

Tm (°C)
80.0
75.0
70.0
65.0
60.0
55.0
CA IX--furosemide
CA IX--furosemide fit Kb (M-1) 4.0E+06
1.E-08
1.E-07
1.E-06
1.E-05
1.E-04
1.E-03
Lt (M)

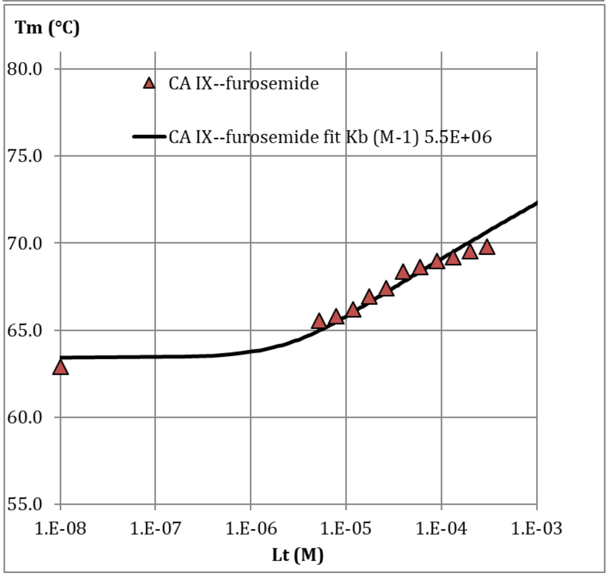

Tm (°C)
80.0
75.0
70.0
65.0
60.0
55.0
CA IX--furosemide
CA IX--furosemide fit Kb (M-1) 5.5E+06
1.E-08
1.E-07
1.E-06
1.E-05
1.E-04
1.E-03
Lt (M)

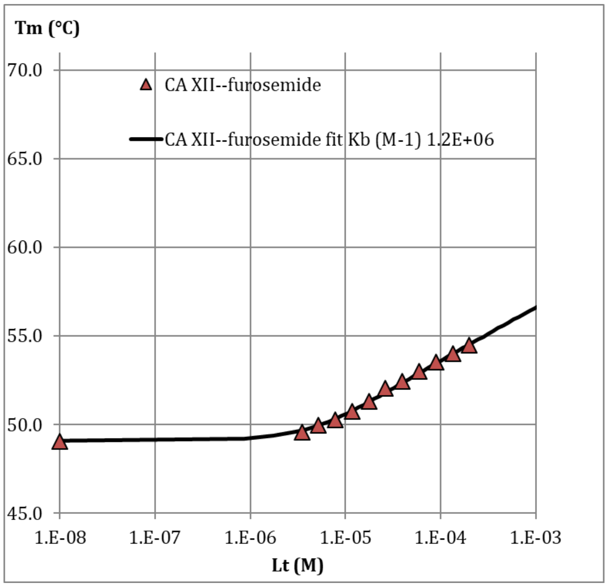
Tm (°C)
70.0
65.0
60.0
55.0
50.0
45.0
CA XII--furosemide
CA XII--furosemide fit Kb (M-1) 1.2E+06
1.E-08
1.E-07
1.E-06
1.E-05
1.E-04
1.E-03
Lt (M)

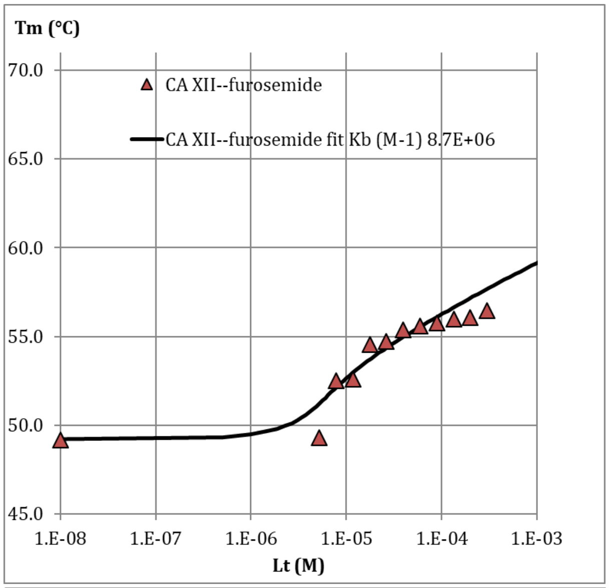
Tm (°C)
70.0
65.0
60.0
55.0
50.0
45.0
CA XII--furosemide
CA XII--furosemide fit Kb (M-1) 8.7E+06
1.E-08
1.E-07
1.E-06
1.E-05
1.E-04
1.E-03
Lt (M)

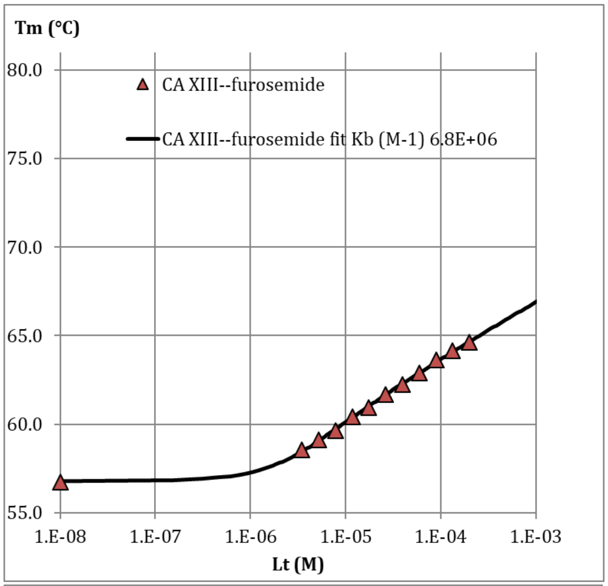
Tm (°C)
80.0
75.0
70.0
65.0
60.0
55.0
CA XIII--furosemide
CA XIII--furosemide fit Kb (M-1) 6.8E+06
1.E-08
1.E-07
1.E-06
1.E-05
1.E-04
1.E-03
Lt (M)

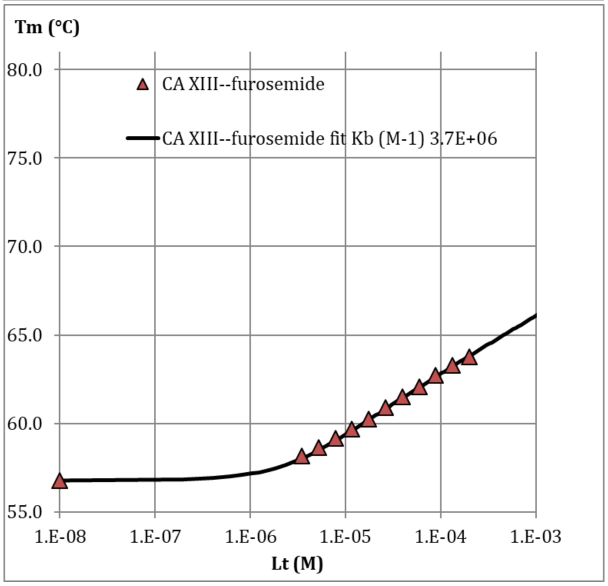
Tm (°C)
80.0
75.0
70.0
65.0
60.0
55.0
CA XIII--furosemide
CA XIII--furosemide fit Kb (M-1) 3.7E+06
1.E-08
1.E-07
1.E-06
1.E-05
1.E-04
1.E-03
Lt (M)

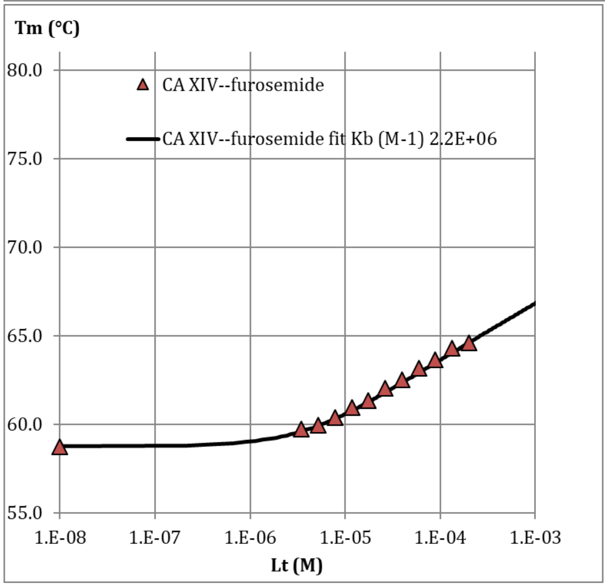
Tm (°C)
80.0
75.0
70.0
65.0
60.0
55.0
CA XIV--furosemide
CA XIV--furosemide fit Kb (M-1) 2.2E+06
1.E-08
1.E-07
1.E-06
1.E-05
1.E-04
1.E-03
Lt (M)

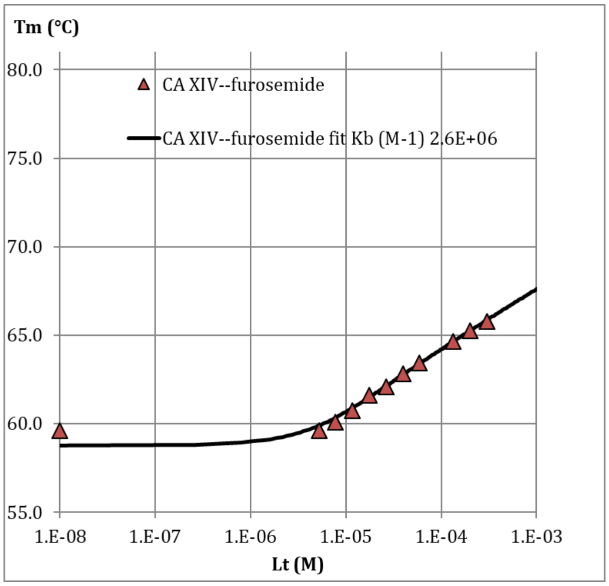
Tm (°C)
80.0
75.0
70.0
65.0
60.0
55.0
CA XIV--furosemide
CA XIV--furosemide fit Kb (M-1) 2.6E+06
1.E-08
1.E-07
1.E-06
1.E-05
1.E-04
1.E-03
Lt (M)

16. Chlorthalidone

Fluorecence-based thermal shift assay

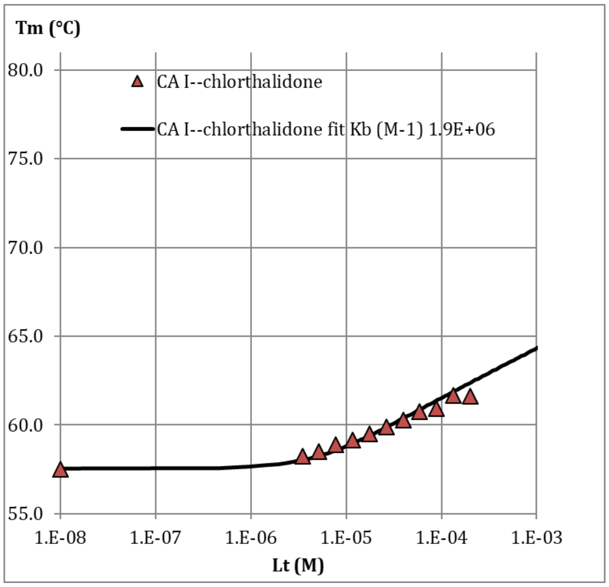

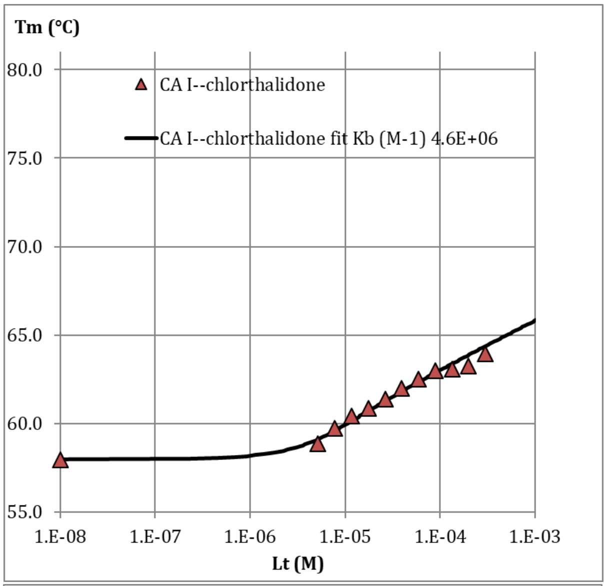

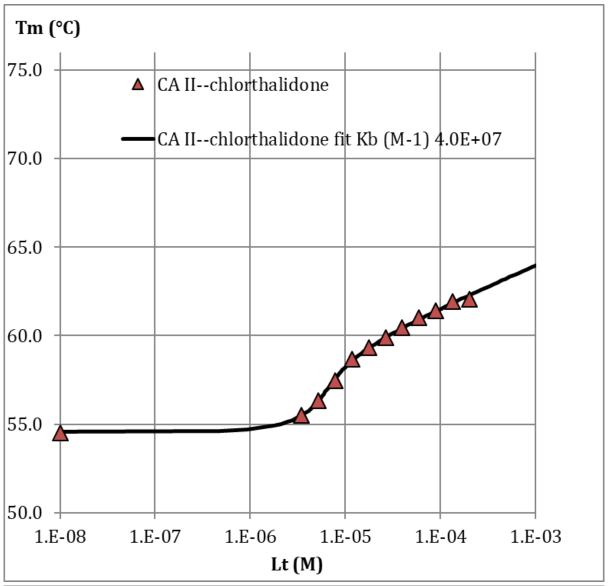

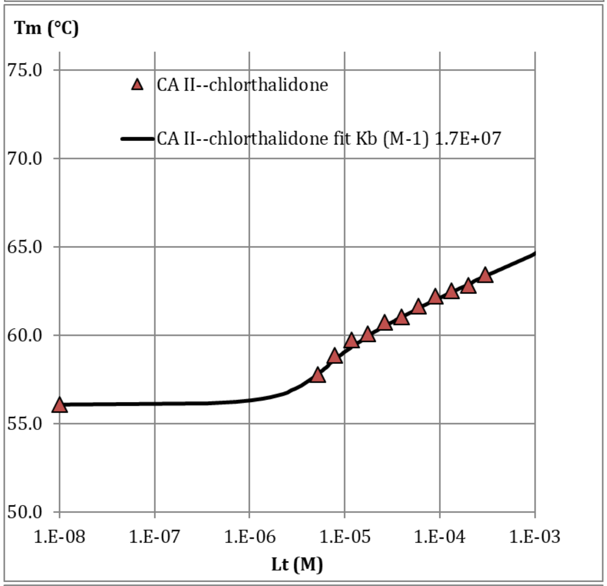

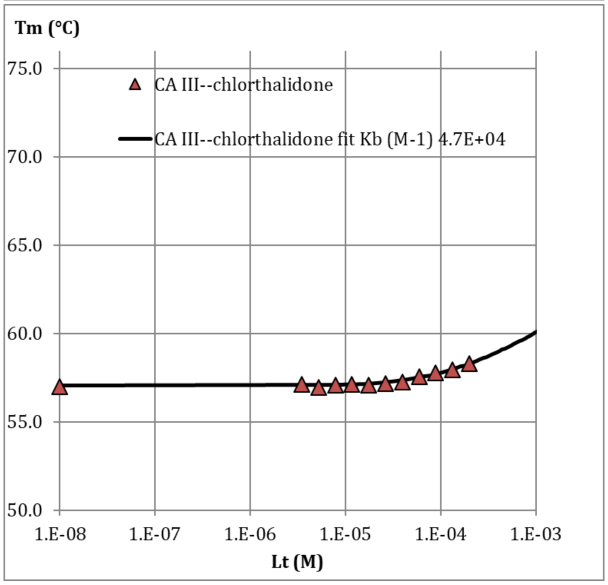

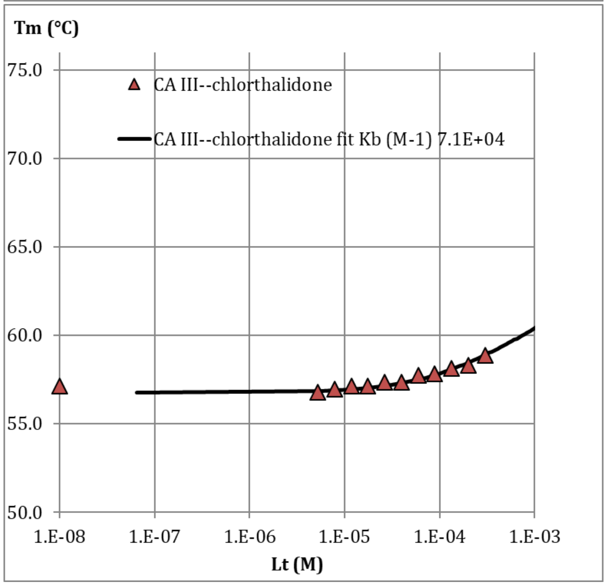

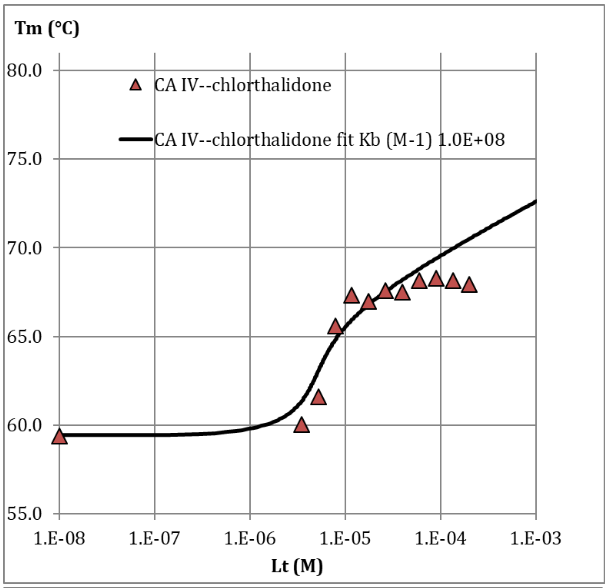
Tm (°C)
CA IV--chlorthalidone
CA IV--chlorthalidone fit Kb (M-1) 1.0E+08
Lt (M)

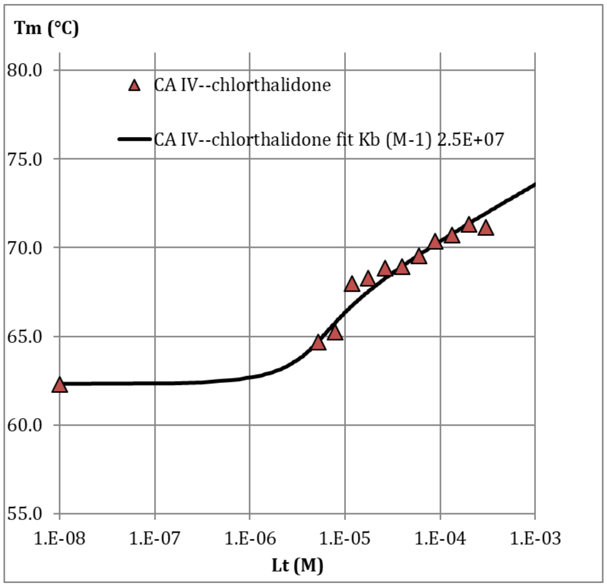
Tm (°C)
CA IV--chlorthalidone
CA IV--chlorthalidone fit Kb (M-1) 2.5E+07
Lt (M)

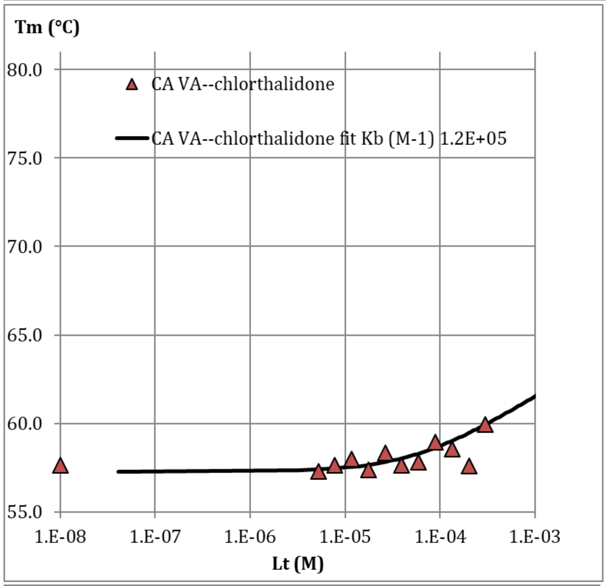
Tm (°C)
CA VA--chlorthalidone
CA VA--chlorthalidone fit Kb (M-1) 1.2E+05
Lt (M)

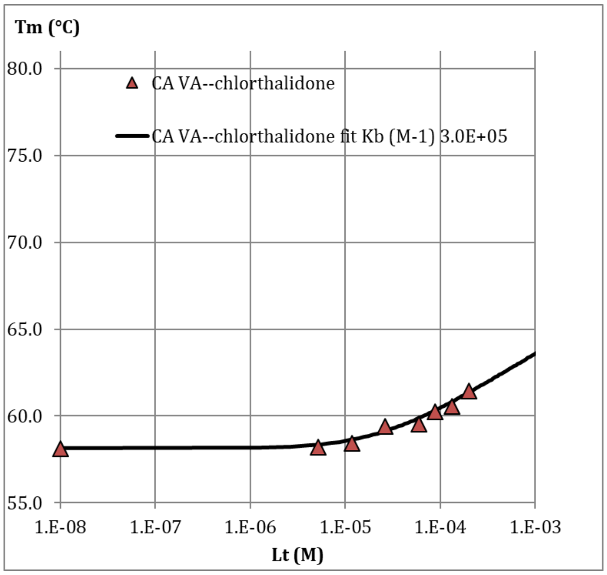
Tm (°C)
CA VA--chlorthalidone
CA VA--chlorthalidone fit Kb (M-1) 3.0E+05
Lt (M)

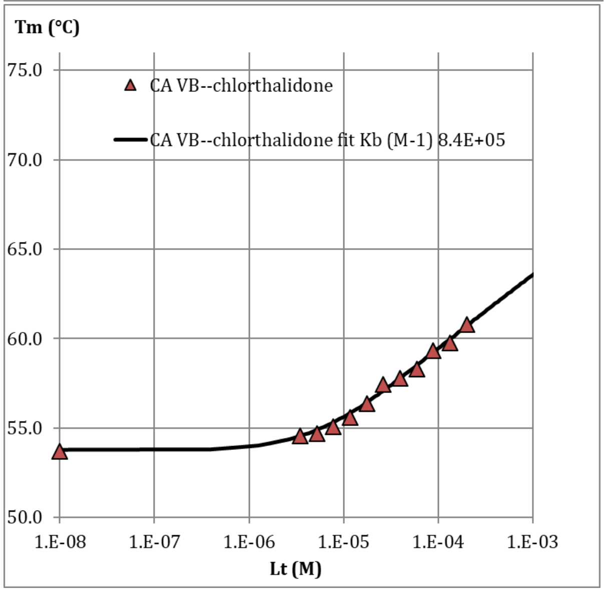
Tm (°C)
CA VB--chlorthalidone
CA VB--chlorthalidone fit Kb (M-1) 8.4E+05
Lt (M)

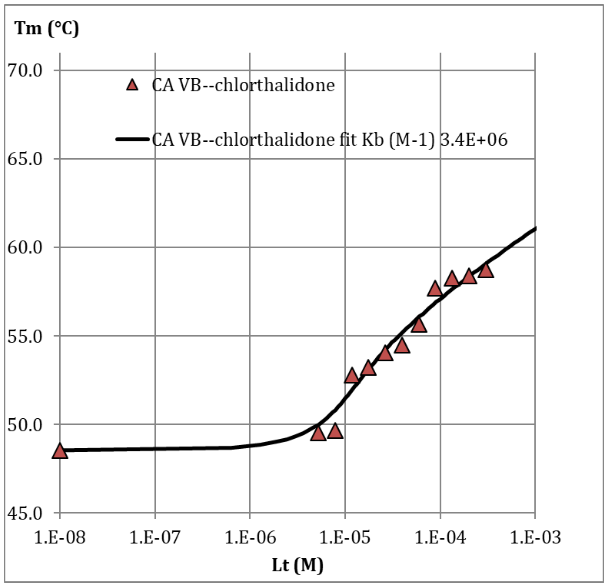
Tm (°C)
CA VB--chlorthalidone
CA VB--chlorthalidone fit Kb (M-1) 3.4E+06
Lt (M)

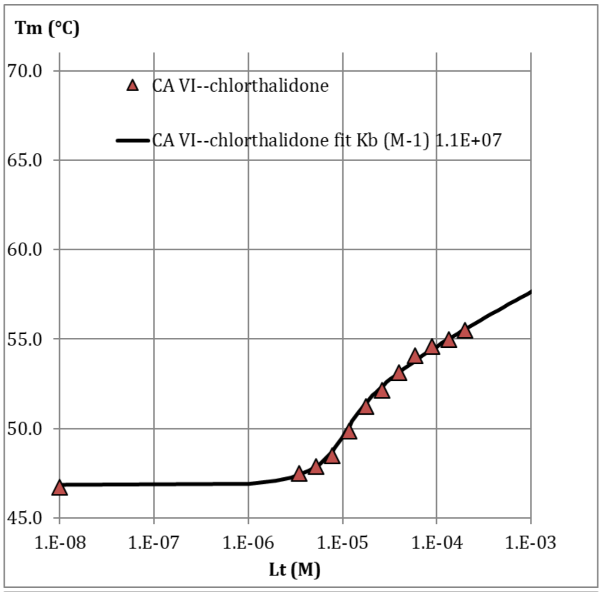
Tm (°C)
70.0
65.0
60.0
55.0
50.0
45.0
CA VI--chlorthalidone
CA VI--chlorthalidone fit Kb (M-1) 1.1E+07
1.E-08
1.E-07
1.E-06
1.E-05
1.E-04
1.E-03
Lt (M)

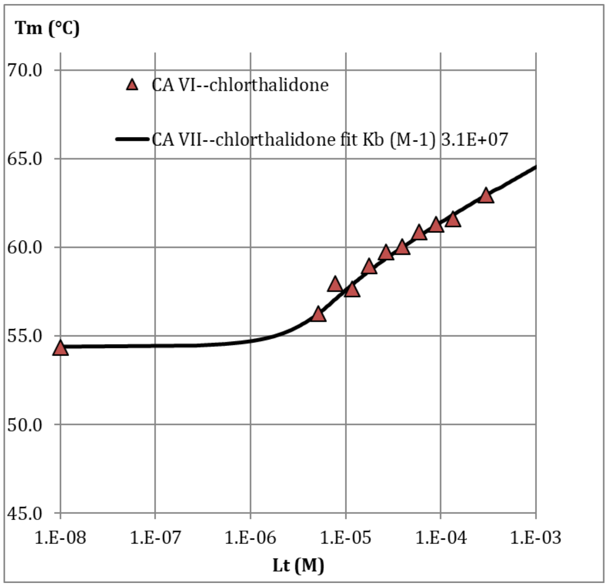
Tm (°C)
70.0
65.0
60.0
55.0
50.0
45.0
CA VI--chlorthalidone
CA VII--chlorthalidone fit Kb (M-1) 3.1E+07
1.E-08
1.E-07
1.E-06
1.E-05
1.E-04
1.E-03
Lt (M)

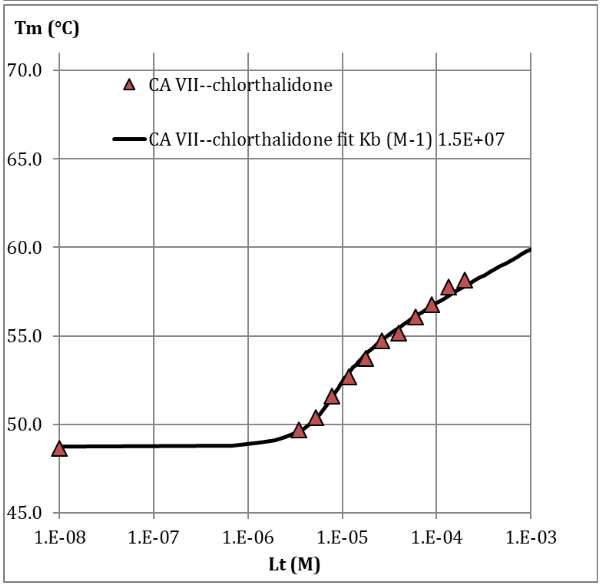
Tm (°C)
70.0
65.0
60.0
55.0
50.0
45.0
CA VII--chlorthalidone
CA VII--chlorthalidone fit Kb (M-1) 1.5E+07
1.E-08
1.E-07
1.E-06
1.E-05
1.E-04
1.E-03
Lt (M)

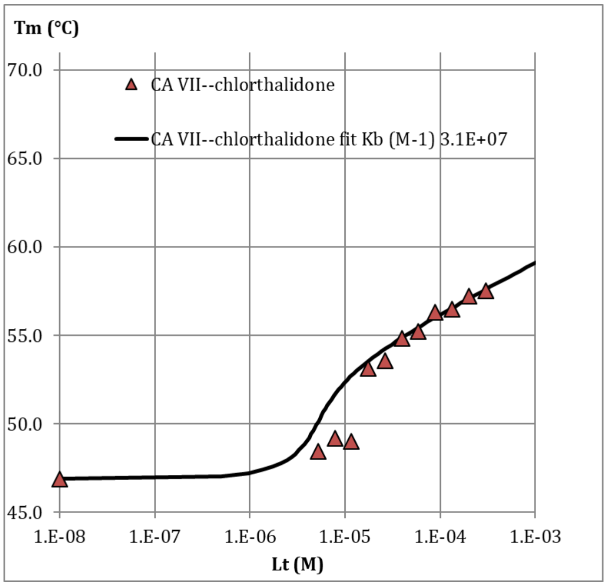
Tm (°C)
70.0
65.0
60.0
55.0
50.0
45.0
CA VII--chlorthalidone
CA VII--chlorthalidone fit Kb (M-1) 3.1E+07
1.E-08
1.E-07
1.E-06
1.E-05
1.E-04
1.E-03
Lt (M)

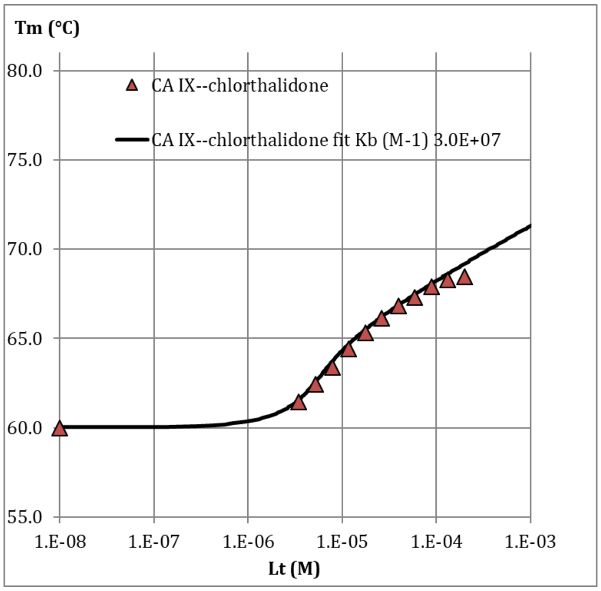
Tm (°C)
80.0
75.0
70.0
65.0
60.0
55.0
CA IX--chlorthalidone
CA IX--chlorthalidone fit Kb (M-1) 3.0E+07
1.E-08
1.E-07
1.E-06
1.E-05
1.E-04
1.E-03
Lt (M)

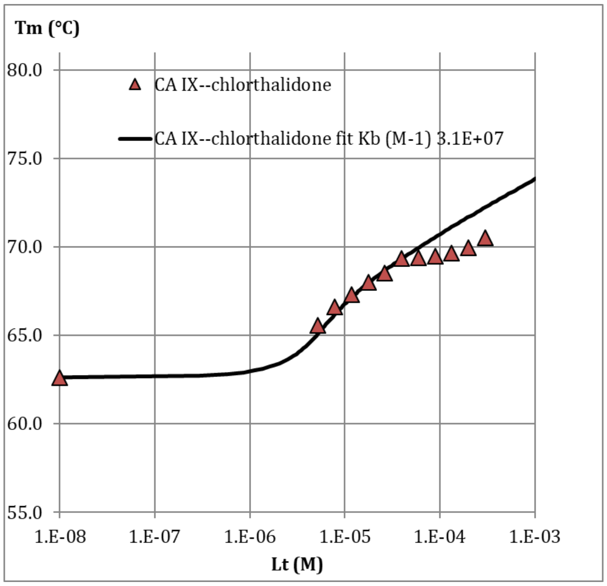
Tm (°C)
80.0
75.0
70.0
65.0
60.0
55.0
CA IX--chlorthalidone
CA IX--chlorthalidone fit Kb (M-1) 3.1E+07
1.E-08
1.E-07
1.E-06
1.E-05
1.E-04
1.E-03
Lt (M)

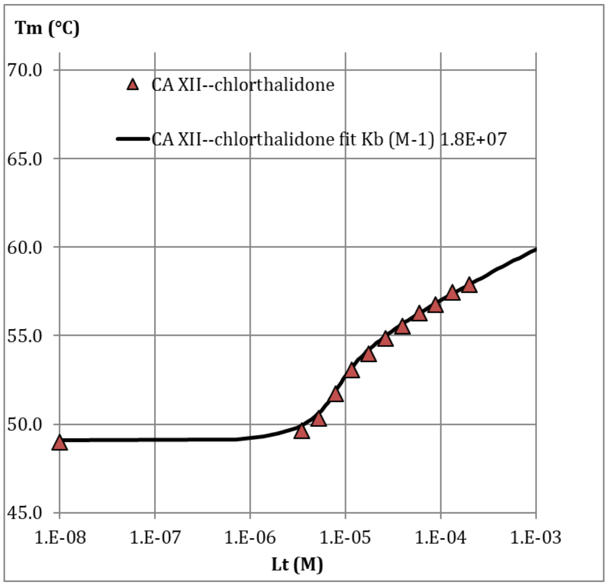
Tm (°C)
CA XII--chlorthalidone
CA XII--chlorthalidone fit Kb (M-1) 1.8E+07
70.0
65.0
60.0
55.0
50.0
45.0
1.E-08
1.E-07
1.E-06
1.E-05
1.E-04
1.E-03
Lt (M)

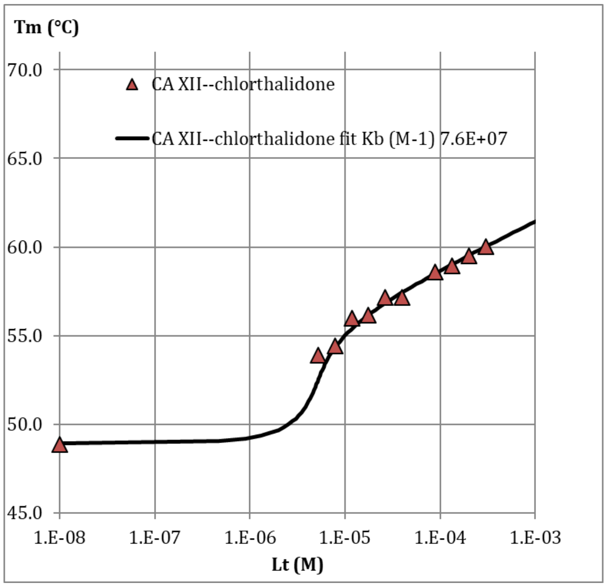
Tm (°C)
CA XII--chlorthalidone
CA XII--chlorthalidone fit Kb (M-1) 7.6E+07
70.0
65.0
60.0
55.0
50.0
45.0
1.E-08
1.E-07
1.E-06
1.E-05
1.E-04
1.E-03
Lt (M)

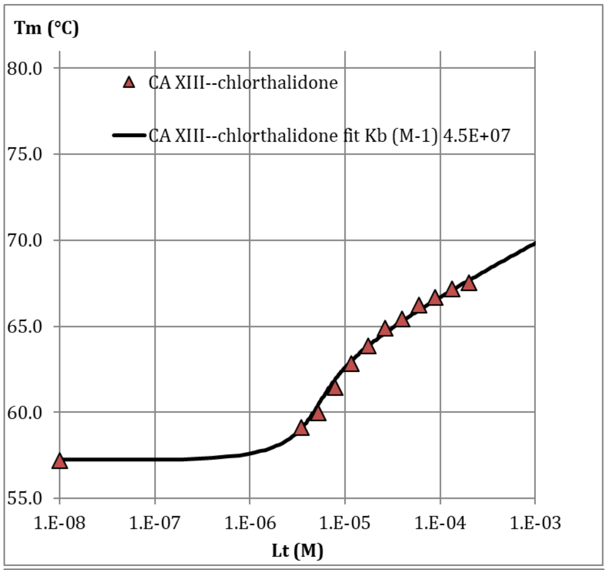
Tm (°C)
CA XIII--chlorthalidone
CA XIII--chlorthalidone fit Kb (M-1) 4.5E+07
80.0
75.0
70.0
65.0
60.0
55.0
1.E-08
1.E-07
1.E-06
1.E-05
1.E-04
1.E-03
Lt (M)

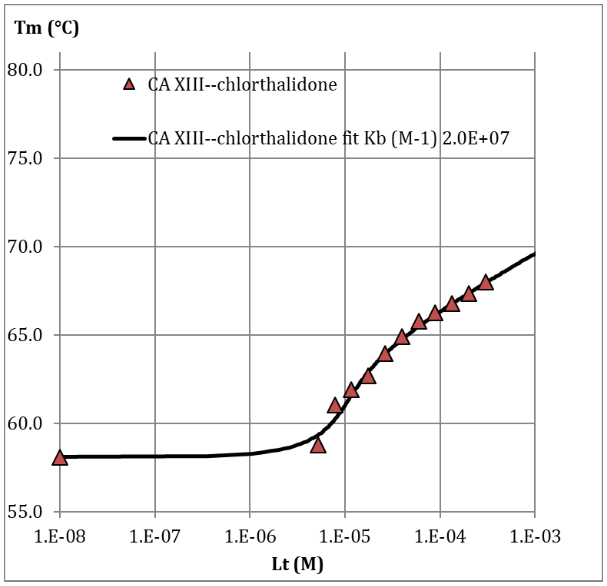
Tm (°C)
CA XIII--chlorthalidone
CA XIII--chlorthalidone fit Kb (M-1) 2.0E+07
80.0
75.0
70.0
65.0
60.0
55.0
1.E-08
1.E-07
1.E-06
1.E-05
1.E-04
1.E-03
Lt (M)

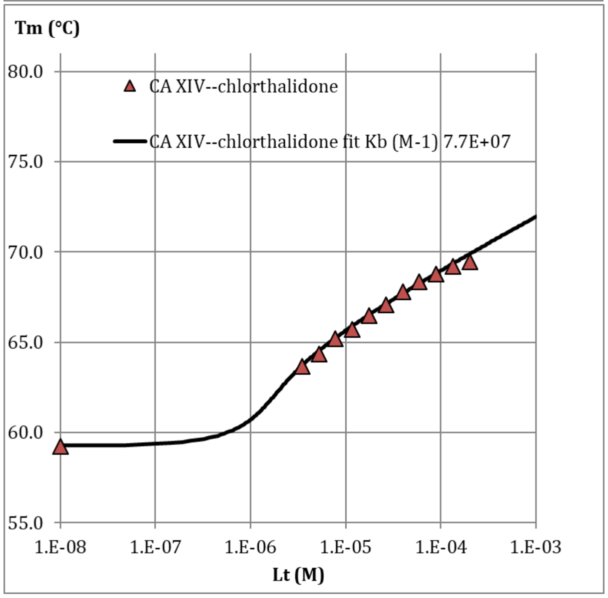
Tm (°C)
CA XIV--chlorthalidone
CA XIV--chlorthalidone fit Kb (M-1) 7.7E+07
80.0
75.0
70.0
65.0
60.0
55.0
1.E-08
1.E-07
1.E-06
1.E-05
1.E-04
1.E-03
Lt (M)

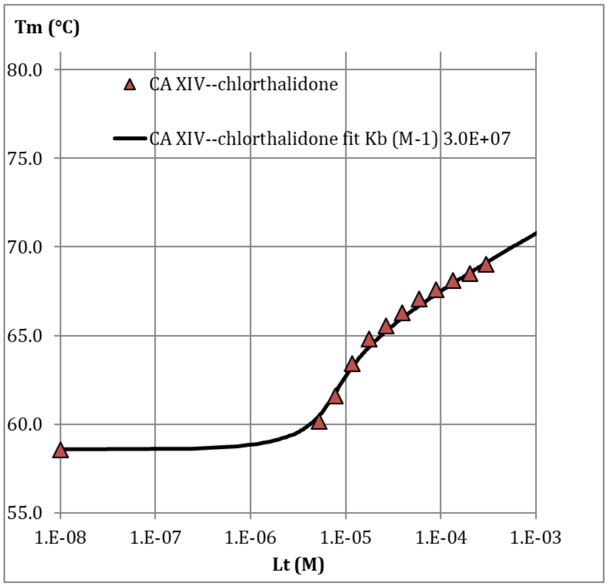
Tm (°C)
CA XIV--chlorthalidone
CA XIV--chlorthalidone fit Kb (M-1) 3.0E+07
80.0
75.0
70.0
65.0
60.0
55.0
1.E-08
1.E-07
1.E-06
1.E-05
1.E-04
1.E-03
Lt (M)

CO_2 hydration activity inhibition

CA XII - chlorthalidone

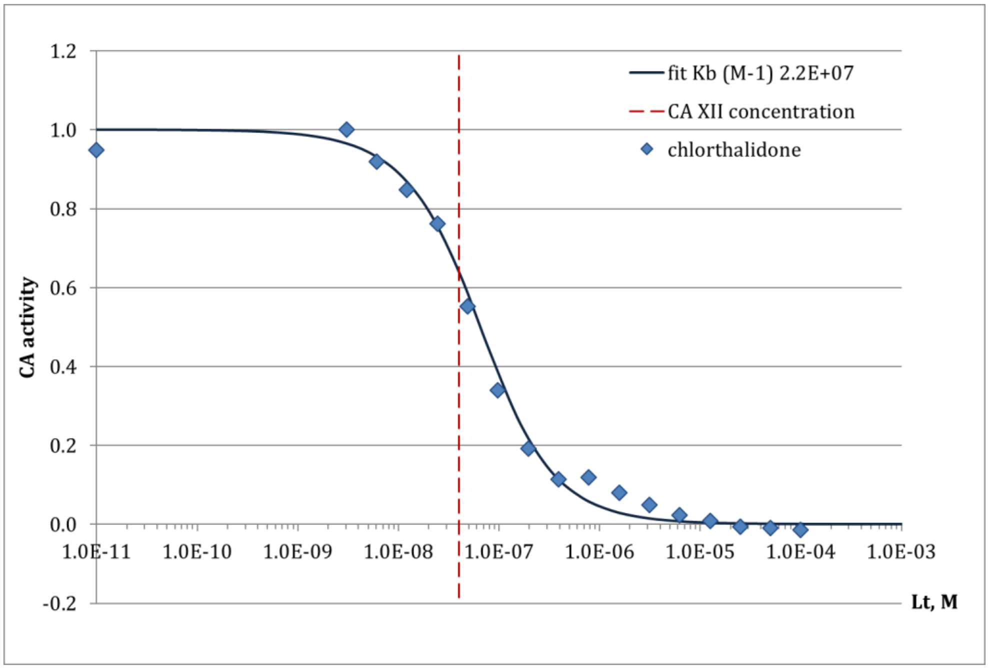

Isothermal titration calorimetry

CA II – chlorthalidone

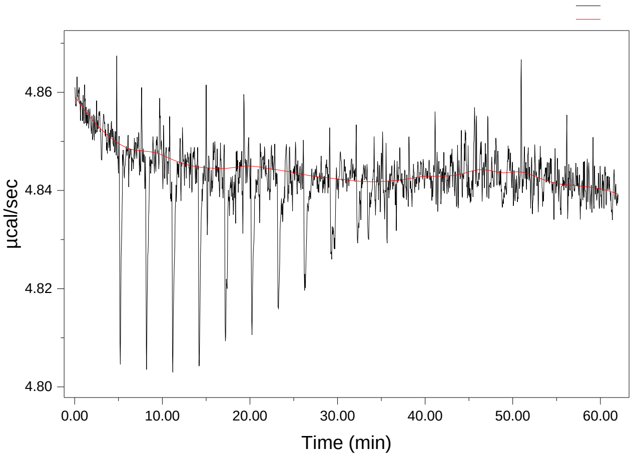

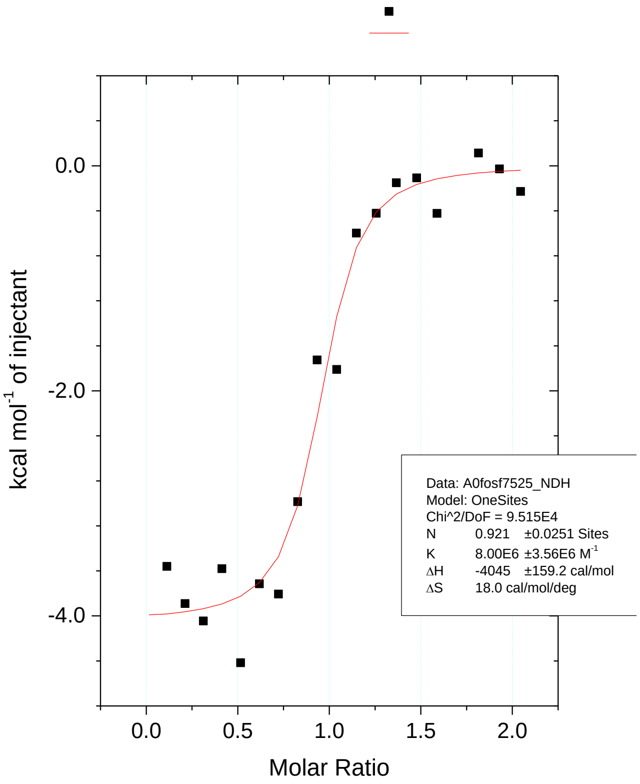

CA II – chlorthalidone

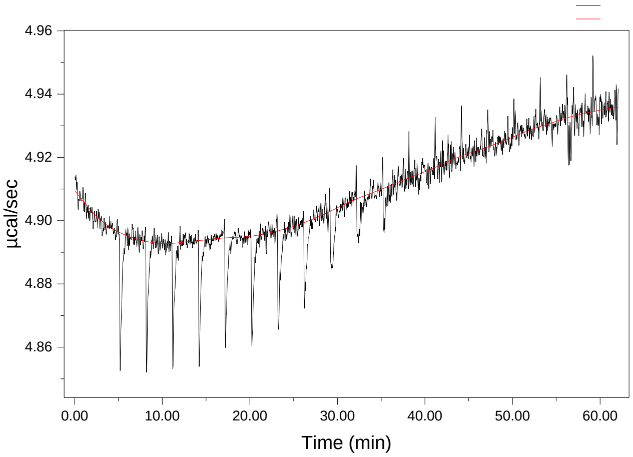

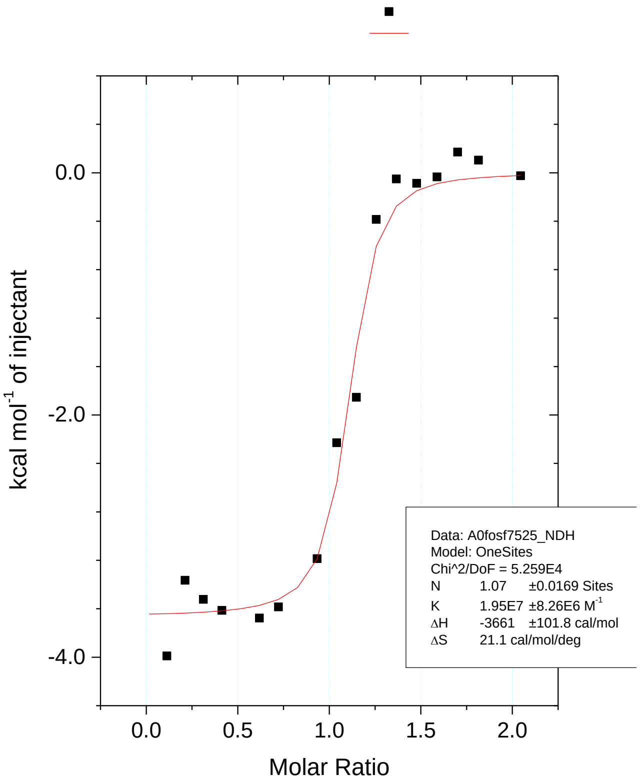

17. Clorexolone

Fluorecence-based thermal shift assay

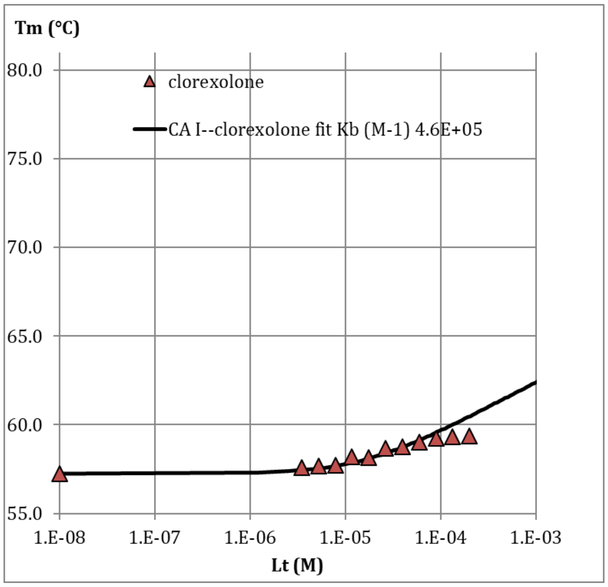

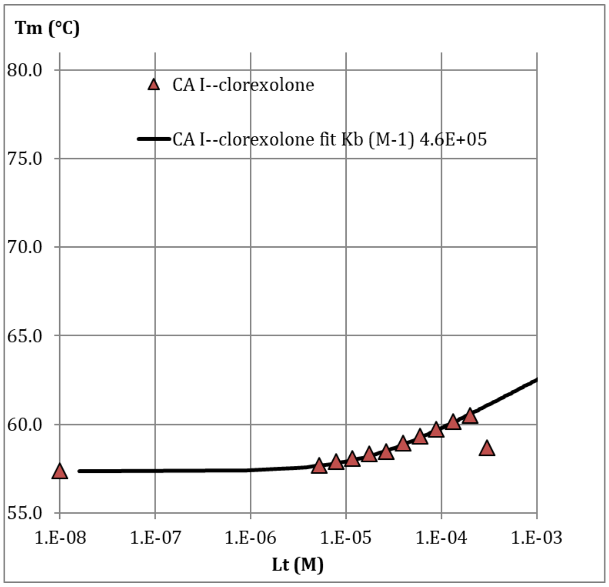

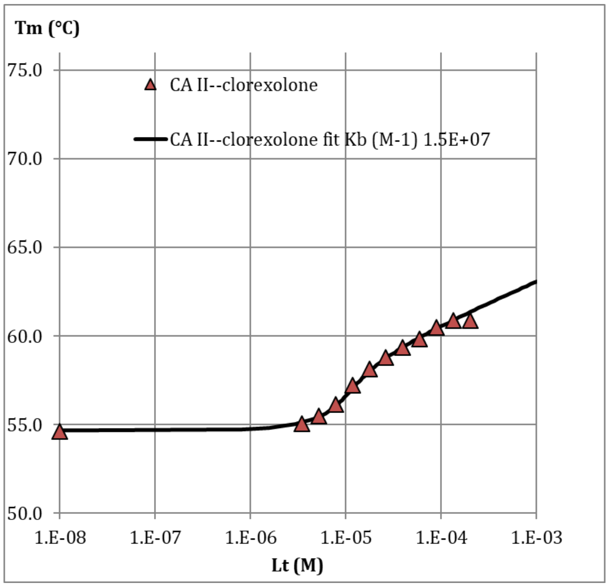

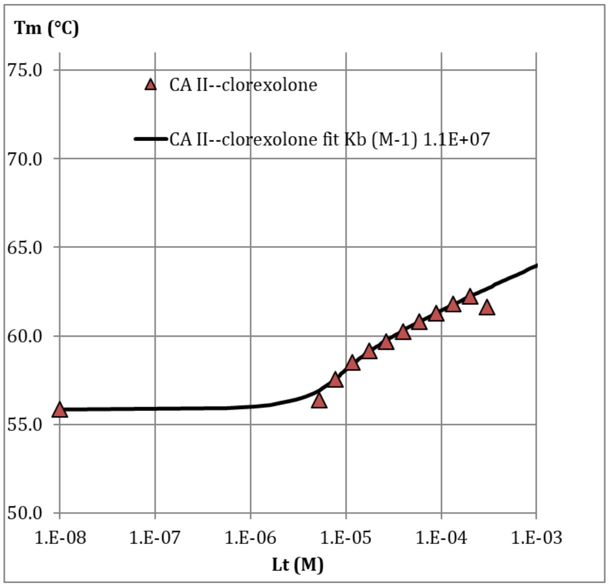

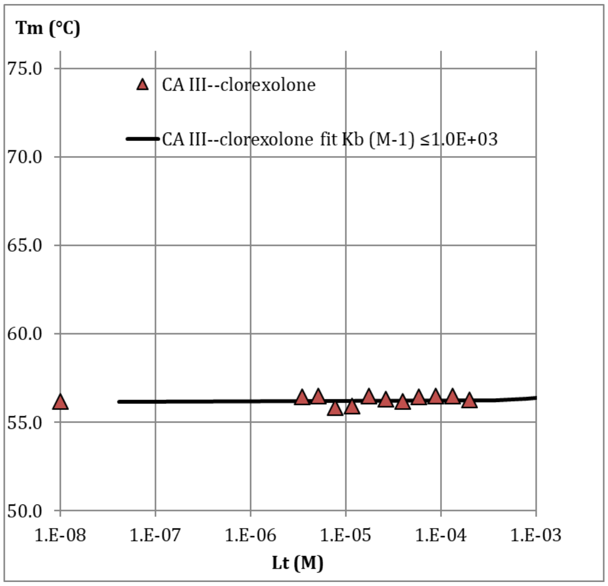

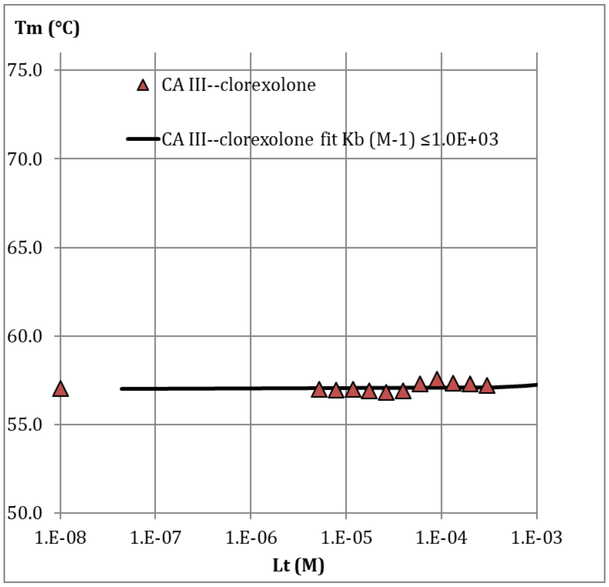

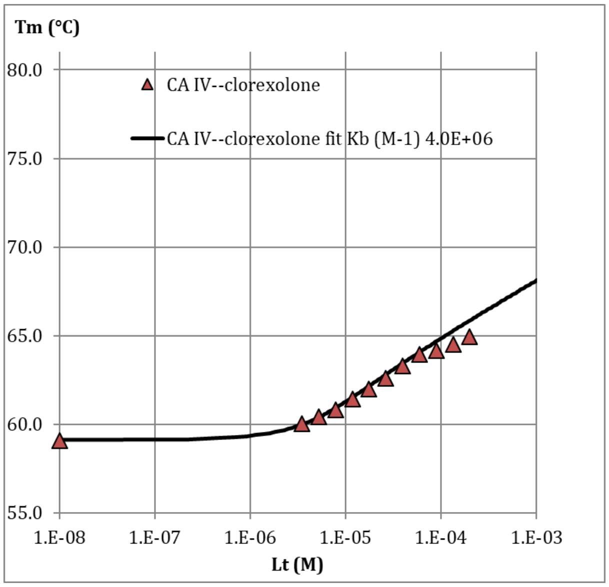
Tm (°C)
80.0
75.0
70.0
65.0
60.0
55.0
CA IV--clorexolone
CA IV--clorexolone fit Kb (M-1) 4.0E+06
1.E-08
1.E-07
1.E-06
1.E-05
1.E-04
1.E-03
Lt (M)

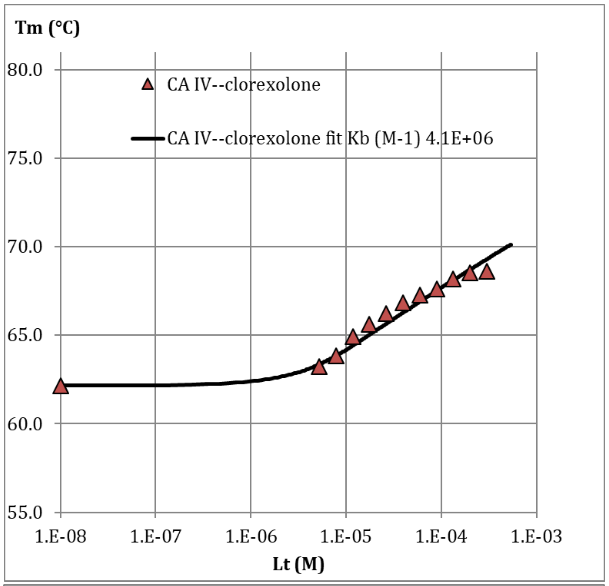
Tm (°C)
80.0
75.0
70.0
65.0
60.0
55.0
CA IV--clorexolone
CA IV--clorexolone fit Kb (M-1) 4.1E+06
1.E-08
1.E-07
1.E-06
1.E-05
1.E-04
1.E-03
Lt (M)

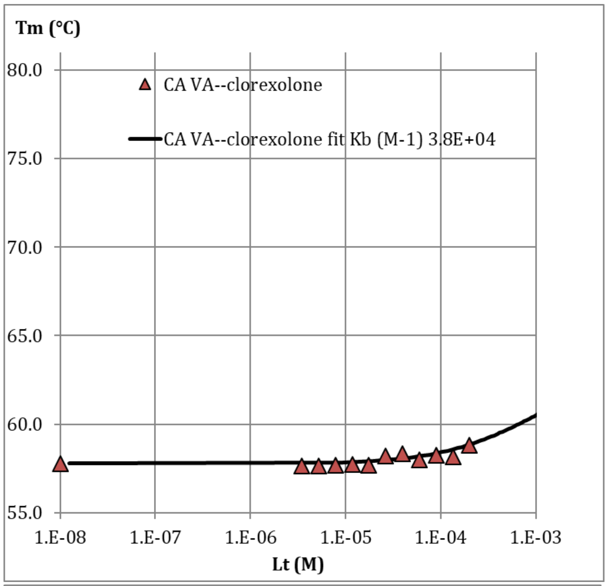
Tm (°C)
80.0
75.0
70.0
65.0
60.0
55.0
CA VA--clorexolone
CA VA--clorexolone fit Kb (M-1) 3.8E+04
1.E-08
1.E-07
1.E-06
1.E-05
1.E-04
1.E-03
Lt (M)

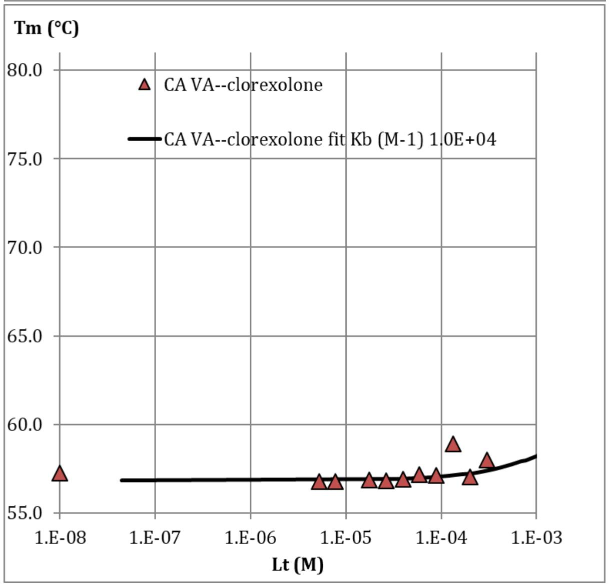
Tm (°C)
80.0
75.0
70.0
65.0
60.0
55.0
CA VA--clorexolone
CA VA--clorexolone fit Kb (M-1) 1.0E+04
1.E-08
1.E-07
1.E-06
1.E-05
1.E-04
1.E-03
Lt (M)

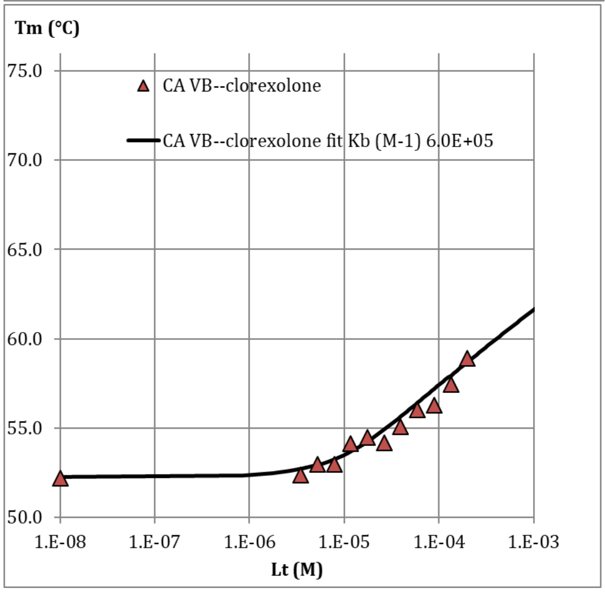
Tm (°C)
75.0
70.0
65.0
60.0
55.0
50.0
CA VB--clorexolone
CA VB--clorexolone fit Kb (M-1) 6.0E+05
1.E-08
1.E-07
1.E-06
1.E-05
1.E-04
1.E-03
Lt (M)

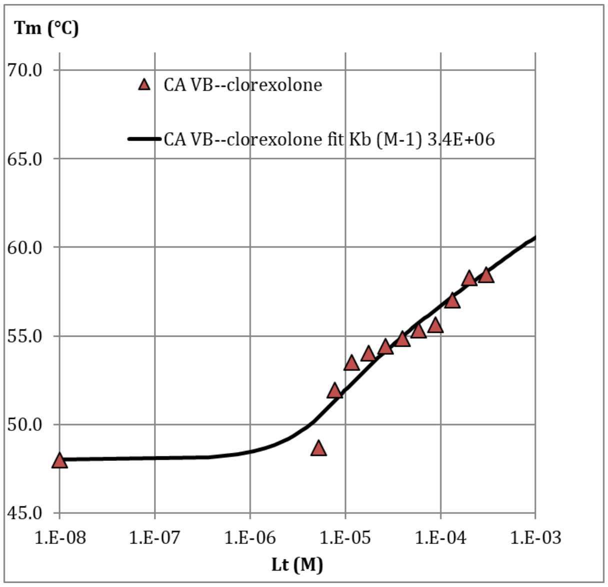
Tm (°C)
70.0
65.0
60.0
55.0
50.0
45.0
CA VB--clorexolone
CA VB--clorexolone fit Kb (M-1) 3.4E+06
1.E-08
1.E-07
1.E-06
1.E-05
1.E-04
1.E-03
Lt (M)

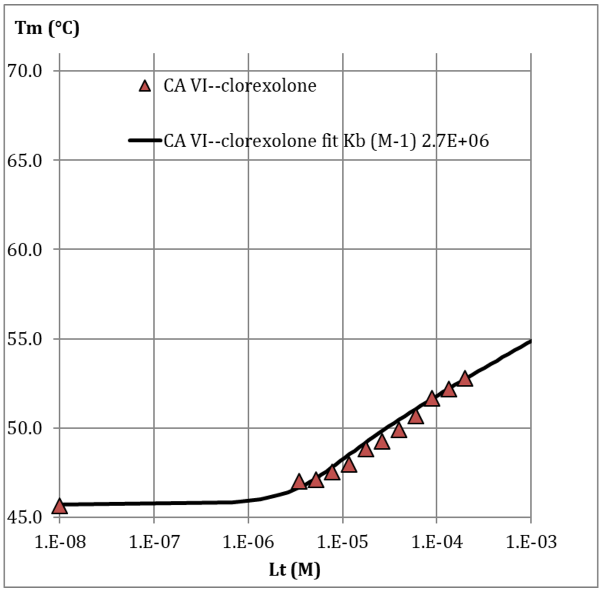
Tm (°C)
70.0
65.0
60.0
55.0
50.0
45.0
CA VI--clorexolone
CA VI--clorexolone fit Kb (M-1) 2.7E+06
1.E-08
1.E-07
1.E-06
1.E-05
1.E-04
1.E-03
Lt (M)

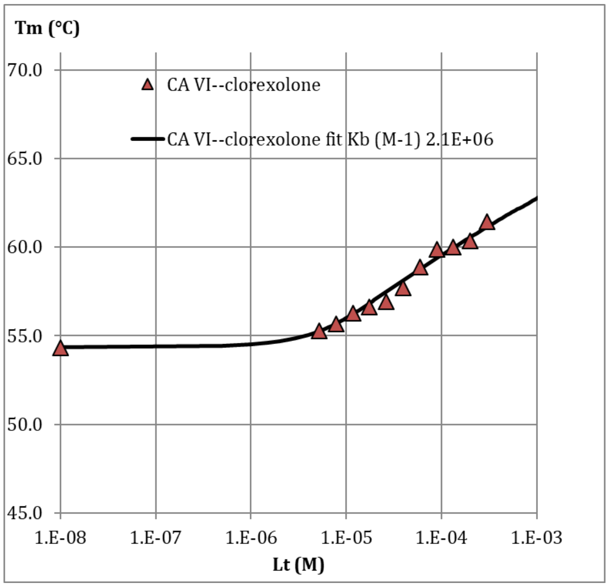
Tm (°C)
70.0
65.0
60.0
55.0
50.0
45.0
CA VI--clorexolone
CA VI--clorexolone fit Kb (M-1) 2.1E+06
1.E-08
1.E-07
1.E-06
1.E-05
1.E-04
1.E-03
Lt (M)

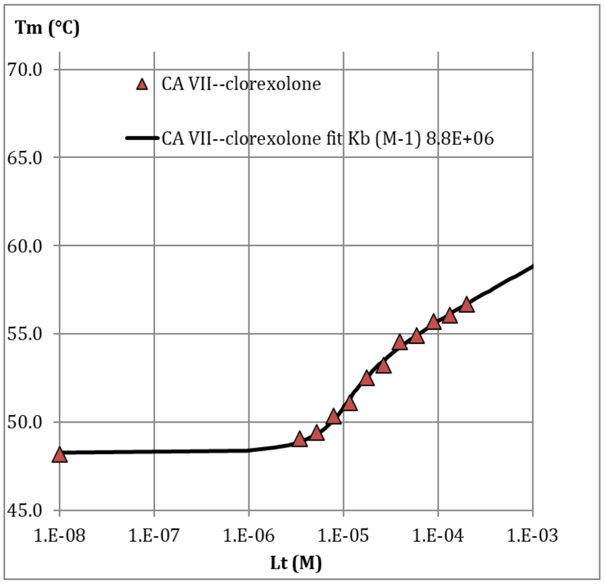
Tm (°C)
70.0
65.0
60.0
55.0
50.0
45.0
CA VII--clorexolone
CA VII--clorexolone fit Kb (M-1) 8.8E+06
1.E-08
1.E-07
1.E-06
1.E-05
1.E-04
1.E-03
Lt (M)

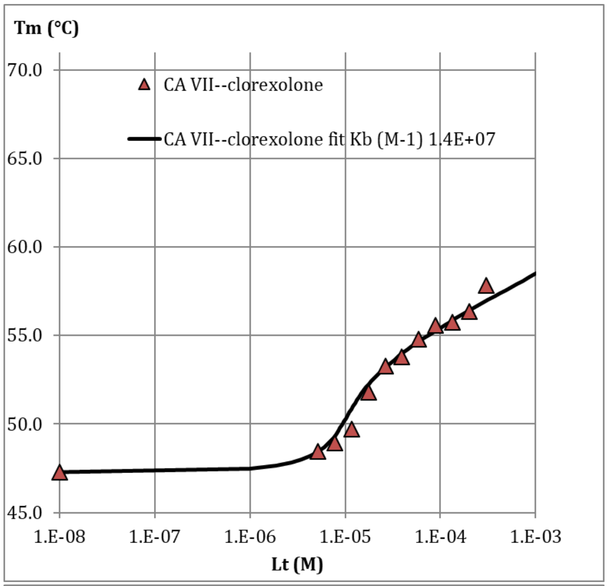
Tm (°C)
70.0
65.0
60.0
55.0
50.0
45.0
CA VII--clorexolone
CA VII--clorexolone fit Kb (M-1) 1.4E+07
1.E-08
1.E-07
1.E-06
1.E-05
1.E-04
1.E-03
Lt (M)

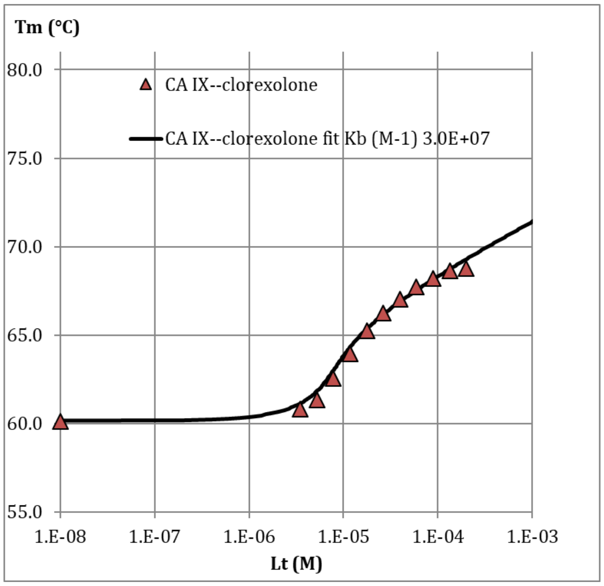
Tm (°C)
80.0
75.0
70.0
65.0
60.0
55.0
CA IX--clorexolone
CA IX--clorexolone fit Kb (M-1) 3.0E+07
1.E-08
1.E-07
1.E-06
1.E-05
1.E-04
1.E-03
Lt (M)

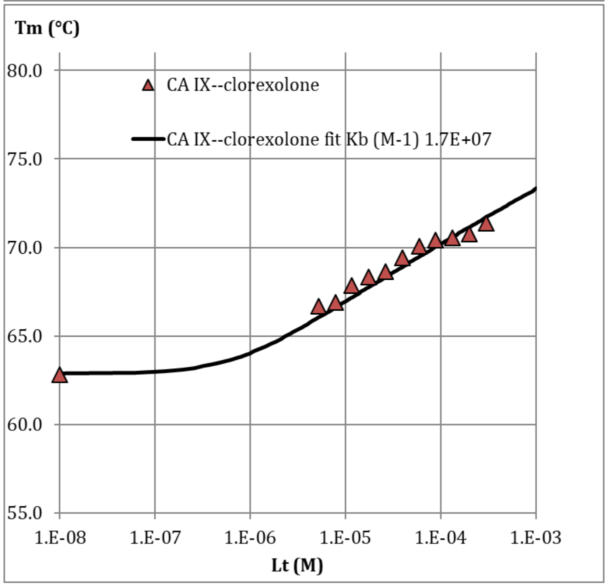
Tm (°C)
80.0
75.0
70.0
65.0
60.0
55.0
CA IX--clorexolone
CA IX--clorexolone fit Kb (M-1) 1.7E+07
1.E-08
1.E-07
1.E-06
1.E-05
1.E-04
1.E-03
Lt (M)

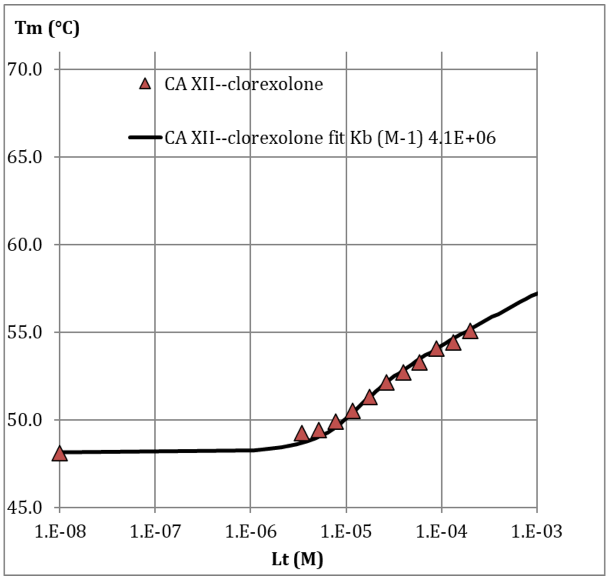
Tm (°C)
CA XII--clorexolone
CA XII--clorexolone fit Kb (M-1) 4.1E+06
70.0
65.0
60.0
55.0
50.0
45.0
1.E-08
1.E-07
1.E-06
1.E-05
1.E-04
1.E-03
Lt (M)

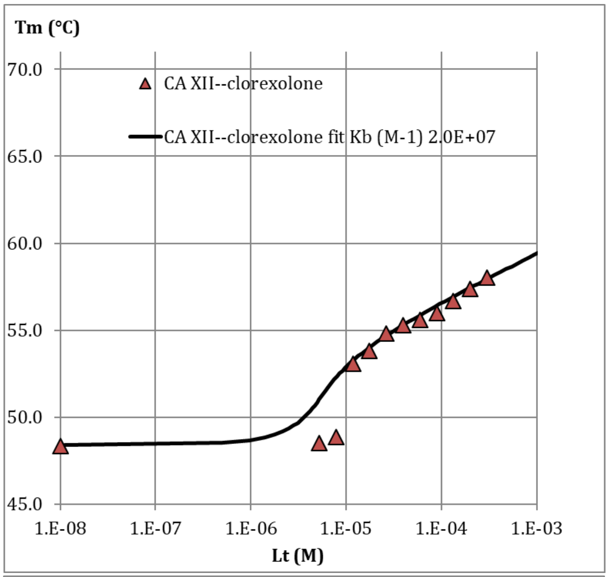
Tm (°C)
CA XII--clorexolone
CA XII--clorexolone fit Kb (M-1) 2.0E+07
70.0
65.0
60.0
55.0
50.0
45.0
1.E-08
1.E-07
1.E-06
1.E-05
1.E-04
1.E-03
Lt (M)

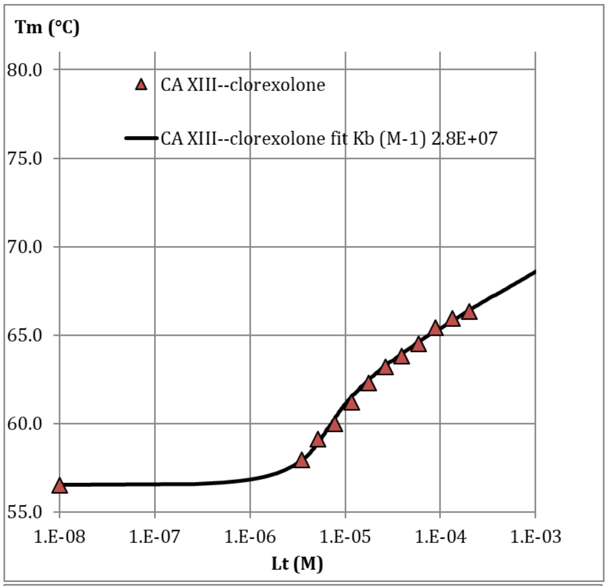
Tm (°C)
CA XIII--clorexolone
CA XIII--clorexolone fit Kb (M-1) 2.8E+07
80.0
75.0
70.0
65.0
60.0
55.0
1.E-08
1.E-07
1.E-06
1.E-05
1.E-04
1.E-03
Lt (M)

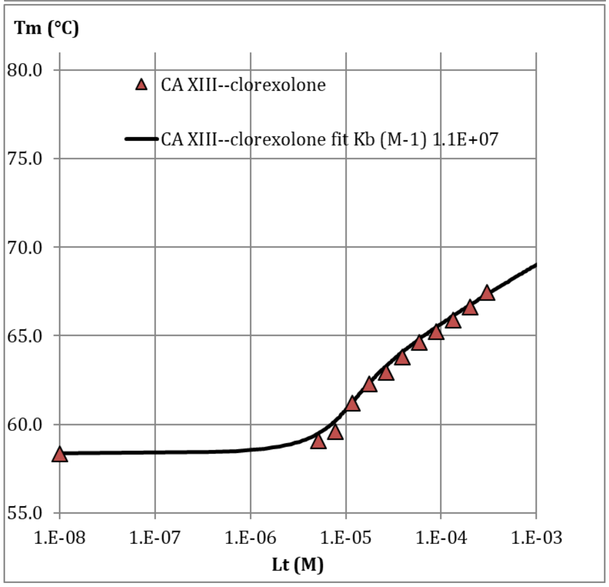
Tm (°C)
CA XIII--clorexolone
CA XIII--clorexolone fit Kb (M-1) 1.1E+07
80.0
75.0
70.0
65.0
60.0
55.0
1.E-08
1.E-07
1.E-06
1.E-05
1.E-04
1.E-03
Lt (M)

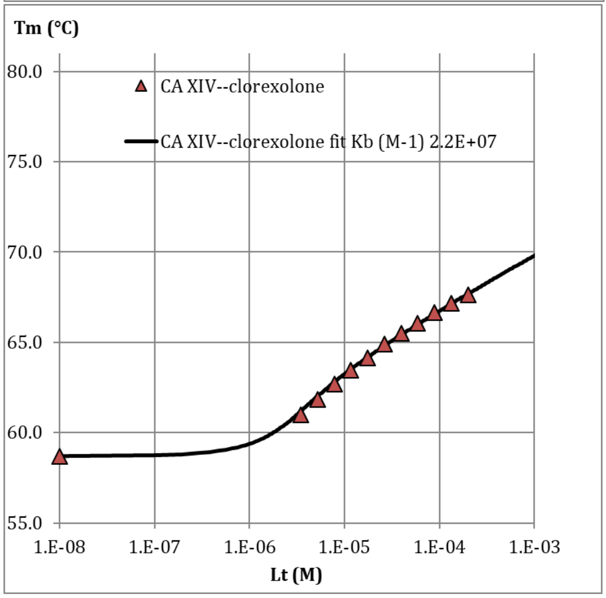
Tm (°C)
CA XIV--clorexolone
CA XIV--clorexolone fit Kb (M-1) 2.2E+07
80.0
75.0
70.0
65.0
60.0
55.0
1.E-08
1.E-07
1.E-06
1.E-05
1.E-04
1.E-03
Lt (M)

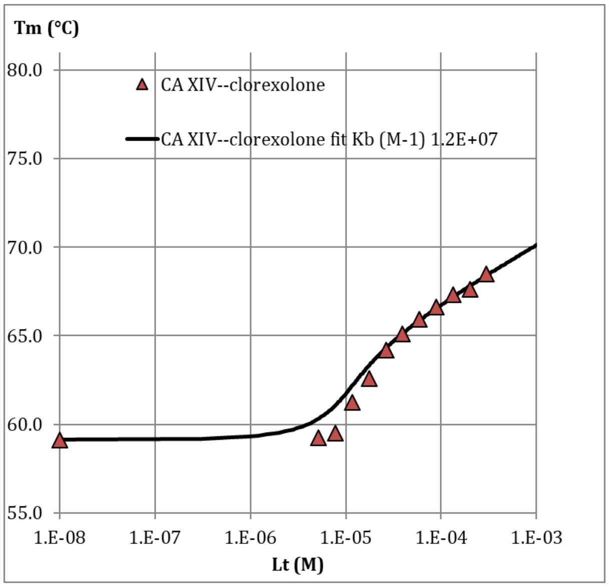
Tm (°C)
CA XIV--clorexolone
CA XIV--clorexolone fit Kb (M-1) 1.2E+07
80.0
75.0
70.0
65.0
60.0
55.0
1.E-08
1.E-07
1.E-06
1.E-05
1.E-04
1.E-03
Lt (M)

18. Quinethazone

Fluorecence-based thermal shift assay

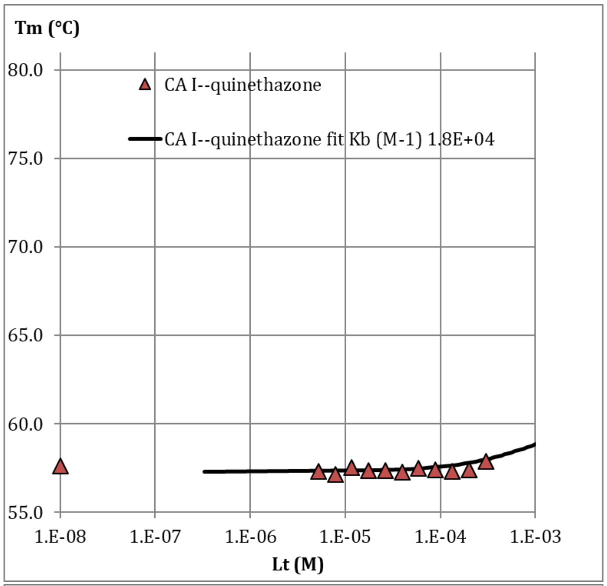

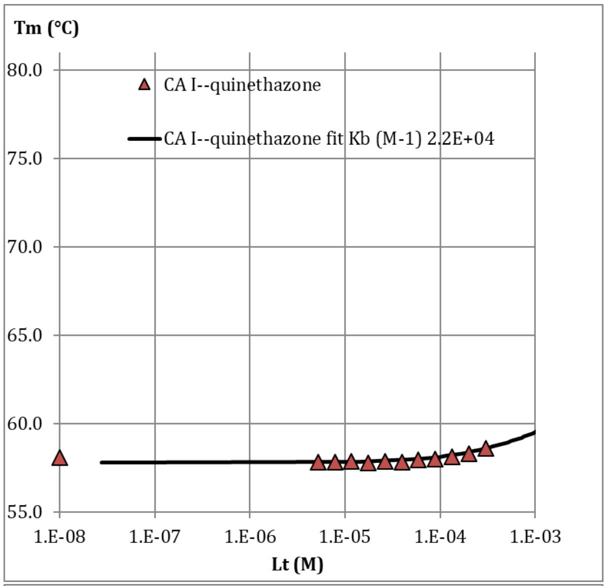

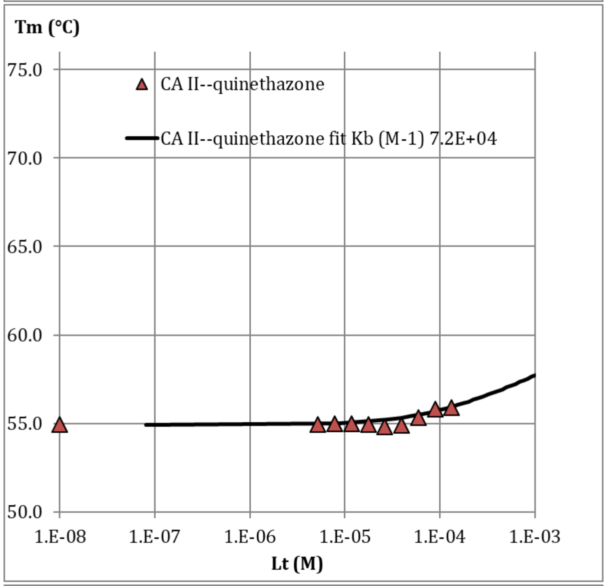

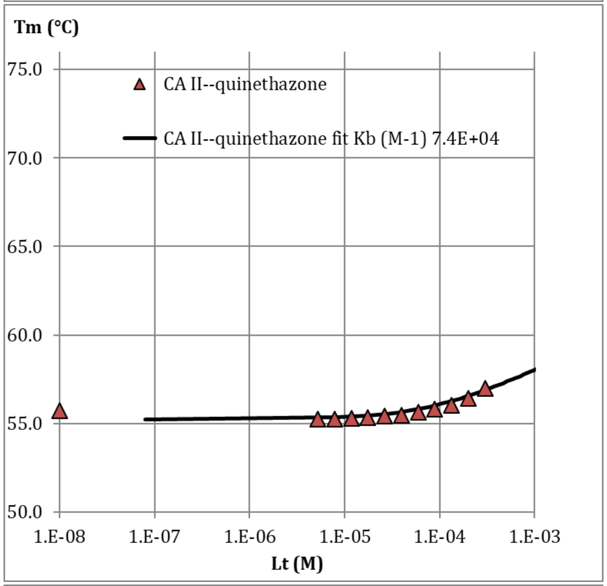

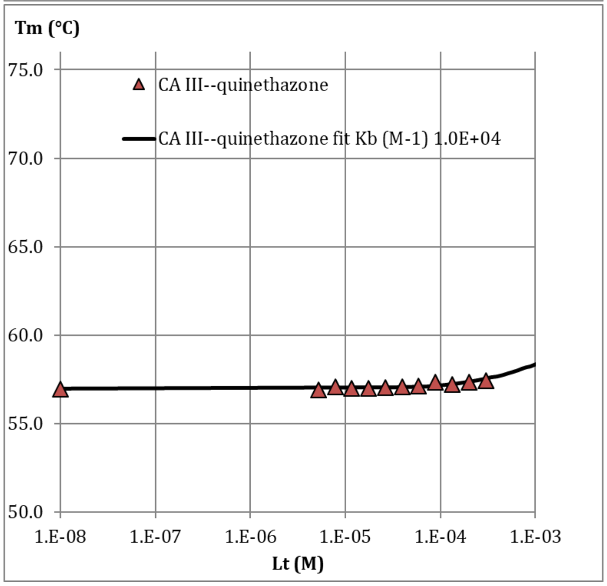

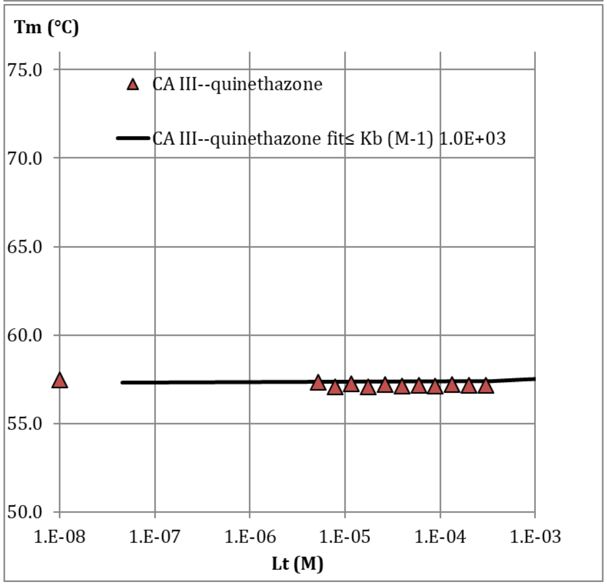

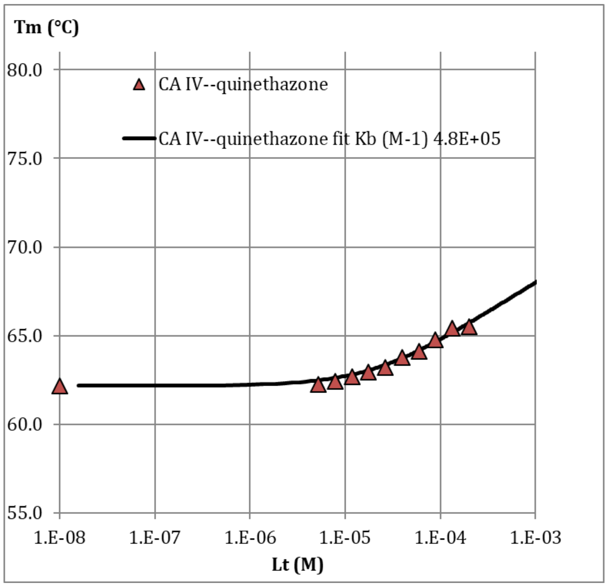
Tm (°C)
80.0
75.0
70.0
65.0
60.0
55.0
CA IV--quinethazone
CA IV--quinethazone fit Kb (M-1) 4.8E+05
1.E-08
1.E-07
1.E-06
1.E-05
1.E-04
1.E-03
Lt (M)

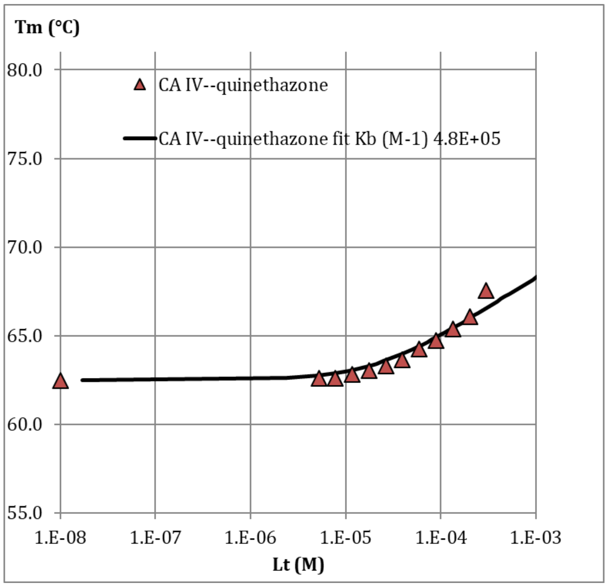
Tm (°C)
80.0
75.0
70.0
65.0
60.0
55.0
CA IV--quinethazone
CA IV--quinethazone fit Kb (M-1) 4.8E+05
1.E-08
1.E-07
1.E-06
1.E-05
1.E-04
1.E-03
Lt (M)

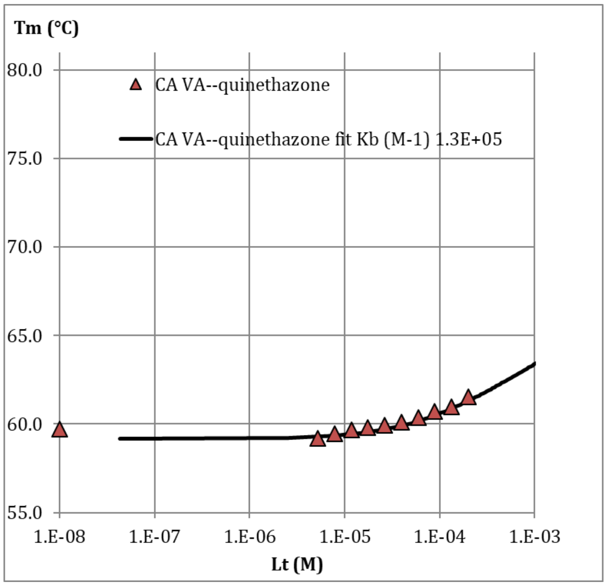
Tm (°C)
80.0
75.0
70.0
65.0
60.0
55.0
CA VA--quinethazone
CA VA--quinethazone fit Kb (M-1) 1.3E+05
1.E-08
1.E-07
1.E-06
1.E-05
1.E-04
1.E-03
Lt (M)

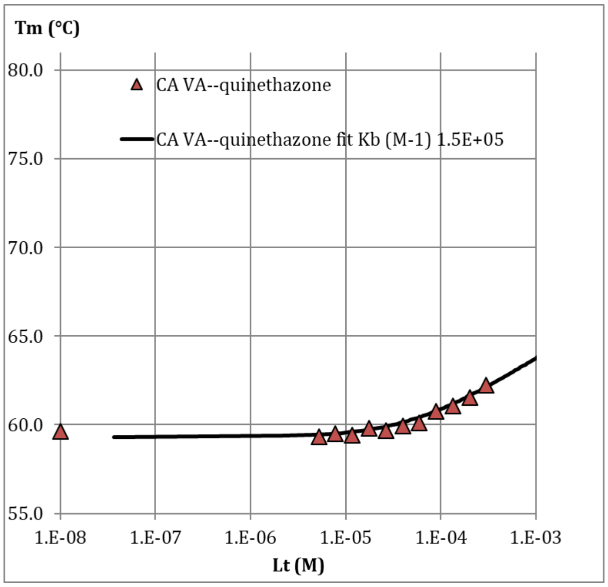
Tm (°C)
80.0
75.0
70.0
65.0
60.0
55.0
CA VA--quinethazone
CA VA--quinethazone fit Kb (M-1) 1.5E+05
1.E-08
1.E-07
1.E-06
1.E-05
1.E-04
1.E-03
Lt (M)

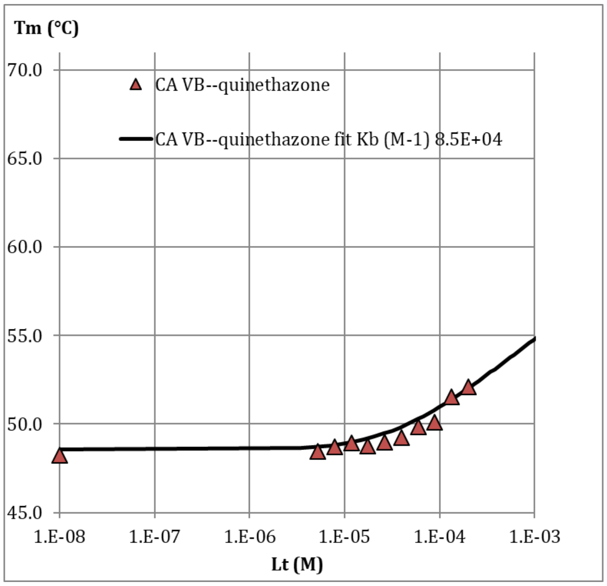
Tm (°C)
70.0
65.0
60.0
55.0
50.0
45.0
CA VB--quinethazone
CA VB--quinethazone fit Kb (M-1) 8.5E+04
1.E-08
1.E-07
1.E-06
1.E-05
1.E-04
1.E-03
Lt (M)

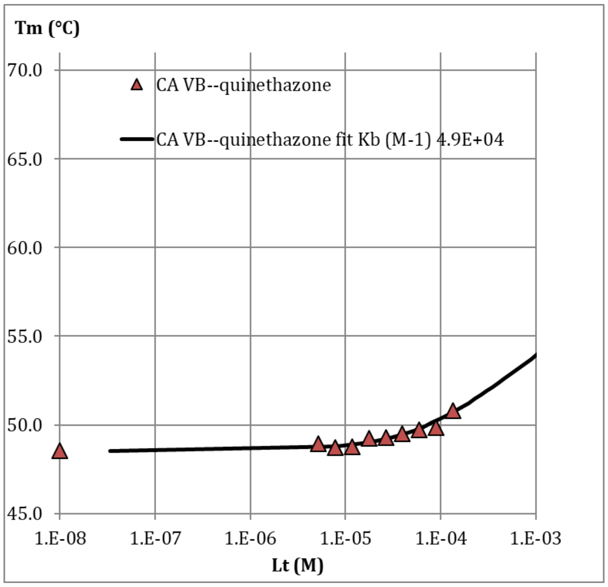
Tm (°C)
70.0
65.0
60.0
55.0
50.0
45.0
CA VB--quinethazone
CA VB--quinethazone fit Kb (M-1) 4.9E+04
1.E-08
1.E-07
1.E-06
1.E-05
1.E-04
1.E-03
Lt (M)

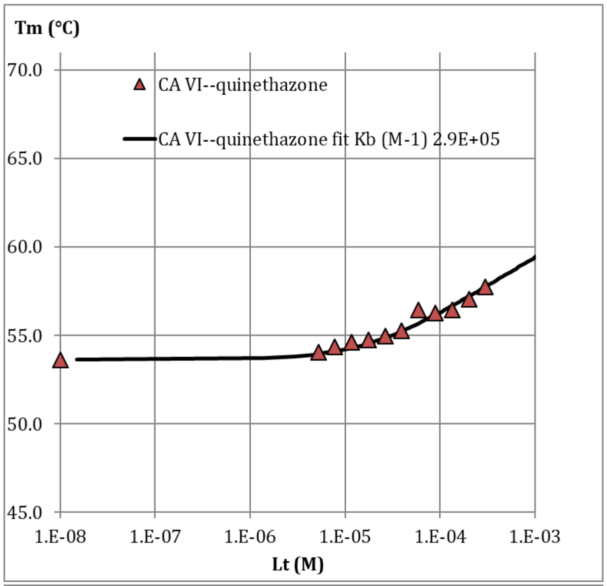
Tm (°C)
CA VI--quinethazone
CA VI--quinethazone fit Kb (M-1) 2.9E+05
70.0
65.0
60.0
55.0
50.0
45.0
1.E-08
1.E-07
1.E-06
1.E-05
1.E-04
1.E-03
Lt (M)

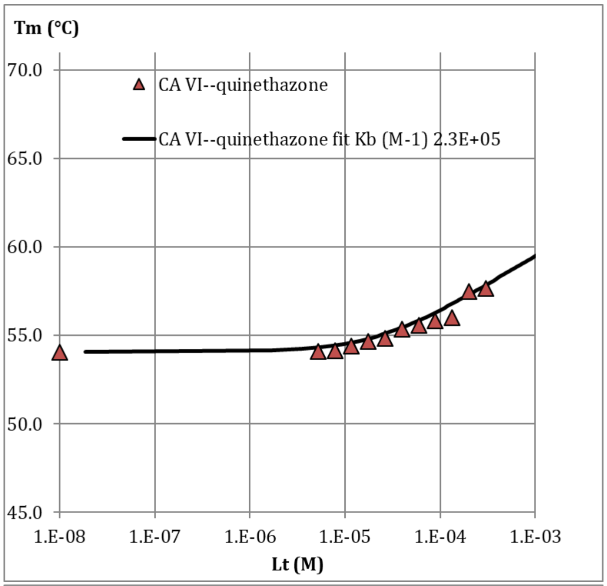
Tm (°C)
CA VI--quinethazone
CA VI--quinethazone fit Kb (M-1) 2.3E+05
70.0
65.0
60.0
55.0
50.0
45.0
1.E-08
1.E-07
1.E-06
1.E-05
1.E-04
1.E-03
Lt (M)

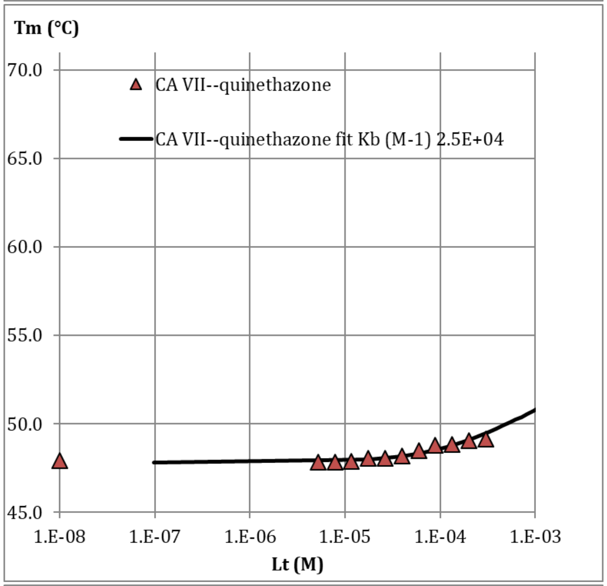
Tm (°C)
CA VII--quinethazone
CA VII--quinethazone fit Kb (M-1) 2.5E+04
70.0
65.0
60.0
55.0
50.0
45.0
1.E-08
1.E-07
1.E-06
1.E-05
1.E-04
1.E-03
Lt (M)

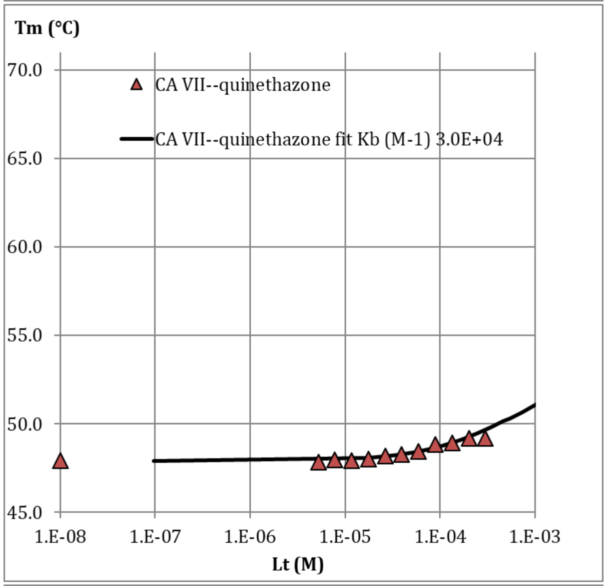
Tm (°C)
CA VII--quinethazone
CA VII--quinethazone fit Kb (M-1) 3.0E+04
70.0
65.0
60.0
55.0
50.0
45.0
1.E-08
1.E-07
1.E-06
1.E-05
1.E-04
1.E-03
Lt (M)

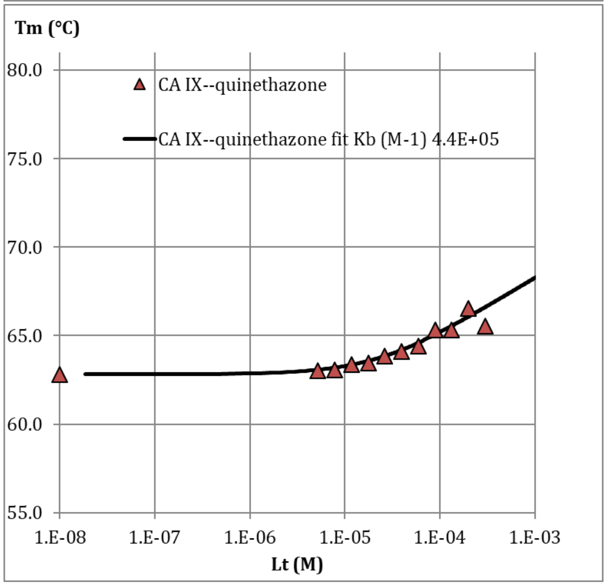
Tm (°C)
CA IX--quinethazone
CA IX--quinethazone fit Kb (M-1) 4.4E+05
80.0
75.0
70.0
65.0
60.0
55.0
1.E-08
1.E-07
1.E-06
1.E-05
1.E-04
1.E-03
Lt (M)

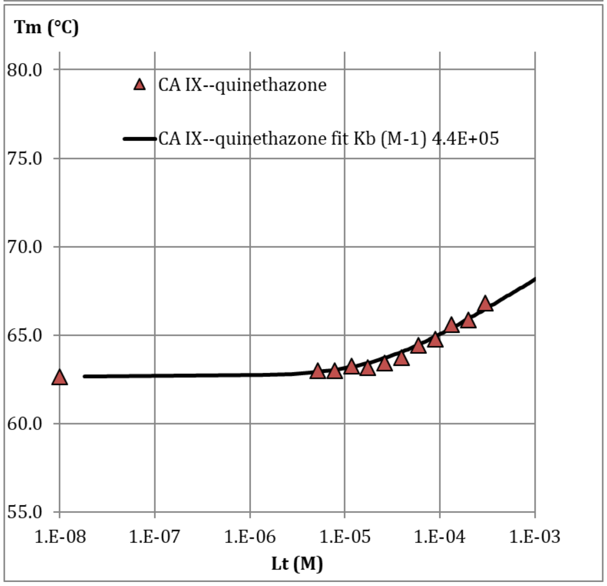
Tm (°C)
CA IX--quinethazone
CA IX--quinethazone fit Kb (M-1) 4.4E+05
80.0
75.0
70.0
65.0
60.0
55.0
1.E-08
1.E-07
1.E-06
1.E-05
1.E-04
1.E-03
Lt (M)

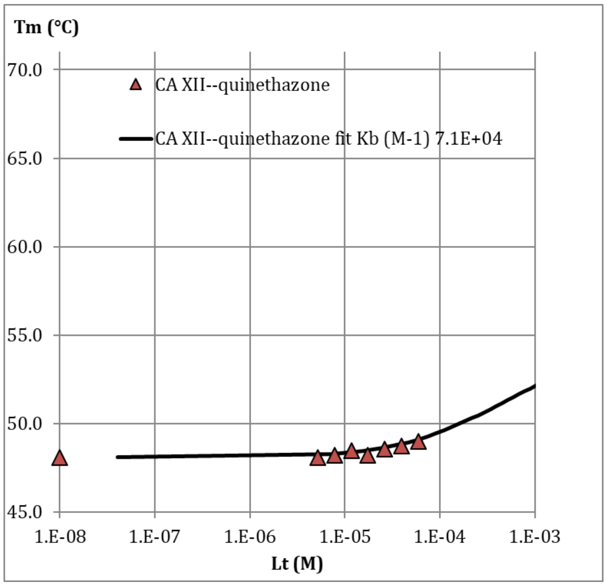
Tm (°C)
70.0
65.0
60.0
55.0
50.0
45.0
CA XII--quinethazone
CA XII--quinethazone fit Kb (M-1) 7.1E+04
1.E-08
1.E-07
1.E-06
1.E-05
1.E-04
1.E-03
Lt (M)

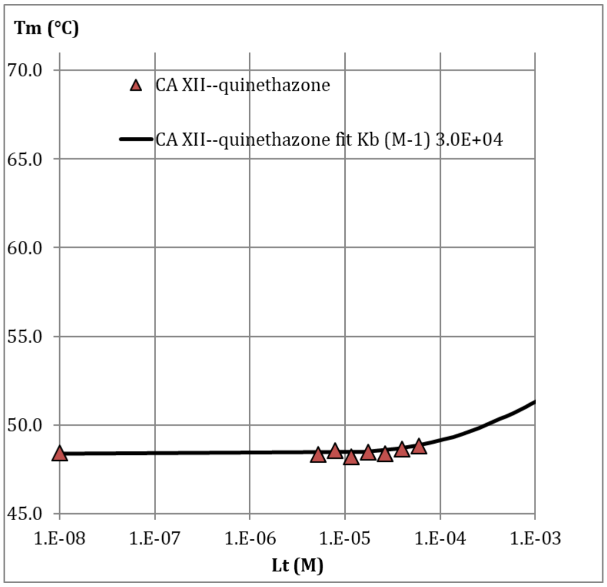
Tm (°C)
70.0
65.0
60.0
55.0
50.0
45.0
CA XII--quinethazone
CA XII--quinethazone fit Kb (M-1) 3.0E+04
1.E-08
1.E-07
1.E-06
1.E-05
1.E-04
1.E-03
Lt (M)

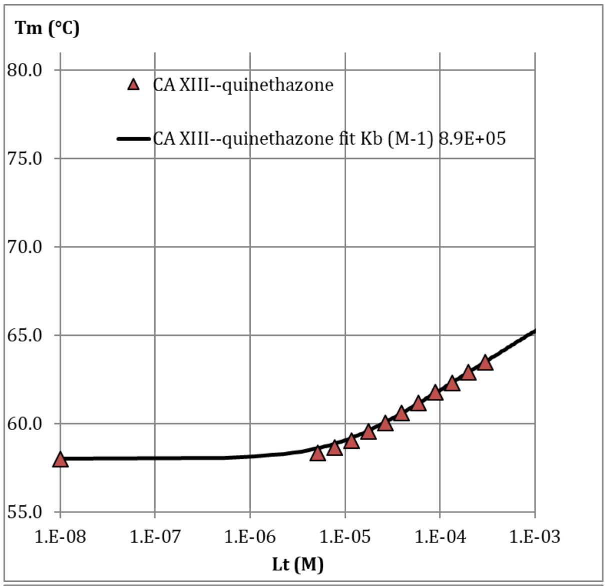
Tm (°C)
80.0
75.0
70.0
65.0
60.0
55.0
CA XIII--quinethazone
CA XIII--quinethazone fit Kb (M-1) 8.9E+05
1.E-08
1.E-07
1.E-06
1.E-05
1.E-04
1.E-03
Lt (M)

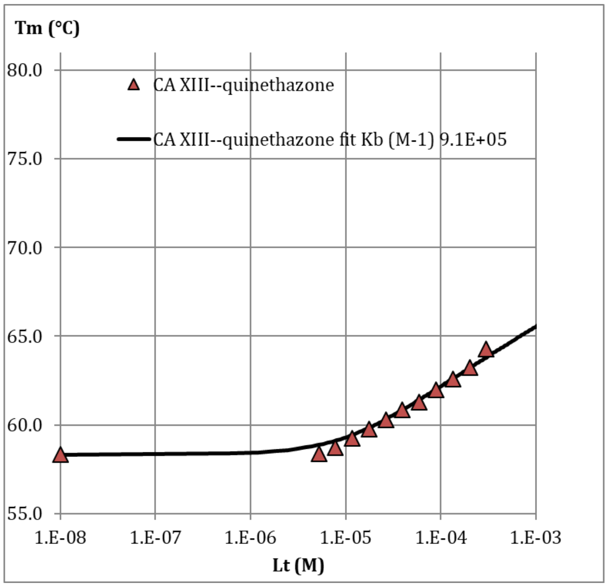
Tm (°C)
80.0
75.0
70.0
65.0
60.0
55.0
CA XIII--quinethazone
CA XIII--quinethazone fit Kb (M-1) 9.1E+05
1.E-08
1.E-07
1.E-06
1.E-05
1.E-04
1.E-03
Lt (M)

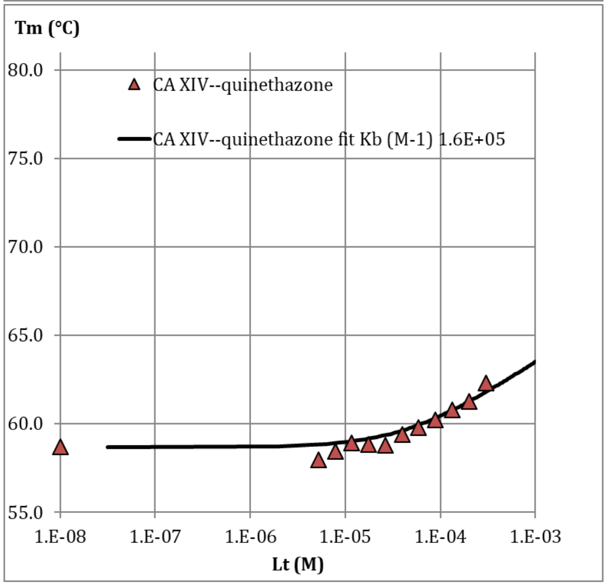
Tm (°C)
80.0
75.0
70.0
65.0
60.0
55.0
CA XIV--quinethazone
CA XIV--quinethazone fit Kb (M-1) 1.6E+05
1.E-08
1.E-07
1.E-06
1.E-05
1.E-04
1.E-03
Lt (M)

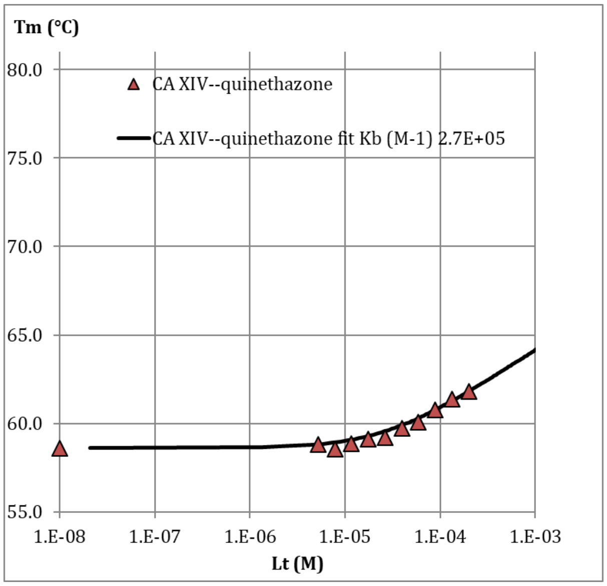
Tm (°C)
80.0
75.0
70.0
65.0
60.0
55.0
CA XIV--quinethazone
CA XIV--quinethazone fit Kb (M-1) 2.7E+05
1.E-08
1.E-07
1.E-06
1.E-05
1.E-04
1.E-03
Lt (M)

19. Fenquizone

Fluorecence-based thermal shift assay

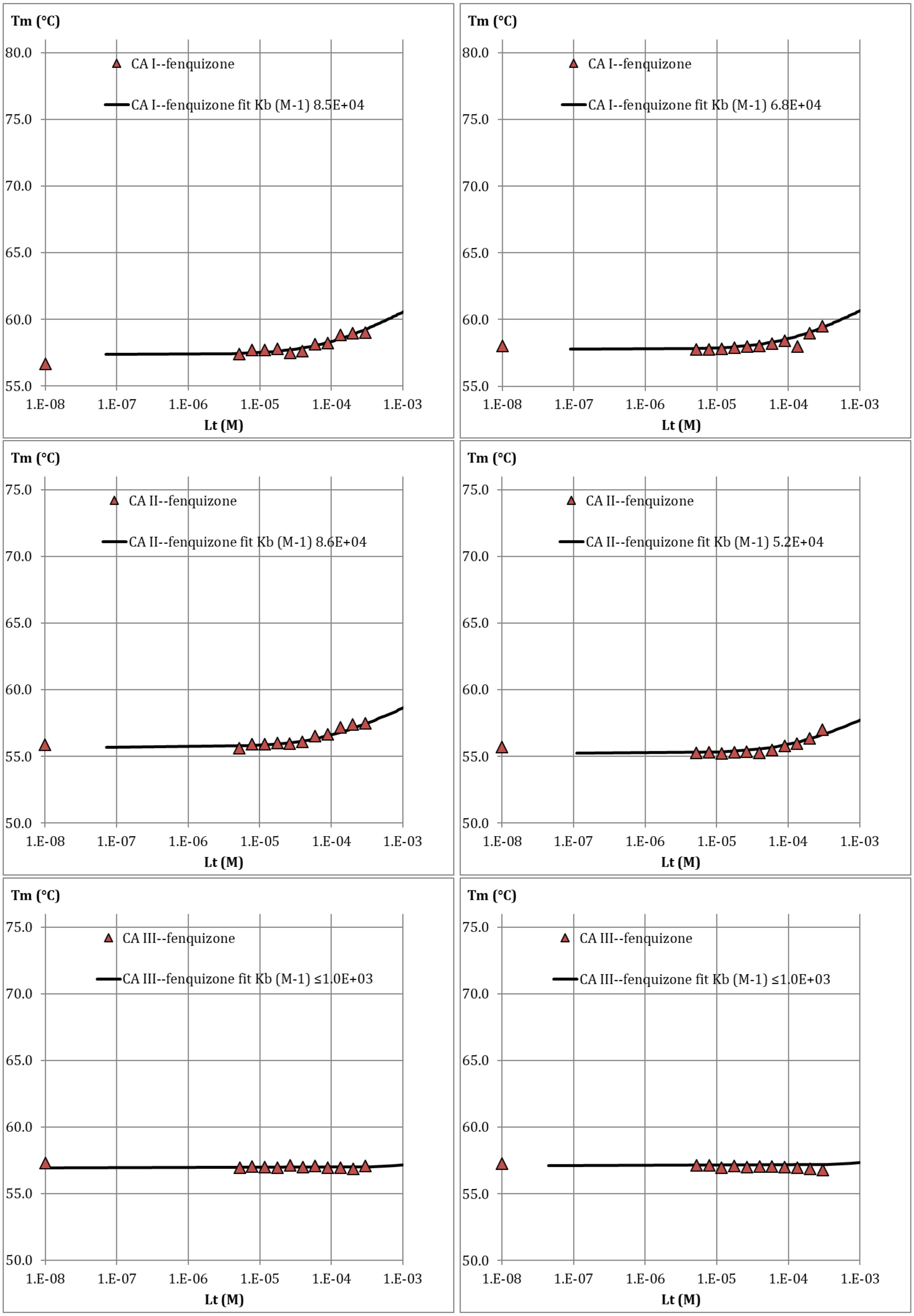

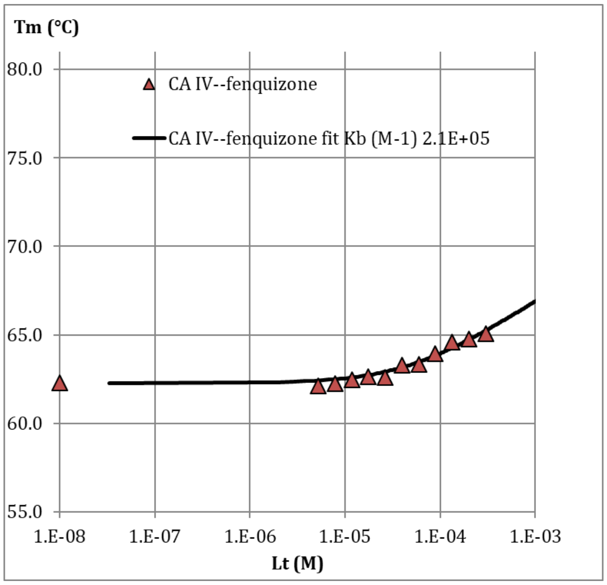
Tm (°C)
80.0
75.0
70.0
65.0
60.0
55.0
CA IV--fenquizone
CA IV--fenquizone fit Kb (M-1) 2.1E+05
1.E-08
1.E-07
1.E-06
1.E-05
1.E-04
1.E-03
Lt (M)

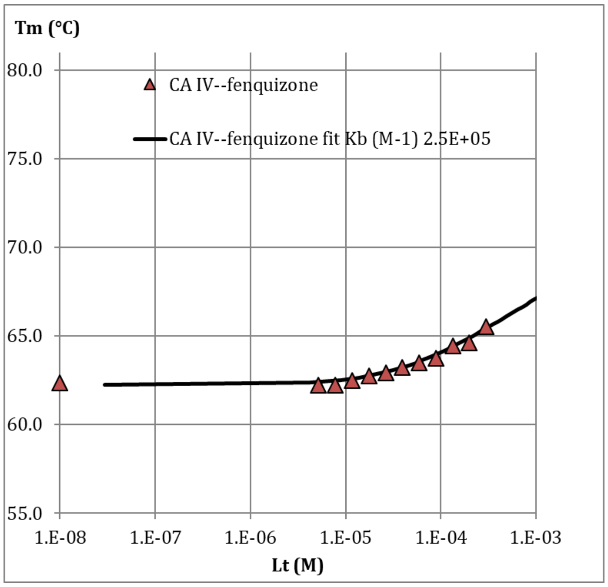
Tm (°C)
80.0
75.0
70.0
65.0
60.0
55.0
CA IV--fenquizone
CA IV--fenquizone fit Kb (M-1) 2.5E+05
1.E-08
1.E-07
1.E-06
1.E-05
1.E-04
1.E-03
Lt (M)

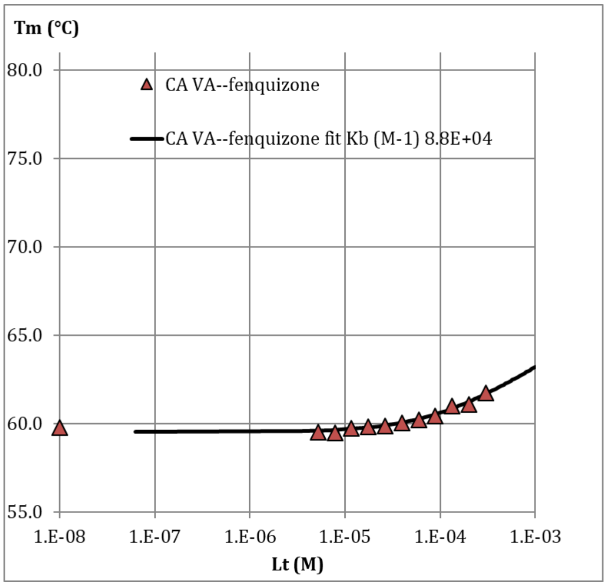
Tm (°C)
80.0
75.0
70.0
65.0
60.0
55.0
CA VA--fenquizone
CA VA--fenquizone fit Kb (M-1) 8.8E+04
1.E-08
1.E-07
1.E-06
1.E-05
1.E-04
1.E-03
Lt (M)

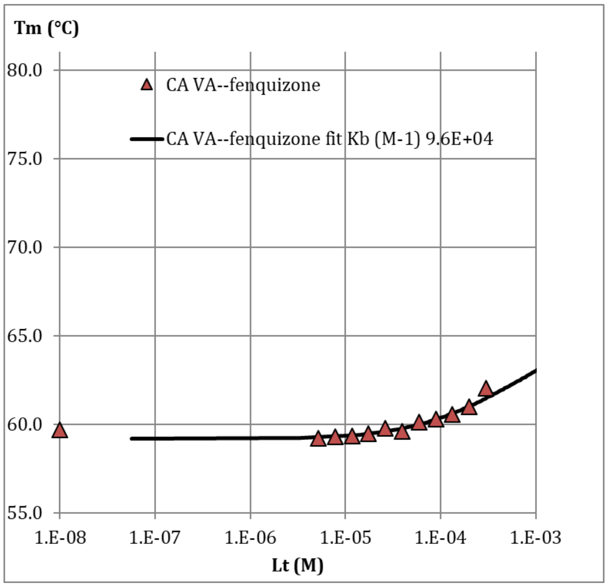
Tm (°C)
80.0
75.0
70.0
65.0
60.0
55.0
CA VA--fenquizone
CA VA--fenquizone fit Kb (M-1) 9.6E+04
1.E-08
1.E-07
1.E-06
1.E-05
1.E-04
1.E-03
Lt (M)

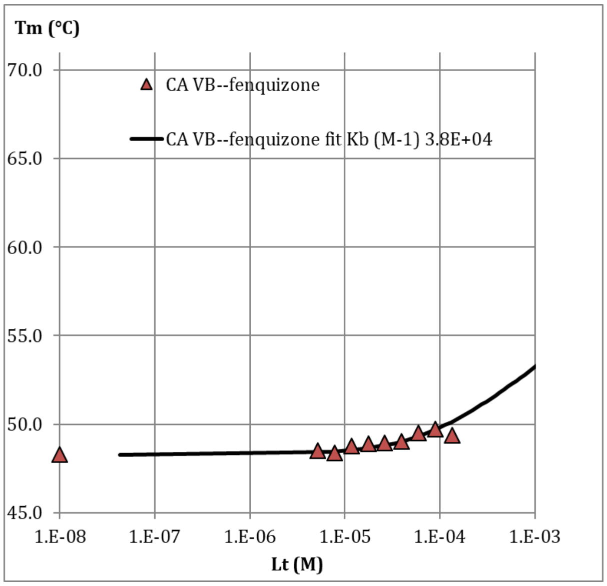
Tm (°C)
70.0
65.0
60.0
55.0
50.0
45.0
CA VB--fenquizone
CA VB--fenquizone fit Kb (M-1) 3.8E+04
1.E-08
1.E-07
1.E-06
1.E-05
1.E-04
1.E-03
Lt (M)

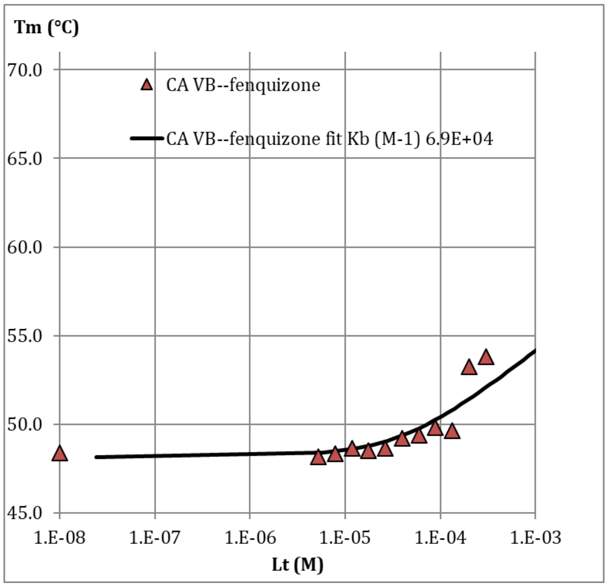
Tm (°C)
70.0
65.0
60.0
55.0
50.0
45.0
CA VB--fenquizone
CA VB--fenquizone fit Kb (M-1) 6.9E+04
1.E-08
1.E-07
1.E-06
1.E-05
1.E-04
1.E-03
Lt (M)

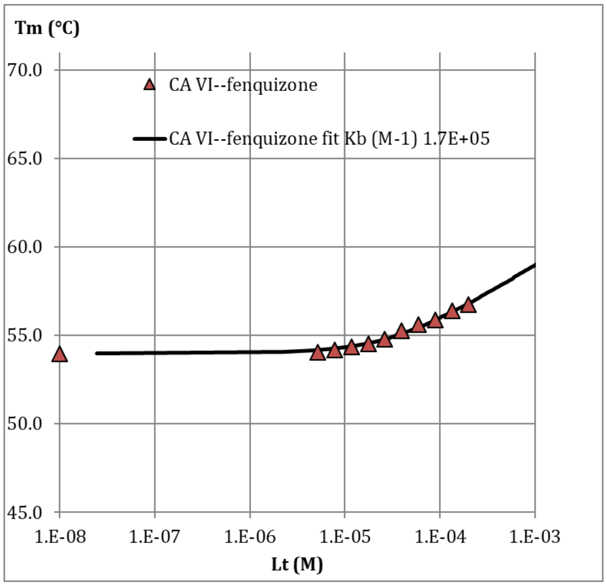
Tm (°C)
70.0
65.0
60.0
55.0
50.0
45.0
CA VI--fenquizone
CA VI--fenquizone fit Kb (M-1) 1.7E+05
1.E-08
1.E-07
1.E-06
1.E-05
1.E-04
1.E-03
Lt (M)

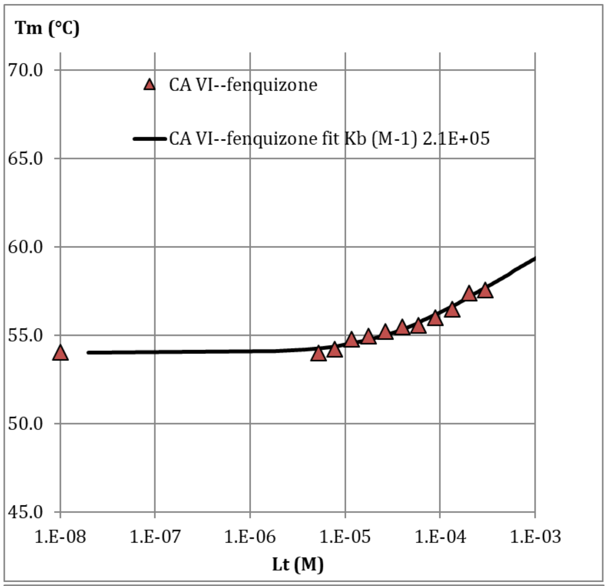
Tm (°C)
70.0
65.0
60.0
55.0
50.0
45.0
CA VI--fenquizone
CA VI--fenquizone fit Kb (M-1) 2.1E+05
1.E-08
1.E-07
1.E-06
1.E-05
1.E-04
1.E-03
Lt (M)

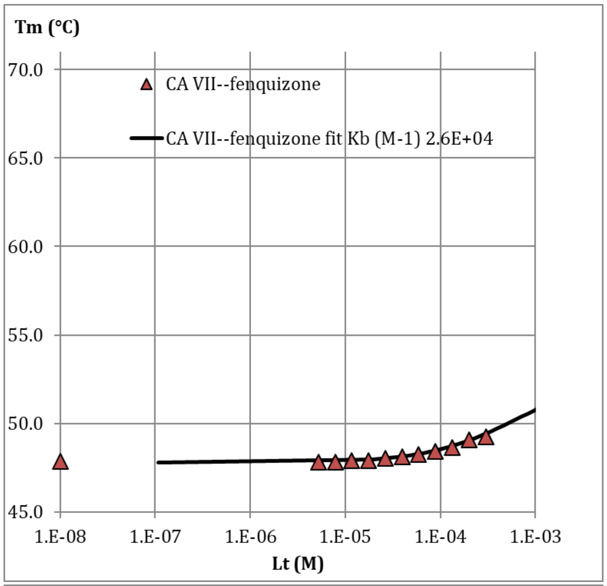
Tm (°C)
70.0
65.0
60.0
55.0
50.0
45.0
CA VII--fenquizone
CA VII--fenquizone fit Kb (M-1) 2.6E+04
1.E-08
1.E-07
1.E-06
1.E-05
1.E-04
1.E-03
Lt (M)

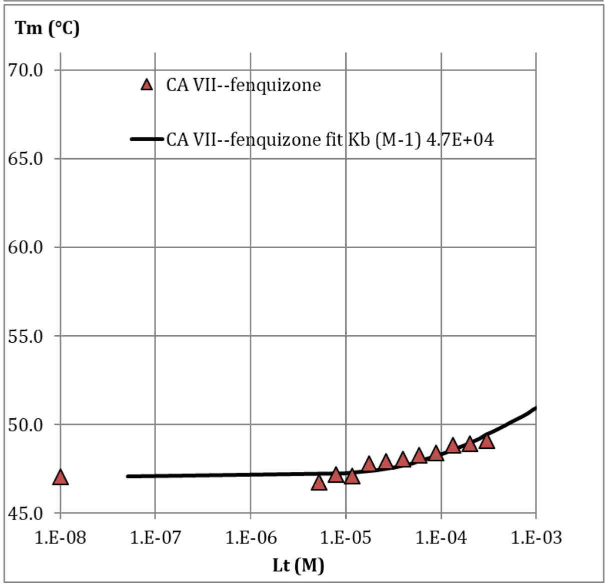
Tm (°C)
70.0
65.0
60.0
55.0
50.0
45.0
CA VII--fenquizone
CA VII--fenquizone fit Kb (M-1) 4.7E+04
1.E-08
1.E-07
1.E-06
1.E-05
1.E-04
1.E-03
Lt (M)

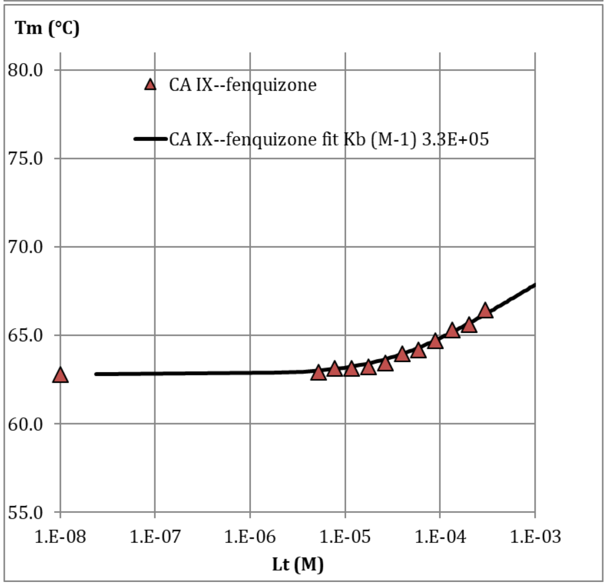
Tm (°C)
80.0
75.0
70.0
65.0
60.0
55.0
CA IX--fenquizone
CA IX--fenquizone fit Kb (M-1) 3.3E+05
1.E-08
1.E-07
1.E-06
1.E-05
1.E-04
1.E-03
Lt (M)

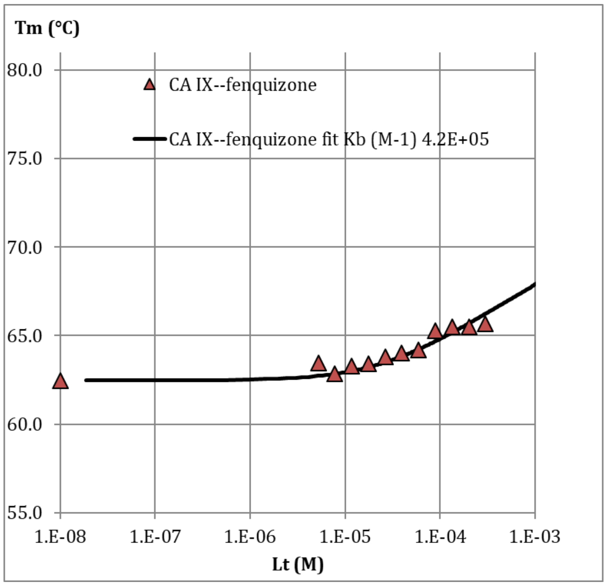
Tm (°C)
80.0
75.0
70.0
65.0
60.0
55.0
CA IX--fenquizone
CA IX--fenquizone fit Kb (M-1) 4.2E+05
1.E-08
1.E-07
1.E-06
1.E-05
1.E-04
1.E-03
Lt (M)

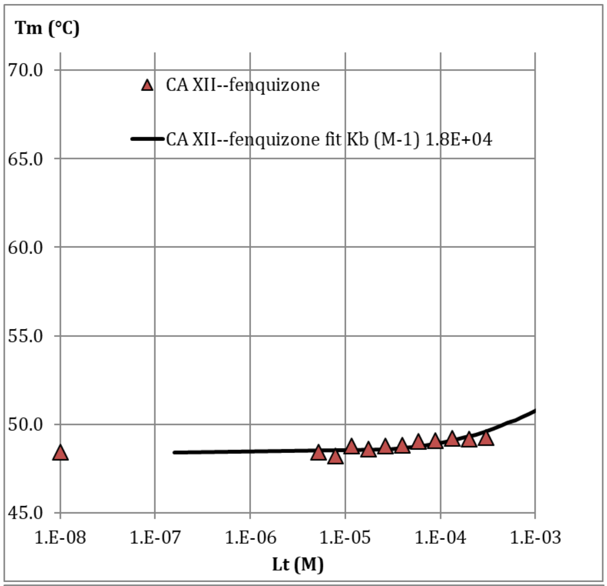
Tm (°C)
CA XII--fenquizone
CA XII--fenquizone fit Kb (M-1) 1.8E+04
70.0
65.0
60.0
55.0
50.0
45.0
1.E-08
1.E-07
1.E-06
1.E-05
1.E-04
1.E-03
Lt (M)

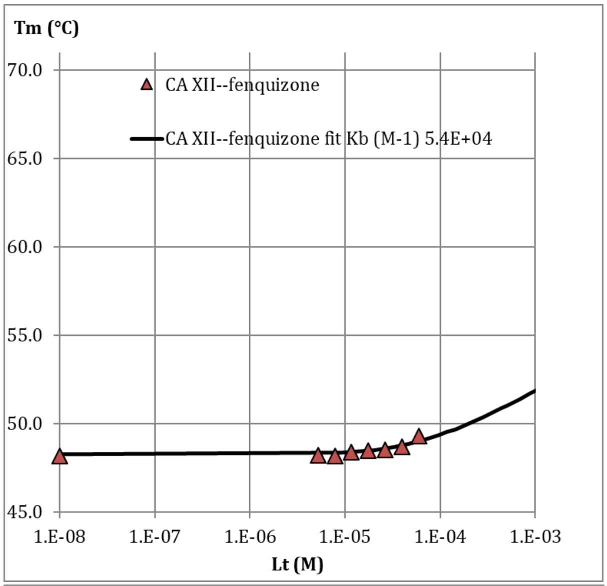
Tm (°C)
CA XII--fenquizone
CA XII--fenquizone fit Kb (M-1) 5.4E+04
70.0
65.0
60.0
55.0
50.0
45.0
1.E-08
1.E-07
1.E-06
1.E-05
1.E-04
1.E-03
Lt (M)

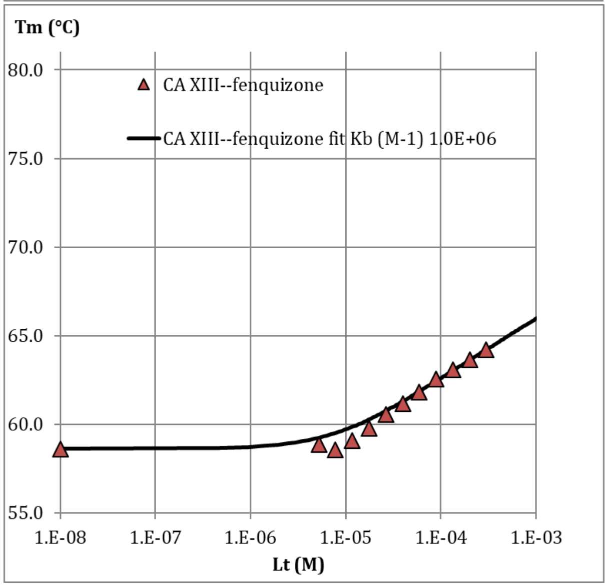
Tm (°C)
CA XIII--fenquizone
CA XIII--fenquizone fit Kb (M-1) 1.0E+06
80.0
75.0
70.0
65.0
60.0
55.0
1.E-08
1.E-07
1.E-06
1.E-05
1.E-04
1.E-03
Lt (M)

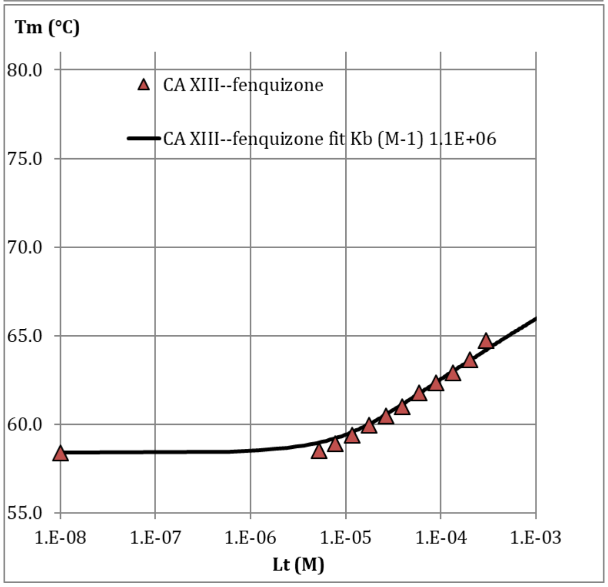
Tm (°C)
CA XIII--fenquizone
CA XIII--fenquizone fit Kb (M-1) 1.1E+06
80.0
75.0
70.0
65.0
60.0
55.0
1.E-08
1.E-07
1.E-06
1.E-05
1.E-04
1.E-03
Lt (M)

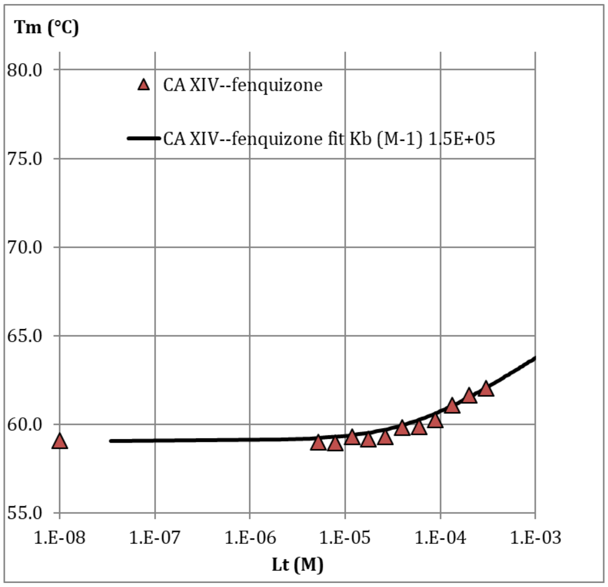
Tm (°C)
CA XIV--fenquizone
CA XIV--fenquizone fit Kb (M-1) 1.5E+05
80.0
75.0
70.0
65.0
60.0
55.0
1.E-08
1.E-07
1.E-06
1.E-05
1.E-04
1.E-03
Lt (M)

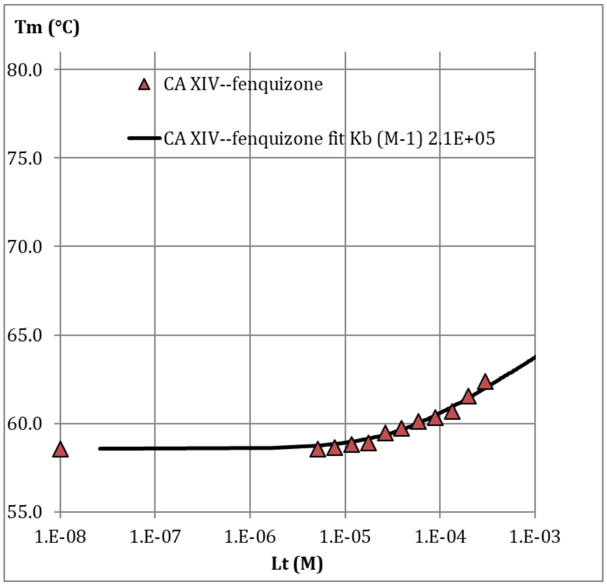
Tm (°C)
CA XIV--fenquizone
CA XIV--fenquizone fit Kb (M-1) 2.1E+05
80.0
75.0
70.0
65.0
60.0
55.0
1.E-08
1.E-07
1.E-06
1.E-05
1.E-04
1.E-03
Lt (M)

20. Metolazone

Fluorecence-based thermal shift assay

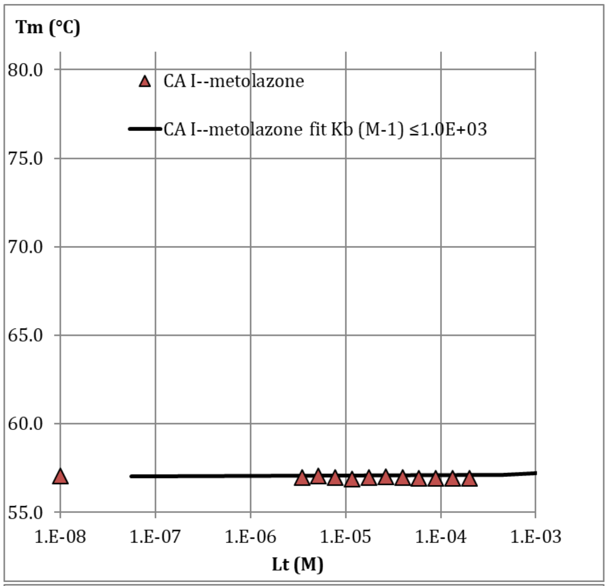

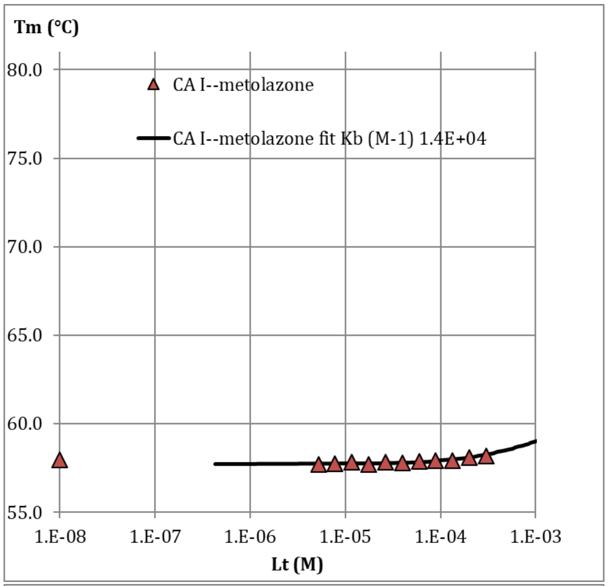

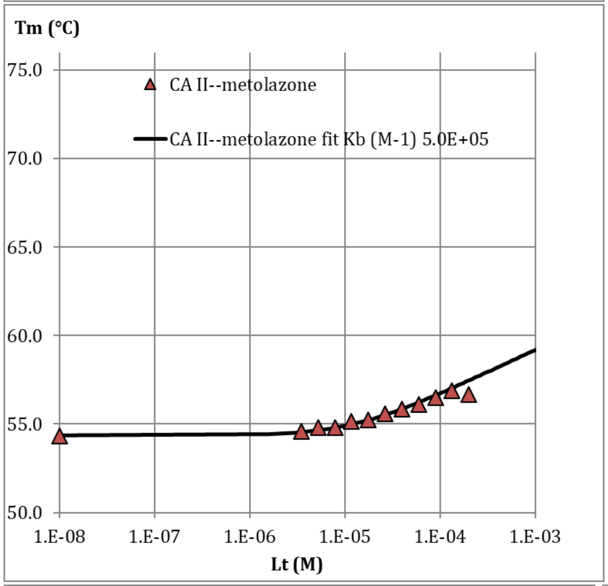

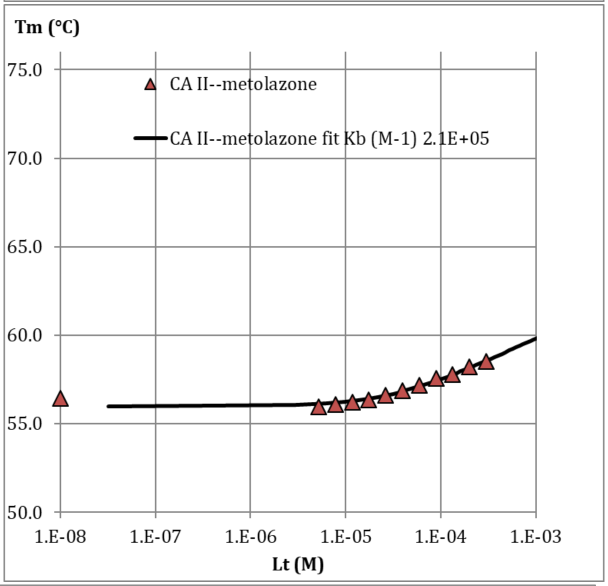

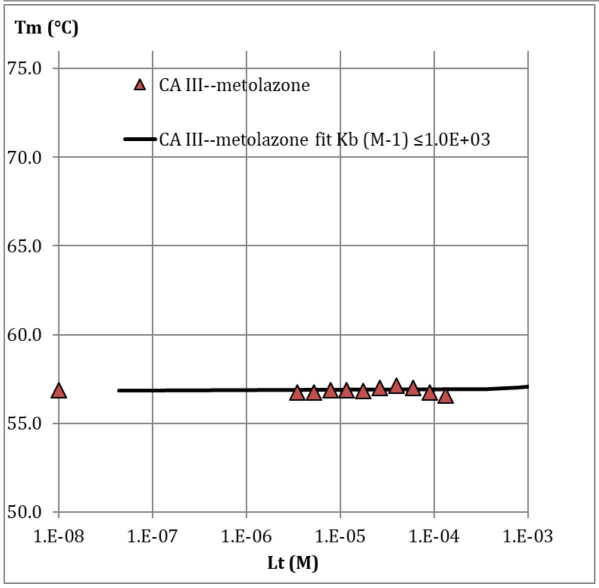

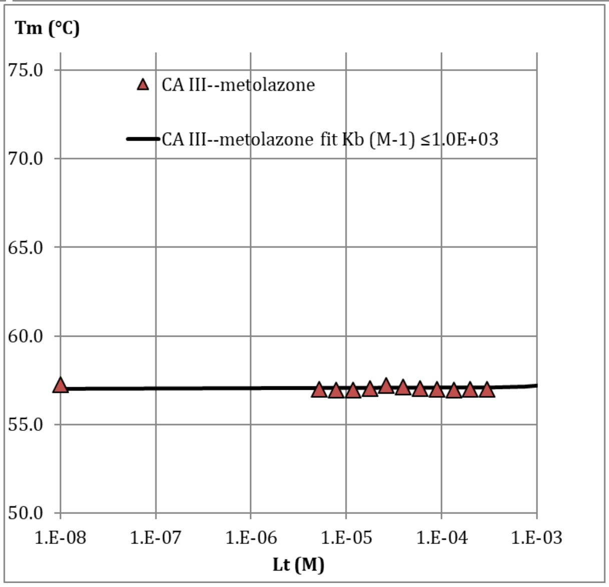

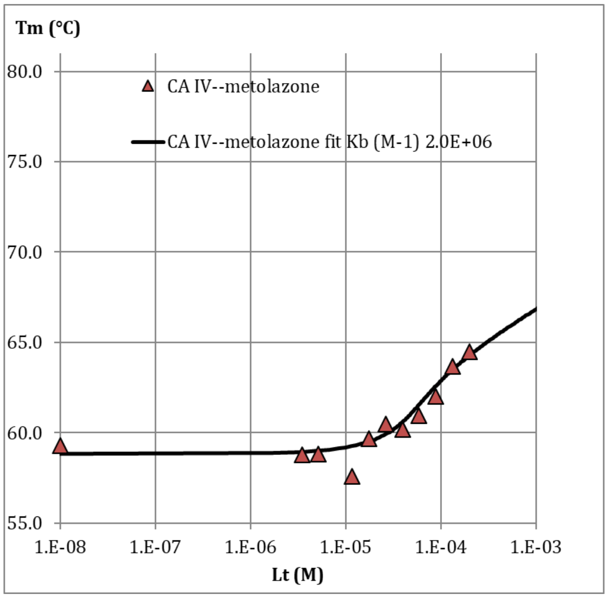
Tm (°C)
80.0
75.0
70.0
65.0
60.0
55.0
CA IV--metolazone
CA IV--metolazone fit Kb (M-1) 2.0E+06
1.E-08
1.E-07
1.E-06
1.E-05
1.E-04
1.E-03
Lt (M)

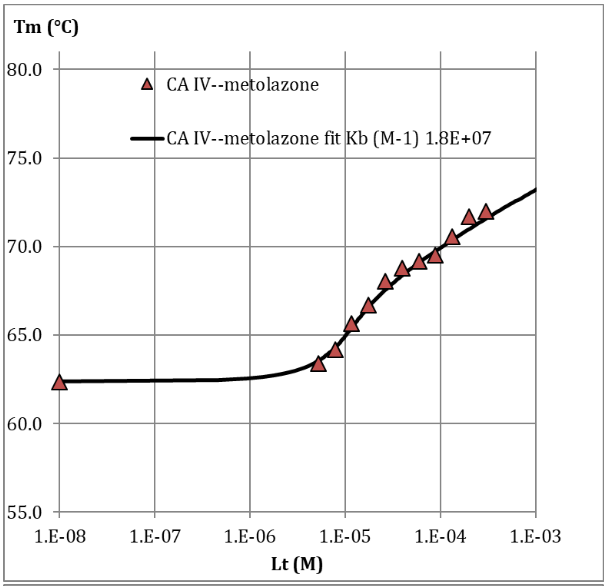
Tm (°C)
80.0
75.0
70.0
65.0
60.0
55.0
CA IV--metolazone
CA IV--metolazone fit Kb (M-1) 1.8E+07
1.E-08
1.E-07
1.E-06
1.E-05
1.E-04
1.E-03
Lt (M)

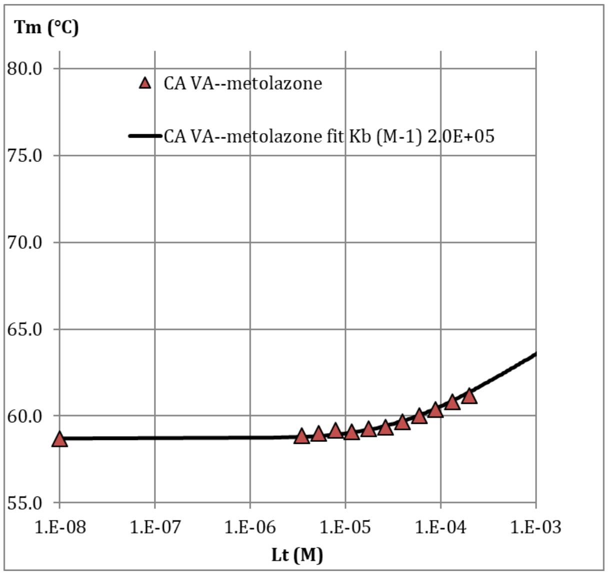
Tm (°C)
80.0
75.0
70.0
65.0
60.0
55.0
CA VA--metolazone
CA VA--metolazone fit Kb (M-1) 2.0E+05
1.E-08
1.E-07
1.E-06
1.E-05
1.E-04
1.E-03
Lt (M)

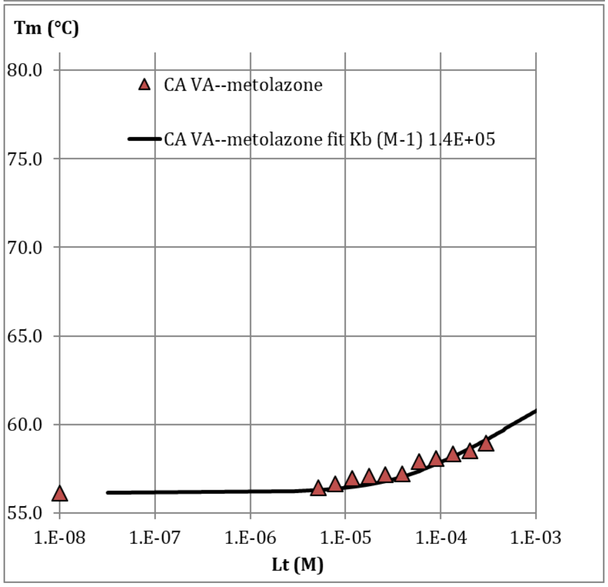
Tm (°C)
80.0
75.0
70.0
65.0
60.0
55.0
CA VA--metolazone
CA VA--metolazone fit Kb (M-1) 1.4E+05
1.E-08
1.E-07
1.E-06
1.E-05
1.E-04
1.E-03
Lt (M)

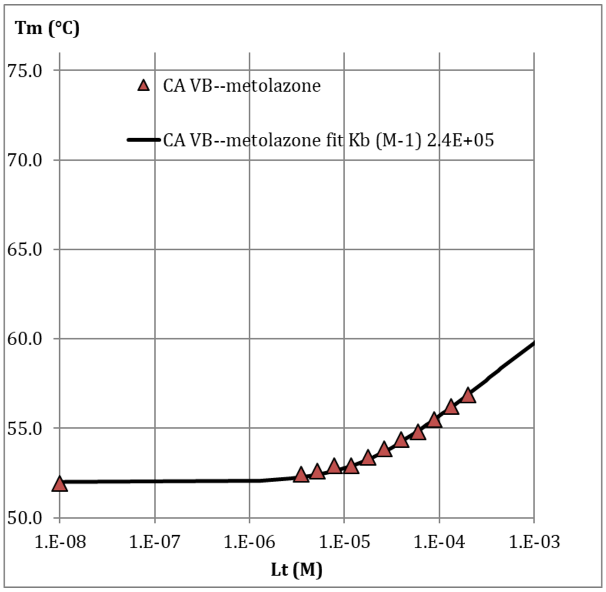
Tm (°C)
75.0
70.0
65.0
60.0
55.0
50.0
CA VB--metolazone
CA VB--metolazone fit Kb (M-1) 2.4E+05
1.E-08
1.E-07
1.E-06
1.E-05
1.E-04
1.E-03
Lt (M)

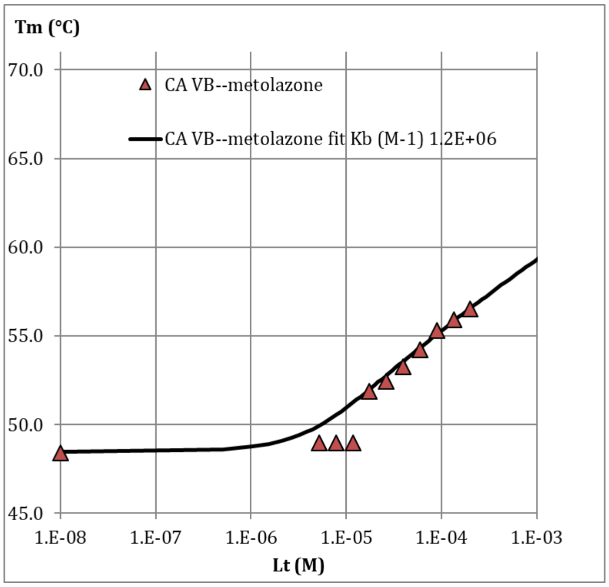
Tm (°C)
70.0
65.0
60.0
55.0
50.0
45.0
CA VB--metolazone
CA VB--metolazone fit Kb (M-1) 1.2E+06
1.E-08
1.E-07
1.E-06
1.E-05
1.E-04
1.E-03
Lt (M)

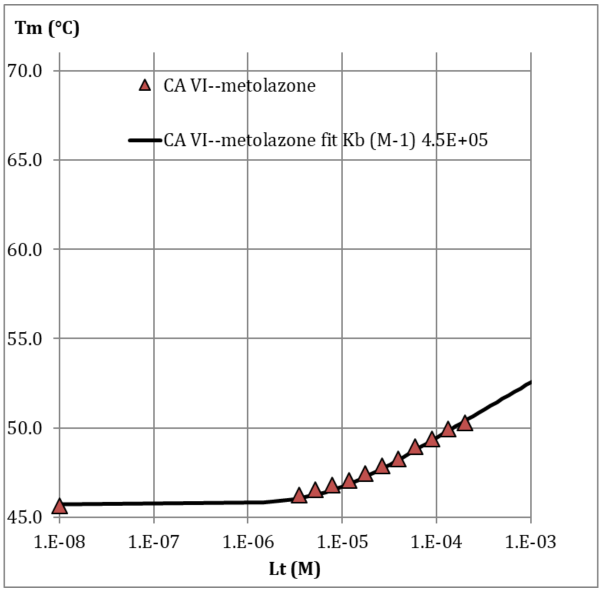

Tm (°C)
70.0
65.0
60.0
55.0
50.0
45.0
CA VI--metolazone
CA VI--metolazone fit Kb (M-1) 4.5E+05
1.E-08
1.E-07
1.E-06
1.E-05
1.E-04
1.E-03
Lt (M)

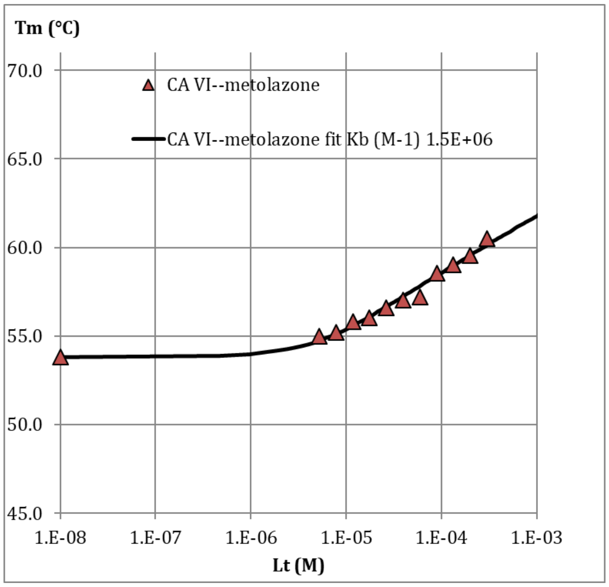

Tm (°C)
70.0
65.0
60.0
55.0
50.0
45.0
CA VI--metolazone
CA VI--metolazone fit Kb (M-1) 1.5E+06
1.E-08
1.E-07
1.E-06
1.E-05
1.E-04
1.E-03
Lt (M)

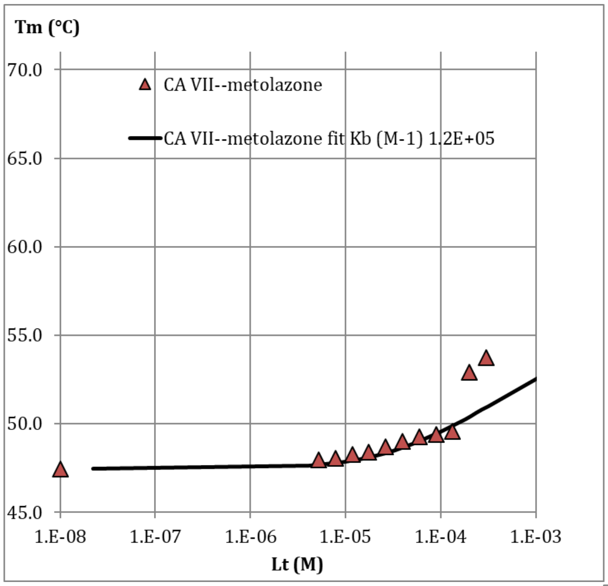

Tm (°C)
70.0
65.0
60.0
55.0
50.0
45.0
CA VII--metolazone
CA VII--metolazone fit Kb (M-1) 1.2E+05
1.E-08
1.E-07
1.E-06
1.E-05
1.E-04
1.E-03
Lt (M)

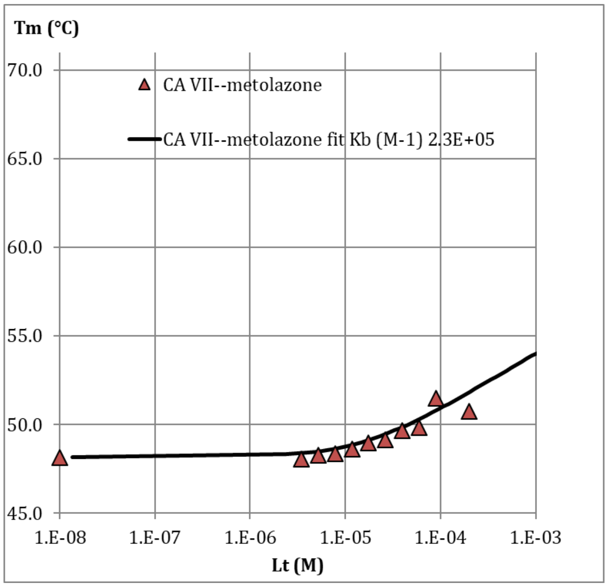

Tm (°C)
70.0
65.0
60.0
55.0
50.0
45.0
CA VII--metolazone
CA VII--metolazone fit Kb (M-1) 2.3E+05
1.E-08
1.E-07
1.E-06
1.E-05
1.E-04
1.E-03
Lt (M)

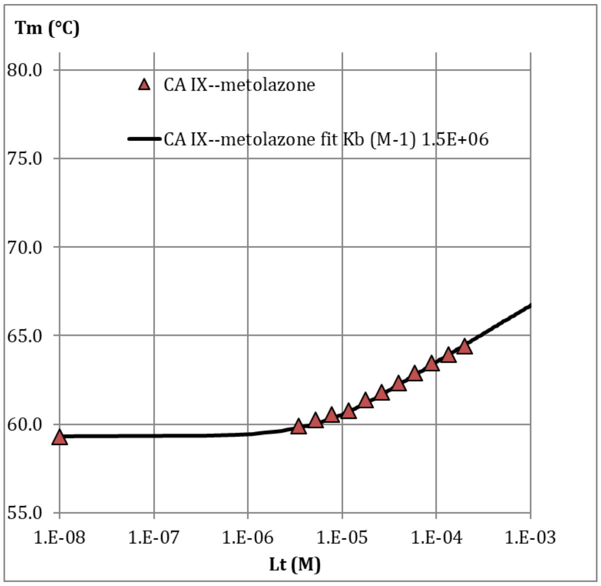

Tm (°C)
80.0
75.0
70.0
65.0
60.0
55.0
CA IX--metolazone
CA IX--metolazone fit Kb (M-1) 1.5E+06
1.E-08
1.E-07
1.E-06
1.E-05
1.E-04
1.E-03
Lt (M)

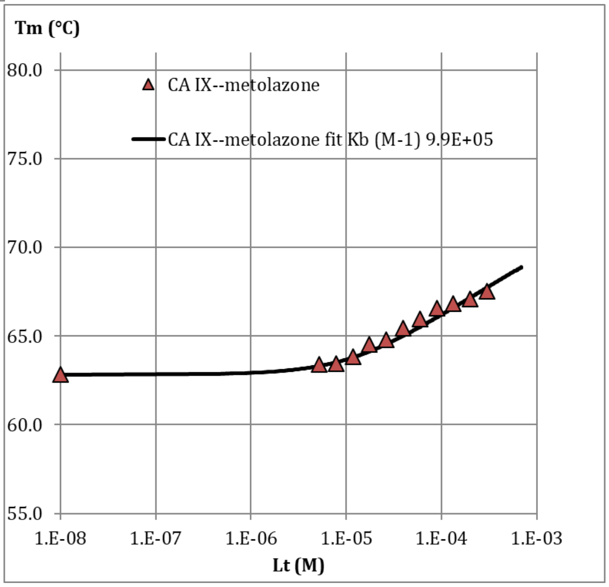

Tm (°C)
80.0
75.0
70.0
65.0
60.0
55.0
CA IX--metolazone
CA IX--metolazone fit Kb (M-1) 9.9E+05
1.E-08
1.E-07
1.E-06
1.E-05
1.E-04
1.E-03
Lt (M)

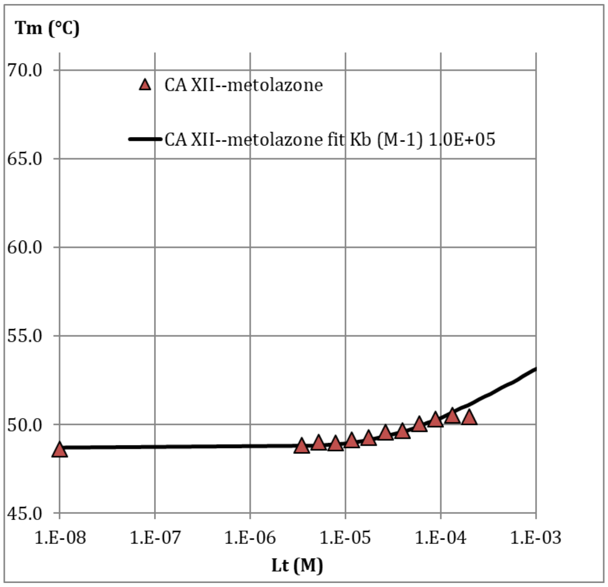

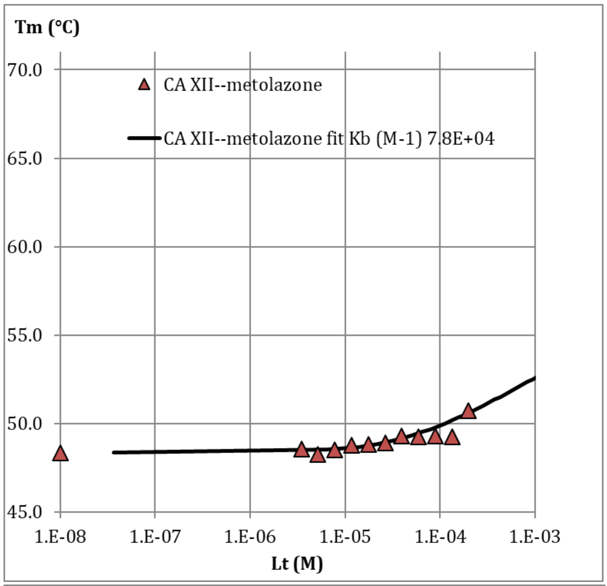

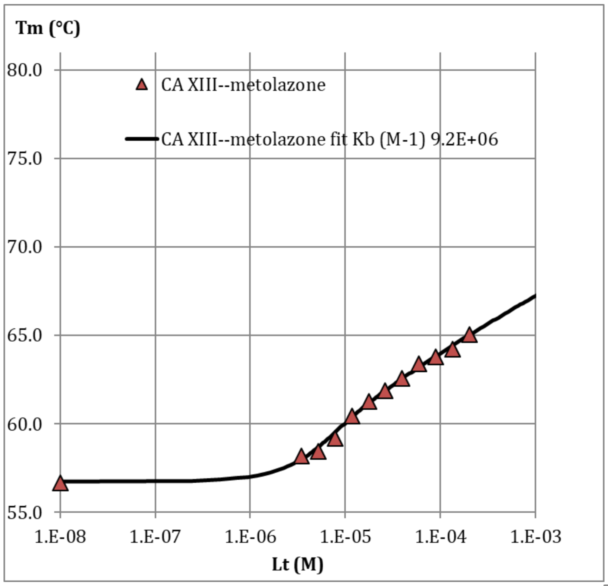

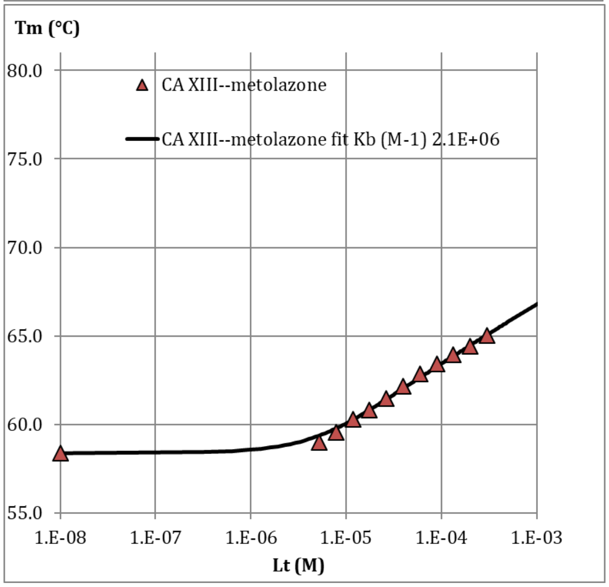

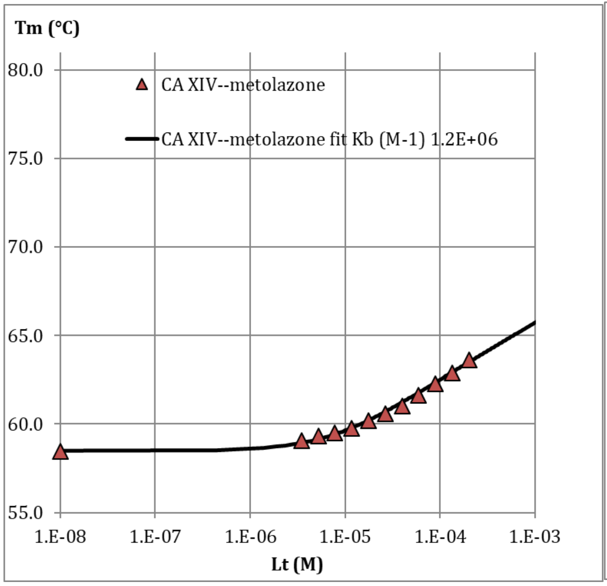

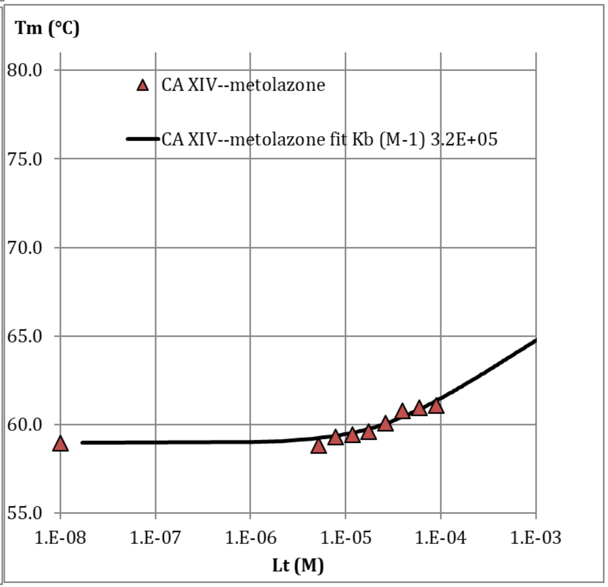

Note: T_m variations for the same protein depend on exact protein concentration in the experiment and vary within approx. +/-3 degrees, except for CA VI – in this case, protein preparations produced in bacterial (less stable) or eucaryotic (more stable) expression systems were used [1].

Equations used to fit the fluorescence-based thermal shift assay (FTSA) data

Equations used to fit the fluorescent thermal shift assay (FTSA) data are provided in this section describe the model for ligand-induced protein stabilization against thermal denaturation. Fluorescence yield, f, upon protein unfolding is

$$f = f_N X_N + f_U X_U, \tag{1}$$

where f_N and X_N are the fluorescence yield and fraction of the native form of a protein, f_U and X_U are those of the unfolded form. We consider native (N) and unfolded (U) states of a protein. The molar fractions of native and unfolded protein

$$X_N = \frac{[\mathrm{N}]}{[\mathrm{N}]+[\mathrm{U}]}, \tag{2}$$

$$X_U = \frac{[\mathrm{U}]}{[\mathrm{N}]+[\mathrm{U}]} \tag{3}$$

allow to obtain the following equation of fluorescence yield:

$$f = f_N + \frac{f_U - f_N}{1 + \frac{[\mathrm{N}]}{[\mathrm{U}]}}. \tag{4}$$

The fluorescence yields in the native and unfolded states are usually temperature-dependent and can be approximated by linear equations:

$$f_N = f_{N\_T_m} + m_N(T - T_m), \tag{5}$$

$$f_U = f_{U\_T_m} + m_U(T - T_m), \tag{6}$$

where m_N and m_U are slopes in the pre- and post-transition states, while $f_{N\_T_m}$ and $f_{U\_T_m}$ are the levels of fluorescence yields of the native and unfolded protein states at the melting temperature, T_m. Substituting f_N and f_U terms in Equation (4) by their temperature dependencies (Equations (5) and (6)) we obtain the final equation for the fluorescence yield:

$$f = f_{N\_T_m} + m_N(T - T_m) + \frac{f_{U\_T_m} - f_{N\_T_m} + (m_U - m_N)(T - T_m)}{1 + \frac{[\mathrm{N}]}{[\mathrm{U}]}}. \tag{7}$$

In the Fluorescent Thermal Shift Assay (FTSA), a protein stabilization is described by the shift in its melting temperature upon addition of a ligand. Mathematically this phenomenon requires the equation that relates the concentration of added ligand with the changes in the Gibbs energy, enthalpy, and entropy of protein unfolding and protein-ligand binding. To derive this relationship, we start with four equations:

$$[\mathrm{P}]_t = [\mathrm{N}] + [\mathrm{P \cdot L}] + [\mathrm{U}], \tag{8}$$

$$[\mathrm{L}]_t = [\mathrm{L}] + [\mathrm{P \cdot L}], \tag{9}$$

$$K_U = \frac{[\mathrm{U}]}{[\mathrm{N}]}, \tag{10}$$

$$K_b = \frac{[\mathrm{P \cdot L}]}{[\mathrm{N}][\mathrm{L}]}, \tag{11}$$

where K_U and K_b are equilibrium constants of protein unfolding and protein-ligand binding, respectively; $[\mathrm{P \cdot L}]$, $[\mathrm{P}]_t$ and $[\mathrm{L}]_t$ denotes the molar concentrations of protein-ligand complex, initial protein

and initial ligand, respectively. Unbound ligand molarity, [L], in the Equation (9) can be substituted by $[\mathrm{L}] = \frac{[\mathrm{P}\cdot\mathrm{L}]}{K_b[\mathrm{N}]}$ from Equation (11). We also make use of the relation $[\mathrm{N}] = \frac{[\mathrm{U}]}{K_U}$ from Equation (10), which leads to

$$[\mathrm{L}]_t = \frac{K_U[\mathrm{P}\cdot\mathrm{L}]}{K_b[\mathrm{U}]} + [\mathrm{P}\cdot\mathrm{L}] = [\mathrm{P}\cdot\mathrm{L}]\left(\frac{K_U}{K_b[\mathrm{U}]} + 1\right). \tag{12}$$

Equations (8) and (10) are combined to obtain the molarity of protein-ligand complex

$$[\mathrm{P}\cdot\mathrm{L}] = [\mathrm{P}]_t - [\mathrm{U}] - \frac{[\mathrm{U}]}{K_U}. \tag{13}$$

Writing the $[\mathrm{P}\cdot\mathrm{L}]$ term into Equation (12) results in:

$$[\mathrm{L}]_t = \left([\mathrm{P}]_t - [\mathrm{U}] - \frac{[\mathrm{U}]}{K_U}\right)\left(\frac{K_U}{K_b[\mathrm{U}]} + 1\right). \tag{14}$$

We describe the temperature dependencies of equilibrium constants K_U and K_b in terms of the standard Gibbs energies of protein unfolding, ΔG°_U, and protein–ligand binding, ΔG°_b:

$$K_U(T) = \exp\left(-\frac{\Delta G^\circ_U}{RT}\right), \tag{15}$$

$$K_b(T) = \exp\left(-\frac{\Delta G^\circ_b}{RT}\right), \tag{16}$$

where R is the molar gas constant. The change in Gibbs energy has two constituent parts: enthalpy (ΔH) and entropy (ΔS). Both terms are temperature-dependent and their standard values at a particular temperature, T, can be calculated using equations

$$\Delta H^\circ_x = \Delta H^\circ_{x\_T_r} + \int_{T_r}^{T} \Delta C_{p\_x} \mathrm{d}T, \tag{17}$$

$$\Delta S^\circ_x = \Delta S^\circ_{x\_T_r} + \int_{T_r}^{T} \left(\frac{\Delta C_{p\_x}}{T}\right) \mathrm{d}T, \tag{18}$$

where T_r is a reference (initial) temperature, $\Delta H^\circ_{x\_T_r}$ and $\Delta S^\circ_{x\_T_r}$ – standard enthalpy and entropy at T_r, ΔC_{p_x} – the change in constant pressure heat capacity. The subscript x in Equations (17) and (18) stands for either unfolding or binding process. If ΔC_p is constant in the temperature range from T_r to T_m, we can integrate Equations (17) and (18) and the resulting Gibbs energy is

$$\begin{aligned}\Delta G^\circ_x &= \Delta H^\circ_x - T_m \Delta S^\circ_x = \\ &= \Delta H^\circ_{x\_T_r} + \Delta C_{p\_x}(T_m - T_r) - T_m\left(\Delta S^\circ_{x\_T_r} + \Delta C_{p\_x} \ln\frac{T_m}{T_r}\right).\end{aligned} \tag{19}$$

Folded and unfolded fractions of a protein are equal at melting temperature ($[\mathrm{U}] = \frac{[\mathrm{P}]_t}{2}$ at T_m), thus Equation (14) simplifies, and together with Equations (15) and (16) provides the mathematical background of the FTSA:

$$\begin{cases} [\mathrm{L}]_t = (K_U - 1)\left(\frac{1}{K_b} + \frac{[\mathrm{P}]_t}{2K_U}\right), \\ K_U = \exp\left(-\frac{\Delta H^\circ_{U\_T_r} + \Delta C_{p\_U}(T_m - T_r) - T_m\left(\Delta S^\circ_{U\_T_r} + \Delta C_{p\_U}\ln\frac{T_m}{T_r}\right)}{RT_m}\right), \\ K_b = \exp\left(-\frac{\Delta H^\circ_{b\_T_0} + \Delta C_{p\_b}(T_m - T_0) - T_m\left(\Delta S^\circ_{b\_T_0} + \Delta C_{p\_b}\ln\frac{T_m}{T_0}\right)}{RT_m}\right). \end{cases} \tag{20}$$

Equation used to fit stopped-flow enzymatic inhibition assay (SFA) data

This equation is also known as Morrison's equation [2,3] or Greco & Hakala equation [4,5], which is used when enzyme concentration in the assay is comparable with inhibition constant, as is in the case of carbonic anhydrase inhibition:

$$CA\,activity = 1 - \frac{([CA] + [I] + K_d) - \sqrt{([CA] + [I] + K_d)^2 - 4[CA][I]}}{2[CA]}$$

Where [CA] is the total concentration of CA, [I] is the total concentration of added inhibitor, and K_d is the apparent inhibition constant, which is equal to the protein – ligand dissociation constant in 1:1 binding model.

References

1. Kazokaitė J, Kairys V, Smirnovienė J, Smirnov A, Manakova E, Tolvanen M, et al. Engineered Carbonic Anhydrase VI-Mimic Enzyme Switched the Structure and Affinities of Inhibitors. Sci Rep. 2019;9: 12710. doi:10.1038/s41598-019-49094-0

2. Morrison JF. Kinetics of the reversible inhibition of enzyme-catalysed reactions by tight-binding inhibitors. Biochim Biophys Acta BBA - Enzymol. 1969;185: 269–286. doi:10.1016/0005-2744(69)90420-3

3. Copeland. Evaluation of Enzyme Inhibitors in Drug Discovery a Guide for Medicinal Chemists and Pharmacologists. JOHN WILEY & SONS, INC.; 2013.

4. Greco WR, Hakala MT. Evaluation of methods for estimating the dissociation constant of tight binding enzyme inhibitors. J Biol Chem. 1979;254: 12104–12109.

5. Kuzmic P. History, variants and usage of the "Morrison equation" in enzyme inhibition kinetics, BioKin Technical Note TN-2015-01, BioKin Ltd., Watertown MA, [Online] www.biokin.com/TN/2015/01. 2015 [cited 12 Oct 2018]. Available: http://www.biokin.com/publications/technotes/TN201501.html
